# Desymmetrization of Cyclic 1,3-Diketones under *N*-Heterocyclic Carbene Organocatalysis: Access to Organofluorines with Multiple Stereogenic Centers

**DOI:** 10.34133/2021/9867915

**Published:** 2021-08-23

**Authors:** Guanjie Wang, Min Zhang, Yezhi Guan, Ye Zhang, Xianfang Hong, Chenlong Wei, Pengcheng Zheng, Donghui Wei, Zhenqian Fu, Yonggui Robin Chi, Wei Huang

**Affiliations:** ^1^Key Laboratory of Flexible Electronics (KLOFE) & Institute of Advanced Materials (IAM) Nanjing Tech University (NanjingTech), 30 South Puzhu Road, Nanjing 211816, China; ^2^College of Chemistry and Green Catalysis Center, Zhengzhou University, Zhengzhou, Henan 450001, China; ^3^School of Chemistry and Molecular Engineering, Nanjing Tech University (NanjingTech), 30 South Puzhu Road, Nanjing 211816, China; ^4^Laboratory Breeding Base of Green Pesticide and Agricultural Bioengineering, Key Laboratory of Green Pesticide and Agricultural Bioengineering, Ministry of Education, Guizhou University, Huaxi District, Guiyang 550025, China; ^5^Division of Chemistry & Biological Chemistry, School of Physical & Mathematical Sciences, Nanyang Technological University, Singapore, Singapore 637371; ^6^Frontiers Science Center for Flexible Electronics (FSCFE) & Shaanxi Institute of Flexible Electronics (SIFE), Northwestern Polytechnical University (NPU), 127 West Youyi Road, Xi'an 710072, China

## Abstract

Symmetric 1,3-diketones with fluorine or fluorinated substituents on the prochiral carbon remain to be established. Herein, we have developed a novel prochiral fluorinated oxindanyl 1,3-diketone and successfully applied these substrates in carbene-catalyzed asymmetric desymmetrization. Accordingly, a versatile strategy for asymmetric generation of organofluorines with fluorine or fluorinated methyl groups has been developed. Multiple stereogenic centers were selectively constructed with satisfactory outcomes. Structurally diverse enantioenriched organofluorines were generated with excellent results in terms of yields, diastereoselectivities, and enantioselectivities. Notably, exchanging fluorinated methyl groups to fluorine for this prochiral 1,3-diketones leads to switchable stereoselectivity. Mechanistic aspects and origin of stereoselectivity were studied by DFT calculations. Notably, some of the prepared organofluorines demonstrated competitive antibacterial activities.

## 1. Introduction

**Figure 1:**
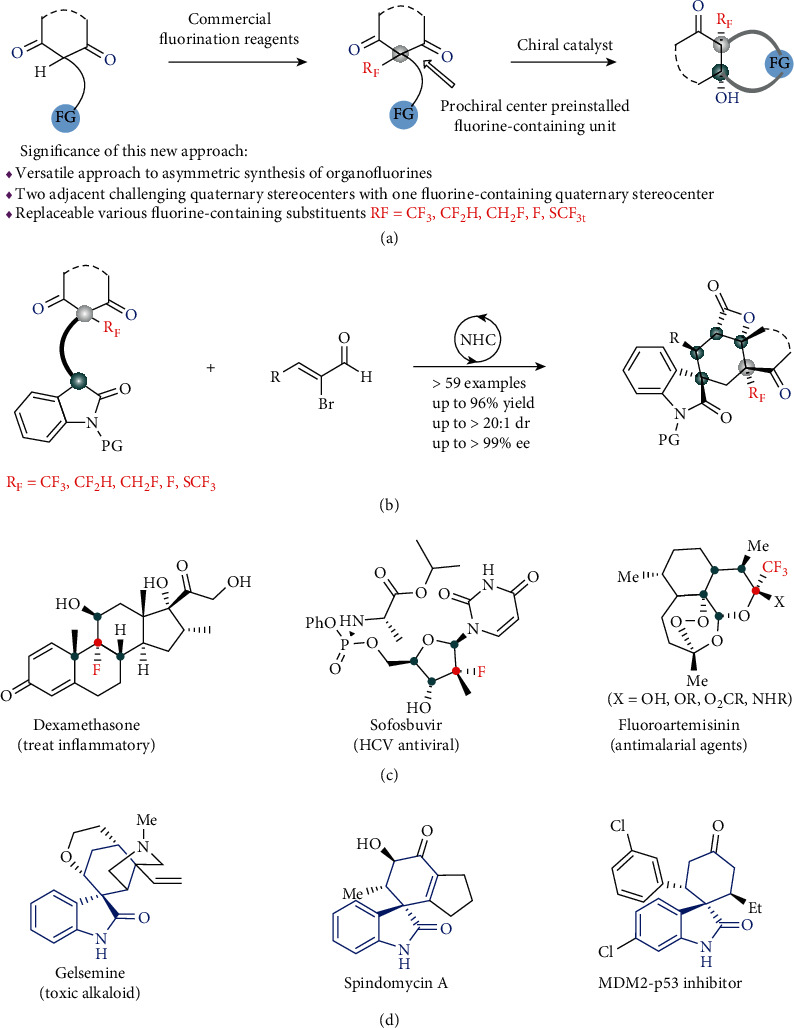
Asymmetric desymmetrization of cyclic 1,3-diketone with fluorine functionalized groups: (a) our design on introducing fluorine or fluorinated substituents into symmetric 1,3-diketones; (b) this work: NHC-catalyzed asymmetric desymmetrization of fluorine functionalized 1,3-diketones; (c) biologically active molecules with multiple stereogenic centers containing fluorine-containing quaternary stereocenter; (d) naturally occurring and biologically active spirocyclohexane oxindoles.

Asymmetric desymmetrization represents one of the most facile and efficient methods for the generation of enantioenriched organic compounds, especially with multiple stereogenic centers, from *meso* or prochiral raw materials [1–4]. In this area, catalytic desymmetrization of prochiral 1,3-diketones has been investigated widely, including asymmetric reduction, intramolecular adol-type reactions, and others [5–21]. Furthermore, this desymmetric strategy as the key step has shown wide application in diverse natural product synthesis associated with diverse promising biological activities [22, 23]. Owing to privileged structural characters of prochiral 1,3-diketones, such as containing unique and versatile carbonyl groups, and easily introducing substituents, further development of novel diverse prochiral diketones and their desymmetric strategies is still of high importance. Organic molecules with fluorine or fluorinated substituents can significantly change their physical, chemical, and biological properties [24–29]. For example, fluorocortisone, the first fluorine-containing pharmaceutical, possesses remarkable glucocorticoid activity that exceeds the activities of the parent hormones by a factor of 10 [30]. Although fluorine is the 13th most common element in the earth's crust, it mainly exists as inorganic salts. Indeed, the number of biogenic organofluorines is extremely limited (around 20) [31]. Therefore, organofluorine synthesis and application have received tremendous attention in organic chemistry and achieved great advances [32–36]. Currently, a large number of pharmaceuticals and agrochemicals involve at least one fluorine atom. Surprisingly, symmetric 1,3-diketones with fluorine or fluorinated substituents on the prochiral carbon are largely overlooked and remain to be established (Figure 1(a)).

Notably, prochiral 1,3-diketones possess several privileged advantages, including the following: (i) The acidic prochiral carbon can be easily functionalized by deprotonation with various commercially available fluorination reagents (F, CF_3_, CF_2_H, CH_2_F, etc.) [37–42]. Catalytic asymmetric desymmetrization of these substrates ensures a versatile method for the synthesis of enantioenriched organofluorines. Actually, it is challenging to be compatible with fluorine and fluorinated substituents in one asymmetric reaction due to their distinct properties. (ii) Asymmetrically modifying one of the two ketone carbonyl groups leads to the formation of fluorine-containing multiple stereogenic centers. Notably, the synthesis of such great challenging motifs has been largely underdeveloped, although they have already appeared in several invaluable pharmaceuticals (Figure 1(c)) [36].

Given the significant success of asymmetric *N*-heterocyclic carbene (NHC) catalysis [43–52] and privileged structural characters of prochiral 1,3-diketones, based on our ongoing interest in organocatalysis [53–57], we envisioned that a versatile method for the synthesis of enantioenriched organofluorines with multiple stereogenic centers might be established based on NHC-catalyzed asymmetric desymmetrization of novel prochiral fluorinated or fluoromethylated oxindolyl 1,3-ketones. Notably, asymmetric synthesis of spirocycle compounds has attracted a lot of synthetic attention [58–61]. Among them, spiro compounds containing oxindole moieties are proven among the important scaffolds in natural products and bioactive molecules exemplified by those in Figure 1(d) [61, 62]. These easily available prochiral 1,3-diketones could react with unsaturated acyl triazolium intermediates [63–65] obtained from bromoenals with NHC to construct spiropolycyclic organofluorines with five stereogenic centers, including three quaternary stereocenters. While one requirement would be to achieve intermolecular domino desymmetrization, the connection of the reactive site (carbonyl group) to a sterically hindered quaternary carbon center may impede this process. Furthermore, the ability to achieve satisfactory diastereoselectivity and enantioselectivity would remain important. Importantly, organocatalyzed generation of enantioenriched organofluorines prevents heavy metal residues, considerably increasing the potential utilities. We herein have developed a novel prochiral fluorinated oxindanyl 1,3-diketone and successfully applied these substrates in carbene-catalyzed asymmetric desymmetrization. Accordingly, a versatile and practical strategy for asymmetric generation of organofluorines with fluorine or fluorinated methyl groups (CF_3_, CF_2_H, or CH_2_F) has been developed. Multiple stereogenic centers were selectively constructed with satisfactory outcomes. It may be mentioned that enantioselective synthesis of tricyclic *β*-lactones by NHC-catalyzed desymmetrization of cyclic 1,3-diketones has been demonstrated recently by Shee and coworkers [66].

## 2. Results

We initially attempted this synthetic approach with prochiral trifluoromethylated oxindolyl 1,3-diketone 1a and (*Z*)-2-bromo-3-phenylacrylaldehyde 2a under NHC organocatalysis. Trifluoromethylated spiropolycyclic compound 3a was obtained in 11% yield when sterically hindered aminoindanol-derived triazolium precatalyst A was employed in the presence of K_2_CO_3_ in toluene at room temperature (Table 1, entry 1). Subsequently, several bases were screened, and sodium acetate was the best base, giving the product 3a in good yield (82%) with >20 : 1 dr and >99% ee (entries 2-8). Several NHC catalysts were next investigated, with NHC precatalyst B, with a Br atom on the indane moiety, proving to be the better choice to deliver the product 3a in high yield without any loss in dr or ee (entries 9–12). The yield could be further improved to 93% by using mesitylene as the solvent (entries 13-16). 5 mol% catalyst ensured this transformation to give the product in 78% yield (entry 17).

**Figure 2:**
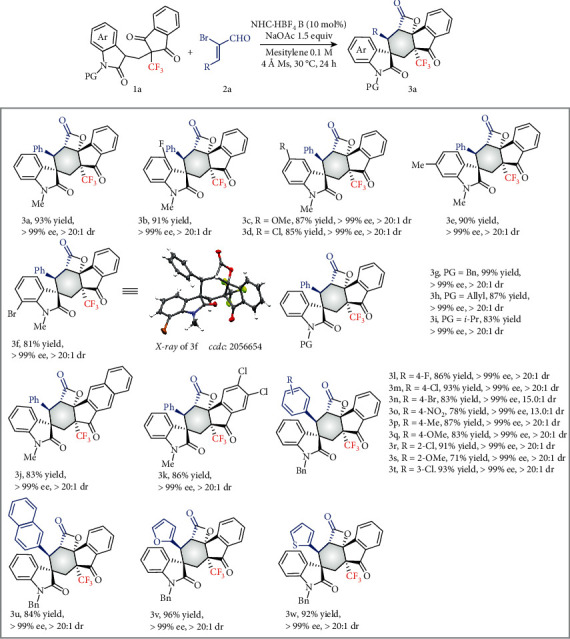
Scope of reactions.

Under optimal conditions (Table 1, entry 15), the scope of this desymmetrization domino reaction was examined (Figure 2). For trifluoromethylated oxindolyl 1,3-diketones, several substituents at the 4-, 5-, 6-, and 7-positons on the oxindole ring were well tolerated to form 3a–3f in good to excellent yields (81-93%) with excellent diastereoselectivities (>20 : 1 dr values) and enantioselectivities (>99% ee values). Substrates with *N*-benzyl, *N*-allyl, and *N*-isopropyl groups reacted efficiently to form products 3g–3i in 83%–93% yields and without any erosion of dr values and ee values. For the indane motif, substrate 1 with a naphthalene unit gave 3j in 83% yield with >20 : 1 dr and >99% ee. Introducing two symmetrical chloride atoms to the indane ring did not influence the efficiency, furnishing the product 3k in 86% yield with >20 : 1 dr and >99% ee. Subsequently, the generality of 2-bromoenals was evaluated. For 2-bromoenals associated with electron-donating or electron-withdrawing groups on the aromatic ring, the reactions worked efficiently to afford the products 3l-3w in good to excellent yields with good to excellent diastereoselectivities and excellent enantioselectivities. Bromoenals with a naphthalene or heteroaryl unit (such as 2-furyl and 2-thienyl) were also compatible with the reaction. Unfortunately, *β*-alkyl-substituted enals failed to deliver the product in our reaction.

**Figure 3:**
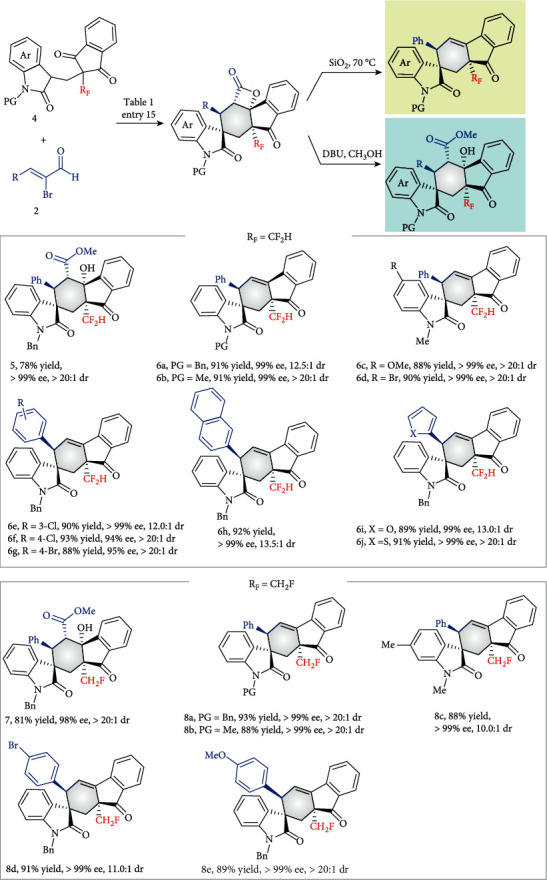
Scope of reactions.

To further investigate the scope and limitations of this organocatalytic strategy, other fluorinated methyl groups, such as difluoromethyl, and monofluoromethyl groups were introduced into the prochiral substrates (Figure 3). Notably, for these substrates, the release of CO_2_ could not be completely avoided under the reaction conditions and in the purification step that followed. Thus, one more decarbonation operation was performed for these substrates. Delightingly, all reactions proceeded smoothly, forming the products 6 and 8 with acceptable results. On the other hand, the intermediate underwent ring opening with a nucleophile, such as methanol, delivering products 5 and 7, respectively. We consider that asymmetric synthesis of important organofluorines containing CF_2_H and CFH_2_ has been overlooked to date [34, 67–69]. Our novel strategy for asymmetric synthesis of these organofluorines by carbene-catalyzed desymmetrization is effective.

**Figure 4:**
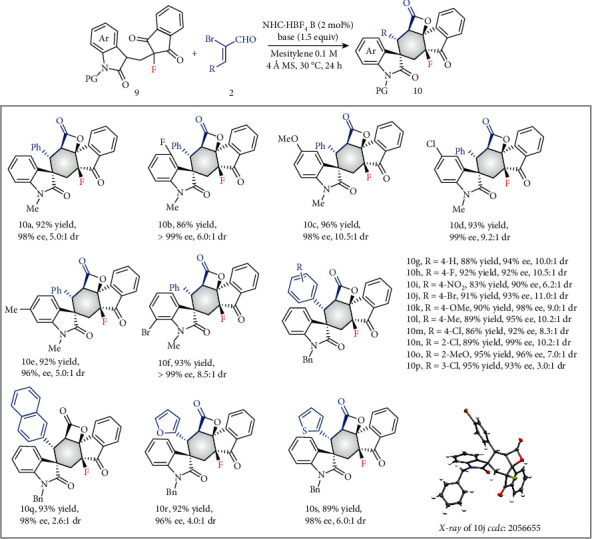
Scope of reactions. Reaction conditions: 9 (0.1 mmol), 2 (1.2 mmol), NHC·HBF_4_ B (2 mol%), NaOAc (1.5 mmol), mesitylene (1 mL), 4 Å MS (50 mg), 30°C, 24 h.

After successfully documenting the synthesis of fluoromethylated molecules with five stereogenic centers under NHC organocatalysis, the generality of this desymmetrization strategy was further explored to construct fluorinated molecules (Figure 4). Different from the above-obtained products 3, 5, 6, 7, and 8, which featured the more sterically hindered fluorinated methyl groups, the resulting product 10 featured the relatively small fluorine atom and showed the opposite absolute configuration under the identical reaction conditions. In all the cases, the desymmetrization cascade process proceeded smoothly by using just 2 mol% carbene catalyst, delivering spirocyclohexene products in 86-93% yields with 2.6 : 1-11 : 1 dr and 90-99% ee values.

**Figure 5:**
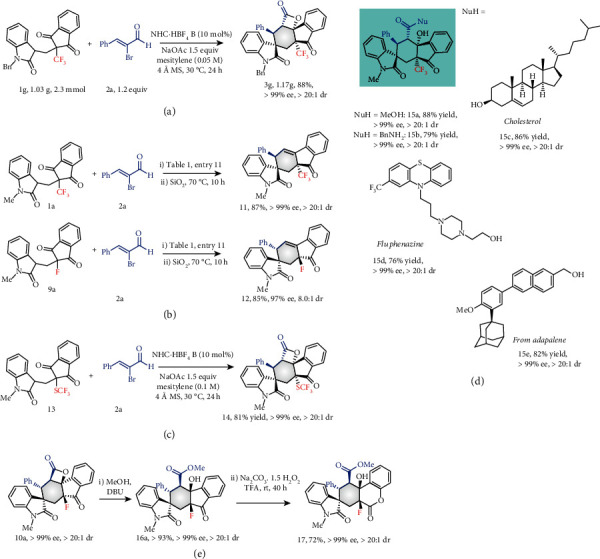
Synthetic application: (a) gram scale synthesis; (b) one pot process to enantioenriched spirocyclohexene product; (c) synthesis of enantioenriched trifluoromethylthiolated spirocyclohexene product; (d) one pot process to cycle-open product; (e) Baeyer-Villiger oxidation of the enantioenriched product.

Under 10 mol% of NHC precatalyst B catalysis, the reaction worked efficiently on a gram scale to generate 3 g in 88% yield with >20 : 1 dr and >99% ee (Figure 5(a)). To demonstrate the practicality of the present strategy, further synthetic transformation of the resulting product was performed as shown in Figure 2. The substrates 1a with CF_3_ and 9a with F underwent intramolecular desymmetrical cyclisation, followed by decarbonation to generate the corresponding products 11 and 12 in good yields with excellent dr and ee values, respectively. Impressively, our method reported here is also effective to prepare enantioenriched trifluoromethylthiolated compounds, leading to product 14 with excellent yields, dr and ee values (Figure 5(c)). Ring opening of the product 3a was achieved by treatment with nucleophiles such as methanol and benzylamine at room temperature and led to the formation of amides 15a and 15b in excellent outcomes. More importantly, for the alcohols with promising biological activities, such as cholesterol, fluphenazine, and adapalene-derived alcohol, the ring-opened products 15c-e were formed in good yields with excellent dr and ee (or de) values. The treatment of 10a with methanol under basic conditions followed by oxygen insertion converted the carbonyl moiety into a carboxylic moiety, leading to product 17 in good yield with the retention of dr and ee.

**Figure 6:**
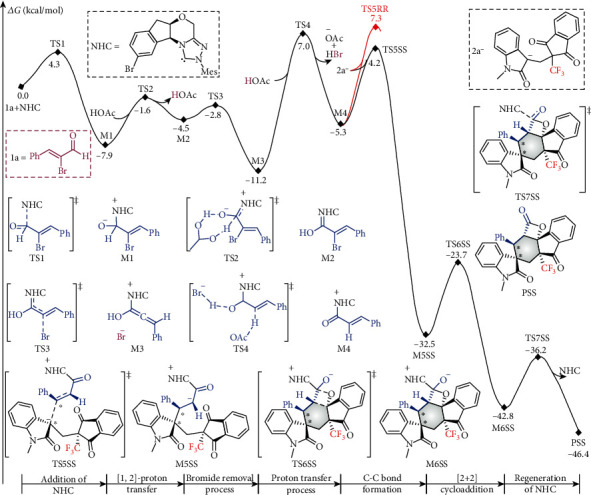
Gibbs free energy profile of the entire catalytic cycle computed at the M06-2X/6-31G(d, p)/IEFPCM mesitylene level.

DFT calculations were conducted to explore the possible pathway. As shown in Figure 6, the carbonyl carbon of reactant 1a is susceptible to nucleophilic attack by the carbene carbon of the actual NHC catalyst via transition state TS1, which is followed by an acetic acid-mediated [1, 2]-proton transfer via transition state TS2 for the formation of Breslow intermediate M2. The third step is a bromide removal process via transition state TS3. Deprotonation can then be mediated by bromide ion, coupled with protonation by acetic acid via transition state TS4. The fifth step involves diastereoselective transition state TS5SS (or TS5RR) for C*β*–C1 bond formation between intermediate M4 and deprotonated achiral 1,3-diketone 2a^−^, which is followed by an intramolecular [2 + 2] cycloaddition to complete the six-membered ring closure via transition state TS6SS. The two letters after the names of the stationary points represent the chirality of the molecules associated with the centrally chiral C1 and C*β* atoms. The last step is the dissociation of catalyst NHC from the main product PSS via transition state TS7SS. The Gibbs free energy barriers of the seven steps via transition states TS1-7 are 4.3, 6.3, 1.7, 18.2, 9.5 (or 12.6 for RR-configured isomer), 8.8, and 5.4 kcal/mol, respectively.

**Figure 7:**
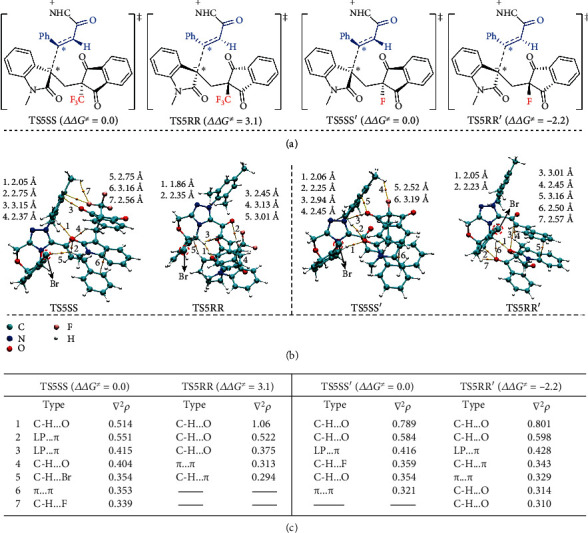
(a) The Gibbs free energy barrier differences (ΔΔ*G*^≠^, energy in kcal/mol), (b) AIM analyses for transition states TS5SS, TS5RR, TS5SS′, and TS5RR′ with different substitutes (distance in Å), and (c) summary of the values of Laplacian electron density (*∇*^2^*ρ* in eÅ^−3^).

To further examine the origin of enantioselectivity, quantitative Bader atoms-in-molecules analyses were carried out. The energy barrier of transition state TS5RR is 3.1 kcal/mol higher than that of transition state TS5SS, whereas the energy barrier of the transition state TS5RR′ is 2.2 kcal/mol lower than that of transition state TS5SS′ (Figure 7(a)), meaning that the enantioselectivity can be switched by exchanging the -CF_3_ substituent with -F. As shown in Figures 3(b) and 3(c), there are no C-H…F hydrogen bond [70] and (long pair) LP…*π* interaction in the TS5RR (RR-configured transition state), which is due to the long distance between the -CF_3_ substituent and NHC catalyst in the RR-configuration. This is why TS5SS is more stable than TS5RR. While the -CF_3_ substituent is replaced by the small -F group, although there is still not C-H…F interaction, the C-H…O hydrogen bond interactions between the substrate and NHC catalyst in TS5RR′ become significantly stronger than those of C-H…O and C-H…F hydrogen bond interactions in transition state TS5SS′, which is the key factor in strengthening the stability of the RR-configured transition state TS5RR′ and leads to the switchable stereoselectivity for the reaction. Noteworthily, 20 possible conformations for TS5SS, TS5RR, TS5SS′, and TS5RR′ were proposed and optimized to ensure that the conformation with the lowest energy was selected. The calculated results were provided in Figure S1 of the SI.

To show the practical value of the resulting enantioenriched fluorinated and fluoromethylated molecules, their antibacterial activities were initially examined by using *Xanthomonas oryzae*, *Xanthomonas axonopodis*, and *Ralstonia solanacearum* as target bacteria [71–73]. These bacteria are widespread plant pathogens that can cause serious plant diseases and huge economic losses in agricultural production [74]. Some of the chiral organofluorines from the current study exhibited superior antibacterial activities, as shown in Table 2. For example, compounds 10a and 8b showed higher inhibitory rates against *Xoo* than the commercial bacteriocide bismerthiazol when used at 50 *μ*g/mL. Similarly, compounds 6b and 16 showed inhibitory rates against *Xal* that were similar to thiodiazole-copper. Under the same conditions, compounds 12 and 15a showed better inhibitory rates against *Rs* than thiodiazole-copper. These promising results indicated that the organofluorines produced in the current study have potential use as agrochemicals.

## 3. Discussion

In summary, asymmetric desymmetrization of a novel prochiral 1,3-diketone with a fluorine or fluorinated methyl group has been demonstrated under NHC organocatalysis. Accordingly, a versatile and practical strategy for the construction of diverse organofluorines featuring fluorine or fluoromethyl groups (CF_3_, CF_2_H, or CH_2_F) has been successfully developed by the current strategy. Notably, products featuring five stereogenic centers, including three quaternary centers and two rings, have been efficiently constructed in this transformation. Mechanism studies and DFT calculations demonstrated that the first C-C bond formation is the stereoselectivity-determining step as well as C-H…O and C-H…F interactions are the key factors to control and even switch the enantioselectivity of the reaction by exchanging the fluoromethyl substituents. The initial test indicated that some of the obtained enantioenriched organofluorines showed competitive antibacterial activities. Further investigations and exploration of this catalytic process and the resulting enantioenriched organofluorines are underway in our laboratory.

## 4. Materials and Methods

### 4.1. General Procedure for the Synthesis of Organofluorine 3

To an oven-dried screw-capped test tube equipped with a magnetic stir bar, the prochiral 1,3-diketones 1 (0.1 mmol), 2-bromoenals 2 (0.12 mmol), triazolium salt NHC B (5.0 mg, 10 mol %), NaOAc (12.3 mg, 1.5 equiv), and 4 Å MS (50 mg) were added. To this mixture was added anhydrous mesitylene (0.1 M). After completion of the reaction, purification of the crude residue gave the desired product 3.

## Figures and Tables

**Table 1:** Optimized conditions^[a]^.

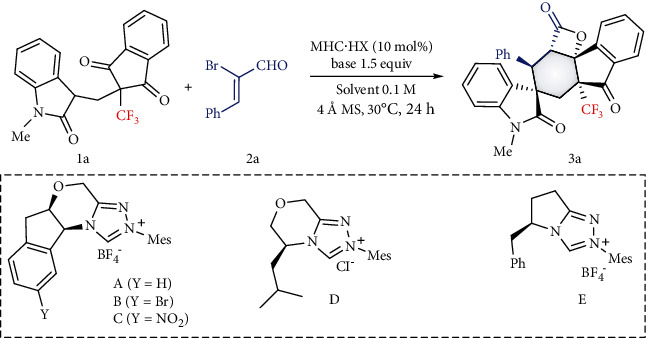						
Entry^[a]^	NHC·HX	Base	Solvent	Yield (%)^[b]^	dr^[c]^	ee^[d]^
1	A	K_2_CO_3_	Toluene	11	—	—
2	A	Cs_2_CO_3_	Toluene	<10	—	—
3	A	Na_2_CO_3_	Toluene	<10	—	—
4	A	NaHCO_3_	Toluene	<10	—	—
5	A	DBU	Toluene	<10	—	—
6	A	KOAc	Toluene	80	>20 : 1	>99
7	A	NaOAc	Toluene	82	>20 : 1	>99
8	A	Et_3_N	Toluene	19	—	—
9	B	NaOAc	Toluene	88	>20 : 1	>99
10	C	NaOAc	Toluene	84	>20 : 1	>99
11	D	NaOAc	Toluene	81	>20 : 1	98
12	E	NaOAc	Toluene	80	>20 : 1	89
13	B	NaOAc	DCM	81	>20 : 1	>99
14	B	NaOAc	THF	<10	—	—
15	B	NaOAc	Mesitylene	93	>20 : 1	>99
16	B	NaOAc	o-Xylene	90	>20 : 1	>99
17^[e]^	B	NaOAc	Mesitylene	78	>20 : 1	>99

^[a]^Standard condition: 1a (0.1 mmol), 2a (1.2 equiv), NHC·HX (10 mol%), solvent (0.1 M), 30°C, 24 h. ^[b]^Yield of the product after column chromatography. ^[c]^Determined via ^1^H NMR spectroscopy. ^[d]^Determined by chiral HPLC, %ee = (R‐S)/(R + S)∗100. ^[e]^5 mol% catalyst, 60 h.

**Table 2:** The antibacterial activity of the products.

Compound	Xanthomonas oryzae pv. oryzae (Xoo) inhibition rate 50 ppm	Xanthomonas axonopodis pv. citri (Xal) inhibition rate 50 ppm	Ralstonia solanacearum (Rs) inhibition rate 50 ppm
3a	0.00% ± 9.86%	44.30% ± 3.66%	0.00% ± 4.53%
6b	74.71% ± 10.22%	73.14% ± 6.77%	78.26% ± 4.53%
8b	99.69% ± 7.57%	64.75% ± 3.60%	38.90% ± 13.88%
10a	93.40% ± 21.20%	41.87% ± 4.98%	58.95% ± 4.70%
11	21.46% ± 1.91%	35.69% ± 11.07%	47.03% ± 6.87%
12	0.00% ± 19.67%	41.28% ± 2.90%	94.57% ± 0.55%
15a	3.90% ± 0.11%	41.65% ± 6.29%	99.69% ± 18.24%
16	0.00% ± 5.79%	70.42% ± 12.69%	0.00% ± 5.57%
Thiodiazole-copper	89.29% ± 10.69%	79.32% ± 0.64%	93.29% ± 1.19%
Bismerthiazol	94.39% ± 22.58%	54.67% ± 1.22%	47.40% ± 7.43%
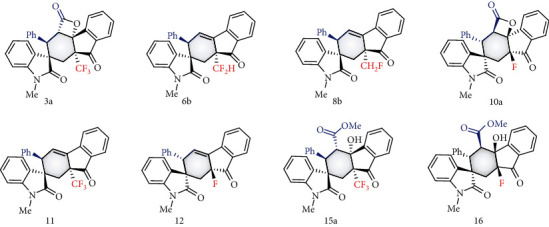			

## Data Availability

The data that support the finding of this study are available from the corresponding authors upon reasonable request.

## Supplementary Materials

Supplementary Materials Preparation of substrates. Characterization of reaction substrates. General procedures. Characterization of reaction products. X-ray crystallographic data of 3f and 10j. Computational details. In vitro antibacterial bioassay. Copies of NMR spectra and HPLC chromatographs. Energies, Cartesian coordinates, and frequencies of all the optimized stationary points in DFT studies.
