# Supplementary material for: Desymmetrization of Cyclic 1,3-Diketones under N-Heterocyclic Carbene Organocatalysis: Access to Organofluorines with Multiple Stereogenic Centers
Source: Research (Wash D C). 2021 Aug 23;2021:9867915. doi: 10.34133/2021/9867915 (PMC8422277; doi:10.34133/2021/9867915)
Supplement: Supplementary Materials — Preparation of substrates. Characterization of reaction substrates. General procedures. Characterization of reaction products. X-ray crystallographic data of 3f and 10j. Computational details. In vitro antibacterial bioassay. Copies of NMR spectra and HPLC chromatographs. Energies, Cartesian coordinates, and frequencies of all the optimized stationary points in DFT studies. [file 9867915.f1.doc]

**Supplementary Information**

**Desymmetrization of Cyclic 1,3-Diketones under *N*-Heterocyclic Carbene Organocatalysis：Access to Organofluorines with Multiple Stereogenic Centers**

Guanjie Wang, Min Zhang, Yezhi Guan, Ye Zhang, Xianfang Hong, Chenlong Wei, Pengcheng Zheng*, Donghui Wei*, Zhenqian Fu*, Yonggui Robin Chi and Wei Huang

| **I** | General information |
| --- | --- |
| **II** | 1. Preparation of Substrates.   b) Full Characterization of Reaction substrates.  c) General Procedure of NHC-catalysed asymmetric desymmetrization of fluorine functionalized 1,3-Diketones.  d) Procedure for the scale-up synthesis of product **3g**.  e) Full Characterization of Reaction Products.  f) X-Ray Crystallographic Data of **3f** and **10j.**  g) Computational Details.  h) Different conformers and configurations of **TS5**s.  i) *In Vitro* Antibacterial Bioassay.  j) Reference. |
| **III** | Copies of NMR Spectra and HPLC Chromatographs |
| **IV** | Energies, Cartesian coordinates, and frequencies of all the optimized stationary points |

**Supplementary Note 1**

**General Information**

Chemicals were purchased as reagent grade and used without further purification. Solvents (THF, toluene) were distilled from appropriate drying agents prior to use. In addition, more solvents were purchased from commercial suppliers and dried over molecular sieves. Thin-layer chromatography (TLC) was performed using silica gel pre-coated glass plates (0.20 mm), which were visualized with a UV lamp (254 or 366 nm). Column chromatography (CC) was carried out using Tsingdao silica gel (60 Å, 200−300 mesh, particle size 0.040−0.063 mm). All reported yields, unless otherwise specified, refer to spectroscopically and chromatographically pure compounds. ^1^H, ^13^C, ^19^F nuclear magnetic resonance (NMR) spectra were recorded on a Bruker spectrometer (400 MHz) in a suitable deuterated solvent. The solvent employed and respective measuring frequency is indicated for each experiment. Chemical shifts are reported with tetramethylsilane (TMS) serving as a universal reference of all nuclides. The resonance multiplicity is described as s (singlet), d (doublet), t (triplet), q (quartet), m (multiplet), and bs (broad singlet). All spectra were recorded at 298 K unless otherwise noted. The residual deuterated solvent signal relative to tetramethylsilane was used as the internal reference in ^1^H NMR spectra (CDCl_3_ δ 7.26), and are reported as follows: chemical shift in ppm (multiplicity, coupling constant J in Hz, number of protons). ^13^C NMR spectra reported in ppm from tetramethylsilane (TMS) with the solvent resonance as the internal standard (CDCl_3_ δ 77.2). High resolution mass spectrometry (HRMS) was performed on a Waters Q-TOF Permier Spectrometer. Optical rotations were determined with Jasco P-1030 polarimeter. Data are reported as follows: [α] _D_^rt^, concentration (c; g/100 mL), and solvents. Enantiomeric excesses (ee) were determined on a Shimadzu LC-20AD HPLC system employing a chiral stationary phase column specified in the individual experiment, by comparing the samples with the appropriate racemic mixtures.

**II**

1. **Preparation of Substrates.**

**Step A**: Aldehyde **S1** (1.05 equiv.) and 1,3-indandione **S2** (10 mmol, 1.0 equiv.) were dissolved in 100 mL ethanol. The reaction mixture was stirred at reflux temperature for about 6 hours, at which time the condensation product precipitates from the reaction mixture. Remove the solvent though decompression filtration , the yellow solid product was obtained in almost equivalent yield which was used directly in the next step. Condensation product (5 mmol, 1.0 equiv) and Hantzsch ester (1.0 equiv) were dissolved in 50 mL CHCl_3_ and the mixture was stirred at reflux temperature for about 12 hours, after the complete conversion of starting material, the reaction mixture was concentrated *in vacuo* to afford a yellow solid. Crystallization of the solid from EtOAc/PE to remove the reduction byproduct, gaving substrates **S3** as pale yellow solids in excellent yield (two steps, >80% yield).

**Step B**: To a solution of substrates **S3** (5.0 mmol, 1.0 equiv) in 1,4-dioxane was added Bu_4_NOH (40% in H_2_O, 1.2 euquiv). After the mixture was stirred at room temperature for 30 min, Selectfluor (1.05 equiv) was slowly added in small portions and the solution was stirred for further 12 h at room temperature. When the reaction is complete, the mixture was poured into aqueous NH_4_Cl solution, and diluted with EtOAc. The organic layer was washed with brine, dried over Na_2_SO_4_, filtered and concentrated. The residue was purified by column chromatography on silica gel to afford the desired fluorination product **S4** as a yellow solid.

Trifluoromethylation product **S6** were prepared following the same produre described above by using regent **S5** as trifluoromethyl cation donor. **S10** were prepared following the same produre described above by using **S9** as trifluoromethylthiolation agent.

Substrates **S7** ^[1]^ and **S8** ^[2]^ were synthesized according to a procedure described in the literature for similar compounds.

**Step C**: Fluoroalkylation (or fluorination) 1, 3-diketones substrates (3.0 mmol, 1.0 equiv) and Bu_4_NCl (3.6 mmol, 1.2 equiv) were dissolved in 20 mL CH_2_Cl_2_, the reaction mixture was cooled to -10 ^o^C. A solution of PIFA (3.6 mmol, 1.2 euquiv) in CH_2_Cl_2_ (5 mL) was added dropwise. The reaction was completed in ten minutes, upon which time the mixture was poured into water, and extracted with CH_2_Cl_2_. The organic layer was washed with brine, dried over Na_2_SO_4_, filtered and concentrated. The residue was purified by column chromatography on silica gel using DCM/PE (4:1-1:0) as eluent to afford the desired product as a white solid in excellent yield.

1. **Full Characterization of Reaction substrates.**

**2-((1-methyl-2-oxoindolin-3-yl)methyl)-2-(trifluoromethyl)-1*H*-indene-1,3(2*H*)-dione （1a）**

**^1^H NMR** **(400 MHz, CDCl_3_)** δ 7.99-8.02 (m, 2H), 7.82-7.89 (m, 2H), 7.17-7.26 (m, 2H), 7.00 (t, *J* = 7.6 Hz, 1H), 6.64 (d, *J* = 8.0 Hz, 1H), 3.51-3.55 (m, 1H), 2.99 (s, 3H), 2.89-2.94 (m, 1H), 2.57-2.64 (m, 1H); **^13^C NMR (100 MHz, CDCl_3_)** δ 193.3, 192.2, 176.0, 144.1, 143.3, 142.0, 136.4, 135.7, 128.8, 127.1, 124.5, 123.9, 123.6, 123.3 (q, 283.7 Hz), 122.8, 108.2, 59.3 (q, 23.4 Hz), 40.5, 27.5, 26.2. **^19^F NMR** **(376 MHz, CDCl_3_)** δ -68.5; **HRMS** (ESI) Calcd for C_20_H_14_F_3_NNaO_3_^+^ [M+Na]^+^ 396.0818; Found: 396.0827.

**2-((4-fluoro-1-methyl-2-oxoindolin-3-yl)methyl)-2-(trifluoromethyl)-1*H*-indene-1,3(2*H*)-dione（1b）**

**^1^H NMR** **(400 MHz, CDCl_3_)** δ 7.97-8.02 (m, 2H), 7.83-7.88 (m, 2H), 7.15-7.21 (m, 1H), 6.70 (t, *J* = 8.8 Hz, 1H), 6.43 (d, *J* = 8.0 Hz, 1H), 3.74 (dd, *J* = 11.2, 4.0 Hz, 1H), 3.14 (dd, *J* = 14.4, 4.0 Hz, 1H), 2.96 (s, 3H), 2.57-2.64 (m, 1H); **^13^C NMR (100 MHz, CDCl_3_)** δ 192.9, 191.9, 175.3, 159.1 (d, *J* = 247.4 Hz), 146.0 (d, *J* = 9.1 Hz), 143.0, 142.2, 136.3, 135.7, 130.8 (d, *J* = 8.7 Hz), 123.8, 123.6, 123.3 (q, 283.8 Hz), 112.9 (d, *J* = 20.2 Hz ), 110.4 (d, *J* = 20.9 Hz ), 104.4, 104.3, 59.2 (q, 23.3 Hz), 39.1, 26.6, 25.9. **^19^F NMR** **(376 MHz, CDCl_3_)** δ -68.5, -117.0 (Ar, 1F); **HRMS** (ESI) Calcd for C_20_H_14_F_4_NO_3_^+^ [M+H]^+^ 392.0904; Found: 392.0907.

**2-((5-methoxy-1-methyl-2-oxoindolin-3-yl)methyl)-2-(trifluoromethyl)-1*H*-indene-1,3(2*H*)-dione（1c）**

**^1^H NMR** **(400 MHz, CDCl_3_)** δ 7.97-8.02 (m, 2H), 7.81-7.88 (m, 2H), 6.84 (s, 1H), 6.68-6.70 (m, 1H), 6.51-6.40 (m, 1H), 3.78 (s, 3H), 3.49-3.52 (m, 1H), 2.96 (s, 3H), 2.89 (dd, *J* = 14.8, 4.0 Hz, 1H), 2.59-2.65 (m, 1H); **^13^C NMR (100 MHz, CDCl_3_)** δ 193.2, 192.3, 175.6, 156.0, 143.3, 142.0, 137.6, 136.4, 135.6, 128.3, 123.8, 123.6, 123.3 (q, 283.8 Hz), 113.5, 111.6, 108.6, 59.3 (q, 23.4 Hz), 55.9, 41.0, 27.6, 26.3. **^19^F NMR** **(376 MHz, CDCl_3_)** δ -68.5; **HRMS** (ESI) Calcd for C_21_H_17_F_3_NO_4_^+^ [M+H]^+^ 404.1104; Found: 404.1108.

**2-((5-chloro-1-methyl-2-oxoindolin-3-yl)methyl)-2-(trifluoromethyl)-1*H*-indene-1,3(2*H*)-dione（1d）**

**^1^H NMR** **(400 MHz, CDCl_3_)** δ 7.83-8.06 (m, 4H), 7.14-7.21 (m, 2H), 6.58 (d, *J* = 8.0 Hz, 1H), 3.53-3.56 (m, 1H), 2.99 (s, 3H), 2.84-2.89 (m, 1H), 2.57-2.63 (m, 1H); **^13^C NMR (100 MHz, CDCl_3_)** δ 193.1, 192.2, 175.5, 143.2, 142.7, 142.0, 136.5, 135.8, 128.8, 128.7, 128.2, 125.0, 124.6, 123.9, 123.7, 123.2 (q, 283.7 Hz), 121.8, 109.2, 59.2 (q, 23.5 Hz), 40.6, 27.3, 26.3. **^19^F NMR** **(376 MHz, CDCl_3_)** δ -68.4; **HRMS** (ESI) Calcd for C_20_H_14_ClF_3_NO_3_^+^ [M+H]^+^ 408.0609; Found: 408.0603.

**2-((1,6-dimethyl-2-oxoindolin-3-yl)methyl)-2-(trifluoromethyl)-1*H*-indene-1,3(2*H*)-dione（1e）**

**^1^H NMR** **(400 MHz, CDCl_3_)** δ 7.95-7.99 (m, 2H), 7.81-7.88 (m, 2H), 7.07 (d, *J* = 7.6 Hz, 1H), 6.77 (d, *J* = 7.6 Hz, 1H), 6.41 (s, 1H), 3.48 (dd, *J* = 10.8, 3.6 Hz, 1H), 2.97 (s, 3H), 2.91 (dd, *J* = 14.4, 4.0 Hz, 1H), 2.59-2.66 (m, 1H), 2.28 (s, 3H); **^13^C NMR (100 MHz, CDCl_3_)** δ 193.2, 192.4, 176.3, 144.3, 143.3, 142.0, 139.0, 136.2, 135.6, 124.4, 124.0, 123.8, 123.5, 123.3 (q, 283.8 Hz), 123.2, 109.1, 59.3 (q, 23.4 Hz), 40.4, 27.9, 26.1, 21.8. **^19^F NMR** **(376 MHz, CDCl_3_)** δ -68.5; **HRMS** (ESI) Calcd for C_21_H_17_F_3_NO_3_^+^ [M+H]^+^ 388.1155; Found: 388.1161.

**2-((7-bromo-1-methyl-2-oxoindolin-3-yl)methyl)-2-(trifluoromethyl)-1*H*-indene-1,3(2*H*)-dione（1f）**

**^1^H NMR** **(400 MHz, CDCl_3_)** δ 7.98-8.03 (m, 2H), 7.85-7.91 (m, 2H), 7.28 (d, *J* = 8.0 Hz, 1H), 7.17 (d, *J* = 7.6 Hz, 1H), 6.85 (t, *J* = 8.0 Hz, 1H), 3.54 (dd *J* = 10.8, 4.0 Hz, 1H), 3.35 (s, 3H), 2.88 (dd *J* = 14.8, 4.4 Hz, 1H), 2.57-2.64 (m, 1H); **^13^C NMR (100 MHz, CDCl_3_)** δ 193.1, 192.1, 176.2, 143.1, 142.0, 141.4, 136.5, 135.9, 134.4, 130.00, 124.6, 123.9, 123.8, 123.7, 123.6, 102.5, 59.2 (q, 23.3 Hz), 40.4, 29.8, 27.7. **^19^F NMR** **(376 MHz, CDCl_3_)** δ -68.5; **HRMS** (ESI) Calcd for C_20_H_14_BrF_3_NO_3_^+^ [M+H]^+^ 452.0104; Found: 452.0113.

**2-((1-benzyl-2-oxoindolin-3-yl)methyl)-2-(trifluoromethyl)-1*H*-indene-1,3(2*H*)-dione （1g）**

**^1^H NMR** **(400 MHz, CDCl_3_)** δ 8.00-8.06 (m, 2H), 7.83-7.90 (m, 2H), 7.22-7.32 (m, 4H), 7.17 (d, *J* = 6.8 Hz, 2H), 7.09 (t, *J* = 7.6 Hz, 1H), 6.99 (t, *J* = 7.2 Hz, 1H), 6.55 (d, *J* = 8.0 Hz, 1H), 4.69 (s, 2H), 3.66 (dd, *J* = 11.6, 4.4 Hz, 1H), 2.95 (dd, *J* = 14.4, 4.0 Hz, 1H), 2.96-2.67 (m, 1H); **^13^C NMR (100 MHz, CDCl_3_)** δ 193.4, 192.1, 176.2, 143.4, 143.1, 142.2, 136.4, 135.7, 135.4, 128.9, 128.7, 127.8, 127.5, 127.3, 124.4, 123.9, 123.8, 123.4 (q, 283.9 Hz), 122.8, 109.4, 59.5 (q, 23.3 Hz), 43.7, 40.4, 27.9. **^19^F NMR** **(376 MHz, CDCl_3_)** δ -68.3; **HRMS** (ESI) Calcd for C_26_H_19_F_3_NO_3_^+^ [M+H]^+^ 450.1312; Found: 450.1314.

**2-((1-allyl-2-oxoindolin-3-yl)methyl)-2-(trifluoromethyl)-1*H*-indene-1,3(2*H*)-dione（1h）**

**^1^H NMR** **(400 MHz, CDCl_3_)** δ 7.99-8.03 (m, 2H), 7.81-7.88 (m, 2H), 7.25 (d, *J* = 7.2 Hz, 1H), 7.15 (t, *J* = 7.6 Hz, 1H), 6.99 (t, *J* = 7.6 Hz, 1H), 6.66 (d, *J* = 8.0 Hz, 1H), 5.64-5.73 (m, 1H), 5.09-5.16 (m, 2H), 4.15-4.21 (m, 1H), 4.02-4.07 (m, 1H), 3.55-3.59 (m, 1H), 2.88-2.93 (m, 1H), 2.56-2.62 (m, 1H); **^13^C NMR (100 MHz, CDCl_3_)** δ 193.3, 192.1, 175.7, 143.4, 143.3, 142.1, 136.4, 135.7, 131.3, 128.7, 127.3, 124.5, 123.9, 123.7, 123.3 (q, 284.2 Hz), 122.7, 117.8, 109.2, 59.3 (q, 23.0 Hz), 42.3, 40.4, 27.8. **^19^F NMR** **(376 MHz, CDCl_3_)** δ -68.4; **HRMS** (ESI) Calcd for C_22_H_17_F_3_NO_3_^+^ [M+H]^+^ 400.1155; Found: 400.1156.

**2-((1-isopropyl-2-oxoindolin-3-yl)methyl)-2-(trifluoromethyl)-1*H*-indene-1,3(2*H*)-dione（1i）**

**^1^H NMR** **(400 MHz, CDCl_3_)** δ 7.99-8.03 (m, 2H), 7.81-7.88 (m, 2H), 7.23-7.26 (m, 1H), 7.14 (t, *J* = 7.6 Hz, 1H), 6.96 (t, *J* = 7.6 Hz, 1H), 6.87 (d, *J* = 7.6 Hz, 1H), 4.39-4.50 (m, 1H), 3.49 (dd, *J* = 11.2, 4.0 Hz, 1H), 2.90 (dd, *J* = 14.4, 4.0 Hz, 1H), 2.55-2.62 (m, 1H), 1.36 (d, *J* = 6.8 Hz, 6H), 1.31 (d, *J* = 6.8 Hz, 6H); **^13^C NMR (100 MHz, CDCl_3_)** δ 193.3, 192.2, 175.6, 143.4, 142.6, 142.1, 136.3, 135.6, 128.4, 127.9, 124.7, 123.9, 123.7, 123.2 (q, 283.9 Hz), 122.1, 110.2, 59.4 (q, *J* = 23.5 Hz), 43.5, 40.4, 27.8, 19.4, 19.2. **^19^F NMR** **(376 MHz, CDCl_3_)** δ -68.4; **HRMS** (ESI) Calcd for C_22_H_19_F_3_NO_3_^+^ [M+H]^+^ 402.1312; Found: 402.1317.

**2-((1-methyl-2-oxoindolin-3-yl)methyl)-2-(trifluoromethyl)-1*H*-cyclopenta[*b*]naphthalene-1,3(2*H*)-dione（1j）**

**^1^H NMR** **(400 MHz, CDCl_3_)** δ 8.56 (d, *J* = 2.8 Hz, 2H), 8.10-8.14 (m, 2H), 7.71-7.77 (m, 2H), 7.29 (d, *J* = 7.6 Hz, 1H), 7.18 (t, *J* = 7.6 Hz, 1H), 7.02 (t, *J* = 7.6 Hz, 1H), 6.63 (d, *J* = 8.0 Hz, 1H), 3.64 (dd *J* = 11.6, 4.4 Hz, 1H), 2.94-3.01 (m, 4H), 2.60-2.67 (m, 1H); **^13^C NMR (100 MHz, CDCl_3_)** δ 193.6, 192.4, 176.2, 144.1, 137.8, 136.9, 136.8, 136.4, 130.8, 130.1, 129.9, 128.7, 127.5, 125.1, 124.3, 122.8, 108.2, 60.6 (q, 22.9 Hz), 40.4, 27.7, 26.2. **^19^F NMR** **(376 MHz, CDCl_3_)** δ -68.2; **HRMS** (ESI) Calcd for C_24_H_17_F_3_NO_3_^+^ [M+H]^+^ 424.1155; Found: 424.1157.

**5,6-dichloro-2-((1-methyl-2-oxoindolin-3-yl)methyl)-2-(trifluoromethyl)-1H-indene-1,3(2H)-dione（1k）**

**^1^H NMR** **(400 MHz, CDCl_3_)** δ 8.08 (d, *J* = 7.6 Hz, 2H), 7.23-7.27 (m, 2H), 7.05 (t, *J* = 7.6 Hz, 1H), 6.71 (d, *J* = 8.0 Hz, 1H), 3.52 (dd *J* = 12.0, 4.4 Hz, 1H), 3.00 (s, 3H), 2.88 (dd *J* = 14.4, 4.4 Hz, 1H), 2.46-2.53 (m, 1H); **^13^C NMR (100 MHz, CDCl_3_)** δ 191.3, 189.8, 176.1, 144.0, 142.1, 141.8, 141.1, 140.7, 128.9, 127.2, 125.6, 125.4, 124.2, 123.1 (q, 284.2 Hz), 123.0, 108.4, 59.9 (q, 23.7 Hz), 40.2, 27.3, 26.2. **^19^F NMR** **(376 MHz, CDCl_3_)** δ -68.4; **HRMS** (ESI) Calcd for C_20_H_13_Cl_2_F_3_NO_3_^+^ [M+H]^+^ 442.0219; Found: 442.0218.

**2-((1-benzyl-2-oxoindolin-3-yl)methyl)-2-(difluoromethyl)-1*H*-indene-1,3(2*H*)-dione （4a）**

**^1^H NMR** **(400 MHz, CDCl_3_)** δ 7.92-7.98 (m, 2H), 7.78-7.84 (m, 2H), 7.16-7.30 (m, 6H), 7.04 (t, *J* = 8.0 Hz, 1H), 6.94 (t, *J* = 7.2 Hz, 1H), 5.95 (t, *J* = 55.2 Hz, 1H), 4.74 (d, *J* = 15.6 Hz, 1H), 4.63 (d, *J* = 15.6 Hz, 1H), 3.61 (dd *J* = 10.8, 4.0 Hz, 1H), 2.76 (dd *J* = 14.8, 4.4 Hz, 1H), 2.56 (dd *J* = 14.4, 10.8 Hz, 1H); **^13^C NMR (100 MHz, CDCl_3_)** δ 196.5, 195.4, 176.3, 143.6, 143.2, 142.3, 136.2, 135.5, 135.5, 128.8, 128.6, 127.7, 127.5, 127.4, 124.6, 123.6, 123.3, 122.7, 116.2 (t, *J* = 250.0 Hz, 1H), 109.3, 59.5 (t, *J* = 18.3 Hz, 1H), 43.7, 40.8, 28.5. **^19^F NMR** **(376 MHz, CDCl_3_)** δ -121.8 (dd, *J* = 283.5, 55.6 Hz), -123.8 (dd, *J* = 283.9, 56.4 Hz); **HRMS** (ESI) Calcd for C_26_H_20_F_2_NO_3_^+^ [M+H]^+^ 432.1406; Found: 432.1392.

**2-(difluoromethyl)-2-((1-methyl-2-oxoindolin-3-yl)methyl)-1*H*-indene-1,3(2*H*)-dione （4b）**

**^1^H NMR** **(400 MHz, CDCl_3_)** δ 7.92-7.94 (m, 2H), 7.78-7.84 (m, 2H), 7.13-7.20 (m, 2H), 6.96 (t, *J* = 7.2 Hz, 1H), 6.58 (d, *J* = 7.6 Hz, 1H), 5.94 (t, *J* = 55.2 Hz, 1H), 3.50 (dd *J* = 10.4, 3.6 Hz, 1H), 2.99 (s, 3H), 2.75 (dd *J* = 14.4, 4.0 Hz, 1H), 2.55 (dd *J* = 14.4, 10.4 Hz, 1H); **^13^C NMR (100 MHz, CDCl_3_)** δ 196.4, 195.6, 176.1, 144.2, 143.5, 142.2, 136.1, 135.4, 128.8, 127.1, 124.7, 123.5, 123.2, 122.6, 116.3 (t, *J* = 250.0 Hz), 108.1, 59.4 (t, *J* = 19.0 Hz), 41.0, 28.3, 28.2, 26.2. **^19^F NMR** **(376 MHz, CDCl_3_)** δ -122.1 (dd, *J* = 284.3, 56.4 Hz), -124.2 (dd, *J* = 284.6, 56.4 Hz); **HRMS** (ESI) Calcd for C_20_H_16_F_2_NO_3_^+^ [M+H]^+^ 356.1093; Found: 356.1097.

**2-(difluoromethyl)-2-((5-methoxy-1-methyl-2-oxoindolin-3-yl)methyl)-1H-indene-1,3(2H)-dione （4c）**

**^1^H NMR** **(400 MHz, CDCl_3_)** δ 7.90-7.95 (m, 2H), 7.77-7.83 (m, 2H), 6.79 (s, 1H), 6.64 (d, *J* = 8.8 Hz, 1H), 6.45-6.48 (m, 1H), 5.93 (t, *J* = 55.2 Hz, 1H), 3.75 (s, 3H), 3.46-3.49 (m, 1H), 2.97 (s, 3H), 2.73 (dd *J* = 14.8, 4.0 Hz, 1H), 2.57 (dd *J* = 14.8, 10.4 Hz, 1H); **^13^C NMR (100 MHz, CDCl_3_)** δ 196.2, 195.6, 175.7, 155.9, 143.5, 142.2, 137.7, 136.1, 135.4, 128.2, 123.4, 123.2, 116.3 (t, *J* = 249.9 Hz), 113.6, 111.7, 108.5, 59.3 (t, *J* = 18.6 Hz), 55.9, 41.4, 28.3, 26.3. **^19^F NMR** **(376 MHz, CDCl_3_)** δ -122.0 (dd, *J* = 284.3, 56.4 Hz), -124.3 (dd, *J* = 284.3, 56.4 Hz); **HRMS** (ESI) Calcd for C_21_H_18_F_2_NO_4_^+^ [M+H]^+^ 386.1198; Found: 386.1193.

**2-((5-bromo-1-methyl-2-oxoindolin-3-yl)methyl)-2-(difluoromethyl)-1*H*-indene-1,3(2*H*)-dione （4d）**

**^1^H NMR** **(400 MHz, CDCl_3_)** δ 8.00 (d, *J* = 7.2 Hz, 1H), 7.92 (d, *J* = 7.6 Hz, 1H), 7.79-7.87 (m, 2H), 7.25-7.29 (m, 2H), 6.49 (d, *J* = 8.4 Hz, 1H), 5.93 (t, *J* = 55.2 Hz, 1H), 3.52 (dd, *J* = 10.4, 3.6 Hz, 1H), 3.00 (s, 3H), 2.70 (d, *J* = 14.4, 4.0 Hz, 1H), 2.55 (dd, *J* = 14.8, 10.8 Hz, 1H); **^13^C NMR (100 MHz, CDCl_3_)** δ 196.2, 195.5, 175.6, 143.4, 143.3, 142.2, 136.3, 135.6, 131.6, 129.2, 127.9, 123.6, 123.3, 116.1 (t, *J* = 249.8 Hz), 113.6, 109.5, 59.3 (t, *J* = 18.2 Hz), 40.9, 28.0, 26.3. **^19^F NMR** **(376 MHz, CDCl_3_)** δ -121.7 (dd, *J* = 283.9, 56.0 Hz), -124.0 (dd, *J* = 284.3, 56.4 Hz); **HRMS** (ESI) Calcd for C_20_H_15_BrF_2_NO_3_^+^ [M+H]^+^ 434.0198; Found: 434.0194.

**2-((1-benzyl-2-oxoindolin-3-yl)methyl)-2-(fluoromethyl)-1*H*-indene-1,3(2*H*)-dione （4e）**

**^1^H NMR** **(400 MHz, CDCl_3_)** δ 7.88-7.93 (m, 2H), 7.75-7.80 (m, 2H), 7.18-7.30 (m, 5H), 7.12 (d, *J* = 7.2 Hz, 1H), 7.00 (t, *J* = 7.6 Hz, 1H), 6.90 (t, *J* = 7.6 Hz, 1H), 6.47 (d, *J* = 8.0 Hz, 1H), 4.58-4.82 (m, 4H), 3.60 (t, *J* = 6.8 Hz, 1H), 2.36 (d, *J* = 6.8 Hz, 2H); **^13^C NMR (100 MHz, CDCl_3_)** δ 200.3, 199.5, 176.4, 143.3, 143.2, 142.1, 135.9, 135.6, 135.3, 128.8, 128.5, 127.7, 127.6, 127.5, 124.6, 123.3, 123.1, 122.6, 109.1, 85.5 (d, *J* = 177.8 Hz), 57.4 (d, *J* = 18.6 Hz), 43.8, 41.2, 29.2, 29.1. **^19^F NMR** **(376 MHz, CDCl_3_)** δ -220.5 (t, *J* = 47.4 Hz); **HRMS** (ESI) Calcd for C_26_H_20_FNNaO_3_^+^ [M+Na]^+^ 436.1319; Found: 436.1325.

**2-(fluoromethyl)-2-((1-methyl-2-oxoindolin-3-yl)methyl)-1*H*-indene-1,3(2*H*)-dione （4f）**

**^1^H NMR** **(400 MHz, CDCl_3_)** δ 7.86-7.91 (m, 2H), 7.75-7.79 (m, 2H), 7.09-7.12 (m, 2H), 6.92 (t, *J* = 7.6 Hz, 1H), 6.52 (t, *J* = 7.6 Hz, 1H), 4.56-4.77 (m, 2H), 3.47 (t, *J* = 6.4 Hz, 1H), 2.99 (s, 3H), 2.34 (d, *J* = 6.4 Hz, 2H); **^13^C NMR (100 MHz, CDCl_3_)** δ 200.1, 199.6, 176.2, 144.2, 143.1, 142.1, 135.9, 135.3, 128.7, 127.2, 124.8, 123.3, 122.9, 122.5, 108.0, 85.4 (d, *J* = 177.0 Hz), 57.3 (d, *J* = 18.8 Hz), 41.3, 28.8, 28.7, 26.2. **^19^F NMR** **(376 MHz, CDCl_3_)** δ -220.6 (t, *J* = 47.4 Hz); **HRMS** (ESI) Calcd for C_20_H_17_FNO_3_^+^ [M+H]^+^ 338.1187; Found: 338.1189.

**2-((1,6-dimethyl-2-oxoindolin-3-yl)methyl)-2-(fluoromethyl)-1*H*-indene-1,3(2*H*)-dione （4g）^1^H NMR** **(400 MHz, CDCl_3_)** δ 7.71-7.85 (m, 4H), 6.92 (d, *J* = 7.6 Hz, 1H), 6.67 (d, *J* = 7.6 Hz, 1H), 6.25 (s, 1H), 4.54-4.75 (m, 2H), 3.41 (t, *J* = 6.0 Hz, 1H), 2.95 (s, 3H), 2.34-2.39 (m, 2H), 2.20 (s, 3H); **^13^C NMR (100 MHz, CDCl_3_)** δ 199.9, 199.8, 176.5, 144.4, 143.0, 142.1, 138.9, 135.4, 135.1, 124.8, 123.9, 123.1, 122.9, 122.7, 108.8, 85.3 (d, *J* = 176.5 Hz), 57.2 (d, *J* = 18.7 Hz), 41.2, 29.2, 29.1, 26.1, 21.7. **^19^F NMR** **(376 MHz, CDCl_3_)** δ -220.7 (t, *J* = 47.4 Hz); **HRMS** (ESI) Calcd for C_21_H_19_FNO_3_^+^ [M+H]^+^ 352.1343; Found: 352.1341.

**2-fluoro-2-((1-methyl-2-oxoindolin-3-yl)methyl)-1*H*-indene-1,3(2*H*)-dione (9a)**

**^1^H NMR (400 MHz, CDCl_3_)** δ 8.04-8.06 (m, 1H), 7.89-7.98 (m, 3H), 7.45 (d, *J* = 7.6 Hz, 1H), 7.25 (t, *J* = 7.6 Hz, 1H), 7.07 (t, *J* = 7.6 Hz, 1H), 6.71 (d, *J* = 8.0 Hz, 1H), 3.86 (t, *J* = 6.4 Hz, 1H), 3.07 (s, 3H), 2.66 (t, *J* = 6.4 Hz, 1H), 2.60 (t, *J* = 6.0 Hz, 1H); **^13^C NMR (100 MHz, CDCl_3_)** δ 193.6 (dd, *J* = 46.4, 17.5 Hz), 176.4, 144.1, 140.5, 140.3, 137.2, 137.0, 128.6, 127.5, 125.5, 125.4, 124.5, 122.9, 108.1, 90.5 (d, *J* = 194.0 Hz), 40.8 (d, *J* = 4.0 Hz), 32.5 (d, *J* = 26.0 Hz), 26.4. **^19^F NMR (376 MHz, CDCl_3_)** δ -163.3 (t, *J* = 23.7 Hz). **HRMS** (ESI) Calcd for C_19_H_15_FNO_3_^+^ [M+H]^+^ 324.1030; Found: 324.1033.

**2-fluoro-2-((4-fluoro-1-methyl-2-oxoindolin-3-yl)methyl)-1*H*-indene-1,3(2*H*)-dione (9b)**

**^1^H NMR (400 MHz, CDCl_3_)** δ 8.01-8.03 (m, 1H), 7.85-7.93 (m, 3H), 7.19-7.26 (m, 1H), 6.74 (t, *J* = 8.8 Hz, 1H), 6.47 (d, *J* = 8.0 Hz, 1H), 3.83-3.86 (m, 1H), 2.92-3.06 (m, 4H), 2.73-2.84 (m, 1H); **^13^C NMR (100 MHz, CDCl_3_)** δ 193.0 (dd, *J* = 71.0, 17.0 Hz), 175.4, 159.2 (d, *J* = 247.6 Hz), 146.0 (d, *J* = 9.0 Hz), 141.0, 140.4, 137.0, 136.6, 130.6 (d, *J* = 9.0 Hz), 124.3 (d, *J* = 13.0 Hz), 112.8 (d, *J* = 17.0 Hz), 110.4 (d, *J* = 21.0 Hz), 104.4, 104.4, 90.3 (d, *J* = 190.0 Hz), 39.3 (d, *J* = 8.0 Hz), 29.9 (d, *J* = 28.0 Hz), 26.7. **^19^F NMR (376 MHz, CDCl_3_)** δ -117.0 (Ar, 1F), -159.2 (t, *J* = 21.1 Hz). **HRMS** (ESI) Calcd for C_19_H_14_F_2_NO_3_^+^ [M+H]^+^ 342.0936; Found: 342.0941.

**2-fluoro-2-((5-methoxy-1-methyl-2-oxoindolin-3-yl)methyl)-1*H*-indene-1,3(2*H*)-dione （9c）**

**^1^H NMR** **(400 MHz, CDCl_3_)** δ 7.92-8.05 (m, 4H), 7.08 (s, 1H), 6.77 (d, *J* = 8.0 Hz, 1H), 6.61 (d, *J* = 8.4 Hz, 1H), 3.80-3.85 (m, 4H), 3.04 (s, 3H), 2.58-2.65 (m, 2H); **^13^C NMR (100 MHz, CDCl_3_)** δ 193.5 (dd, *J* = 48.0, 17.0 Hz), 176.0, 156.2, 140.5, 140.2, 137.6, 137.2, 137.0, 128.7, 124.4, 113.3, 112.5, 112.5, 108.5, 90.6 (d, *J* = 194.0 Hz), 56.0, 41.3 (d, *J* = 5.0 Hz), 32.5 (d, *J* = 26.0 Hz), 26.5. **^19^F NMR** **(376 MHz, CDCl_3_)** δ -163.1 (t, *J* = 23.3 Hz); **HRMS** (ESI) Calcd for C_20_H_17_FNO_4_^+^ [M+H]^+^ 354.1136; Found: 354.1137.

**2-((5-chloro-1-methyl-2-oxoindolin-3-yl)methyl)-2-fluoro-1*H*-indene-1,3(2*H*)-dione （9d）**

**^1^H NMR** **(400 MHz, CDCl_3_)** δ 8.01-8.07 (m, 2H), 7.94-7.97 (m, 2H), 7.48 (s, 1H), 7.23-7.25 (m, 1H), 6.68 (d, *J* = 8.4 Hz, 1H), 3.93 (t, *J* = 6.4 Hz, 1H), 3.10 (s, 3H), 2.52-2.65 (m, 2H); **^13^C NMR (100 MHz, CDCl_3_)** δ 193.5 (dd, *J* = 50.0, 18.0 Hz), 176.1, 142.7, 140.3, 140.2, 137.4, 137.2, 129.4, 128.5, 128.4, 126.2, 126.1, 124.6 (d, *J* = 7.0 Hz), 109.0, 90.3 (d, *J* = 196.0 Hz), 40.9 (d, *J* = 4.0 Hz), 32.5 (d, *J* = 26.0 Hz), 26.6. **^19^F NMR** **(376 MHz, CDCl_3_)** δ -163.8 (t, *J* = 25.4 Hz); **HRMS** (ESI) Calcd for C_19_H_13_ClFNNaO_3_^+^ [M+Na]^+^ 380.0460; Found: 380.0460.

**2-((1,6-dimethyl-2-oxoindolin-3-yl)methyl)-2-fluoro-1*H*-indene-1,3(2*H*)-dione （9e）**

**^1^H NMR** **(400 MHz, CDCl_3_)** δ 8.03-8.06 (m, 1H), 7.89-7.97 (m, 3H), 7.31 (d, *J* = 7.6 Hz, 1H), 6.88 (d, *J* = 7.6 Hz, 1H), 6.51 (s, 1H), 3.81 (d, *J* = 6.4 Hz, 1H), 3.05 (s, 3H), 2.65 (d, *J* = 6.4 Hz, 1H), 2.59-2.61 (m, 1H), 2.35 (s, 3H); **^13^C NMR (100 MHz, CDCl_3_)** δ 193.6 (dd, *J* = 42.0, 18.0 Hz), 176.7, 144.2, 140.5, 140.3, 138.7, 137.1, 136.9, 125.2, 125.2, 124.5, 124.4, 123.4, 109.0, 90.6 (d, *J* = 194.0 Hz), 40.6 (d, *J* = 5.0 Hz), 32.6 (d, *J* = 26.0 Hz), 26.3, 21.9. **^19^F NMR** **(376 MHz, CDCl_3_)** δ -163.1 (t, *J* = 22.9 Hz); **HRMS** (ESI) Calcd for C_20_H_17_FNO_3_^+^ [M+H]^+^ 338.1187; Found: 338.1191.

**2-((7-bromo-1-methyl-2-oxoindolin-3-yl)methyl)-2-fluoro-1*H*-indene-1,3(2*H*)-dione （9f）**

**^1^H NMR** **(400 MHz, CDCl_3_)** δ 8.06-8.08 (m, 1H), 7.91-7.98 (m, 3H), 7.36 (t, *J* = 8.0 Hz, 2H), 6.92 (t, *J* = 7.6 Hz, 1H), 3.88 (t, *J* = 6.0 Hz, 1H), 3.44 (s, 3H), 2.56-2.72 (m, 2H); **^13^C NMR (100 MHz, CDCl_3_)** δ 193.4 (dd, *J* = 40.0, 18.0 Hz), 176.7, 141.3, 140.4, 140.2, 137.3, 137.1, 134.2, 130.4, 124.6, 124.5, 124.4, 124.0, 102.4, 90.3 (d, *J* = 195.0 Hz), 40.6 (d, *J* = 5.0 Hz), 32.4 (d, *J* = 27.0 Hz), 30.1. **^19^F NMR** **(376 MHz, CDCl_3_)** δ -162.8 (t, *J* = 23.7 Hz); **HRMS** (ESI) Calcd for C_19_H_14_BrFNO_3_^+^ [M+H]^+^ 402.0136; Found: 402.0126.

**2-((1-benzyl-2-oxoindolin-3-yl)methyl)-2-fluoro-1H-indene-1,3(2H)-dione （9g）**

**^1^H NMR** **(400 MHz, CDCl_3_)** δ 7.92-8.10 (m, 4H), 7.51 (d, *J* = 8.8 Hz, 1H), 7.21-7.32 (m, 5H), 7.16 (t, *J* = 8.0 Hz, 1H), 7.06 (t, *J* = 7.6 Hz, 1H), 6.65 (d, *J* = 8.0 Hz, 1H), 4.86 (d, *J* = 15.6 Hz, 1H), 4.74 (d, *J* = 15.6 Hz, 1H), 4.03 (t, *J* = 6.8 Hz, 1H), 2.70 (d, *J* = 6.4 Hz, 1H), 2.63-2.65 (m, 1H); **^13^C NMR (100 MHz, CDCl_3_)** δ 193.6 (dd, *J* = 57.0, 17.0 Hz), 176.7, 143.2, 140.5, 140.3, 137.3, 137.0, 135.6, 128.9, 128.4, 127.8, 127.8, 127.3, 125.6, 125.5, 124.6, 124.6, 123.0, 109.2, 90.5 (d, *J* = 195.0 Hz), 44.0, 40.9 (d, *J* = 4.0 Hz), 32.8 (d, *J* = 26.0 Hz). **^19^F NMR** **(376 MHz, CDCl_3_)** δ -163.6 (t, *J* = 24.1 Hz); **HRMS** (ESI) Calcd for C_25_H_19_FNO_3_^+^ [M+H]^+^ 400.1343; Found: 400.1347.

1. **General Procedure of asymmetric synthesis of organofluorines with multiple stereogenic centers under carbene organocatalysis**

**General Procedure A**. To an oven-dried screw-capped test tube equipped with a magnetic stir bar, the prochiral 1, 3-diketones **1** (0.1 mmol, 1.0 equiv), 2-bromoenals **2** (0.12 mmol, 1.2 equiv), triazolium salt NHC **B** (5.0 mg, 10 mol %), NaOAc (12.3 mg, 1.5 equiv) and 4Å MS (50 mg) were added. To this mixture was added anhydrous Mesitylene (0.1 M). The resultant reaction mixture was kept stirring at rt for 24 h. When the reaction is complete, the crude residue was purified by flash column chromatography on silica gel using DCM/PE/EtOAc (1:10:1) as eluent to afford the desired product **3** as a white solid.

**General Procedure B**. To an oven-dried screw-capped test tube equipped with a magnetic stir bar, the prochiral 1, 3-diketones **9** (0.1 mmol, 1.0 equiv), 2-bromoenals **2** (0.12 mmol, 1.2 equiv), triazolium salt NHC **B** (1.0 mg, 2 mol %), NaOAc (12.3 mg, 1.5 equiv) and 4Å MS (50 mg) were added. To this mixture was added anhydrous Mesitylene (0.1 M). The resultant reaction mixture was kept stirring at rt for 24 h. When the reaction is complete, the crude residue was purified by flash column chromatography on silica gel using DCM/PE/EtOAc (1:10:1) as eluent to afford the desired product **10** as a white solid.

**General Procedure C**. To an oven-dried screw-capped test tube equipped with a magnetic stir bar, the prochiral 1, 3-diketones **4** (0.1 mmol, 1.0 equiv), 2-bromoenals **2** (0.12 mmol, 1.2 equiv), triazolium salt NHC **B** (5.0 mg, 10 mol %), NaOAc (12.3 mg, 1.5 equiv) and 4Å MS (50 mg) were added. To this mixture was added anhydrous Mesitylene (0.1 M). The resultant reaction mixture was kept stirring at rt for 24 h. When the reaction is complete, 100 mg SiO_2_ was added and the reaction mixture was stirred at 70 ^o^C for further 8 h. After decarbonation was completed, the crude residue was purified by flash column chromatography on silica gel using DCM/PE/EtOAc (1:10:1) as eluent to afford the desired product as a white solid.

**11** and **12** was prepared following the similar procedure described above.

**General Procedure D**. To an oven-dried screw-capped test tube equipped with a magnetic stir bar, the prochiral 1, 3-diketones **4** (0.1 mmol, 1.0 equiv), 2-bromoenals **2** (0.12 mmol, 1.2 equiv), triazolium salt NHC **B** (5.0 mg, 10 mol %), NaOAc (12.3 mg, 1.5 equiv) and 4Å MS (50 mg) were added. To this mixture was added anhydrous Mesitylene (0.1 M). The resultant reaction mixture was kept stirring at rt for 24 h. When the reaction is complete, MeOH (1.0 mmol, 10 equiv) and DBU (0.15 mmol, 1.5 equiv) was added in sequence and the reaction mixture was stirred at rt for further 12 h. After cycle-open process was completed, the crude residue was purified by flash column chromatography on silica gel using DCM/PE/EtOAc (1:6:1) as eluent to afford the desired product as a white solid.

**13** and **14** was prepared following the similar procedure described above.

**General Procedure E**. To an oven-dried screw-capped test tube equipped with a magnetic stir bar, the prochiral 1, 3-diketones**13** (0.1 mmol, 1.0 equiv), 2-bromoenals **2** (0.12 mmol, 1.2 equiv), triazolium salt NHC **B** (5.0 mg, 10 mol %), NaOAc (12.3 mg, 1.5 equiv) and 4Å MS (50 mg) were added. To this mixture was added anhydrous Mesitylene (0.1 M). The resultant reaction mixture was kept stirring at rt for 24 h. When the reaction is complete, the crude residue was purified by flash column chromatography on silica gel using DCM/PE/EtOAc (1:10:1) as eluent to afford the desired product **14** as a white solid.

**General Procedure F**. **17** was prepared following the similar procedure described in literature. ^[3]^ To a solution of the enantioenriched substrate **16** (>99% ee, >20:1 dr, 49.0 mg, 0.1 mmol) and TFA maintained at 0 ^o^C, Na_2_CO_3_.1.5H_2_O_2_ (0.5 mmol) is slowly added. The mixture is then brought to rt and stirred for 40 h. After full consumption of the starting material, the mixture was quenched with ice water and extracted with CH_2_Cl_2_. The organic layer was washed with NaHCO_3_ (aq), dried over Na_2_SO_4_, filtered and concentrated. The residue was purified by column chromatography on silica gel using DCM/PE/EtOAc (1:6:1) as eluent to afford the desired Baeyer-Villiger oxidation product as a white solid (36.0 mg, 72% yield).

1. **Procedure for the scale-up synthesis of product 3g**

To an oven-dried 50 ml round-bottomed flask equipped with a magnetic stir bar, the prochiral 1, 3-diketones **1g** (1.03 g, 2.3 mmol), 2-bromoenals **2a** (582 g, 1.2 equiv), triazolium salt NHC **B** (114 mg, 10 mol %), NaOAc (283 mg, 1.5 equiv) and 4Å MS (500 mg) were added. To this mixture was added anhydrous Mesitylene (0.05 M). The resultant reaction mixture was kept stirring at rt for 24 h. When the reaction is complete, the crude residue was purified by flash column chromatography on silica gel using DCM/PE/EtOAc (1:10:1) as eluent to afford the desired product **3g** as a white solid (1.17g, 88% yield, >99% ee, >20:1 dr).

1. **Full Characterization of Reaction Products**

**(2a*S*,3*R*,4*R*,5a*R*,10b*R*)-1'-methyl-3-phenyl-5a-(trifluoromethyl)-2a,3,5,5a-tetrahydro-2*H*,6*H*-spiro[fluoreno[4a,4-*b*]oxete-4,3'-indoline]-2,2',6-trione (3a):** 93% yield, >99% ee, >20:1 dr. [α]_D_^23^ (c 1.0, CHCl_3_) = + 83.0.

**HPLC condition:** Chiralpak IB (Hex/*i*PrOH = 95/5, 1.0 mL/min, t_R_ (major) = 15.9min).

**^1^H NMR (400 MHz, CDCl_3_)** δ 7.96 (d, *J* = 8.0 Hz, 1H), 7.84-7.89 (m, 2H), 7.69-7.73 (m, 1H), 7.30 (d, *J* = 7.6 Hz, 1H), 7.13 (t, *J* = 7.6 Hz, 1H), 6.97-7.08 (m, 4H), 6.82-6.84 (m, 2H), 6.51 (d, *J* = 7.6 Hz, 1H), 4.41 (d, *J* = 10.4 Hz, 1H), 3.61 (d, *J* = 10.4 Hz, 1H), 2.87-2.91 (m, 4H), 2.70 (d, *J* = 14.8 Hz, 1H); **^13^C NMR (100 MHz, CDCl_3_)** δ 192.4, 176.2, 169.6, 150.5, 142.8, 136.6, 136.3, 136.1, 131.3, 129.3, 129.1, 128.4, 128.1, 123.7, 123.1, 123.1, 122.7, 108.6, 79.2, 62.0, 57.3 (q, 23.8 Hz), 49.6, 48.8, 33.4, 26.1; **^19^F NMR (376 MHz, CDCl_3_)** δ -67.7. **HRMS** (ESI) Calcd for C_29_H_20_F_3_NNaO_4_^+^ [M+Na]^+^ 526.1237; Found: 526.1237.

**(2a*S*,3*R*,4*R*,5a*R*,10b*R*)-4'-fluoro-1'-methyl-3-phenyl-5a-(trifluoromethyl)-2a,3,5,5a-tetrahydro-2*H*,6*H*-spiro[fluoreno[4a,4-*b*]oxete-4,3'-indoline]-2,2',6-trione (3b):** 91% yield, >99% ee, >20:1 dr. [α]_D_^23^ (c 1.0, CHCl_3_) = + 176.7.

**HPLC condition:** Chiralpak IB (Hex/*i*PrOH = 85/15, 0.8 mL/min, t_R_ (major) = 9.0 min, t_R_ (minor) = 7.6 min).

**^1^H NMR (400 MHz, CDCl_3_)** δ 7.95 (d, *J* = 8.8 Hz, 1H), 7.84-7.89 (m, 2H), 7.68-7.74 (m, 1H), 7.02-7.14 (m, 4H), 6.93-6.95 (m, 2H), 6.72 (t, *J* = 9.2 Hz, 1H), 6.32 (d, *J* = 7.6 Hz, 1H), 4.41 (d, *J* = 10.8 Hz, 1H), 3.95 (d, *J* = 10.8 Hz, 1H), 3.08 (d, *J* = 14.8 Hz, 1H), 2.89-2.93 (m, 4H). **^13^C NMR (100 MHz, CDCl_3_)** δ 192.5, 175.7, 169.4, 158.8 (d, *J* = 250.0 Hz), 150.6, 144.5, 144.4, 136.5, 136.4, 136.3, 131.3, 131.2, 131.1, 128.6, 128.4, 127.5, 123.7, 123.1, 114.9, 114.7, 110.9, 110.7, 104.8, 104.7, 79.2, 61.6, 57.1 (q, *J* = 23.6 Hz), 50.5, 50.5, 46.5, 31.3, 26.6. **^19^F NMR (376 MHz, CDCl_3_)** δ -67.7, -119.3 (Ar, 1F). **HRMS** (ESI) Calcd for C_29_H_20_F_4_NO_4_^+^ [M+H]^+^ 522.1323; Found: 522.1326.

**(2a*S*,3*R*,4*R*,5a*R*,10b*R*)-5'-methoxy-1'-methyl-3-phenyl-5a-(trifluoromethyl)-2a,3,5,5a-tetrahydro-2*H*,6*H*-spiro[fluoreno[4a,4-*b*]oxete-4,3'-indoline]-2,2',6-trione (3c):** 87% yield, >99% ee, >20:1 dr. [α]_D_^23^ (c 1.0, CHCl_3_) = + 56.8.

**HPLC condition:** Chiralpak IB (Hex/*i*PrOH = 95/5, 1.0 mL/min, t_R_ (major) = 21.2 min, t_R_ (minor) = 17.5 min).

**^1^H NMR (400 MHz, CDCl_3_)** δ 7.95 (d, *J* = 8.0 Hz, 1H), 7.83-7.87 (m, 2H), 7.68-7.73 (m, 1H), 6.98-7.06 (m, 3H), 6.84-6.91 (m, 3H), 6.62-6.65 (m, 1H), 6.41 (d, *J* = 8.8 Hz, 1H), 4.40 (d, *J* = 10.4 Hz, 1H), 3.78 (s, 3H), 3.58 (d, *J* = 10.4 Hz, 1H), 2.86-2.90 (m, 4H), 3.16 (d, *J* = 14.8 Hz, 1H). **^13^C NMR (100 MHz, CDCl_3_)** δ 192.4, 175.8, 169.7, 156.4, 150.4, 136.6, 136.3, 136.2, 136.2, 131.3, 130.6, 128.4, 128.1, 123.6, 123.1, 113.1, 110.3, 109.0, 79.2, 77.4, 62.1, 57.3 (q, *J* = 23.6 Hz), 56.0, 50.0, 48.7, 33.6, 26.2. **^19^F NMR (376 MHz, CDCl_3_)** δ -67.7. **HRMS** (ESI) Calcd for C_30_H_23_F_3_NO_4_^+^ [M+H]^+^ 534.5107; Found: 534.5113.

**(2a*S*,3*R*,4*R*,5a*R*,10b*R*)-5'-chloro-1'-methyl-3-phenyl-5a-(trifluoromethyl)-2a,3,5,5a-tetrahydro-2*H*,6*H*-spiro[fluoreno[4a,4-*b*]oxete-4,3'-indoline]-2,2',6-trione (3d):** 85% yield, >99% ee, >20:1 dr. [α]_D_^23^ (c 1.0, CHCl_3_) = + 97.2.

**HPLC condition:** Chiralpak AD (Hex/*i*PrOH = 85/15, 1.0 mL/min, t_R_ (major) = 38.4 min, t_R_ (minor) = 15.2 min).

**^1^H NMR (400 MHz, CDCl_3_)** δ 7.95 (d, *J* = 7.6 Hz, 1H), 7.84-7.89 (m, 2H), 7.69-7.73 (m, 1H), 7.31-7.32 (m, 1H), 7.01-7.11 (m, 4H), 6.83-6.85 (m, 2H), 6.43 (d, *J* = 8.0 Hz, 1H), 4.39 (d, *J* = 10.4 Hz, 1H), 3.60 (d, *J* = 10.4 Hz, 1H), 2.87-2.91 (m, 4H), 2.67 (d, *J* = 15.2 Hz, 1H). **^13^C NMR (100 MHz, CDCl_3_)** δ 192.3, 175.8, 169.3, 150.4, 141.3, 136.5, 136.4, 135.8, 131.4, 131.1, 129.1, 128.6, 128.3, 128.0, 123.7, 123.3, 123.2, 109.5, 79.1, 61.9, 57.2 (q, *J* = 23.5 Hz), 49.9, 48.7, 33.3, 26.2, 19.3. **^19^F NMR (376 MHz, CDCl_3_)** δ -67.7. **HRMS** (ESI) Calcd for C_29_H_20_ClF_3_NO_4_^+^ [M+H]^+^ 538.1027; Found: 538.1034.

**(2a*S*,3*R*,4*R*,5a*R*,10b*R*)-1',6'-dimethyl-3-phenyl-5a-(trifluoromethyl)-2a,3,5,5a-tetrahydro-2*H*,6*H*-spiro[fluoreno[4a,4-*b*]oxete-4,3'-indoline]-2,2',6-trione (3e):** 90% yield, >99% ee, >20:1 dr. [α]_D_^23^ (c 1.0, CHCl_3_) = + 114.8.

**HPLC condition:** Chiralpak IB (Hex/*i*PrOH = 95/5, 1.0 mL/min, t_R_ (major) = 13.9 min, t_R_ (minor) = 11.7 min).

**^1^H NMR (400 MHz, CDCl_3_)** δ 7.95 (d, *J* = 7.6 Hz, 1H), 7.83-7.88 (m, 2H), 7.68-7.72 (m, 1H), 7.17 (d, *J* = 7.6 Hz, 1H), 6.98-7.05 (m, 3H), 6.85 (d, *J* = 7.2 Hz, 3H), 6.33 (s, 1H), 4.41 (d, *J* = 10.4 Hz, 1H), 3.61 (d, *J* = 10.4 Hz, 1H), 2.84-2.87 (m, 4H), 2.68 (d, *J* = 14.8 Hz, 1H), 2.24 (s, 3H). **^13^C NMR (100 MHz, CDCl_3_)** δ 192.5, 176.6, 169.7, 150.5, 142.8, 139.3, 136.6, 136.4, 136.3, 131.3, 128.4, 128.1, 128.0, 126.3, 125.6, 123.7, 123.6, 123.1, 122.4, 109.5, 79.3, 62.2, 57.4 (q, *J* = 23.4 Hz), 49.4, 48.7, 33.7, 26.1, 21.8. **^19^F NMR (376 MHz, CDCl_3_)** δ -67.7. **HRMS** (ESI) Calcd for C_30_H_23_F_3_NO_4_^+^ [M+H]^+^ 518.1574; Found: 518.1573.

**(2a*S*,3*R*,4R,5a*R*,10b*R*)-7'-bromo-1'-methyl-3-phenyl-5a-(trifluoromethyl)-2a,3,5,5a-tetrahydro-2*H*,6*H*-spiro[fluoreno[4a,4-*b*]oxete-4,3'-indoline]-2,2',6-trione (3f):** 81% yield, >99% ee, >20:1 dr. [α]_D_^23^ (c 0.5, CHCl_3_) = + 1.5.

**HPLC condition:** Chiralpak AD (Hex/*i*PrOH = 85/15, 1.0 mL/min, t_R_ (major) = 13.2 min, t_R_ (minor) = 10.7 min).

**^1^H NMR (400 MHz, CDCl_3_)** δ 7.96 (d, *J* = 7.6 Hz, 1H), 7.84-7.89 (m, 2H), 7.69-7.73 (m, 1H), 7.24 (d, *J* = 8.0 Hz, 2H), 7.02-7.10 (m, 3H), 6.91 (t, *J* = 8.0 Hz, 1H), 6.80-6.82 (m, 2H), 4.38 (d, *J* = 10.4 Hz, 1H), 3.57 (d, *J* = 10.4 Hz, 1H), 3.26 (s, 3H), 2.87 (d, *J* = 14.8 Hz, 1H), 2.64 (d, *J* = 14.8 Hz, 1H). **^13^C NMR (100 MHz, CDCl_3_)** δ 192.2, 176.8, 169.4, 150.4, 140.2, 136.5, 136.4, 135.8, 134.7, 132.4, 131.4, 128.6, 128.4, 128.0, 124.2, 123.7, 123.1, 121.8, 102.8, 79.2, 62.0, 57.3 (q, *J* = 23.4 Hz), 49.4, 48.9, 33.7, 29.7. **^19^F NMR (376 MHz, CDCl_3_)** δ -67.7. **HRMS** (ESI) Calcd for C_29_H_20_BrF_3_NO_4_^+^ [M+H]^+^ 582.0522; Found: 582.0521.

**(2a*S*,3*R*,4*R*,5a*R*,10b*R*)-1'-benzyl-3-phenyl-5a-(trifluoromethyl)-2a,3,5,5a-tetrahydro-2*H*,6*H*-spiro[fluoreno[4a,4-*b*]oxete-4,3'-indoline]-2,2',6-trione (3g):** 93% yield, >99% ee, >20:1 dr. [α]_D_^23^ (c 1.0, CHCl_3_) = -4.1.

**HPLC condition:** Chiralpak AS-H (Hex/*i*PrOH = 90/10, 1.0 mL/min, t_R_ (major) = 15.7 min).

**^1^H NMR (400 MHz, CDCl_3_)** δ 7.96 (d, *J* = 7.6 Hz, 1H), 7.84-7.89 (m, 2H), 7.68-7.72 (m, 1H), 7.32-7.35 (m, 1H), 7.22-7.28 (m, 3H), 6.92-7.09 (m, 9H), 6.41-6.43 (m, 1H), 4.72 (d, *J* = 16.0 Hz, 1H), 4.59 (d, *J* = 16.0 Hz, 1H), 4.50 (d, *J* = 10.4 Hz, 1H), 3.74 (d, *J* = 10.4 Hz, 1H), 2.94 (d, *J* = 14.8 Hz, 1H), 2.74 (d, *J* = 14.8 Hz, 1H). **^13^C NMR (100 MHz, CDCl_3_)** δ 192.3, 176.5, 169.7, 150.5, 142.0, 136.7, 136.4, 136.3, 134.8, 131.3, 129.4, 129.0, 128.8, 128.7, 128.4, 128.1, 127.7, 127.1, 123.7, 123.2, 123.2, 122.9, 109.9, 79.3, 62.3, 57.4 (q, *J* = 23.6 Hz), 49.9, 47.9, 43.6, 34.7. **^19^F NMR (376 MHz, CDCl_3_)** δ -67.6. **HRMS** (ESI) Calcd for C_35_H_25_F_3_NO_4_^+^ [M+H]^+^ 580.1730; Found: 580.1722.

**(2a*S*,3*R*,4*R*,5a*R*,10b*R*)-1'-allyl-3-phenyl-5a-(trifluoromethyl)-2a,3,5,5a-tetrahydro-2*H*,6*H*-spiro[fluoreno[4a,4-*b*]oxete-4,3'-indoline]-2,2',6-trione (3h):** 87% yield, >99% ee, >20:1 dr. [α]_D_^23^ (c 1.0, CHCl_3_) = + 64.7.

**HPLC condition:** Chiralpak AS-H (Hex/*i*PrOH = 85/15, 1.0 mL/min, t_R_ (major) = 13.4 min).

**^1^H NMR (400 MHz, CDCl_3_)** δ 7.94 (d, *J* = 8.4 Hz, 1H), 7.86 (d, *J* = 3.6 Hz, 2H), 7.68-7.73 (m, 1H), 7.33 (d, *J* = 7.2 Hz, 1H), 6.99-7.13 (m, 5H), 6.89-6.91 (m, 2H), 6.54 (d, *J* = 7.6 Hz, 1H), 5.44-5.54 (m, 1H), 5.05 (d, *J* = 10.4 Hz, 1H), 4.85 (d, *J* = 17.2 Hz, 1H), 4.46 (d, *J* = 10.4 Hz, 1H), 4.16-4.22 (m, 1H), 3.90-3.96 (m, 1H), 3.68 (d, *J* = 10.4 Hz, 1H), 2.88 (d, *J* = 15.2 Hz, 1H), 2.71 (d, *J* = 15.2 Hz, 1H). **^13^C NMR (100 MHz, CDCl_3_)** δ 192.3, 176.1, 169.7, 150.4, 142.0, 136.6, 136.3, 136.3, 131.3, 130.5, 129.3, 129.0, 128.5, 128.4, 128.1, 123.7, 123.1, 123.1, 122.8, 118.0, 109.7, 79.3, 62.1, 57.4 (q, *J* = 23.4 Hz), 49.7, 48.2, 42.2, 34.2. **^19^F NMR (376 MHz, CDCl_3_)** δ -67.7. **HRMS** (ESI) Calcd for C_31_H_23_F_3_NO_4_^+^ [M+H]^+^ 530.1574; Found: 530.1572.

**(2a*S*,3*R*,4*R*,5a*R*,10b*R*)-1'-isopropyl-3-phenyl-5a-(trifluoromethyl)-2a,3,5,5a-tetrahydro-2*H*,6*H*-spiro[fluoreno[4a,4-*b*]oxete-4,3'-indoline]-2,2',6-trione (3i):** 83% yield, >99% ee, >20:1 dr. [α]_D_^23^ (c 1.0, CHCl_3_) = + 95.8.

**HPLC condition:** Chiralpak AS-H (Hex/*i*PrOH = 95/5, 1.0 mL/min, t_R_ (major) = 12.9 min, t_R_ (minor) = 44.6 min).

**^1^H NMR (400 MHz, CDCl_3_)** δ 7.95 (d, *J* = 8.0 Hz, 1H), 7.86 (d, *J* = 4.0 Hz, 2H), 7.68-7.73 (m, 1H), 7.31 (d, *J* = 7.6 Hz, 1H), 6.98-7.12 (m, 5H), 6.89-6.92 (m, 2H), 6.73 (d, *J* = 7.6 Hz, 1H), 4.44 (d, *J* = 10.4 Hz, 1H), 4.34-4.42 (m, 1H), 3.63 (d, *J* = 10.4 Hz, 1H), 2.85 (d, *J* = 15.2 Hz, 1H), 2.66 (d, *J* = 15.2 Hz, 1H), 1.29 (d, *J* = 6.8 Hz, 3H), 1.12 (d, *J* = 6.8 Hz, 3H). **^13^C NMR (100 MHz, CDCl_3_)** δ 192.2, 175.9, 169.9, 150.4, 141.3, 136.7, 136.4, 136.2, 131.3, 129.9, 128.7, 128.4, 128.4, 128.0, 123.6, 123.1, 123.0, 122.5, 110.5, 79.3, 62.2, 57.4 (q, *J* = 23.5 Hz), 49.2, 48.2, 43.5, 34.2, 19.4, 18.7. **^19^F NMR (376 MHz, CDCl_3_)** δ -67.6. **HRMS** (ESI) Calcd for C_31_H_25_F_3_NO_4_^+^ [M+H]^+^ 532.1730; Found: 532.1732.

**(2a*S*,3*R*,4*R*,5a*R*,12b*R*)-1'-methyl-3-phenyl-5a-(trifluoromethyl)-2a,3,5,5a-tetrahydro-2*H*,6*H*-spiro[benzo[6,7]fluoreno[4a,4-*b*]oxete-4,3'-indoline]-2,2',6-trione (3j):** 83% yield, >99% ee, >20:1 dr. [α]_D_^23^ (c 1.0, CHCl_3_) = -49.4.

**HPLC condition:** Chiralpak OD-H (Hex/*i*PrOH = 95/5, 1.0 mL/min, t_R_ (major) = 24.0 min).

**^1^H NMR (400 MHz, CDCl_3_)** δ 8.51 (s, 1H), 8.29 (s, 1H), 8.10 (d, *J* = 8.0 Hz, 1H), 8.00 (d, *J* = 8.0 Hz, 1H), 7.62-7.71 (m, 2H), 7.32 (d, *J* = 7.2 Hz, 1H), 6.95-7.14 (m, 5H), 6.82 (d, *J* = 6.8 Hz, 2H), 6.50 (d, *J* = 7.6 Hz, 1H), 4.51 (d, *J* = 10.4 Hz, 1H), 3.63 (d, *J* = 10.4 Hz, 1H), 2.95 (d, *J* = 14.8 Hz, 1H), 2.86 (s, 3H), 2.74 (d, *J* = 14.8 Hz, 1H). **^13^C NMR (100 MHz, CDCl_3_)** δ 192.6, 176.3, 170.0, 144.4, 142.7, 137.5, 136.2, 134.3, 133.5, 130.7, 129.7, 129.4, 129.1, 128.9, 128.3, 128.1, 128.0, 128.0, 124.8, 123.2, 122.8, 108.6, 79.4, 63.0, 57.9 (q, 23.3 Hz), 49.5, 49.0, 33.4, 26.1. **^19^F NMR (376 MHz, CDCl_3_)** δ -67.4. **HRMS** (ESI) Calcd for C_33_H_23_F_3_NO_4_^+^ [M+H]^+^ 554.1574; Found: 554.1565.

**(2a*S*,3*R*,4*R*,5a*R*,10b*R*)-8,9-dichloro-1'-methyl-3-phenyl-5a-(trifluoromethyl)-2a,3,5,5a-tetrahydro-2*H*,6*H*-spiro[fluoreno[4a,4-*b*]oxete-4,3'-indoline]-2,2',6-trione (3k):** 86% yield, >99% ee, >20:1 dr. [α]_D_^23^ (c 1.0, CHCl_3_) = + 57.5.

**HPLC condition:** Chiralpak AS-H (Hex/*i*PrOH = 95/5, 0.8 mL/min, t_R_ (major) = 22.9 min).

**^1^H NMR (400 MHz, CDCl_3_)** δ 8.02 (s, 1H), 7.93 (s, 1H), 7.29 (d, *J* = 7.2 Hz, 1H), 7.14 (t, *J* = 8.8 Hz, 1H), 6.98-7.09 (m, 4H), 6.80-6.82 (m, 2H), 6.52 (d, *J* = 7.6 Hz, 1H), 4.42 (d, *J* = 10.4 Hz, 1H), 3.59 (d, *J* = 10.4 Hz, 1H), 2.90 (s, 3H), 2.84 (d, *J* = 14.8 Hz, 1H), 2.69 (d, *J* = 14.8 Hz, 1H). **^13^C NMR (100 MHz, CDCl_3_)** δ 190.2, 176.3, 168.7, 149.3, 142.6, 141.1, 136.7, 136.0, 135.8, 129.2, 129.0, 128.4, 128.2, 128.0, 125.4, 125.2, 123.3, 122.7, 108.7, 78.3, 62.3, 57.7 (q, 23.9 Hz), 49.5, 48.6, 33.3, 26.1. **^19^F NMR (376 MHz, CDCl_3_)** δ -67.7. **HRMS** (ESI) Calcd for C_29_H_19_Cl_2_F_3_NO_4_^+^ [M+H]^+^ 572.0638; Found: 572.0634.

**(2a*S*,3*R*,4*R*,5a*R*,10b*R*)-1'-benzyl-3-(4-fluorophenyl)-5a-(trifluoromethyl)-2a,3,5,5a-tetrahydro-2*H*,6*H*-spiro[fluoreno[4a,4-*b*]oxete-4,3'-indoline]-2,2',6-trione (3l):** 86% yield, >99% ee, >20:1 dr. [α]_D_^23^ (c 1.0, CHCl_3_) = + 20.4.

**HPLC condition:** Chiralpak IA (Hex/*i*PrOH =90/10, 1.0 mL/min, t_R_ (major) = 91.9 min).

**^1^H NMR (400 MHz, CDCl_3_)** δ 7.96 (d, *J* = 7.6 Hz, 1H), 7.85-7.88 (m, 2H), 7.68-7.74 (m, 1H), 7.23-7.32 (m, 4H), 7.03-7.08 (m, 2H), 6.94-6.96 (m, 2H), 6.85-6.89 (m, 2H), 6.67 (t, *J* = 8.4 Hz, 2H), 6.46-6.48 (m, 1H), 4.70 (d, *J* = 16.0 Hz, 1H), 4.56 (d, *J* = 16.0 Hz, 1H), 4.41 (d, *J* = 10.4 Hz, 1H), 3.69 (d, *J* = 10.0 Hz, 1H), 2.92 (d, *J* = 14.8 Hz, 1H), 2.70 (d, *J* = 14.8 Hz, 1H). **^13^C NMR (100 MHz, CDCl_3_)** δ 192.1, 176.3, 169.6, 162.3 (d, *J* = 250.0 Hz), 150.3, 142.0, 136.6, 136.3, 134.7, 132.2, 132.2, 131.4, 130.2, 130.1, 129.2, 129.2, 128.8, 127.9, 127.2, 123.8, 123.3, 123.1, 122.8, 115.7, 115.5, 110.0, 79.3, 62.2, 57.4 (q, *J* = 23.2 Hz), 49.9, 47.1, 43.6, 34.5. **^19^F NMR (376 MHz, CDCl_3_)** δ -67.6, -113.6 (Ar, 1F). **HRMS** (ESI) Calcd for C_35_H_24_F_4_NO_4_^+^ [M+H]^+^ 598.1636; Found: 598.1643.

**(2a*S*,3*R*,4*R*,5a*R*,10b*R*)-1'-benzyl-3-(4-chlorophenyl)-5a-(trifluoromethyl)-2a,3,5,5a-tetrahydro-2*H*,6*H*-spiro[fluoreno[4a,4-*b*]oxete-4,3'-indoline]-2,2',6-trione (3m):** 93% yield, >99% ee, >20:1 dr. [α]_D_^23^ (c 1.0, CHCl_3_) = + 40.4.

**HPLC condition:** Chiralpak AS-H (Hex/*i*PrOH = 90/10, 1.0 mL/min, t_R_ (major) = 16.7 min, t_R_ (minor) = 38.2 min).

**^1^H NMR (400 MHz, CDCl_3_)** δ 7.95 (d, *J* = 7.6 Hz, 1H), 7.83-7.87 (m, 2H), 7.67-7.71 (m, 1H), 7.32-7.34 (m, 1H), 7.21-7.26 (m, 3H), 7.03-7.09 (m, 2H), 6.83-6.95 (m, 6H), 6.46-6.48 (m, 1H), 4.69 (d, *J* = 16.0 Hz, 1H), 4.59 (d, *J* = 16.0 Hz, 1H), 4.42 (d, *J* = 10.4 Hz, 1H), 3.72 (d, *J* = 10.4 Hz, 1H), 2.92 (d, *J* = 14.8 Hz, 1H), 2.71 (d, *J* = 14.8 Hz, 1H). **^13^C NMR (100 MHz, CDCl_3_)** δ 192.1, 176.2, 169.5, 150.3, 142.0, 136.6, 136.4, 135.0, 134.6, 134.0, 131.4, 129.8, 129.3, 129.1, 128.9, 128.8, 127.9, 127.2, 123.8, 123.3, 123.1, 122.8, 110.1, 79.3, 62.1, 57.4 (q, *J* = 23.5 Hz), 49.8, 47.1, 43.6, 34.5. **^19^F NMR (376 MHz, CDCl_3_)** δ -67.6. **HRMS** (ESI) Calcd for C_35_H_24_ClF_3_NO_4_^+^ [M+H]^+^ 614.1340; Found: 614.1342.

**(2a*S*,3*R*,4*R*,5a*R*,10b*R*)-1'-benzyl-3-(4-bromophenyl)-5a-(trifluoromethyl)-2a,3,5,5a-tetrahydro-2*H*,6*H*-spiro[fluoreno[4a,4-*b*]oxete-4,3'-indoline]-2,2',6-trione (3n):** 83% yield, >99% ee, 15.0:1 dr. [α]_D_^23^ (c 1.0, CHCl_3_) = + 82.3.

**HPLC condition:** Chiralpak IB (Hex/*i*PrOH = 95/5, 1.0 mL/min, t_R_ (major) = 16.8 min, t_R_ (minor) = 15.7 min).

**^1^H NMR (400 MHz, CDCl_3_)** δ 7.95 (d, *J* = 7.6 Hz, 1H), 7.83-7.86 (m, 2H), 7.66-7.72 (m, 1H), 7.21-7.33 (m, 4H), 7.03-7.11 (m, 4H), 6.90-6.92 (m, 2H), 6.76-6.78 (m, 2H), 6.46-6.48 (m, 1H), 4.68 (d, *J* = 16.0 Hz, 1H), 4.59 (d, *J* = 16.0 Hz, 1H), 4.41 (d, *J* = 10.0 Hz, 1H), 3.69 (d, *J* = 10.4 Hz, 1H), 2.91 (d, *J* = 14.8 Hz, 1H), 2.70 (d, *J* = 14.8 Hz, 1H). **^13^C NMR (100 MHz, CDCl_3_)** δ 192.1, 176.2, 169.5, 150.3, 142.0, 136.6, 136.4, 135.5, 134.6, 131.8, 131.4, 130.2, 129.3, 129.1, 128.8, 127.9, 127.1, 123.8, 123.3, 123.1, 122.8, 122.2, 110.1, 79.2, 62.0, 57.4 (q, 23.6 Hz), 49.7, 47.2, 43.6, 34.5. **^19^F NMR (376 MHz, CDCl_3_)** δ -67.6. **HRMS** (ESI) Calcd for C_35_H_24_BrF_3_NO_4_^+^ [M+H]^+^ 658.0835; Found: 658.0843.

**(2a*S*,3*R*,4*R*,5a*R*,10b*R*)-1'-benzyl-3-(4-nitrophenyl)-5a-(trifluoromethyl)-2a,3,5,5a-tetrahydro-2*H*,6*H*-spiro[fluoreno[4a,4-*b*]oxete-4,3'-indoline]-2,2',6-trione (3o):** 78% yield, >99% ee, 13:1 dr. [α]_D_^23^ (c 1.0, CHCl_3_) = + 24.0.

**HPLC condition:** Chiralpak IB (Hex/*i*PrOH = 85/15, 1.0 mL/min, t_R_ (major) = 21.5 min, t_R_ (minor) = 16.3 min).

**^1^H NMR (400 MHz, CDCl_3_)** δ 7.97 (d, *J* = 8.0 Hz, 1H), 7.85-7.90 (m, 2H), 7.70-7.78 (m, 3H), 7.34 (d, *J* = 7.2 Hz, 1H), 7.22-7.26 (m, 3H), 6.97-7.10 (m, 6H), 6.53 (d, *J* = 7.2 Hz, 1H), 4.70 (d, *J* = 15.6 Hz, 1H), 4.55 (d, *J* = 15.6 Hz, 1H), 4.42 (d, *J* = 10.4 Hz, 1H), 3.84 (d, *J* = 10.4 Hz, 1H), 2.94 (d, *J* = 14.8 Hz, 1H), 2.73 (d, *J* = 14.8 Hz, 1H). **^13^C NMR (100 MHz, CDCl_3_)** δ 191.9, 175.9, 169.1, 150.0, 147.5, 143.8, 141.8, 136.6, 136.4, 134.6, 131.5, 129.6, 129.5, 128.9, 128.5, 128.2, 127.4, 123.9, 123.8, 123.5, 123.1, 122.8, 110.1, 79.1, 61.7, 57.4 (q, *J* = 23.8 Hz), 49.5, 47.6, 43.7, 34.4. **^19^F NMR (376 MHz, CDCl_3_)** δ -67.6. **HRMS** (ESI) Calcd for C_35_H_24_F_3_N_2_O_6_^+^ [M+H]^+^ 625.1581; Found: 625.1581.

**(2a*S*,3*R*,4*R*,5a*R*,10b*R*)-1'-benzyl-3-(p-tolyl)-5a-(trifluoromethyl)-2a,3,5,5a-tetrahydro-2*H*,6*H*-spiro[fluoreno[4a,4-*b*]oxete-4,3'-indoline]-2,2',6-trione (3p):** 87% yield, >99% ee, >20:1 dr. [α]_D_^23^ (c 1.0, CHCl_3_) = + 52.7.

**HPLC condition:** Chiralpak ID (Hex/*i*PrOH = 85/15, 1.0 mL/min, t_R_ (major) = 31.5 min, t_R_ (minor) = 9.1 min).

**^1^H NMR (400 MHz, CDCl_3_)** δ 7.95 (d, *J* = 7.6 Hz, 1H), 7.82-7.87 (m, 2H), 7.66-7.71 (m, 1H), 7.31-7.33 (m, 1H), 7.22-7.25 (m, 3H), 7.00-7.05 (m, 2H), 6.93-6.95 (m, 2H), 6.77-6.86 (m, 4H), 6.40-6.42 (m, 1H), 4.69 (d, *J* = 16.0 Hz, 1H), 4.60 (d, *J* = 16.0 Hz, 1H), 4.47 (d, *J* = 10.4 Hz, 1H), 3.69 (d, *J* = 10.4 Hz, 1H), 2.91 (d, *J* = 15.2 Hz, 1H), 2.71 (d, *J* = 15.2 Hz, 1H), 2.16 (s, 3H). **^13^C NMR (100 MHz, CDCl_3_)** δ 192.3, 176.6, 169.8, 150.5, 142.0, 137.7, 136.6, 136.3, 134.8, 133.4, 131.3, 129.6, 129.4, 128.9, 128.7, 128.3, 127.7, 127.1, 123.7, 123.1, 122.9, 109.9, 79.3, 62.4, 57.4 (q, *J* = 23.4 Hz), 49.9, 47.5, 43.6, 34.8, 21.1. **^19^F NMR (376 MHz, CDCl_3_)** δ -67.6. **HRMS** (ESI) Calcd for C_36_H_27_F_3_NO_4_^+^ [M+H]^+^ 594.1887; Found: 594.1893.

**(2a*S*,3*R*,4*R*,5a*R*,10b*R*)-1'-benzyl-3-(4-methoxyphenyl)-5a-(trifluoromethyl)-2a,3,5,5a-tetrahydro-2*H*,6*H*-spiro[fluoreno[4a,4-*b*]oxete-4,3'-indoline]-2,2',6-trione (3q):** 83% yield, >99% ee, >20:1 dr. [α]_D_^23^ (c 1.0, CHCl_3_) = + 21.9.

**HPLC condition:** Chiralpak AS-H (Hex/*i*PrOH = 90/10, 1.0 mL/min, t_R_ (major) = 25.2 min, t_R_ (minor) = 39.3 min).

**^1^H NMR (400 MHz, CDCl_3_)** δ 7.95 (d, *J* = 7.6 Hz, 1H), 7.83-7.88 (m, 2H), 7.66-7.72 (m, 1H), 7.31-7.34 (m, 1H), 7.20-7.27 (m, 3H), 7.02-7.06 (m, 2H), 6.94-6.97 (m, 2H), 6.81-6.85 (m, 2H), 6.50-6.53 (m, 2H), 6.42-6.45 (m, 1H), 4.70 (d, *J* = 16.0 Hz, 1H), 4.59 (d, *J* = 16.0 Hz, 1H), 4.44 (d, *J* = 10.4 Hz, 1H), 3.68 (d, *J* = 10.4 Hz, 1H), 3.65 (s, 3H), 2.91 (d, *J* = 15.2 Hz, 1H), 2.71 (d, *J* = 15.2 Hz, 1H). **^13^C NMR (100 MHz, CDCl_3_)** δ 192.3, 176.5, 169.8, 159.1, 150.5, 142.0, 136.6, 136.3, 134.8, 131.3, 129.6, 129.5, 128.9, 128.8, 128.3, 127.7, 127.2, 123.7, 123.2, 123.1, 122.9, 114.0, 109.9, 79.4, 62.4, 57.4 (q, *J* = 23.5 Hz), 55.2, 50.0, 47.2, 43.6, 34.6. **^19^F NMR (376 MHz, CDCl_3_)** δ -67.6. **HRMS** (ESI) Calcd for C_36_H_27_F_3_NO_5_^+^ [M+H]^+^ 610.1836; Found: 610.1836.

**(2a*S*,3*S*,4*R*,5a*R*,10b*R*)-1'-benzyl-3-(2-chlorophenyl)-5a-(trifluoromethyl)-2a,3,5,5a-tetrahydro-2*H*,6*H*-spiro[fluoreno[4a,4-*b*]oxete-4,3'-indoline]-2,2',6-trione (3r):** 91% yield, >99% ee, >20:1 dr. [α]_D_^23^ (c 1.0, CHCl_3_) = -59.6.

**HPLC condition:** Chiralpak IB (Hex/*i*PrOH = 95/5, 1.0 mL/min, t_R_ (major) = 16.6 min, t_R_ (minor) = 20.3 min).

**^1^H NMR (400 MHz, CDCl_3_)** δ 7.95 (d, *J* = 7.6 Hz, 1H), 7.82-7.86 (m, 2H), 7.66-7.72 (m, 1H), 7.51 (d, *J* = 7.2 Hz, 1H), 7.23-7.31 (m, 4H), 7.16 (d, *J* = 8.0 Hz, 1H), 6.91-7.08 (m, 6H), 6.80-6.84 (m, 1H), 6.48 (d, *J* = 6.8 Hz, 1H), 4.79 (d, *J* = 15.6 Hz, 1H), 4.58-4.64 (m, 2H), 4.32 (d, *J* = 11.2 Hz, 1H), 2.93 (d, *J* = 15.2 Hz, 1H), 2.78 (d, *J* = 15.2 Hz, 1H). **^13^C NMR (100 MHz, CDCl_3_)** δ 192.3, 176.7, 168.9, 150.4, 141.7, 136.6, 136.3, 134.9, 134.8, 134.6, 131.3, 130.1, 129.2, 129.2, 128.9, 128.6, 127.9, 127.5, 127.4, 123.7, 123.4, 123.2, 123.1, 109.6, 79.2, 62.7, 57.5 (q, *J* = 23.6 Hz), 49.7, 43.7, 42.0, 35.4. **^19^F NMR (376 MHz, CDCl_3_)** δ -67.6. **HRMS** (ESI) Calcd for C_35_H_24_ClF_3_NO_4_^+^ [M+H]^+^ 614.1340; Found: 614.1341.

**(2a*S*,3*R*,4*R*,5a*R*,10b*R*)-1'-benzyl-3-(2-methoxyphenyl)-5a-(trifluoromethyl)-2a,3,5,5a-tetrahydro-2*H*,6*H*-spiro[fluoreno[4a,4-*b*]oxete-4,3'-indoline]-2,2',6-trione (3s):** 71% yield, >99% ee, >20:1 dr. [α]_D_^23^ (c 1.0, CHCl_3_) = -44.0.

**HPLC condition:** Chiralpak ID (Hex/*i*PrOH = 85/15, 1.0 mL/min, t_R_ (major) = 24.6 min).

**^1^H NMR (400 MHz, CDCl_3_)** δ 7.94 (d, *J* = 7.6 Hz, 1H), 7.81-7.85 (m, 2H), 7.65-7.71 (m, 1H), 7.38 (d, *J* = 6.8 Hz, 1H), 7.19-7.25 (m, 3H), 6.93-7.03 (m, 5H), 6.85 (d, *J* = 7.6 Hz, 1H), 6.56-6.59 (m, 2H), 6.37 (d, *J* = 7.2 Hz, 1H), 4.73 (d, *J* = 16.0 Hz, 1H), 4.54-4.62 (m, 2H), 4.38 (d, *J* = 10.8 Hz, 1H), 3.70 (s, 3H), 2.89 (d, *J* = 14.8 Hz, 1H), 2.76 (d, *J* = 14.8 Hz, 1H). **^13^C NMR (100 MHz, CDCl_3_)** δ 192.5, 176.9, 169.7, 156.9, 150.7, 142.0, 136.6, 136.2, 134.9, 131.2, 129.3, 129.0, 128.8, 128.8, 127.7, 127.2, 125.3, 123.6, 123.5, 123.1, 122.4, 120.7, 110.9, 109.4, 79.3, 62.2, 57.5 (q, *J* = 23.5 Hz), 55.5, 49.9, 43.6, 35.0. **^19^F NMR (376 MHz, CDCl_3_)** δ -67.6. **HRMS** (ESI) Calcd for C_36_H_27_F_3_NO_5_^+^ [M+H]^+^ 610.1836; Found: 610.1837.

**(2a*S*,3*R*,4*R*,5a*R*,10b*R*)-1'-benzyl-3-(3-chlorophenyl)-5a-(trifluoromethyl)-2a,3,5,5a-tetrahydro-2*H*,6*H*-spiro[fluoreno[4a,4-*b*]oxete-4,3'-indoline]-2,2',6-trione (3t):** 93% yield, >99% ee, >20:1 dr. [α]_D_^23^ (c 1.0, CHCl_3_) = + 4.7.

**HPLC condition:** Chiralpak IB (Hex/*i*PrOH =95/5, 1.0 mL/min, t_R_ (major) = 14.3 min).

**^1^H NMR (400 MHz, CDCl_3_)** δ 7.96 (d, *J* = 7.6 Hz, 1H), 7.84-7.89 (m, 2H), 7.68-7.73 (m, 1H), 7.20-7.33 (m, 4H), 6.98-7.07 (m, 6H), 6.92 (t, *J* = 8.0 Hz, 1H), 6.82 (d, *J* = 7.6 Hz, 1H), 6.43-6.45 (m, 1H), 4.73 (d, *J* = 15.6 Hz, 1H), 4.59 (d, *J* = 15.6 Hz, 1H), 4.45 (d, *J* = 10.4 Hz, 1H), 3.71 (d, *J* = 10.4 Hz, 1H), 2.93 (d, *J* = 14.8 Hz, 1H), 2.71 (d, *J* = 14.8 Hz, 1H). **^13^C NMR (100 MHz, CDCl_3_)** δ 192.1, 176.2, 169.4, 150.3, 141.9, 138.6, 136.6, 136.4, 134.7, 134.6, 131.5, 130.0, 129.2, 129.0, 128.9, 128.5, 128.2, 127.8, 127.1, 123.8, 123.4, 123.2, 122.8, 110.2, 79.3, 62.0, 57.4 (q, *J* = 23.5 Hz), 49.7, 47.5, 43.7, 34.7. **^19^F NMR (376 MHz, CDCl_3_)** δ -67.6. **HRMS** (ESI) Calcd for C_35_H_23_ClF_3_NNaO_4_^+^ [M+Na]^+^ 636.1160; Found: 636.1162.

**(2a*S*,3*R*,4*R*,5a*R*,10b*R*)-1'-benzyl-3-(naphthalen-2-yl)-5a-(trifluoromethyl)-2a,3,5,5a-tetrahydro-2*H*,6*H*-spiro[fluoreno[4a,4-*b*]oxete-4,3'-indoline]-2,2',6-trione (3u):** 84% yield, >99% ee, >20:1 dr. [α]_D_^23^ (c 1.0, CHCl_3_) = + 80.8.

**HPLC condition:** Chiralpak IB (Hex/*i*PrOH = 95/5, 1.0 mL/min, t_R_ (major) = 18.3 min).

**^1^H NMR (400 MHz, CDCl_3_)** δ 7.99 (d, *J* = 8.0 Hz, 1H), 7.86-7.92 (m, 2H), 7.67-7.74 (m, 2H), 7.59 (d, *J* = 7.2 Hz, 1H), 7.37-7.50 (m, 5H), 7.14 (t, *J* = 7.2 Hz, 1H), 7.01-7.06 (m, 4H), 6.96 (t, *J* = 7.2 Hz, 1H), 6.82 (d, *J* = 7.6 Hz, 2H), 6.32 (d, *J* = 7.6 Hz, 1H), 4.66-4.68 (m, 3H), 3.96 (d, *J* = 10.4 Hz, 1H), 2.98 (d, *J* = 14.8 Hz, 1H), 2.81 (d, *J* = 14.8 Hz, 1H). **^13^C NMR (100 MHz, CDCl_3_)** δ 192.3, 176.6, 169.7, 150.5, 141.9, 136.7, 136.4, 134.6, 134.1, 133.2, 132.9, 131.4, 129.4, 129.1, 128.8, 128.5, 128.1, 127.6, 126.8, 126.4, 123.7, 123.3, 122.9, 110.1, 79.4, 62.3, 57.5 (q, *J* = 23.5 Hz), 50.1, 47.9, 43.7, 35.0. **^19^F NMR (376 MHz, CDCl_3_)** δ -67.5. **HRMS** (ESI) Calcd for C_39_H_27_F_3_NO_4_^+^ [M+H]^+^ 630.1887; Found: 630.1886.

**(2a*S*,3*S*,4*R*,5a*R*,10b*R*)-1'-benzyl-3-(furan-2-yl)-5a-(trifluoromethyl)-2a,3,5,5a-tetrahydro-2*H*,6*H*-spiro[fluoreno[4a,4-*b*]oxete-4,3'-indoline]-2,2',6-trione (3v):** 96% yield, >99% ee, >20:1 dr. [α]_D_^23^ (c 1.0, CHCl_3_) = + 51.2.

**HPLC condition:** Chiralpak IA (Hex/*i*PrOH = 90/10, 1.0 mL/min, t_R_ (major) = 109.8 min, t_R_ (minor) = 17.1 min).

**^1^H NMR (400 MHz, CDCl_3_)** δ 7.93 (d, *J* = 7.6 Hz, 1H), 7.82-7.87 (m, 2H), 7.65-7.69 (m, 1H), 7.19-7.32 (m, 4H), 7.05-7.13 (m, 2H), 6.97-7.00 (m, 3H), 6.52 (d, *J* = 7.2 Hz, 1H), 6.02-6.03 (m, 1H), 5.85 (d, *J* = 3.2 Hz, 1H), 4.70 (d, *J* = 16.0 Hz, 1H), 4.61 (d, *J* = 16.0 Hz, 1H), 4.56 (d, *J* = 10.0 Hz, 1H), 3.89 (d, *J* = 10.0 Hz, 1H), 2.92 (d, *J* = 14.8 Hz, 1H), 2.67 (d, *J* = 14.8 Hz, 1H). **^13^C NMR (100 MHz, CDCl_3_)** δ 192.1, 175.9, 169.5, 150.3, 149.1, 142.5, 142.3, 136.5, 136.3, 134.9, 131.3, 129.3, 129.2, 128.8, 127.7, 127.2, 123.8, 123.2, 123.1, 122.8, 110.5, 109.9, 109.2, 79.1, 59.4, 57.3 (q, *J* = 23.5 Hz), 48.5, 43.6, 41.4, 34.1. **^19^F NMR (376 MHz, CDCl_3_)** δ -67.6. **HRMS** (ESI) Calcd for C_33_H_23_F_3_NO_5_^+^ [M+H]^+^ 570.1523; Found: 570.1520.

**(2a*S*,3*S*,4*R*,5a*R*,10b*R*)-1'-benzyl-3-(thiophen-2-yl)-5a-(trifluoromethyl)-2a,3,5,5a-tetrahydro-2*H*,6*H*-spiro[fluoreno[4a,4-*b*]oxete-4,3'-indoline]-2,2',6-trione (3w):** 92% yield, >99% ee, >20:1 dr. [α]_D_^23^ (c 1.0, CHCl_3_) = -23.2.

**HPLC condition:** Chiralpak IA (Hex/*i*PrOH = 90/10, 1.0 mL/min, t_R_ (major) = 90.0 min).

**^1^H NMR (400 MHz, CDCl_3_)** δ 7.93 (d, *J* = 7.6 Hz, 1H), 7.83-7.88 (m, 2H), 7.66-7.72 (m, 1H), 7.33-7.35 (m, 1H), 7.19-7.26 (m, 3H), 7.06-7.12 (m, 2H), 6.91-6.96 (m, 3H), 6.64-6.70 (m, 2H), 6.47-6.50 (m, 1H), 4.74 (d, *J* = 16.0 Hz, 1H), 4.54 (d, *J* = 16.0 Hz, 1H), 4.49 (d, *J* = 10.0 Hz, 1H), 4.05 (d, *J* = 10.0 Hz, 1H), 2.94 (d, *J* = 15.2 Hz, 1H), 2.71 (d, *J* = 15.2 Hz, 1H). **^13^C NMR (100 MHz, CDCl_3_)** δ 192.1, 176.0, 169.3, 150.4, 142.40, 138.8, 136.5, 136.4, 134.7, 131.4, 129.4, 129.3, 128.8, 127.7, 127.1, 126.8, 126.6, 125.4, 123.8, 123.3, 123.1, 122.9, 110.1, 79.4, 63.0, 57.3 (q, *J* = 23.6 Hz), 50.1, 43.7, 42.8, 34.2. **^19^F NMR (376 MHz, CDCl_3_)** δ -67.6. **HRMS** (ESI) Calcd for C_33_H_22_F_3_NNaO_4_S^+^ [M+Na]^+^ 608.1114; Found: 608.1115.

**Methyl (2*R*,3*R*,4*S*,4a*R*,9a*R*)-1'-benzyl-9a-(difluoromethyl)-4a-hydroxy-2',9-dioxo-3-phenyl-1,3,4,4a,9,9a-hexahydrospiro[fluorene-2,3'-indoline]-4-carboxylate (5):** 78% yield, >99% ee, >20:1 dr. [α]_D_^23^ (c 1.0, CHCl_3_) = + 95.1.

**HPLC condition:** Chiralpak OD-H (Hex/*i*PrOH = 95/5, 1.0 mL/min, t_R_ (major) = 46.4 min, t_R_ (minor) = 34.8 min).

**^1^H NMR (400 MHz, CDCl_3_)** δ 7.97 (d, *J* = 7.6 Hz, 1H), 7.63-7.67 (m, 1H), 7.50-7.54 (m, 1H), 7.36-7.40 (m, 2H), 7.19-7.26 (m, 3H), 6.85-7.06 (m, 9H), 6.42-6.44 (m, 1H), 6.12 (t, *J* = 54.8 Hz, 1H), 5.92 (s, 1H), 4.79 (d, *J* = 15.6 Hz, 1H), 4.68 (d, *J* = 15.6 Hz, 1H), 4.53 (d, *J* = 12.8 Hz, 1H), 3.78 (d, *J* = 12.8 Hz, 1H), 3.20 (s, 3H), 2.75-2.79 (m, 1H), 2.35 (d, *J* = 15.2 Hz, 1H). **^13^C NMR (100 MHz, CDCl_3_)** δ 195.1, 177.2, 175.5, 156.1, 142.0, 135.4, 135.4, 135.1, 135.0, 131.4, 129.4, 128.7, 128.5, 127.8, 127.6, 127.5, 127.5, 124.5, 122.9, 122.7, 121.8, 117.8 (t, 248.8 Hz), 109.3, 59.5 (t, 17.0 Hz), 51.9, 50.7, 49.4, 47.7, 43.7, 31.6. **^19^F NMR (376 MHz, CDCl_3_)** δ -117.0 (dd, *J* = 275.6, 54.5 Hz, 1F), -130.8 (dd, *J* = 275.2, 55.6 Hz, 1F). **HRMS** (ESI) Calcd for C_36_H_30_F_2_NO_5_^+^ [M+H]^+^ 594.2087; Found: 594.2082.

**Methyl (2*R*,3*R*,4*S*,4a*R*,9a*R*)-1'-benzyl-9a-(fluoromethyl)-4a-hydroxy-2',9-dioxo-3-phenyl-1,3,4,4a,9,9a-hexahydrospiro[fluorene-2,3'-indoline]-4-carboxylate (7):** 81% yield, >98% ee, >20:1 dr. [α]_D_^23^ (c 1.0, CHCl_3_) = + 59.3.

**HPLC condition:** Chiralpak OD-H (Hex/*i*PrOH = 95/5, 1.0 mL/min, t_R_ (major) = 51.1 min, t_R_ (minor) = 37.4 min).

**^1^H NMR (400 MHz, CDCl_3_)** δ 7.98 (d, *J* = 7.6 Hz, 1H), 7.63-7.67 (m, 1H), 7.51-7.55 (m, 1H), 7.41 (d, *J* = 7.6 Hz, 1H), 7.35-7.37 (m, 1H), 7.20-7.25 (m, 3H), 6.93-7.05 (m, 5H), 6.85-6.86 (m, 4H), 6.41-6.43 (m, 1H), 5.68 (s, 1H), 4.80 (d, *J* = 16.0 Hz, 1H), 4.68 (d, *J* = 16.0 Hz, 1H), 4.55 (d, *J* = 47.2 Hz, 2H), 4.53 (d, *J* = 12.8 Hz, 1H), 3.80 (d, *J* = 12.8 Hz, 1H), 3.22 (s, 3H), 2.72 (d, *J* = 14.8 Hz, 1H), 2.19 (d, *J* = 14.8 Hz, 1H). **^13^C NMR (100 MHz, CDCl_3_)** δ 199.2, 177.4, 175.5, 155.9, 142.0, 135.8, 135.5, 134.8, 134.1, 131.7, 129.4, 128.7, 128.3, 127.8, 127.5, 127.3, 124.7, 122.8, 122.6, 122.1, 109.2, 88.1 (d, 181.2 Hz), 57.9 (d, 15.4 Hz), 51.8, 50.9, 49.7, 47.8, 43.6, 32.9. **^19^F NMR (376 MHz, CDCl_3_)** δ -223.8 (t, *J* = 48.5 Hz, 1F). **HRMS** (ESI) Calcd for C_36_H_30_FNNaO_5_^+^ [M+Na]^+^ 598.2000; Found: 598.1995.

**(2*R*,3*R*,9a*R*)-1'-benzyl-9a-(difluoromethyl)-3-phenyl-3,9a-dihydrospiro[fluorene-2,3'-indoline]-2',9(1*H*)-dione (6a):** 91% yield, >99% ee, 12.5:1 dr. [α]_D_^23^ (c 1.0, CHCl_3_) = + 9.6.

**HPLC condition:** Chiralpak IC (Hex/*i*PrOH = 85/15, 1.0 mL/min, t_R_ (major) = 20.5 min).

**^1^H NMR (400 MHz, CDCl_3_)** δ 7.80-7.84 (m, 2H), 7.70 (t, *J* = 7.6 Hz, 1H), 7.52 (d, *J* = 7.2 Hz, 1H), 7.47 (t, *J* = 7.6 Hz, 1H), 7.02-7.17 (m, 8H), 6.94-6.96 (m, 2H), 6.84 (d, *J* = 3.6 Hz, 1H), 6.74-6.76 (m, 2H), 6.30 (d, *J* = 7.6 Hz, 1H), 5.97 (t, *J* = 56.4 Hz, 1H), 4.44-4.55 (m, 2H), 4.24 (d, *J* = 3.6 Hz, 1H), 2.76 (d, *J* = 15.6 Hz, 1H), 2.56 (d, *J* = 15.6 Hz, 1H). **^13^C NMR (100 MHz, CDCl_3_)** δ 200.1, 178.4, 148.6, 142.4, 138.9, 136.0, 135.7, 135.4, 134.3, 132.7, 129.4, 129.3, 128.6, 128.4, 128.0, 127.5, 127.3, 127.2, 127.1, 124.6, 123.2, 122.8, 121.7, 117.2 (t, *J* = 251.0 Hz), 109.0, 57.9 (t, *J* = 19.0 Hz), 54.7, 49.2, 43.6, 31.6. **^19^F NMR (376 MHz, CDCl_3_)** δ -118.7 (dd, *J* = 274.1, 56.8 Hz, 1F), -121.9 (dd, *J* = 274.5, 57.9 Hz, 1F). **HRMS** (ESI) Calcd for C_34_H_26_F_2_NO_2_^+^ [M+H]^+^ 518.1926; Found: 518.1923.

**(2*R*,3*R*,9a*R*)-9a-(difluoromethyl)-1'-methyl-3-phenyl-3,9a-dihydrospiro[fluorene-2,3'-indoline]-2',9(1*H*)-dione (6b):** 91% yield, >99% ee, >20:1 dr. [α]_D_^23^ (c 0.6, CHCl_3_) = -1.1.

**HPLC condition:** Chiralpak IA (Hex/*i*PrOH = 95/5, 0.8 mL/min, t_R_ (major) = 30.7 min, t_R_ (minor) = 25.8 min).

**^1^H NMR (400 MHz, CDCl_3_)** δ 7.82 (d, *J* = 7.6 Hz, 1H), 7.78 (d, *J* = 7.6 Hz, 1H), 7.69 (t, *J* = 8.0 Hz, 1H), 7.44-7.48 (m, 2H), 7.00-7.16 (m, 5H), 6.83-6.85 (m, 2H), 6.75 (d, *J* = 3.6 Hz, 1H), 6.37-6.39 (m, 1H), 5.94 (t, *J* = 56.4 Hz, 1H), 4.11 (d, *J* = 3.6 Hz, 1H), 2.66-2.76 (m, 4H), 2.47-2.51 (m, 1H). **^13^C NMR (100 MHz, CDCl_3_)** δ 200.0, 178.2, 148.6, 143.1, 138.6, 136.0, 134.3, 132.4, 129.3, 128.9, 128.4, 127.5, 127.2, 127.1, 124.5, 123.0, 122.6, 121.6, 117.1 (t, 250.3 Hz), 107.6, 58.0 (t, 18.8 Hz), 54.5, 50.1, 30.1, 25.5. **^19^F NMR (376 MHz, CDCl_3_)** δ -118.8 (dd, *J* = 273.7, 56.4 Hz, 1F), -122.1 (dd, *J* = 274.1, 58.3 Hz, 1F). **HRMS** (ESI) Calcd for C_28_H_22_F_2_NO_2_^+^ [M+H]^+^ 442.1613; Found: 442.1609.

**(2*R*,3*R*,9a*R*)-9a-(difluoromethyl)-5'-methoxy-1'-methyl-3-phenyl-3,9a-dihydrospiro[fluorene-2,3'-indoline]-2',9(1*H*)-dione (6c):** 88% yield, >99% ee, >20:1 dr. [α]_D_^23^ (c 1.0, CHCl_3_) = + 43.8.

**HPLC condition:** Chiralpak IB (Hex/*i*PrOH = 85/15, 1.0 mL/min, t_R_ (major) = 9.8 min).

**^1^H NMR (400 MHz, CDCl_3_)** δ 7.76-7.82 (m, 2H), 7.68 (t, *J* = 7.2 Hz, 1H), 7.45 (t, *J* = 7.6 Hz, 1H), 7.03-7.08 (m, 4H), 6.86-6.88 (m, 2H), 6.74 (s, 1H), 6.66 (d, *J* = 8.0 Hz, 1H), 6.28 (t, *J* = 8.4 Hz, 1H), 5.94 (t, *J* = 56.8 Hz, 1H), 4.08 (s, 1H), 3.84 (s, 3H), 2.67-2.70 (m, 4H), 2.49 (d, *J* = 15.2 Hz, 1H). **^13^C NMR (100 MHz, CDCl_3_)** δ 200.0, 177.8, 156.1, 148.6, 138.7, 136.7, 136.0, 135.8, 134.3, 133.8, 129.3, 128.9, 127.6, 127.2, 124.5, 121.7, 117.1 (t, *J* = 251.0 Hz), 112.3, 110.8, 107.9, 58.0 (t, *J* = 19.0 Hz), 56.1, 54.9, 50.2, 30.2, 25.6. **^19^F NMR (376 MHz, CDCl_3_)** δ -118.7 (dd, *J* = 274.1, 57.9 Hz, 1F), -122.0 (dd, *J* = 274.1, 57.9 Hz, 1F). **HRMS** (ESI) Calcd for C_29_H_24_F_2_NO_3_^+^ [M+H]^+^ 472.1719; Found: 472.1723.

**(2*R*,3*R*,9a*R*)-5'-bromo-9a-(difluoromethyl)-1'-methyl-3-phenyl-3,9a-dihydrospiro[fluorene-2,3'-indoline]-2',9(1*H*)-dione (6d):** 90% yield, >99% ee, >20:1 dr. [α]_D_^23^ (c 1.0, CHCl_3_) = + 37.2.

**HPLC condition:** Chiralpak IB (Hex/*i*PrOH = 85/15, 1.0 mL/min, t_R_ (major) = 8.8 min, t_R_ (minor) = 20.9 min).

**^1^H NMR (400 MHz, CDCl_3_)** δ 7.76-7.82 (m, 2H), 7.69 (t, *J* = 7.6 Hz, 1H), 7.57 (s, 1H), 7.46 (t, *J* = 7.6 Hz, 1H), 7.24-7.27 (m, 1H), 7.04-7.11 (m, 3H), 6.85-6.86 (m, 2H), 6.71 (d, *J* = 3.2 Hz, 1H), 6.26 (d, *J* = 8.4 Hz, 1H), 5.93 (t, *J* = 56.4 Hz, 1H), 4.08 (d, *J* = 3.6 Hz, 1H), 2.65-2.69 (m, 4H), 2.48 (d, *J* = 15.2 Hz, 1H). **^13^C NMR (100 MHz, CDCl_3_)** δ 199.7, 177.6, 148.4, 142.2, 138.3, 136.0, 136.0, 134.5, 134.2, 131.3, 129.4, 128.8, 127.7, 127.4, 126.7, 126.3, 124.6, 121.7, 117.0 (t, *J* = 251.0 Hz), 114.5, 109.1, 58.0 (t, *J* = 19.0 Hz), 54.7, 50.1, 29.9, 25.7. **^19^F NMR (376 MHz, CDCl_3_)** δ -118.7 (dd, *J* = 274.1, 57.2 Hz, 1F), -121.9 (dd, *J* = 274.5, 57.5 Hz, 1F). **HRMS** (ESI) Calcd for C_28_H_21_BrF_2_NO_2_^+^ [M+H]^+^ 520.0718; Found: 520.0720.

**(2*R*,3*R*,9a*R*)-1'-benzyl-3-(3-chlorophenyl)-9a-(difluoromethyl)-3,9a-dihydrospiro[fluorene-2,3'-indoline]-2',9(1*H*)-dione (6e):** 90% yield, >99% ee, 12.0:1 dr. [α]_D_^23^ (c 1.0, CHCl_3_) = + 13.3.

**HPLC condition:** Chiralpak IB (Hex/*i*PrOH = 85/15, 1.0 mL/min, t_R_ (major) = 7.7 min).

**^1^H NMR (400 MHz, CDCl_3_)** δ 7.80-7.84 (m, 2H), 7.70 (t, *J* = 7.6 Hz, 1H), 7.52 (d, *J* = 7.2 Hz, 1H), 7.47 (t, *J* = 7.6 Hz, 1H), 7.02-7.17 (m, 8H), 6.94-6.96 (m, 2H), 6.85 (d, *J* = 3.2 Hz, 1H), 6.74-6.76 (m, 2H), 6.30 (d, *J* = 7.6 Hz, 1H), 5.97 (t, *J* = 56.4 Hz, 1H), 4.44-4.55 (m, 2H), 4.23 (d, *J* = 3.2 Hz, 1H), 2.76 (d, *J* = 15.6 Hz, 1H), 2.56 (d, *J* = 15.6 Hz, 1H). **^13^C NMR (100 MHz, CDCl_3_)** δ 200.0, 178.4, 148.6, 142.4, 138.9, 136.0, 135.7, 135.4, 134.3, 132.7, 129.4, 129.3, 128.6, 128.4, 128.0, 127.5, 127.3, 127.2, 127.0, 124.6, 123.2, 122.8, 121.7, 117.2 (t, *J* = 251.0 Hz), 109.0, 57.9 (t, *J* = 19.0 Hz), 54.7, 49.2, 43.6, 31.6. **^19^F NMR (376 MHz, CDCl_3_)** δ -118.7 (dd, *J* = 274.1, 56.4 Hz, 1F), -121.9 (dd, *J* = 274.5, 58.3 Hz, 1F). **HRMS** (ESI) Calcd for C_34_H_25_ClF_2_NO_2_^+^ [M+H]^+^ 552.1536; Found: 552.1537.

**(2*R*,3*R*,9a*R*)-1'-benzyl-9a-(difluoromethyl)-3-(p-tolyl)-3,9a-dihydrospiro[fluorene-2,3'-indoline]-2',9(1*H*)-dione (6f):** 93% yield, 94% ee, >20:1 dr. [α]_D_^23^ (c 1.0, CHCl_3_) = + 38.9.

**HPLC condition:** Chiralpak IC (Hex/*i*PrOH = 85/15, 1.0 mL/min, t_R_ (major) = 22.0 min, t_R_ (minor) = 46.0 min).

**^1^H NMR (400 MHz, CDCl_3_)** δ 7.78-7.84 (m, 2H), 7.70 (t, *J* = 7.6 Hz, 1H), 7.44-7.52 (m, 2H), 7.03-7.20 (m, 5H), 6.74-6.86 (m, 7H), 6.32 (d, *J* = 7.6 Hz, 1H), 5.96 (t, *J* = 56.4 Hz, 1H), 4.62 (d, *J* = 16.0 Hz, 1H), 4.43 (d, *J* = 16.0 Hz, 1H), 4.20 (d, *J* = 3.2 Hz, 1H), 2.74 (d, *J* = 15.2 Hz, 1H), 2.54 (d, *J* = 15.6 Hz, 1H), 2.25 (s, 3H). **^13^C NMR (100 MHz, CDCl_3_)** δ 200.1, 178.5, 148.6, 142.5, 136.6, 136.0, 135.9, 135.6, 135.5, 134.3, 132.9, 129.3, 129.2, 128.7, 128.5, 128.3, 127.9, 127.3, 127.1, 124.6, 123.2, 122.8, 121.7, 117.2 (t, *J* = 250.0 Hz), 109.1, 57.9 (t, *J* = 19.0 Hz), 54.7, 48.7, 43.6, 31.7, 21.2. **^19^F NMR (376 MHz, CDCl_3_)** δ -118.7 (dd, *J* = 274.1, 57.2 Hz, 1F), -121.9 (dd, *J* = 274.1, 57.9 Hz, 1F). **HRMS** (ESI) Calcd for C_35_H_28_F_2_NO_2_^+^ [M+H]^+^ 532.2083; Found: 532.2082.

**(2*R*,3*R*,9a*R*)-1'-benzyl-3-(4-bromophenyl)-9a-(difluoromethyl)-3,9a-dihydrospiro[fluorene-2,3'-indoline]-2',9(1*H*)-dione (6g):** 88% yield, 95% ee, >20:1 dr. [α]_D_^23^ (c 1.0, CHCl_3_) = + 11.0.

**HPLC condition:** Chiralpak IC (Hex/*i*PrOH = 85/15,1.0 mL/min, t_R_ (major) = 20.4 min, t_R_ (minor) = 41.3 min).

**^1^H NMR (400 MHz, CDCl_3_)** δ 7.80-7.84 (m, 2H), 7.68-7.72 (m, 1H), 7.52 (d, *J* = 8.4 Hz, 1H), 7.47 (t, *J* = 7.2 Hz, 1H), 7.02-7.19 (m, 8H), 6.93-6.96 (m, 2H), 6.84 (d, *J* = 3.2 Hz, 1H), 6.74-6.76 (m, 2H), 6.30 (d, *J* = 7.6 Hz, 1H), 5.97 (t, *J* = 56.0 Hz, 1H), 4.44-4.55 (m, 2H), 4.24 (d, *J* = 3.2 Hz, 1H), 2.76 (d, *J* = 15.6 Hz, 1H), 2.56 (d, *J* = 15.6 Hz, 1H). **^13^C NMR (100 MHz, CDCl_3_)** δ 200.0, 178.4, 148.6, 142.4, 138.9, 136.0, 135.7, 135.7, 135.4, 134.3, 132.7, 129.4, 129.3, 128.6, 128.4, 128.0, 127.5, 127.3, 127.2, 127.1, 124.6, 123.2, 122.8, 121.7, 117.2 (t, *J* = 250.0 Hz), 109.0, 57.9 (t, *J* = 19.0 Hz), 54.7, 49.2, 43.6, 31.6. **^19^F NMR (376 MHz, CDCl_3_)** δ -118.7 (dd, *J* = 274.1, 56.4 Hz, 1F), -121.9 (dd, *J* = 274.1, 57.2 Hz, 1F). **HRMS** (ESI) Calcd for C_34_H_24_BrF_2_NNaO_2_^+^ [M+Na]^+^ 618.0851; Found: 618.0858.

**(2*R*,3*R*,9a*R*)-1'-benzyl-9a-(difluoromethyl)-3-(naphthalen-2-yl)-3,9a-dihydrospiro[fluorene-2,3'-indoline]-2',9(1*H*)-dione (6h):** 92% yield, >99% ee, 13.5:1 dr. [α]_D_^23^ (c 1.0, CHCl_3_) = + 116.4.

**HPLC condition:** Chiralpak IC (Hex/*i*PrOH = 85/15,1.0 mL/min, t_R_ (major) = 24.0 min, t_R_ (minor) = 62.0 min).

**^1^H NMR (400 MHz, CDCl_3_)** δ 7.84-7.87 (m, 2H), 7.71-7.76 (m, 2H), 7.61-7.66 (m, 2H), 7.40-7.54 (m, 5H), 7.14 (t, *J* = 7.6 Hz, 1H), 6.98-7.04 (m, 4H), 6.75 (t, *J* = 7.2 Hz, 2H), 6.48 (d, *J* = 7.6 Hz, 2H), 6.18 (d, *J* = 8.0 Hz, 1H), 6.01 (t, *J* = 56.4 Hz, 1H), 4.60 (d, *J* = 16.0 Hz, 1H), 4.44 (d, *J* = 2.8 Hz, 1H), 4.34 (d, *J* = 16.0 Hz, 1H), 2.82 (d, *J* = 15.6 Hz, 1H), 2.61 (d, *J* = 16.0 Hz, 1H). **^13^C NMR (100 MHz, CDCl_3_)** δ 200.0, 178.5, 148.6, 142.4, 136.7, 136.1, 135.8, 135.1, 134.3, 133.2, 132.7, 132.5, 129.4, 128.5, 128.4, 128.1, 127.7, 127.6, 127.6, 127.5, 127.1, 126.6, 126.2, 126.0, 124.6, 123.3, 122.9, 121.8, 117.2 (t, *J* = 250.0 Hz), 109.3, 58.0 (t, *J* = 19.0 Hz), 54.8, 49.0, 43.6, 31.9. **^19^F NMR (376 MHz, CDCl_3_)** δ -118.6 (dd, *J* = 274.5, 57.5 Hz, 1F), -121.8 (dd, *J* = 274.1, 57.9 Hz, 1F). **HRMS** (ESI) Calcd for C_38_H_28_F_2_NO_2_^+^ [M+H]^+^ 568.2083; Found: 568.2090.

**(2*R*,3*S*,9a*R*)-1'-benzyl-9a-(difluoromethyl)-3-(furan-2-yl)-3,9a-dihydrospiro[fluorene-2,3'-indoline]-2',9(1*H*)-dione (6i):** 89% yield, 99% ee, 13.0:1 dr. [α]_D_^23^ (c 0.5, CHCl_3_) = + 7.6.

**HPLC condition:** Chiralpak IB (Hex/*i*PrOH = 85/15, 1.0 mL/min, t_R_ (major) = 8.5 min, t_R_ (minor) = 13.7 min).

**^1^H NMR (400 MHz, CDCl_3_)** δ 7.81 (d, *J* = 8.0 Hz, 2H), 7.69 (t, *J* = 7.6 Hz, 1H), 7.44-7.47 (m, 2H), 7.19-7.29 (m, 3H), 6.98-7.12 (m, 5H), 6.83 (d, *J* = 3.2 Hz, 1H), 6.48-6.51 (m, 1H), 5.78-6.07 (m, 2H), 5.74 (d, *J* = 2.8 Hz, 1H), 4.69 (d, *J* = 15.6 Hz, 1H), 4.54 (d, *J* = 15.6 Hz, 1H), 4.41 (d, *J* = 3.6 Hz, 1H), 2.72 (d, *J* = 15.6 Hz, 1H), 2.55 (d, *J* = 15.6 Hz, 1H). **^13^C NMR (100 MHz, CDCl_3_)** δ 199.8, 178.3, 152.4, 148.4, 142.7, 142.0, 136.1, 135.9, 135.6, 134.3, 132.4, 129.5, 128.7, 128.5, 127.5, 127.3, 124.7, 124.6, 123.1, 123.0, 121.9, 117.1 (t, *J* = 250.0 Hz), 110.2, 109.0, 107.8, 57.8 (t, *J* = 18.0 Hz), 53.3, 43.7, 43.5, 31.6. **^19^F NMR (376 MHz, CDCl_3_)** δ -118.3 (dd, *J* = 274.1, 57.5 Hz, 1F), -122.2 (dd, *J* = 274.1, 57.9 Hz, 1F). **HRMS** (ESI) Calcd for C_32_H_24_F_2_NO_3_^+^ [M+H]^+^ 508.1719; Found: 508.1717.

**(2*R*,3*S*,9a*R*)-1'-benzyl-9a-(difluoromethyl)-3-(thiophen-2-yl)-3,9a-dihydrospiro[fluorene-2,3'-indoline]-2',9(1*H*)-dione (6j):** 91% yield, >99% ee, >20:1 dr. [α]_D_^23^ (c 0.6, CHCl_3_) = + 0.33.

**HPLC condition:** Chiralpak IC (Hex/*i*PrOH = 85/15, 1.0 mL/min, t_R_ (major) = 22.9 min, t_R_ (minor) = 37.7 min).

**^1^H NMR (400 MHz, CDCl_3_)** δ 7.82 (d, *J* = 8.0 Hz, 2H), 7.71 (t, *J* = 8.0 Hz, 1H), 7.45-7.51 (m, 2H), 7.10-7.19 (m, 5H), 7.01 (d, *J* = 7.2 Hz, 1H), 6.87 (d, *J* = 3.6 Hz, 1H), 6.75-6.81 (m, 3H), 6.66-6.67 (m, 1H), 6.42 (d, *J* = 7.2 Hz, 1H), 5.96 (t, *J* = 56.4 Hz, 1H), 4.47-4.66 (m, 3H), 2.75 (d, *J* = 15.6 Hz, 1H), 2.57 (d, *J* = 15.6 Hz, 1H). **^13^C NMR (100 MHz, CDCl_3_)** δ 199.9, 178.3, 148.4, 142.9, 141.3, 136.1, 135.9, 135.8, 135.4, 134.3, 132.8, 129.6, 128.7, 127.5, 127.4, 127.1, 126.6, 126.3, 124.6, 124.6, 123.2, 123.1, 121.8, 117.2 (t, *J* = 251.0 Hz), 109.2, 57.7 (t, *J* = 19.0 Hz), 54.8, 44.4, 43.6, 31.6. **^19^F NMR (376 MHz, CDCl_3_)** δ -118.4 (dd, *J* = 274.1, 56.4 Hz, 1F), -121.9 (dd, *J* = 274.5, 57.2 Hz, 1F). **HRMS** (ESI) Calcd for C_32_H_24_F_2_NO_2_S^+^ [M+H]^+^ 524.1490; Found: 524.1483.

**(2*R*,3*R*,9a*R*)-1'-benzyl-9a-(fluoromethyl)-3-phenyl-3,9a-dihydrospiro[fluorene-2,3'-indoline]-2',9(1*H*)-dione (8a):** 93% yield, >99% ee, >20:1 dr. [α]_D_^23^ (c 1.0, CHCl_3_) = -6.4.

**HPLC condition:** Chiralpak IC (Hex/*i*PrOH = 75/25, 1.0 mL/min, t_R_ (major) = 22.4 min, t_R_ (minor) = 40.2 min).

**^1^H NMR (400 MHz, CDCl_3_)** δ 7.81 (t, *J* = 8.4 Hz, 2H), 7.69 (t, *J* = 7.2 Hz, 1H), 7.44-7.49 (m, 2H), 7.03-7.16 (m, 8H), 6.95-6.97 (m, 2H), 6.79 (s, 1H), 6.71 (d, *J* = 5.6 Hz, 2H), 6.31 (d, *J* = 7.6 Hz, 1H), 4.72 (d, *J* = 47.6 Hz, 2H), 4.42-4.56 (m, 2H), 4.22 (s, 1H), 2.57 (s, 2H). **^13^C NMR (100 MHz, CDCl_3_)** δ 203.0 (d, *J* = 6.0 Hz), 178.5, 148.0, 142.5, 139.0, 139.0, 135.7, 135.5, 134.3, 133.2, 129.3, 129.3, 128.6, 128.3, 128.0, 127.3, 127.2, 127.0, 125.3, 124.4, 123.1, 122.8, 121.9, 109.1, 88.5 (d, *J* = 183.0 Hz), 55.3, 55.0 (d, *J* = 18.0 Hz), 49.1, 43.6, 34.4, 34.4. **^19^F NMR (376 MHz, CDCl_3_)** δ -219.4 (t, *J* = 47.0 Hz, 1F). **HRMS** (ESI) Calcd for C_34_H_27_FNO_2_^+^ [M+H]^+^ 500.2020; Found: 500.2029.

**(2*R*,3*R*,9a*R*)-9a-(fluoromethyl)-1'-methyl-3-phenyl-3,9a-dihydrospiro[fluorene-2,3'-indoline]-2',9(1*H*)-dione (8b):** 88% yield, >99% ee, >20:1 dr. [α]_D_^23^ (c 1.0, CHCl_3_) = + 25.2.

**HPLC condition:** Chiralpak ID (Hex/*i*PrOH = 90/10, 1.0 mL/min, t_R_ (major) = 31.0 min).

**^1^H NMR (400 MHz, CDCl_3_)** δ 7.81 (d, *J* = 7.6 Hz, 1H), 7.77 (d, *J* = 7.6 Hz, 1H), 7.67 (t, *J* = 7.2 Hz, 1H), 7.41-7.46 (m, 2H), 7.01-7.16 (m, 5H), 6.84 (d, *J* = 6.8 Hz, 2H), 6.69 (d, *J* = 3.6 Hz, 1H), 6.39 (d, *J* = 7.6 Hz, 1H), 4.68 (d, *J* = 47.6 Hz, 2H), 4.08 (d, *J* = 3.6 Hz, 1H), 2.69 (s, 3H), 2.48-2.57 (m, 2H). **^13^C NMR (100 MHz, CDCl_3_)** δ 203.0 (d, 5.7 Hz), 178.2, 148.0, 143.2, 139.1, 139.1, 138.7, 135.7, 134.3, 132.9, 129.2, 128.9, 128.3, 127.5, 127.1, 124.9, 124.4, 122.9, 122.6, 121.8, 107.6, 87.5 (d, 183.0 Hz), 55.2, 55.1 (d, 19.4 Hz), 50.1, 32.8 (d, 3.6 Hz), 25.6. **^19^F NMR (376 MHz, CDCl_3_)** δ -219.4 (t, *J* = 47.4 Hz, 1F). **HRMS** (ESI) Calcd for C_28_H_23_FNO_2_^+^ [M+H]^+^ 424.1707; Found: 424.1707.

**(2*R*,3*R*,9a*R*)-9a-(fluoromethyl)-1',6'-dimethyl-3-phenyl-3,9a-dihydrospiro[fluorene-2,3'-indoline]-2',9(1*H*)-dione (8c):** 88% yield, >99% ee, 10.0:1 dr. [α]_D_^23^ (c 1.0, CHCl_3_) = + 29.7.

**HPLC condition:** Chiralpak AD (Hex/*i*PrOH = 85/15, 1.0 mL/min, t_R_ (major) = 14.9 min, t_R_ (minor) = 8.3 min).

**^1^H NMR (400 MHz, CDCl_3_)** δ 7.79 (d, *J* = 7.6 Hz, 1H), 7.75 (d, *J* = 8.0 Hz, 1H), 7.66 (t, *J* = 7.2 Hz, 1H), 7.43 (t, *J* = 7.2 Hz, 1H), 7.27 (d, *J* = 7.2 Hz, 1H), 6.99-7.09 (m, 3H), 6.89 (d, *J* = 7.6 Hz, 1H), 6.84 (d, *J* = 7.6 Hz, 2H), 6.68 (d, *J* = 3.6 Hz, 1H), 6.21 (s, 1H), 4.67 (d, *J* = 47.6 Hz, 2H), 4.04 (d, *J* = 3.2 Hz, 1H), 2.66 (s, 3H), 2.47 (s, 2H), 2.28 (s, 3H). **^13^C NMR (100 MHz, CDCl_3_)** δ 203.1 (d, 5.6 Hz), 178.6, 148.0, 143.2, 139.0, 138.9, 138.4, 135.6, 134.3, 129.9, 129.1, 128.9, 127.6, 127.5, 127.1, 125.0, 124.4, 123.1, 122.6, 121.8, 108.6, 87.4 (d, 182.6 Hz), 55.1 (d, 18.4 Hz), 55.1, 50.0, 33.0 (d, 3.3 Hz), 25.5, 21.9. **^19^F NMR (376 MHz, CDCl_3_)** δ -219.3 (t, *J* = 48.9 Hz, 1F). **HRMS** (ESI) Calcd for C_29_H_25_FNO_2_^+^ [M+H]^+^ 438.1864; Found: 438.1861.

**(2*R*,3*R*,9a*R*)-1'-benzyl-3-(4-bromophenyl)-9a-(fluoromethyl)-3,9a-dihydrospiro[fluorene-2,3'-indoline]-2',9(1*H*)-dione (8d):** 91% yield, >99% ee, 11:1 dr. [α]_D_^23^ (c 1.0, CHCl_3_) = + 47.9.

**HPLC condition:** Chiralpak IC (Hex/*i*PrOH = 80/20, 1.0 mL/min, t_R_ (major) = 25.2 min, t_R_ (minor) = 54.4 min).

**^1^H NMR (400 MHz, CDCl_3_)** δ 7.80 (t, *J* = 9.2 Hz, 2H), 7.69 (t, *J* = 7.6 Hz, 1H), 7.44-7.48 (m, 2H), 7.21-7.26 (m, 3H), 7.06-7.13 (m, 4H), 6.80 (d, *J* = 8.0 Hz, 2H), 6.67-6.72 (m, 3H), 6.38 (d, *J* = 7.2 Hz, 1H), 4.60-4.77 (m, 3H), 4.39 (d, *J* = 16.0 Hz, 1H), 4.18 (s, 1H), 2.55 (s, 2H). **^13^C NMR (100 MHz, CDCl_3_)** δ 202.7 (d, *J* = 6.0 Hz), 178.3, 147.8, 142.5, 139.3, 138.1, 135.8, 135.3, 134.3, 132.9, 131.1, 131.0, 129.4, 128.8, 128.7, 128.5, 127.5, 127.0, 124.5, 124.5, 123.1, 122.9, 121.9, 121.3, 109.2, 87.6 (d, *J* = 183.0 Hz), 77.5, 77.2, 76.9, 55.1, 55.1 (d, *J* = 18.0 Hz), 48.4, 43.7, 34.4. **^19^F NMR (376 MHz, CDCl_3_)** δ -219.5 (t, *J* = 50.4 Hz, 1F). **HRMS** (ESI) Calcd for C_34_H_25_BrFNNaO_2_^+^ [M+Na]^+^ 600.0945; Found: 600.0944.

**(2*R*,3*R*,9a*R*)-1'-benzyl-9a-(fluoromethyl)-3-(4-methoxyphenyl)-3,9a-dihydrospiro[fluorene-2,3'-indoline]-2',9(1*H*)-dione (8e):** 89% yield, >99% ee, >20:1 dr. [α]_D_^23^ (c 1.0, CHCl_3_) = + 52.3.

**HPLC condition:** Chiralpak IC (Hex/*i*PrOH = 65/35, 1.0 mL/min, t_R_ (major) = 21.3 min).

**^1^H NMR (400 MHz, CDCl_3_)** δ 7.80 (t, *J* = 8.0 Hz, 2H), 7.68 (t, *J* = 7.6 Hz, 1H), 7.43-7.47 (m, 2H), 7.03-7.19 (m, 5H), 6.86 (d, *J* = 8.8 Hz, 2H), 6.76 (d, *J* = 3.2 Hz, 1H), 6.68-6.70 (m, 2H), 6.57-6.60 (m, 2H), 6.33 (d, *J* = 7.6 Hz, 1H), 4.77 (s, 1H), 4.61-4.66 (m, 2H), 4.41 (d, *J* = 16.0 Hz, 1H), 4.18 (d, *J* = 3.2 Hz, 1H), 3.72 (s, 3H), 2.54 (s, 2H). **^13^C NMR (100 MHz, CDCl_3_)** δ 203.1 (d, *J* = 6.0 Hz), 178.7, 158.7, 148.0, 142.5, 138.8, 135.7, 135.4, 134.3, 133.5, 131.0, 130.4, 129.2, 128.5, 128.2, 127.3, 127.0, 125.7, 124.4, 123.1, 122.8, 121.8, 113.4, 109.1, 87.3 (d, *J* = 182.0 Hz), 55.5, 55.2, 54.9 (d, *J* = 19.0 Hz), 48.2, 43.6, 34.4, 34.4. **^19^F NMR (376 MHz, CDCl_3_)** δ -219.4 (t, *J* = 47.8 Hz, 1F). **HRMS** (ESI) Calcd for C_35_H_28_FNNaO_3_^+^ [M+Na]^+^ 552.1945; Found: 552.1945.

**(2a*R*,3*S*,4*S*,5a*S*,10b*S*)-5a-fluoro-1'-methyl-3-phenyl-2a,3,5,5a-tetrahydro-2*H*,6*H*-spiro[fluoreno[4a,4-*b*]oxete-4,3'-indoline]-2,2',6-trione (10a):** 92% yield, 98% ee, 5.0:1 dr. [α]_D_^23^ (c 1.0, CHCl_3_) = + 51.8.

**HPLC condition:** Chiralpak AD-H (Hex/*i*PrOH = 80/20, 1.0 mL/min, t_R_ (major) = 32.4 min, t_R_ (minor) = 19.6 min).

**^1^H NMR (400 MHz, CDCl_3_)** δ 7.80 (d, *J* = 8.0 Hz, 1H), 7.85-7.91 (m, 2H), 7.71-7.75 (m, 1H), 7.36 (d, *J* = 8.4 Hz, 1H), 6.99-7.16 (m, 5H), 6.81-6.83 (m, 2H), 6.46 (d, *J* = 7.2 Hz, 1H), 4.46-4.49 (m, 1H), 3.78 (d, *J* = 9.6 Hz, 1H), 2.67-2.87 (m, 5H). **^13^C NMR (100 MHz, CDCl_3_)** δ 192.8 (d, *J* = 18.5 Hz), 176.6, 169.9, 147.9, 142.8, 136.8, 135.6, 135.6, 131.8, 129.8, 129.1, 128.3, 128.2, 128.1, 124.6, 124.3, 123.4, 122.9, 108.5, 92.5 (d, *J* = 190.8 Hz), 77.6 (d, *J* = 16.6 Hz), 60.7, 52.7 (d, *J* = 7.5 Hz), 49.1, 35.3 (d, *J* = 25.3 Hz), 26.0. **^19^F NMR (376 MHz, CDCl_3_)** δ -160.3. **HRMS** (ESI) Calcd for C_28_H_21_FNO_4_^+^ [M+H]^+^ 454.1449; Found: 454.1449.

**(2a*R*,3*S*,4*S*,5a*S*,10b*S*)-4',5a-difluoro-1'-methyl-3-phenyl-2a,3,5,5a-tetrahydro-2*H*,6*H*-spiro[fluoreno[4a,4-*b*]oxete-4,3'-indoline]-2,2',6-trione (10b):** 86% yield, >99% ee, 6.0:1 dr. [α]_D_^23^ (c1.0, CHCl_3_) = + 98.1.

**HPLC condition:** Chiralpak IA (Hex/*i*PrOH = 80/20, 1.0 mL/min, t_R_ (major) = 12.0 min, t_R_ (minor) = 9.6 min).

**^1^H NMR (400 MHz, CDCl_3_)** δ 8.00 (d, *J* = 7.6 Hz, 1H), 7.84-7.90 (m, 2H), 7.72 (t, *J* = 7.6 Hz, 1H), 7.01-7.14 (m, 4H), 6.91-6.92 (m, 2H), 6.74 (t, *J* = 9.2 Hz, 1H), 6.29 (d, *J* = 7.6 Hz, 1H), 4.39 (d, *J* = 9.6 Hz, 1H), 4.08 (d, *J* = 9.6 Hz, 1H), 2.94-3.10 (m, 2H), 2.86 (s, 3H). **^13^C NMR (100 MHz, CDCl_3_)** δ 192.4 (d, *J* = 19.0 Hz), 175.9, 169.9, 158.9 (d, *J* = 250.0 Hz), 148.4, 144.4 (d, *J* = 9.0 Hz), 136.7, 136.2, 135.7, 131.7, 131.2 (d, *J* = 8.0 Hz), 128.5, 128.4, 127.6, 124.6, 124.2, 114.9 (d, *J* = 19.0 Hz), 111.9 (d, *J* = 21.0 Hz), 104.7, 104.7, 92.0 (d, *J* = 188.0 Hz), 77.3 (d, *J* = 15.0 Hz), 61.4, 53.2 (d, *J* = 9.0 Hz), 46.9, 33.3 (d, *J* = 26.0 Hz), 26.5. **^19^F NMR (376 MHz, CDCl_3_)** δ -119.4 (Ar, 1F), -158.1. **HRMS** (ESI) Calcd for C_28_H_20_F_2_NO_4_^+^ [M+H]^+^ 472.1355; Found: 472.1351.

**(2a*R*,3*S*,4*S*,5a*S*,10b*S*)-5a-fluoro-5'-methoxy-1'-methyl-3-phenyl-2a,3,5,5a-tetrahydro-2*H*,6*H*-spiro[fluoreno[4a,4-*b*]oxete-4,3'-indoline]-2,2',6-trione (10c):** 96% yield, 98% ee, 10.5:1 dr. [α]_D_^23^ (c 1.0, CHCl_3_) = + 79.5.

**HPLC condition:** Chiralpak AD (Hex/*i*PrOH = 80/20, 1.0 mL/min, t_R_ (major) = 51.8 min, t_R_ (minor) = 24.1 min).

**^1^H NMR (400 MHz, CDCl_3_)** δ 7.99 (d, *J* = 7.6 Hz, 1H), 7.85-7.90 (m, 2H), 7.72 (t, *J* = 8.0 Hz, 1H), 6.97-7.07 (m, 4H), 6.85 (d, *J* = 7.2 Hz, 2H), 6.64-6.67 (m, 1H), 6.37 (d, *J* = 8.4 Hz, 1H), 4.47 (d, *J* = 10.0 Hz, 1H), 3.80 (s, 3H), 3.75 (d, *J* = 9.6 Hz, 1H), 2.64-2.86 (m, 5H). **^13^C NMR (100 MHz, CDCl_3_)** δ 192.8 (d, *J* = 18.0 Hz), 176.1, 169.9, 156.5, 147.8, 136.8, 136.2, 135.7, 135.6, 131.8, 131.1, 128.3, 128.1, 124.6, 124.3, 113.3, 110.2, 109.0, 92.6 (d, *J* = 191.0 Hz), 77.5 (d, *J* = 30.0 Hz), 60.8, 56.0, 53.01 (d, *J* = 8.0 Hz), 49.1, 35.3 (d, *J* = 25.0 Hz), 26.1. **^19^F NMR (376 MHz, CDCl_3_)** δ -160.4. **HRMS** (ESI) Calcd for C_29_H_23_FNO_5_^+^ [M+H]^+^ 484.1555; Found: 484.1552.

**(2a*R*,3*S*,4*S*,5a*S*,10b*S*)-5'-chloro-5a-fluoro-1'-methyl-3-phenyl-2a,3,5,5a-tetrahydro-2*H*,6*H*-spiro[fluoreno[4a,4-*b*]oxete-4,3'-indoline]-2,2',6-trione (10d):** 93% yield, 99% ee, 9.2:1 dr. [α]_D_^23^ (c 1.0, CHCl_3_) = + 139.3.

**HPLC condition:** Chiralpak IB (Hex/*i*PrOH = 80/20, 1.0 mL/min, t_R_ (major) = 15.1 min, t_R_ (minor) = 11.2 min).

**^1^H NMR (400 MHz, CDCl_3_)** δ 7.99 (d, *J* = 7.6 Hz, 1H), 7.86-7.91 (m, 2H), 7.71-7.75 (m, 1H), 7.38 (d, *J* = 2.0 Hz, 1H), 7.02-7.13 (m, 4H), 6.83-6.85 (m, 2H), 6.40 (d, *J* = 8.4 Hz, 1H), 4.46-4.48 (m, 1H), 3.77 (d, *J* = 9.6 Hz, 1H), 2.64-2.84 (m, 5H). **^13^C NMR (100 MHz, CDCl_3_)** δ 192.8 (d, *J* = 19.0 Hz), 176.2, 169.5, 147.7, 141.3, 136.9, 135.4, 135.2, 131.9, 131.6, 129.1, 128.8, 128.4, 128.3, 128.2, 124.7, 124.4, 123.5, 109.5, 92.4 (d, *J* = 192.0 Hz), 77.7 (d, *J* = 22.0 Hz), 60.5, 52.8 (d, *J* = 7.0 Hz), 49.0, 35.1 (d, *J* = 25.0 Hz), 26.2. **^19^F NMR (376 MHz, CDCl_3_)** δ -160.8. **HRMS** (ESI) Calcd for C28H19ClFNNaO4+ [M+H]^+^ 510.0879; Found: 510.0879.

**(2a*R*,3*S*,4*S*,5a*S*,10b*S*)-5a-fluoro-1',6'-dimethyl-3-phenyl-2a,3,5,5a-tetrahydro-2*H*,6*H*-spiro[fluoreno[4a,4-*b*]oxete-4,3'-indoline]-2,2',6-trione (10e):** 92% yield, 96% ee, 5.0:1 dr. [α]_D_^23^ (c 1.0, CHCl_3_) = + 56.0.

**HPLC condition:** Chiralpak IB (Hex/*i*PrOH = 90/10, 1.0 mL/min, t_R_ (major) = 19.0 min, t_R_ (minor) = 16.6 min).

**^1^H NMR (400 MHz, CDCl_3_)** δ 7.99 (d, *J* = 7.6 Hz, 1H), 7.84-7.91 (m, 2H), 7.70-7.74 (m, 1H), 7.22 (d, *J* = 7.6 Hz, 1H), 6.98-7.07 (m, 3H), 6.89 (d, *J* = 7.6 Hz, 1H), 6.82-6.84 (m, 2H), 6.29 (s, 1H), 4.45-4.48 (m, 1H), 3.76 (d, *J* = 9.6 Hz, 1H), 2.64-2.84 (m, 5H), 2.25 (s, 3H). **^13^C NMR (100 MHz, CDCl_3_)** δ 192.9 (d, *J* = 19.0 Hz), 176.9, 170.0, 147.9, 142.8, 139.3, 136.8, 135.9, 135.6, 131.8, 128.3, 128.2, 128.0, 126.8, 124.6, 124.3, 123.9, 122.6, 109.4, 92.6 (d, *J* = 191.0 Hz), 77.5 (d, *J* = 29.0 Hz), 60.9, 52.5 (d, *J* = 8.0 Hz), 49.1, 35.5 (d, *J* = 25.0 Hz), 26.0, 21.9. **^19^F NMR (376 MHz, CDCl_3_)** δ -160.1. **HRMS** (ESI) Calcd for C_29_H_23_FNO_4_^+^ [M+H]^+^ 468.1606; Found: 468.1612.

**(2a*R*,3*S*,4*S*,5a*S*,10b*S*)-7'-bromo-5a-fluoro-1'-methyl-3-phenyl-2a,3,5,5a-tetrahydro-2*H*,6*H*-spiro[fluoreno[4a,4-*b*]oxete-4,3'-indoline]-2,2',6-trione (10f):** 93% yield, >99% ee, 8.5:1 dr. [α]_D_^23^ (c 1.0, CHCl_3_) = -3.6.

**HPLC condition:** Chiralpak IB (Hex/*i*PrOH = 80/20, 1.0 mL/min, t_R_ (major) = 13.6 min, t_R_ (minor) = 12.0 min).

**^1^H NMR (400 MHz, CDCl_3_)** δ 8.00 (d, *J* = 8.0 Hz, 1H), 7.86-7.92 (m, 2H), 7.72-7.75 (m, 1H), 7.31 (d, *J* = 7.2 Hz, 1H), 7.25 (d, *J* = 8.8 Hz, 1H), 7.03-7.13 (m, 3H), 6.92-6.96 (m, 1H), 6.80 (d, *J* = 7.2 Hz, 2H), 4.45-4.48 (m, 1H), 3.75 (d, *J* = 10.0 Hz, 1H), 3.19 (s, 3H), 2.63-2.84 (m, 2H). **^13^C NMR (100 MHz, CDCl_3_)** δ 192.8 (d, *J* = 19.0 Hz), 177.1, 169.6, 147.7, 140.2, 136.9, 135.4, 135.2, 134.7, 132.9, 131.9, 128.5, 128.4, 128.2, 124.7, 124.5, 124.4, 122.0, 102.8, 92.4 (d, *J* = 191.0 Hz), 77.5 (d, *J* = 16.0 Hz), 60.5, 52.3 (d, *J* = 7.0 Hz), 49.3, 35.5 (d, *J* = 26.0 Hz), 29.7. **^19^F NMR (376 MHz, CDCl_3_)** δ -160.6. **HRMS** (ESI) Calcd for C_28_H_20_BrFNO_4_^+^ [M+H]^+^ 532.0554; Found: 532.0552.

**(2a*R*,3*S*,4*S*,5a*S*,10b*S*)-1'-benzyl-5a-fluoro-3-phenyl-2a,3,5,5a-tetrahydro-2*H*,6*H*-spiro[fluoreno[4a,4-*b*]oxete-4,3'-indoline]-2,2',6-trione (10g):** 88% yield, 94% ee, 10.0:1 dr. [α]_D_^23^ (c 1.0, CHCl_3_) = + 107.1.

**HPLC condition:** Chiralpak AS-H (Hex/*i*PrOH = 85/15, 1.0 mL/min, t_R_ (major) = 47.0 min, t_R_ (minor) = 78.5 min).

**^1^H NMR (400 MHz, CDCl_3_)** δ 8.00 (d, *J* = 7.6 Hz, 1H), 7.87-7.92 (m, 2H), 7.71-7.76 (m, 1H), 7.40 (d, *J* = 7.2 Hz, 1H), 7.19-7.21 (m, 3H), 6.99-7.11 (m, 5H), 6.91 (d, *J* = 7.6 Hz, 2H), 6.83-6.85 (m, 2H), 6.38 (d, *J* = 7.2 Hz, 1H), 4.52-4.65 (m, 3H), 3.90 (d, *J* = 10.0 Hz, 1H), 2.69-2.89 (m, 2H). **^13^C NMR (100 MHz, CDCl_3_)** δ 192.8 (d, *J* = 18.0 Hz), 176.8, 169.9, 147.8, 142.0, 136.8, 135.9, 135.6, 134.6, 131.8, 129.9, 129.0, 128.8, 128.6, 128.6, 128.1, 127.7, 127.0, 124.7, 124.4, 123.4, 123.1, 109.9, 92.5 (d, *J* = 192.0 Hz), 77.6 (d, *J* = 15.0 Hz), 60.9, 52.8 (d, *J* = 8.0 Hz), 48.2, 43.7, 36.5 (d, *J* = 25.0 Hz). **^19^F NMR (376 MHz, CDCl_3_)** δ -161.0. **HRMS** (ESI) Calcd for C_34_H_25_FNO_4_^+^ [M+H]^+^ 530.1762; Found: 530.1757.

**(2a*R*,3*S*,4*S*,5a*S*,10b*S*)-1'-benzyl-5a-fluoro-3-(4-fluorophenyl)-2a,3,5,5a-tetrahydro-2*H*,6*H*-spiro[fluoreno[4a,4-*b*]oxete-4,3'-indoline]-2,2',6-trione (10h):** 92% yield, 92% ee, 10.5:1 dr. [α]_D_^23^ (c 1.0, CHCl_3_) = +71.0.

**HPLC condition:** Chiralpak AZ-H (Hex/*i*PrOH = 80/20, 1.0 mL/min, t_R_ (major) = 41.0 min, t_R_ (minor) = 18.4 min).

**^1^H NMR (400 MHz, CDCl_3_)** δ 8.00 (d, *J* = 8.0 Hz, 1H), 7.88-7.92 (m, 2H), 7.69-7.76 (m, 1H), 7.39-7.43 (m, 1H), 7.21-7.24 (m, 3H), 7.04-7.12 (m, 2H), 6.85-6.91 (m, 4H), 6.65-6.74 (m, 2H), 6.42-6.47 (m, 1H), 4.51-4.64 (m, 3H), 3.90 (d, *J* = 10.0 Hz, 1H), 2.69-2.89 (m, 2H). **^13^C NMR (100 MHz, CDCl_3_)** δ 192.8 (d, *J* = 18.0 Hz), 176.7, 169.7, 162.3 (d, *J* = 240.0 Hz), 147.6, 142.0, 136.9, 135.5, 134.6, 131.9, 131.6, 131.6, 130.4, 130.3, 129.9, 129.2, 128.8, 127.8, 127.1, 124.8, 124.4, 123.5, 123.1, 115.6, 115.4, 109.9, 92.5 (d, *J* = 192.0 Hz), 77.7 (d, *J* = 15.0 Hz), 60.6, 52.7 (d, *J* = 7.0 Hz), 47.4, 43.7, 36.4 (d, *J* = 25.0 Hz). **^19^F NMR (376 MHz, CDCl_3_)** δ -113.5 (Ar, 1F), -161.6. **HRMS** (ESI) Calcd for C_34_H_24_F_2_NO_4_^+^ [M+H]^+^ 548.1668; Found: 548.1675.

**(2a*R*,3*S*,4*S*,5a*S*,10b*S*)-1'-benzyl-5a-fluoro-3-(4-nitrophenyl)-2a,3,5,5a-tetrahydro-2*H*,6*H*-spiro[fluoreno[4a,4-*b*]oxete-4,3'-indoline]-2,2',6-trione (10i):** 83% yield, 90% ee, 6.2:1 dr. [α]_D_^23^ (c 1.0, CHCl_3_) = + 23.1.

**HPLC condition:** Chiralpak IA (Hex/*i*PrOH = 80/20, 1.0 mL/min, t_R_ (major) = 34.9 min, t_R_ (minor) = 23.0 min).

**^1^H NMR (400 MHz, CDCl_3_)** δ 8.01 (d, *J* = 7.6 Hz, 1H), 7.89-7.94 (m, 2H), 7.73-7.82 (m, 3H), 7.42-7.44 (m, 1H), 7.18-7.27 (m, 3H), 7.10-7.14 (m, 2H), 7.04 (d, *J* = 8.8 Hz, 2H), 6.91 (d, *J* = 6.8 Hz, 2H), 6.52-6.55 (m, 1H), 4.52-4.63 (m, 3H), 4.00 (d, *J* = 10.0 Hz, 1H), 2.70-2.87 (m, 2H). **^13^C NMR (100 MHz, CDCl_3_)** δ 192.5 (d, *J* = 18.0 Hz), 176.3, 169.2, 147.5, 147.2, 143.1, 141.9, 137.0, 135.4, 134.5, 132.1, 129.7, 129.6, 129.2, 128.8, 128.2, 127.4, 124.9, 124.4, 123.8, 123.6, 123.1, 110.0, 92.4 (d, *J* = 194.0 Hz), 77.5 (d, *J* = 10.0 Hz), 60.0, 52.3 (d, *J* = 7.0 Hz), 47.8, 43.9, 36.5 (d, *J* = 25.0 Hz). **^19^F NMR (376 MHz, CDCl_3_)** δ -161.7. **HRMS** (ESI) Calcd for C_34_H_24_FN_2_O_6_^+^ [M+H]^+^ 575.1613; Found: 575.1617.

**(2a*R*,3*S*,4*S*,5a*S*,10b*S*)-1'-benzyl-3-(4-bromophenyl)-5a-fluoro-2a,3,5,5a-tetrahydro-2*H*,6*H*-spiro[fluoreno[4a,4-*b*]oxete-4,3'-indoline]-2,2',6-trione (10j):** 91% yield, 93% ee, 11.0:1 dr. [α]_D_^23^ (c 1.0, CHCl_3_) = + 57.0.

**HPLC condition:** Chiralpak AD (Hex/*i*PrOH = 80/20, 1.0 mL/min, t_R_ (major) = 22.0 min, t_R_ (minor) = 13.4 min).

**^1^H NMR (400 MHz, CDCl_3_)** δ 7.98 (d, *J* = 7.6 Hz, 1H), 7.86-7.91 (m, 2H), 7.69-7.73 (m, 1H), 7.39-7.41 (m, 1H), 7.22-7.27 (m, 3H), 7.08-7.15 (m, 4H), 6.77-6.82 (m, 4H), 6.43-6.45 (m, 1H), 4.49-4.63 (m, 3H), 3.87 (d, *J* = 10.0 Hz, 1H), 2.67-2.84 (m, 2H). **^13^C NMR (100 MHz, CDCl_3_)** δ 192.7 (d, *J* = 18.0 Hz), 176.6, 169.6, 147.5, 142.0, 136.9, 135.5, 134.9, 134.5, 132.0, 131.8, 130.4, 129.7, 129.3, 128.8, 127.8, 127.1, 124.8, 124.4, 123.6, 123.1, 122.3, 110.1, 92.5 (d, *J* = 193.0 Hz), 77.6 (d, *J* = 18.0 Hz), 60.4, 52.6 (d, *J* = 8.0 Hz), 47.5, 43.7, 36.5 (d, *J* = 25.0 Hz). **^19^F NMR (376 MHz, CDCl_3_)** δ -161.7. **HRMS** (ESI) Calcd for C_34_H_24_BrFNO_4_^+^ [M+H]^+^ 608.0867; Found: 608.0871.

**(2a*R*,3*S*,4*S*,5a*S*,10b*S*)-1'-benzyl-5a-fluoro-3-(4-methoxyphenyl)-2a,3,5,5a-tetrahydro-2*H*,6*H*-spiro[fluoreno[4a,4-*b*]oxete-4,3'-indoline]-2,2',6-trione (10k):** 90% yield, 98% ee, 9.0:1 dr. [α]_D_^23^ (c 1.0, CHCl_3_) = +23.9.

**HPLC condition:** Chiralpak IA (Hex/*i*PrOH = 80/20, 1.0 mL/min, t_R_ (major) = 23.2 min, t_R_ (minor) = 15.7 min).

**^1^H NMR (400 MHz, CDCl_3_)** δ 7.99 (d, *J* = 7.6 Hz, 1H), 7.87-7.91 (m, 2H), 7.70-7.74 (m, 1H), 7.40-7.42 (m, 1H), 7.19-7.21 (m, 3H), 7.04-7.11 (m, 2H), 6.80-6.84 (m, 4H), 6.52 (d, *J* = 8.8 Hz, 2H), 6.39-6.41 (m, 1H), 4.60 (s, 2H), 4.52-4.55 (m, 1H), 3.86 (d, *J* = 10.0 Hz, 1H), 3.66 (s, 3H), 2.67-2.85 (m, 2H). **^13^C NMR (100 MHz, CDCl_3_)** δ 192.9 (d, *J* = 19.0 Hz), 176.9, 169.9, 159.2, 147.8, 142.1, 136.8, 135.5, 134.6, 131.8, 130.2, 129.7, 129.0, 128.7, 127.7, 127.1, 124.7, 124.4, 123.4, 123.1, 113.9, 109.9, 92.6 (d, *J* = 192.0 Hz), 77.7 (d, *J* = 16.0 Hz), 60.8, 55.2, 52.9 (d, *J* = 7.0 Hz), 47.4, 43.7, 36.5 (d, *J* = 25.0 Hz). **^19^F NMR (376 MHz, CDCl_3_)** δ -161.6. **HRMS** (ESI) Calcd for C_35_H_26_FNNaO_5_^+^ [M+Na]^+^ 582.1687; Found: 560.1683.

**(2a*R*,3*S*,4*S*,5a*S*,10b*S*)-1'-benzyl-5a-fluoro-3-(p-tolyl)-2a,3,5,5a-tetrahydro-2*H*,6*H*-spiro[fluoreno[4a,4-*b*]oxete-4,3'-indoline]-2,2',6-trione (10l):** 89% yield, 95% ee, 10.2:1 dr. [α]_D_^23^ (c 1.0, CHCl_3_) = + 44.6.

**HPLC condition:** Chiralpak IA (Hex/*i*PrOH = 85/15, 1.0 mL/min, t_R_ (major) = 23.6 min, t_R_ (minor) = 15.4 min).

**^1^H NMR (400 MHz, CDCl_3_)** δ 8.00 (d, *J* = 8.0 Hz, 1H), 7.88-7.89 (m, 2H), 7.69-7.75 (m, 1H), 7.41 (d, *J* = 6.8 Hz, 1H), 7.19-7.21 (m, 3H), 7.03-7.10 (m, 2H), 6.79-6.85 (m, 6H), 6.39 (d, *J* = 6.8 Hz, 1H), 4.55-4.61 (m, 3H), 3.89 (d, *J* = 10.0 Hz, 1H), 2.69-2.87 (m, 2H), 2.19 (s, 3H). **^13^C NMR (100 MHz, CDCl_3_)** δ 192.9 (d, *J* = 18.0 Hz), 176.9, 169.9, 147.9, 142.1, 137.7, 136.8, 135.6, 134.7, 132.8, 131.8, 130.1, 129.3, 129.0, 128.7, 128.5, 127.7, 127.1, 124.7, 124.4, 123.4, 123.1, 109.9, 92.6 (d, *J* = 192.0 Hz), 77.7 (d, *J* = 16.0 Hz), 61.0, 52.9 (d, *J* = 8.0 Hz), 47.8, 43.7, 36.6 (d, *J* = 24.0 Hz), 21.1. **^19^F NMR (376 MHz, CDCl_3_)** δ -161.2. **HRMS** (ESI) Calcd for C_35_H_26_FNNaO_4_^+^ [M+H]^+^ 566.1738; Found: 566.1741.

**(2a*R*,3*S*,4*S*,5a*S*,10b*S*)-1'-benzyl-3-(4-chlorophenyl)-5a-fluoro-2a,3,5,5a-tetrahydro-2*H*,6*H*-spiro[fluoreno[4a,4-*b*]oxete-4,3'-indoline]-2,2',6-trione (10m):** 86% yield, 92% ee, 8.3:1 dr. [α]_D_^23^ (c 1.0, CHCl_3_) = + 35.5.

**HPLC condition:** Chiralpak AZ-H (Hex/*i*PrOH = 80/20, 1.0 mL/min, t_R_ (major) = 45.1 min, t_R_ (minor) = 18.0 min).

**^1^H NMR (400 MHz, CDCl_3_)** δ 8.00 (d, *J* = 7.6 Hz, 1H), 7.89-7.92 (m, 2H), 7.71-7.75 (m, 1H), 7.40-7.42 (m, 1H), 7.23-7.26 (m, 3H), 7.09-7.11 (m, 2H), 6.94-7.01 (m, 2H), 6.82-6.84 (m, 4H), 6.44-6.46 (m, 1H), 4.60 (s, 2H), 4.52 (d, *J* = 9.2 Hz, 1H), 3.89 (d, *J* = 10.0 Hz, 1H), 2.68-2.85 (m, 2H). **^13^C NMR (100 MHz, CDCl_3_)** δ 192.7 (d, *J* = 18.0 Hz), 176.6, 169.6, 147.5, 142.0, 136.9, 135.5, 134.5, 134.3, 134.0, 132.0, 130.0, 129.8, 129.3, 128.8, 127.8, 127.1, 124.8, 124.4, 123.6, 123.1, 110.0, 92.5 (d, *J* = 192.0 Hz), 77.5 (d, *J* = 31.0 Hz), 60.4, 52.7 (d, *J* = 7.0 Hz), 47.4, 43.7, 36.4 (d, *J* = 25.0 Hz). **^19^F NMR (376 MHz, CDCl_3_)** δ -161.7. **HRMS** (ESI) Calcd for C_34_H_24_ClFNO_4_^+^ [M+H]^+^ 564.1372; Found: 564.1377.

**(2a*R*,3*R*,4*S*,5a*S*,10b*S*)-1'-benzyl-3-(2-chlorophenyl)-5a-fluoro-2a,3,5,5a-tetrahydro-2*H*,6*H*-spiro[fluoreno[4a,4-*b*]oxete-4,3'-indoline]-2,2',6-trione (10n):** 89% yield, 99% ee, 10.2:1 dr. [α]_D_^23^ (c 1.0, CHCl_3_) = -20.7.

**HPLC condition:** Chiralpak AS-H (Hex/*i*PrOH = 85/15, 1.0 mL/min, t_R_ (major) = 60.0 min, t_R_ (minor) = 87.1 min).

**^1^H NMR (400 MHz, CDCl_3_)** δ 8.00 (d, *J* = 7.6 Hz, 1H), 7.83-7.89 (m, 2H), 7.71 (t, *J* = 7.2 Hz, 1H), 7.54 (d, *J* = 7.6 Hz, 1H), 7.26-7.28 (m, 3H), 7.14 (d, *J* = 8.0 Hz, 1H), 6.96-7.05 (m, 6H), 6.83 (t, *J* = 7.6 Hz, 1H), 6.48 (d, *J* = 7.6 Hz, 1H), 4.75-4.84 (m, 2H), 4.60 (d, *J* = 15.6 Hz, 1H), 4.31 (d, *J* = 10.0 Hz, 1H), 2.77-2.96 (m, 2H). **^13^C NMR (100 MHz, CDCl_3_)** δ 192.4 (d, *J* = 19.0 Hz), 176.9, 169.3, 148.3, 141.7, 136.7, 135.7, 134.8, 134.7, 134.7, 131.7, 130.0, 129.2, 129.2, 128.9, 128.7, 127.9, 127.8, 127.4, 127.4, 124.7, 124.2, 123.8, 123.3, 109.5, 92.3 (d, *J* = 190.0 Hz), 77.3 (d, *J* = 13.0 Hz), 62.3, 52.7 (d, *J* = 9.0 Hz), 43.8, 42.4, 37.1 (d, *J* = 25.0 Hz). **^19^F NMR (376 MHz, CDCl_3_)** δ -158.9. **HRMS** (ESI) Calcd for C_34_H_24_ClFNO_4_^+^ [M+H]^+^ 564.1372; Found: 564.1371.

**(2a*R*,3*S*,4*S*,5a*S*,10b*S*)-1'-benzyl-5a-fluoro-3-(2-methoxyphenyl)-2a,3,5,5a-tetrahydro-2*H*,6*H*-spiro[fluoreno[4a,4-b]oxete-4,3'-indoline]-2,2',6-trione (10o):** 95% yield, 96% ee, 7.0:1 dr. [α]_D_^23^ (c 1.0, CHCl_3_) = + 37.9.

**HPLC condition:** Chiralpak IB (Hex/*i*PrOH = 80/20, 1.0 mL/min, t_R_ (major) = 11.9 min, t_R_ (minor) = 11.0 min).

**^1^H NMR (400 MHz, CDCl_3_)** δ 8.00 (d, *J* = 7.6 Hz, 1H), 7.85-7.88 (m, 2H), 7.66-7.73 (m, 1H), 7.42-7.44 (m, 1H), 7.20-7.27 (m, 3H), 6.91-7.05 (m, 6H), 6.57-6.63 (m, 2H), 6.35-6.40 (m, 1H), 4.84 (d, *J* = 10.0 Hz, 1H), 4.71 (d, *J* = 16.0 Hz, 1H), 4.57 (d, *J* = 16.0 Hz, 1H), 4.39-4.42 (m, 1H), 3.65 (s, 3H), 2.76-2.94 (m, 2H). **^13^C NMR (100 MHz, CDCl_3_)** δ 192.7 (d, *J* = 18.0 Hz), 177.3, 170.0, 156.9, 148.5, 142.0, 136.7, 135.7, 134.8, 131.6, 129.5, 129.0, 128.8, 127.7, 127.4, 127.2, 125.1, 124.6, 124.2, 123.8, 122.5, 120.7, 110.9, 109.3, 92.5 (d, *J* = 189.0 Hz), 77.6 (d, *J* = 15.0 Hz), 61.7, 55.4, 52.9 (d, *J* = 8.0 Hz), 43.7, 36.8 (d, *J* = 26.0 Hz). **^19^F NMR (376 MHz, CDCl_3_)** δ -159.2. **HRMS** (ESI) Calcd for C_35_H_27_FNO_5_^+^ [M+H]^+^ 560.1868; Found: 560.1861.

**(2a*R*,3*S*,4*S*,5a*S*,10b*S*)-1'-benzyl-3-(3-chlorophenyl)-5a-fluoro-2a,3,5,5a-tetrahydro-2*H*,6*H*-spiro[fluoreno[4a,4-*b*]oxete-4,3'-indoline]-2,2',6-trione (10p):** 95% yield, 93% ee, 3.0:1 dr. [α]_D_^23^ (c 1.0, CHCl_3_) = + 44.7.

**HPLC condition:** Chiralpak AZ-H (Hex/*i*PrOH = 80/20, 1.0 mL/min, t_R_ (major) = 47.9 min, t_R_ (minor) = 19.5 min).

**^1^H NMR (400 MHz, CDCl_3_)** δ 8.00 (d, *J* = 7.6 Hz, 1H), 7.89-7.93 (m, 2H), 7.71-7.76 (m, 1H), 7.39-7.41 (m, 1H), 7.22-7.26 (m, 3H), 7.04-7.12 (m, 3H), 6.79-7.00 (m, 5H), 6.41-6.43 (m, 1H), 4.51-4.69 (m, 3H), 3.89 (d, *J* = 10.0 Hz, 1H), 2.69-2.89 (m, 2H). **^13^C NMR (100 MHz, CDCl_3_)** δ 192.6 (d, *J* = 18.0 Hz), 176.6, 169.6, 147.6, 142.0, 138.0, 136.9, 135.5, 134.6, 134.5, 132.0, 129.8, 129.5, 129.3, 128.9, 128.5, 127.8, 127.2, 127.0, 124.8, 124.4, 123.6, 123.0, 110.1, 92.4 (d, *J* = 192.0 Hz), 77.5 (d, *J* = 28.0 Hz), 60.5, 52.7 (d, *J* = 8.0 Hz), 47.8, 43.8, 36.5 (d, *J* = 25.0 Hz). **^19^F NMR (376 MHz, CDCl_3_)** δ -161.2. **HRMS** (ESI) Calcd for C_34_H_24_ClFNO_4_^+^ [M+H]^+^ 564.1372; Found: 564.1381.

**(2a*R*,3*S*,4*S*,5a*S*,10b*S*)-1'-benzyl-5a-fluoro-3-(naphthalen-2-yl)-2a,3,5,5a-tetrahydro-2*H*,6*H*-spiro[fluoreno[4a,4-*b*]oxete-4,3'-indoline]-2,2',6-trione (10q):** 93% yield, 98% ee, 3.0:1 dr. [α]_D_^23^ (c 1.0, CHCl_3_) = + 51.2.

**HPLC condition:** Chiralpak IB (Hex/*i*PrOH = 90/10, 1.0 mL/min, t_R_ (major) = 30.2 min, t_R_ (minor) = 27.4 min).

**^1^H NMR (400 MHz, CDCl_3_)** δ 8.03 (d, *J* = 7.6 Hz, 1H), 7.88-7.94 (m, 2H), 7.69-7.76 (m, 2H), 7.59 (d, *J* = 8.0 Hz, 1H), 7.37-7.51 (m, 5H), 6.89-7.11 (m, 6H), 6.67 (d, *J* = 7.6 Hz, 2H), 6.28 (d, *J* = 7.6 Hz, 1H), 4.74-4.77 (m, 1H), 4.54-4.65 (m, 2H), 4.13 (d, *J* = 9.6 Hz, 1H), 2.77-2.93 (m, 2H). **^13^C NMR (100 MHz, CDCl_3_)** δ 192.8 (d, *J* = 18.0 Hz), 176.9, 169.9, 147.9, 142.0, 136.9, 135.6, 134.4, 133.5, 133.2, 132.9, 131.9, 130.0, 129.1, 128.7, 128.4, 128.2, 127.9, 127.6, 127.5, 126.7, 126.5, 126.5, 126.4, 124.7, 124.5, 123.5, 123.1, 110.1, 93.5, 91.6, 77.7 (d, *J* = 15.0 Hz), 60.9, 53.0 (d, *J* = 7.0 Hz), 48.2, 43.8, 36.9, 36.8 (d, *J* = 25.0 Hz). **^19^F NMR (376 MHz, CDCl_3_)** δ -161.1. **HRMS** (ESI) Calcd for C_38_H_27_FNO_4_^+^ [M+H]^+^ 580.1919; Found: 580.1923.

**(2a*R*,3*R*,4*S*,5a*S*,10b*S*)-1'-benzyl-5a-fluoro-3-(thiophen-2-yl)-2a,3,5,5a-tetrahydro-2*H*,6*H*-spiro[fluoreno[4a,4-*b*]oxete-4,3'-indoline]-2,2',6-trione (10r):** 92% yield, 96% ee, 4.0:1 dr. [α]_D_^23^ (c 0.5, CHCl_3_) = +1.8.

**HPLC condition:** Chiralpak OD-H (Hex/*i*PrOH = 90/10, 1.0 mL/min, t_R_ (major) = 32.6 min, t_R_ (minor) = 41.0 min).

**^1^H NMR (400 MHz, CDCl_3_)** δ 7.98 (d, *J* = 7.6 Hz, 1H), 7.90 (d, *J* = 4.0 Hz, 2H), 7.70-7.76 (m, 1H), 7.41-7.43 (m, 1H), 7.20-7.23 (m, 3H), 7.11-7.16 (m, 2H), 6.99 (d, *J* = 5.2 Hz, 1H), 6.82-6.84 (m, 2H), 6.71-6.73 (m, 1H), 6.64 (d, *J* = 5.2 Hz, 1H), 6.46-6.48 (m, 1H), 4.56-4.66 (m, 3H), 4.23 (d, *J* = 10.0 Hz, 1H), 2.68-2.85 (m, 2H). **^13^C NMR (100 MHz, CDCl_3_)** δ 192.9 (d, *J* = 18.0 Hz), 176.6, 169.2, 147.4, 142.5, 138.1, 137.0, 135.3, 134.6, 132.0, 130.2, 129.3, 128.8, 127.7, 127.0, 126.8, 126.7, 125.4, 124.8, 124.5, 123.6, 123.2, 110.0, 92.5 (d, *J* = 193.0 Hz), 77.8 (d, *J* = 16.0 Hz), 52.7 (d, *J* = 7.0 Hz), 43.8, 43.1, 36.4 (d, *J* = 25.0 Hz). **^19^F NMR (376 MHz, CDCl_3_)** δ -162.8. **HRMS** (ESI) Calcd for C_32_H_23_FNO_4_S^+^ [M+H]^+^ 536.1326; Found: 536.1342.

**(2a*R*,3*R*,4*S*,5a*S*,10b*S*)-1'-benzyl-5a-fluoro-3-(furan-2-yl)-2a,3,5,5a-tetrahydro-2*H*,6*H*-spiro[fluoreno[4a,4-*b*]oxete-4,3'-indoline]-2,2',6-trione (10s):** 89% yield, 98% ee, 6.0:1 dr. [α]_D_^23^ (c 1.0, CHCl_3_) = + 41.4.

**HPLC condition:** Chiralpak IA (Hex/*i*PrOH = 80/20, 1.0 mL/min, t_R_ (major) = 22.4 min, t_R_ (minor) = 15.5 min).

**^1^H NMR (400 MHz, CDCl_3_)** δ 7.97 (d, *J* = 7.6 Hz, 1H), 7.84-7.88 (m, 2H), 7.65-7.72 (m, 1H), 7.34-7.36 (m, 1H), 7.19-7.26 (m, 3H), 7.07-7.14 (m, 2H), 6.93-7.00 (m, 3H), 6.51-6.53 (m, 1H), 6.02-6.04 (m, 1H), 5.80 (d, *J* = 3.2 Hz, 1H), 4.56-4.71 (m, 3H), 4.05 (d, *J* = 10.0 Hz, 1H), 2.68-2.89 (m, 2H). **^13^C NMR (100 MHz, CDCl_3_)** δ 192.6 (d, *J* = 19.0 Hz), 176.4, 169.7, 148.8, 147.8, 142.5, 142.3, 136.8, 135.5, 134.8, 131.8, 129.8, 129.2, 128.9, 128.8, 127.7, 127.2, 124.7, 124.4, 123.5, 122.9, 110.5, 109.9, 109.3, 92.3 (d, *J* = 191.0 Hz), 77.4, 77.3, 58.4, 51.4 (d, *J* = 8.0 Hz), 43.8, 41.8, 36.0 (d, *J* = 25.0 Hz). **^19^F NMR (376 MHz, CDCl_3_)** δ -160.5. **HRMS** (ESI) Calcd for C_32_H_23_FNO_5_^+^ [M+H]^+^ 520.1555; Found: 520.1553.

**(2*R*,3*R*,9a*R*)-1'-methyl-3-phenyl-9a-(trifluoromethyl)-3,9a-dihydrospiro[fluorene-2,3'-indoline]-2',9(1*H*)-dione (11):** 87% yield, >99% ee, >20:1 dr. [α]_D_^23^ (c 1.0, CHCl_3_) = -36.2.

**HPLC condition:** Chiralpak IA (Hex/*i*PrOH = 85/15, 1.0 mL/min, t_R_ (major) = 8.5 min, t_R_ (minor) = 7.3 min).

**^1^H NMR (400 MHz, CDCl_3_)** δ 7.83 (d, *J* = 7.6 Hz, 1H), 7.77 (d, *J* = 7.6 Hz, 1H), 7.68-7.72 (m, 1H), 7.43-7.48 (m, 2H), 7.01-7.17 (m, 5H), 6.83-6.85 (m, 2H), 6.78 (d, *J* = 3.6 Hz, 1H), 6.38-6.40 (m, 1H), 4.11 (d, *J* = 3.6 Hz, 1H), 2.81 (d, *J* = 15.6 Hz, 1H), 2.70 (s, 3H), 2.59 (d, *J* = 15.6 Hz, 1H). **^13^C NMR (100 MHz, CDCl_3_)** δ 197.2, 178.0, 148.9, 143.1, 138.3, 136.1, 135.1, 134.3, 131.9, 129.3, 128.8, 128.6, 128.0, 127.6, 127.3, 124.6, 123.0, 122.8, 121.4, 107.7, 57.3 (q, 24.6 Hz), 54.5, 49.8, 31.3, 25.6. **^19^F NMR (376 MHz, CDCl_3_)** δ -69.5. **HRMS** (ESI) Calcd for C_28_H_21_F_3_NO_2_^+^ [M+H]^+^ 460.1519; Found: 460.1517.

**(2*S*,3*S*,9a*S*)-9a-fluoro-1'-methyl-3-phenyl-3,9a-dihydrospiro[fluorene-2,3'-indoline]-2',9(1*H*)-dione (12):** 85% yield, 97% ee, 8.0:1 dr. [α]_D_^23^ (c 1.0, CHCl_3_) = - 9.5.

**HPLC condition:** Chiralpak IA (Hex/*i*PrOH = 85/15, 1.0 mL/min, t_R_ (major) = 18.8 min, t_R_ (minor) = 14.2 min).

**^1^H NMR (400 MHz, CDCl_3_)** δ 7.88 (d, *J* = 7.6 Hz, 1H), 7.72-7.78 (m, 2H), 7.61 (d, *J* = 7.2 Hz, 1H), 7.50 (t, *J* = 7.6 Hz, 1H), 7.07-7.24 (m, 5H), 6.97 (t, *J* = 3.6 Hz, 1H), 6.79-6.81 (m, 2H), 6.48 (d, *J* = 7.6 Hz, 1H), 4.26 (t, *J* = 2.8 Hz, 1H), 2.78 (t, *J* = 15.6 Hz, 1H), 2.61 (s, 3H), 2.31 (dd, *J* = 46.0, 15.6 Hz, 1H). **^13^C NMR (100 MHz, CDCl_3_)** δ 197.1 (d, 19.3 Hz), 178.1, 146.3, 143.5, 137.4, 137.1, 136.8, 133.9, 133.6, 129.9, 129.0, 129.0, 128.4, 127.7, 127.4, 125.3, 124.3, 124.2, 123.3, 122.2, 107.6, 91.9 (d, 180.6 Hz), 54.3, 50.0, 36.2 (d, 27.7 Hz), 25.7. **^19^F NMR (376 MHz, CDCl_3_)** δ -155.8. **HRMS** (ESI) Calcd for C_27_H_21_FNO_2_^+^ [M+H]^+^ 410.1551; Found: 410.1554.

**(2a*S*,3*R*,4*R*,5a*R*,10b*R*)-1'-methyl-3-phenyl-5a-((trifluoromethyl)thio)-2a,3,5,5a-tetrahydro-2H,6H-spiro[fluoreno[4a,4-b]oxete-4,3'-indoline]-2,2',6-trione (14):** 81% yield, >99% ee, >20:1 dr. [α]_D_^23^ (c 0.5, CHCl_3_) = + 80.63.

**HPLC condition:** Chiralpak ODH (Hex/*i*PrOH = 97/3, 1.0 mL/min, t_R_ (major) = 32.0 min).

**^1^H NMR (400 MHz, CDCl_3_)** δ 8.00 (d, *J* = 7.6 Hz, 1H), 7.78-7.87 (m, 2H), 7.72 (t, *J* = 6.4Hz, 1H), 7.30 (d, *J* = 7.2 Hz, 1H), 7.14 (t, *J* = 8.8 Hz, 1H), 6.97-7.08 (m, 4H), 6.83-6.85 (m, 2H), 6.52 (d, *J* = 7.6 Hz, 1H), 4.45 (d, *J* = 10.0 Hz, 1H), 3.68 (d, *J* = 10.0 Hz, 1H), 3.28 (d, *J* = 14.4 Hz, 1H), 2.89 (s, 3H), 2.88 (d, *J* = 14.4 Hz, 1H). **^13^C NMR (100 MHz, CDCl_3_)** δ 192.2, 176.0, 169.4, 147.3, 142.8, 136.2, 136.0, 135.4, 133.9, 131.6, 130.8, 129.2 (q, *J* = 309.1 Hz), 129.4, 129.0, 128.4, 128.2, 128.1, 127.7, 124.5, 123.5, 123.2, 122.7, 108.6, 80.8, 61.9, 58.2, 50.9, 48.9, 39.9, 26.1. **^19^F NMR (376 MHz, CDCl_3_)** δ -35.1. **HRMS** (ESI) Calcd for C_27_H_21_FNO_2_^+^ [M+H]^+^ 536.1138; Found: 536.1141.

**Methyl (2*R*,3*R*,4*S*,4a*R*,9a*R*)-4a-hydroxy-1'-methyl-2',9-dioxo-3-phenyl-9a-(trifluoromethyl)-1,3,4,4a,9,9a-hexahydrospiro[fluorene-2,3'-indoline]-4-carboxylate (15a):** 88% yield, >99% ee, >20:1 dr. [α]_D_^23^ (c 1.0, CHCl_3_) = + 37.2.

**HPLC condition:** Chiralpak OD-H (Hex/*i*PrOH = 98/2, 0.8 mL/min, t_R_ (major) = 51.8 min, t_R_ (minor) = 42.2 min).

**^1^H NMR (400 MHz, CDCl_3_)** δ 7.98 (d, *J* = 7.6 Hz, 1H), 7.68 (t, *J* = 7.6 Hz, 1H), 7.55 (t, *J* = 7.6 Hz, 1H), 7.43 (d, *J* = 7.6 Hz, 1H), 7.38 (d, *J* = 7.2 Hz, 1H), 7.03-7.12 (m, 2H), 6.77-6.96 (m, 5H), 6.46 (d, *J* = 7.6 Hz, 1H), 5.84 (s, 1H), 4.46 (d, *J* = 12.8 Hz, 1H), 3.74 (d, *J* = 12.4 Hz, 1H), 3.21 (s, 3H), 2.95 (s, 3H), 2.82 (d, *J* = 15.2 Hz, 1H), 2.70 (d, *J* = 14.8 Hz, 1H). **^13^C NMR (100 MHz, CDCl_3_)** δ 192.9, 177.0, 174.8, 156.5, 142.7, 135.5, 135.1, 133.7, 131.0, 129.5, 128.6, 127.6, 127.5, 124.9, 122.8, 121.8, 108.1, 61.9 (q, *J* = 20.4 Hz), 51.9, 50.7, 49.3, 48.2, 29.5, 26.0. **^19^F NMR (376 MHz, CDCl_3_)** δ -66.2. **HRMS** (ESI) Calcd for C_30_H_25_F_3_NO_5_^+^ [M+H]^+^ 536.1679; Found: 536.1670.

**(2*R*,3*R*,4*S*,4a*R*,9a*R*)-N-benzyl-4a-hydroxy-1'-methyl-2',9-dioxo-3-phenyl-9a-(trifluoromethyl)-1,3,4,4a,9,9a-hexahydrospiro[fluorene-2,3'-indoline]-4-carboxamide (15b):** 79% yield, >99% ee, >20:1 dr. [α]_D_^23^ (c 1.0, CHCl_3_) = + 219.3.

**HPLC condition:** Chiralpak OD-H (Hex/*i*PrOH = 80/20, 1.0 mL/min, t_R_ (major) = 8.7 min).

**^1^H NMR (400 MHz, CDCl_3_)** δ 7.93 (d, *J* = 7.6 Hz, 1H), 7.37-7.56 (m, 5H), 7.03-7.21 (m, 5H), 6.82-7.00 (m, 5H), 6.65 (d, *J* = 6.8 Hz, 2H), 6.50 (d, *J* = 7.2 Hz, 1H), 5.52-5.55 (m, 1H), 3.87-4.04 (m, 4H), 2.99 (s, 3H), 2.79 (d, *J* = 15.2 Hz, 1H), 2.71 (d, *J* = 15.2 Hz, 1H). **^13^C NMR (100 MHz, CDCl_3_)** δ 193.4, 177.8, 172.3, 157.1, 142.4, 136.3, 136.1, 135.2, 133.8, 132.2, 131.4, 129.2, 128.7, 128.6, 127.9, 127.7, 127.6, 126.2, 124.6, 123.0, 122.8, 122.7, 108.1, 77.8, 77.3, 62.0 (q, *J* = 20.4 Hz), 50.9, 49.31, 48.0, 43.5, 29.7, 26.1. **^19^F NMR (376 MHz, CDCl_3_)** δ -66.1. **HRMS** (ESI) Calcd for C_36_H_30_F_3_N_2_O_4_^+^ [M+H]^+^ 611.2152; Found: 611.2148.

**(3*S*,8*S*,9*S*,10*R*,13*R*,14*S*,17*R*)-10,13-dimethyl-17-((*R*)-6-methylheptan-2-yl)-2,3,4,7,8,9,10,11,12,13,14,15,16,17-tetradecahydro-1H-cyclopenta[a]phenanthren-3-yl (2*R*,3*R*,4*S*,4a*R*,9a*R*)-4a-hydroxy-1'-methyl-2',9-dioxo-3-phenyl-9a-(trifluoromethyl)-1,3,4,4a,9,9a-hexahydrospiro[fluorene-2,3'-indoline]-4-carboxylate (15c):** 86% yield, >99% ee, >20:1 dr. [α]_D_^23^ (c 2.0, CHCl_3_) = + 165.7.

**HPLC condition:** Chiralpak OD-H (Hex/*i*PrOH = 98/2, 0.8 mL/min, t_R_ (major) = 15.4 min).

**^1^H NMR (400 MHz, CDCl_3_)** δ 7.98 (d, *J* = 7.6 Hz, 1H), 7.67 (t, *J* = 7.2 Hz, 1H), 7.54 (t, *J* = 7.6 Hz, 1H), 7.48 (d, *J* = 7.6 Hz, 1H), 7.37 (d, *J* = 7.2 Hz, 1H), 7.01-7.11 (m, 2H), 6.66-6.93 (m, 5H), 6.45 (d, *J* = 7.6 Hz, 1H), 6.18 (s, 1H), 5.32 (s, 1H), 4.26-4.35 (m, 2H), 3.72 (d, *J* = 12.4 Hz, 1H), 2.95 (s, 3H), 2.81 (d, *J* = 14.8 Hz, 1H), 2.69 (d, *J* = 14.8 Hz, 1H), 1.93-2.01 (m, 4H), 1.31-1.53 (m, 11H), 1.03-1.16 (m, 6H), 0.78-0.89 (m, 18H), 0.63 (s, 3H). **^13^C NMR (100 MHz, CDCl_3_)** δ 193.0, 177.0, 173.8, 156.6, 142.6, 139.1, 135.4, 135.1, 133.7, 131.1, 129.5, 128.6, 127.5, 124.8, 123.1, 122.8, 122.2, 108.0, 77.3, 75.5, 61.8 (q, *J* = 20.3 Hz), 56.7, 56.2, 50.4, 50.0, 49.4, 48.3, 42.3, 39.7, 39.6, 37.8, 36.6, 36.5, 36.2, 35.8, 31.9, 31.8, 29.8, 29.4, 28.3, 28.1, 26.2, 26.0, 24.3, 23.9, 22.9, 22.7, 21.0, 19.2, 18.8, 11.9. **^19^F NMR (376 MHz, CDCl_3_)** δ -66.2. **HRMS** (ESI) Calcd for C_56_H_67_F_3_NO_5_^+^ [M+H]^+^ 890.4966; Found: 890.4970.

**2-(4-(3-(2-(trifluoromethyl)-10*H*-phenothiazin-10-yl)propyl)piperazin-1-yl)ethyl (2*R*,3*R*,4*S*,4a*R*,9a*R*)-4a-hydroxy-1'-methyl-2',9-dioxo-3-phenyl-9a-(trifluoromethyl)-1,3,4,4a,9,9a-hexahydrospiro[fluorene-2,3'-indoline]-4-carboxylate (15d):** 76% yield, >99% ee, >20:1 dr. [α]_D_^23^ (c 2.0, CHCl_3_) = +158.9.

**HPLC condition:** Chiralpak OD-H (Hex/*i*PrOH = 97/3, 0.8 mL/min, t_R_ (major) = 107.7 min, t_R_ (minor) = 88.3 min).

**^1^H NMR (400 MHz, CDCl_3_)** δ 7.97 (d, *J* = 7.6 Hz, 1H), 7.65 (t, *J* = 7.2 Hz, 1H), 7.49-7.55 (m, 2H), 7.39 (d, *J* = 6.8 Hz, 1H), 7.03-7.18 (m, 7H), 6.86-6.95 (m, 5H), 6.77 (d, *J* = 6.4 Hz, 2H), 6.44 (d, *J* = 7.6 Hz, 1H), 6.14 (s, 1H), 4.38 (d, *J* = 12.8 Hz, 1H), 3.94 (t, *J* = 6.8 Hz, 2H), 3.75-3.86 (m, 3H), 2.91 (s, 3H), 2.82 (d, *J* = 14.8 Hz, 1H), 2.70 (d, *J* = 14.8 Hz, 1H), 2.09-2.47 (m, 12H), 1.87-1.94 (m, 2H). **^13^C NMR (100 MHz, CDCl_3_)** δ 193.3, 176.9, 172.6, 156.5, 145.8, 144.3, 142.7, 135.8, 135.2, 133.8, 131.1, 130.1, 129.8, 129.5, 128.6, 127.8, 127.7, 127.6, 127.5, 125.7, 124.6, 124.2, 123.2, 122.8, 122.7, 122.6, 119.1, 116.0, 112.0, 108.0, 77.4, 77.3, 62.1 (q, *J* = 20.5 Hz), 61.6, 55.6, 55.2, 53.0, 52.7, 51.6, 49.4, 47.9, 45.2, 29.6, 25.9, 24.0. **^19^F NMR (376 MHz, CDCl_3_)** δ -62.2, -66.2. **HRMS** (ESI) Calcd for C_51_H_46_F_6_N_4_NaO_5_S^+^ [M+Na]^+^ 963.2985; Found: 963.2983.

**(6-(3-((1s,3s)-adamantan-1-yl)-4-methoxyphenyl)naphthalen-2-yl)methyl (2*R*,3*R*,4*S*,4a*R*,9a*R*)-4a-hydroxy-1'-methyl-2',9-dioxo-3-phenyl-9a-(trifluoromethyl)-1,3,4,4a,9,9a-hexahydrospiro[fluorene-2,3'-indoline]-4-carboxylate (15e):** 82% yield, >99% ee, >20:1 dr. [α]_D_^23^ (c 2.0, CHCl_3_) = + 416.0.

**HPLC condition:** Chiralpak IC (Hex/*i*PrOH = 95/5, 1.0 mL/min, t_R_ (major) = 149.4 min).

**^1^H NMR (400 MHz, CDCl_3_)** δ 7.96-7.68 (m, 2H), 7.76-7.81 (m*,* 3H), 7.63 (s, 1H), 7.54-7.57 (m*,*1H), 7.46-7.49 (m, 2H), 7.40 (d, *J* = 7.2 Hz, 1H), 7.32 (t, *J* = 7.6 Hz, 1H), 7.22 (d, *J* = 7.6 Hz, 1H), 7.00-7.12 (m, 4H), 6.83 (m,5H), 6.45 (d, *J* = 7.6 Hz, 1H), 5.87 (s, 1H), 5.00 (d, *J* = 12.0 Hz, 1H), 4.72 (d, *J* = 12.0 Hz, 1H), 4.51 (d, *J* = 12.8 Hz, 1H), 3.91 (s, 3H), 3.80 (d, *J* = 12.8 Hz, 1H), 2.93 (s, 3H), 2.71-2.86 (m, 2H), 2.21 (s, 6H), 2.13 (s, 3H), 1.83 (s, 6H). **^13^C NMR (100 MHz, CDCl_3_)** δ 192.9, 177.0, 174.1, 158.8, 156.3, 142.7, 139.7, 139.1, 135.5, 134.9, 133.6, 132.9, 131.8, 131.4, 131.0, 129.4, 128.7, 128.5, 128.4, 128.1, 127.6, 127.5, 126.8, 126.3, 126.0, 125.7, 124.8, 124.7, 122.8, 122.1, 112.2, 108.1, 77.4, 77.4, 67.4, 62.0 (q, *J* = 20.9 Hz), 55.3, 50.7, 49.4, 48.3, 40.7, 37.3, 37.3, 29.4, 29.2, 26.0. **^19^F NMR (376 MHz, CDCl_3_)** δ -66.2. **HRMS** (ESI) Calcd for C_57_H_51_F_3_NO_6_^+^ [M+H]^+^ 902.3663; Found: 902.3667.

**Methyl (2*S*,3*S*,4*R*,4a*S*,9a*S*)-9a-fluoro-4a-hydroxy-1'-methyl-2',9-dioxo-3-phenyl-1,3,4,4a,9,9a-hexahydrospiro[fluorene-2,3'-indoline]-4-carboxylate (16):** 93% yield, >99% ee, >20:1 dr. [α]_D_^23^ (c 1.0, CHCl_3_) = + 277.8.

**HPLC condition:** Chiralpak IA (Hex/*i*PrOH = 85/15, 1.0 mL/min, t_R_ (major) = 37.9 min).

**^1^H NMR (400 MHz, CDCl_3_)** δ 8.00 (d, *J* = 7.6 Hz, 1H), 7.71-7.75 (m, 1H), 7.57-7.62, (m, 2H), 7.43-7.45 (m*,* 1H), 7.06-7.15 (m*,*2H), 6.76-6.96 (m, 5H), 6.42-6.45 (m, 1H), 4.32-4.35 (m, 2H), 3.91 (d, *J* = 12.4 Hz, 1H), 3.37 (s, 3H), 2.85 (s, 3H), 2.61-2.82 (m, 2H). **^13^C NMR (100 MHz, CDCl_3_)** δ 192.9 (d, 18.7 Hz), 176.8, 171.9, 153.8, 142.8, 135.8, 135.6, 133.0, 130.9, 130.1, 128.7, 127.4, 127.3, 125.4, 124.8, 123.0, 122.9, 108.0, 96.2 (d, 179.3 Hz), 76.2 (d, 16.6 Hz), 51.8, 51.3 (d, 10.8 Hz), 50.6, 47.8, 31.5 (d, 25.7 Hz), 25.9. **^19^F NMR (376 MHz, CDCl_3_)** δ -145.7. **HRMS** (ESI) Calcd for C_29_H_25_FNO_5_^+^ [M+H]^+^ 486.1711; Found: 486.1711.

**Methyl (1*R*,2*S*,3*S*,4a*S*,10b*S*)-4a-fluoro-10b-hydroxy-1'-methyl-2',6-dioxo-2-phenyl-1,2,4,4a,6,10b-hexahydrospiro[benzo[*c*]chromene-3,3'-indoline]-1-carboxylate (17):** 72% yield, >99% ee, >20:1 dr. [α]_D_^23^ (c 1.0, CHCl_3_) = + 243.4.

**HPLC condition:** Chiralpak IA (Hex/*i*PrOH = 85/15, 1.0 mL/min, t_R_ (major) = 56.0 min, t_R_ (minor) = 25.4 min).

**^1^H NMR (400 MHz, CDCl_3_)** δ 7.37-7.45 (m, 3H), 7.13-7.26 (m, 4H), 6.80-7.00, (m, 5H), 6.39 (d, *J* = 8.0 Hz, 1H), 4.50-4.55 (m*,* 2H), 3.96 (d, *J* = 12.4 Hz, 1H), 3.10 (s, 3H), 2.90-2.97 (m, 2H), 2.83 (s, 3H). **^13^C NMR (100 MHz, CDCl_3_)** δ 175.7, 172.1, 161.8 (d, 23.5 Hz), 148.1, 141.5, 134.1, 132.0, 130.8, 128.9, 128.2, 127.8, 127.7, 125.9, 125.2, 124.7, 123.4, 117.7, 109.2, 91.3 (d, 177.7 Hz), 72.3 (d, 18.0 Hz), 52.1 (d, 11.9 Hz), 51.9, 49.8, 47.2, 32.8 (d, 24.1 Hz), 26.0. **^19^F NMR (376 MHz, CDCl_3_)** δ -154.2. **HRMS** (ESI) Calcd for C_29_H_25_FNO_6_^+^ [M+H]^+^ 502.1660; Found: 502.1661.

1. X-Ray Crystallographic Data of **3f** and **10j.**

X-ray crystal structure of  **3f**

Product **3f** was crystallized as a colorless crystal via slow vaporization of a hexane and DCM solution, and its absolute configuration was determined by X-ray structure analaysis. CCDC 2056654 contains the supplementary crystallographic data that can be obtained free of charge from The Cambridge Crystallographic Data Centrevia www.ccdc.cam.ac.uk/data request/cif.

X-ray crystal structure of **5j**

Product **10j** was crystallized as a colorless crystal via slow vaporization of a hexane and DCM solution, and its absolute configuration was determined by X-ray structure analaysis. CCDC 2056655 contains the supplementary crystallographic data that can be obtained free of charge from The Cambridge Crystallographic Data Centrevia www.ccdc.cam.ac.uk/data request/cif.

1. Computational Details

All theoretical calculations were performed with Gaussian 09^[4]^. All structures were completely optimized by using the M06-2X^[5]^ method and the 6-31G(d, p) basis set in mesitylene solvent, which employs the integral equation formalism polarizable continuum model (IEF-PCM)^[6]^. Frequency calculations were carried out at the same level to confirm all the optimized structures as minima (no imaginary frequency) or transition states (only one imaginary frequency), and provided the thermal relative Gibbs free energy correction. All the energies discussed in the main text are the relative Gibbs free energies (GFE), which are obtained by the addition of the thermal Gibbs free energy corrections (GFEC) at the M06-2X/6-31G(d, p)/IEFPCM_mesitylene_ level. The atom-in-molecule (AIM)^[7]^ analyses were plotted using Multiwfn (version 3.3.8)^[8]^.

1. Different conformers and configurations of **TS5**s

To ensure the selected and discussed configuration of the diastereoselectivities transition state **TS5**s with the lowest energy in the main text, we have searched multiple possible conformations for the fifth step. By rotating the dihedral angle Φ(Cα−Cβ−C1−C2) with 90° per time in stereo-controlling transition states **TS5SS**, **TS5RR**, **TS5SS'**, and **TS5RR'**, we have totally constructed 4*5=20 conformations as the initial structures, which have been subsequently optimized the structures and refined the single-point energies at the M06-2X/6-31G(d, p)/IEF-PCM_mesitylene_ and M06-2X/6-311++G(2df, 2pd)/IEF-PCM_mesitylene_ levels, respectively. After the optimization, the corresponding dihedral angles Φ(Cα−Cβ−C1−C2) change to -21°/80°/152°/-135°/, -51°/-70°/-157°/150°/, -22°/73°/-82°/152° and 46°/106°/-74°/123° in the **TS5SSs**, **TS5RRs**, **TS5SS's**, and **TS5RR'**s, respectively. As revealed by **Figure S1**, the most stable conformations with the lowest energies of the four diastereoselective transition states (denoted as **TS5SS**, **TS5RR**, **TS5SS'**, and **TS5RR'**) are associated with the dihedral angles Φ(Cα−Cβ−C1−C2) of -73°/71°/-80°/70°, separately.

**Figure S1.** Different configurations and relative energies of the transition states **TS5SS**s, **TS5RR**s, **TS5SS'**s, and **TS5RR'**s with different dihedral angles Φ Φ(Cα−Cβ−C1−C2) (energy in kcal/mol).

1. *In Vitro* Antibacterial Bioassay

The target compounds were dissolved in 150 μL DMSO and diluted with sterile distilled water containing 0.1 % Tween20 (4 mL) to prepare 250 μg/mL stock solution. Their antibacterial activities against Xanthomonas oryzae, Xanthomonas axonopodis, and Ralstonia solanacearum were evaluated by the turbidimeter test. 1 mL of stock solution was added to 4mL nutrient broth liquid medium NB (3 g of beef extract, 5 g of peptone, 1 g of yeast powder, 10 g of glucose, and 1000 mL of distilled water, pH 7.0-7.2) in tubes. Then, to the tube, 40 μL NB containing bacteria was added and incubated with continuous shaking at 180 rpm for 24 h at 30 ± 1 °C. The test concentration was fixed at 50 μg/mL (50 ppm). The data of bacterial growthwas reportedby measuring the optical density at 600 nm (OD600) with a spectrophotometer. DMSO in sterile distilled water containing 0.1 % Tween20 served as the negative control, whereas Thiodiazole-copper and bismerthiazol served as positive control. The inhibitory rate of bacterial culture growth was calculated which formula follows:

Inhibition rate (%) = (CK − T)/CK × 100.

“CK” means the value of corrected optical density of bacterial growth on untreated NB (negative control), and “T” means the value of corrected optical density of bacterial growth on treated NB.

1. Reference

[1] Q. Xie, Z. Zhu, L. Li, C. Ni, J. Hu, *Angew. Chem. Int. Ed.* **2019**, *58*, 6405 –6410.

[2] T. Ding, L. Jiang, J. Yang, Y. Xu, G. Wang, W. Yi, *Org. Lett.* **2019**, *21*, 6025−6028.

[3] G. A. Olah, Q. Wang, N. J. Trive, G. K. S. Prakash, *Synthesis* **1991**, 739-740.

[4] M. J. Frisch, G. W. Trucks, H. B. Schlegel, G. E. Scuseria, M. A. Robb, J. R. Cheeseman, G. Scalmani, V. Barone, B. Mennucci, G. A. Petersson, H. Nakatsuji, M. Caricato, X. Li, H. P. Hratchian, A. F. Izmaylov, J. Bloino, G. Zheng, J. L. Sonnenberg, M. Hada, M. Ehara, K. Toyota, R. Fukuda, M. Hada, M. Ehara, K. Toyota, R. Fukuda, J. Hasegawa, M. Ishida, T. Nakajima, Y. Honda, O. Kitao, H. Nakai, T. Vreven, J. A. Montgomery Jr., J. E. Peralta, F. Ogliaro, M. Bearpark, J. J. Heyd, E. Brothers, K. N. Kudin, V. N. taroverov, R. Kobayashi, J. Normand, K. Raghavachari, A. Rendell, J. C. Burant, S. S. Iyengar, J. Tomasi, M. Cossi, N. Rega, J. M. Millam, M. Klene, J. E. Knox, J. B. Cross, V. Bakken, C. Adamo, J. Jaramillo, R. Gomperts, R. E. Stratmann, O. Yazyev, A. J. Austin, R. Cammi, C. Pomelli, J. W. Ochterski, R. L. Martin, K. Morokuma, V. G. Zakrzewski, G. A. Voth, P. Salvador, J. J. Dannenberg, S. Dapprich, A. D. Daniels, O. Farkas, J. B. Foresman, J. V. Ortiz, J. Cioslowski, D. J. Fox. Gaussian 09, Revision C.01, Gaussian, Inc., Wallingford, CT, 2010.

[5] Y. Zhao, D. G. Truhlar, *Theor. Chem. Acc.* **2007**, 215-241.

[6] S. Miertuš, E. Scrocco, J. Tomasi, *Chem. Phys*. **1981**, 55, 117

[7] R. F. W. Bader, *Chem. Rev.* **1991**, 91, 893-928.

[8] T. Lu, F. Chen., *J. Comput. Chem.* **2012**, 580-592.

**III Copies of NMR Spectra and HPLC Chromatographs**


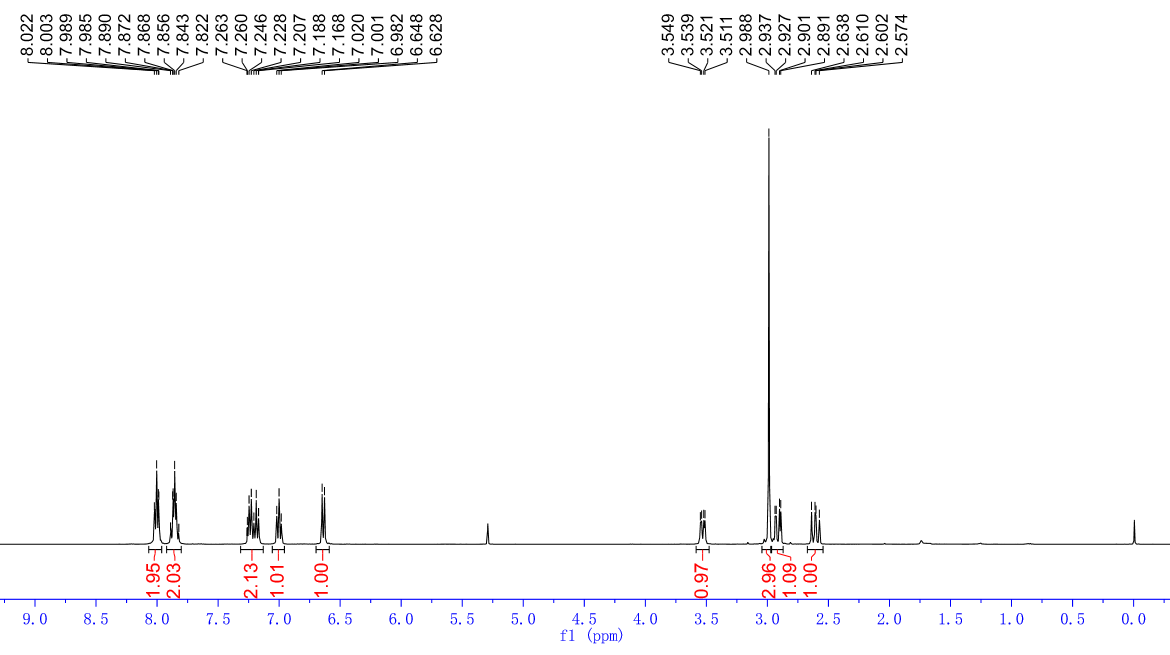


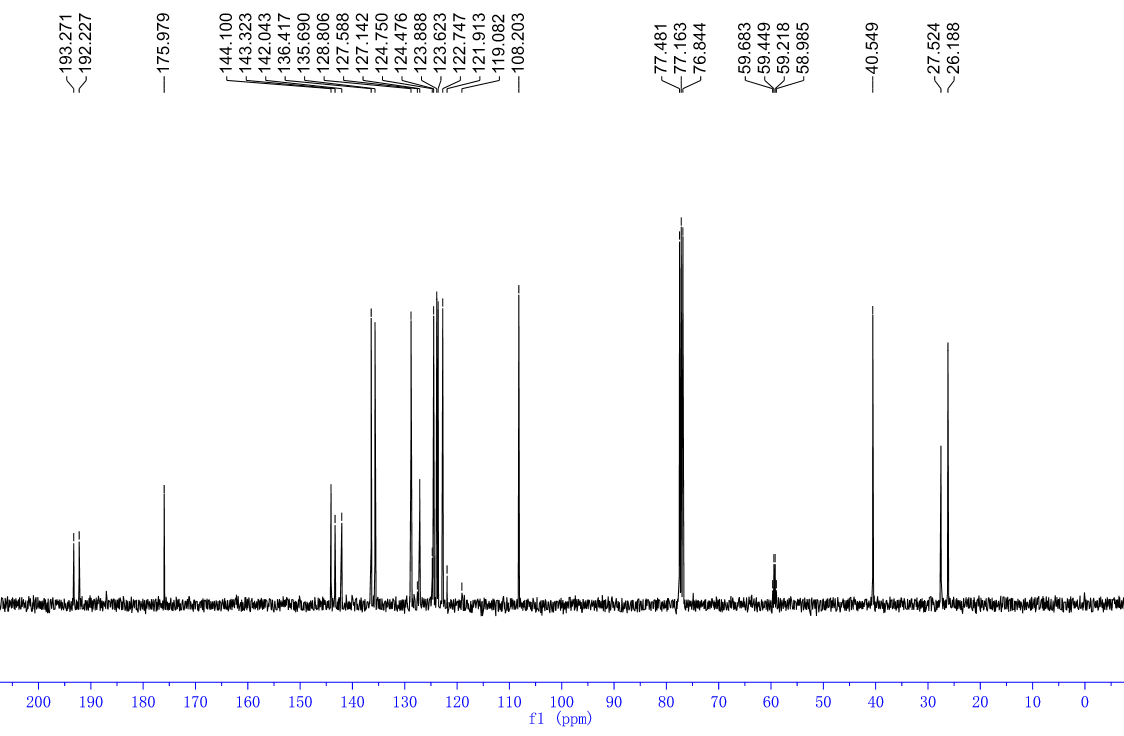

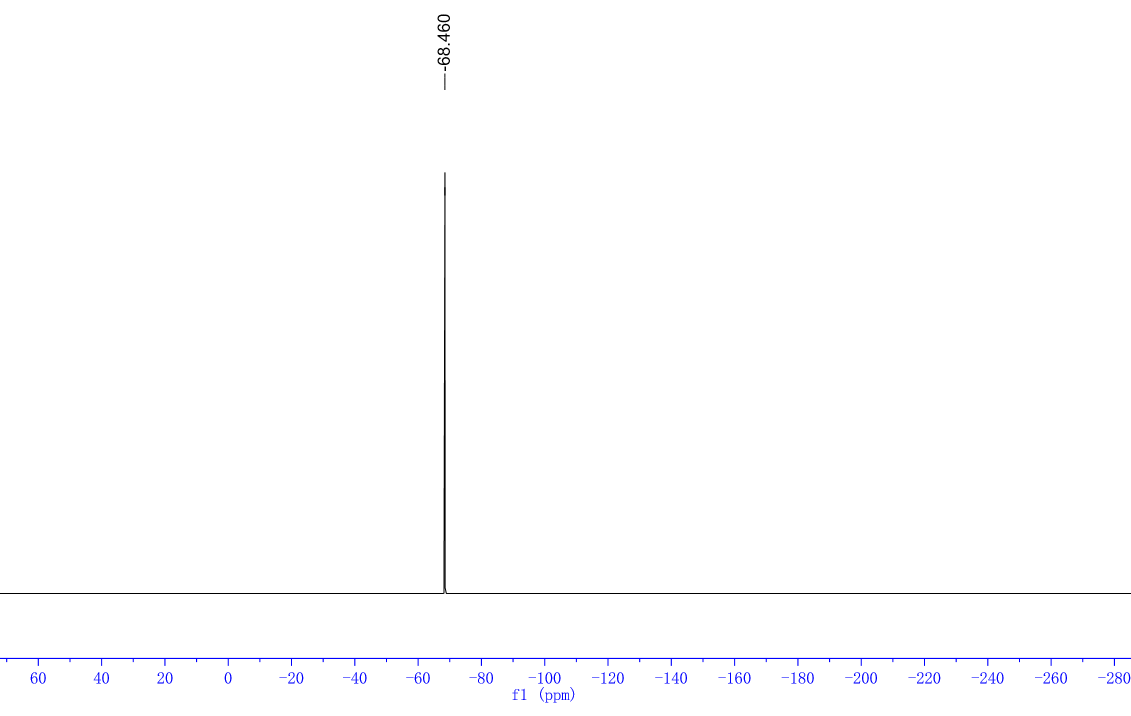


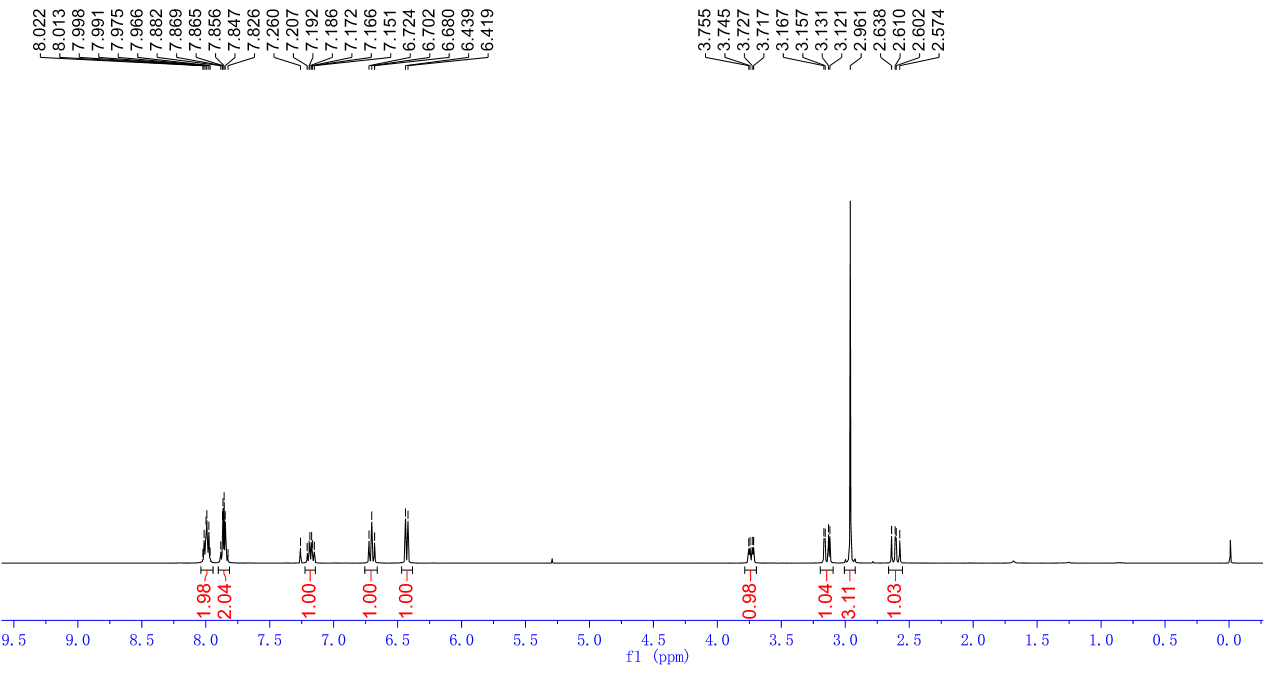

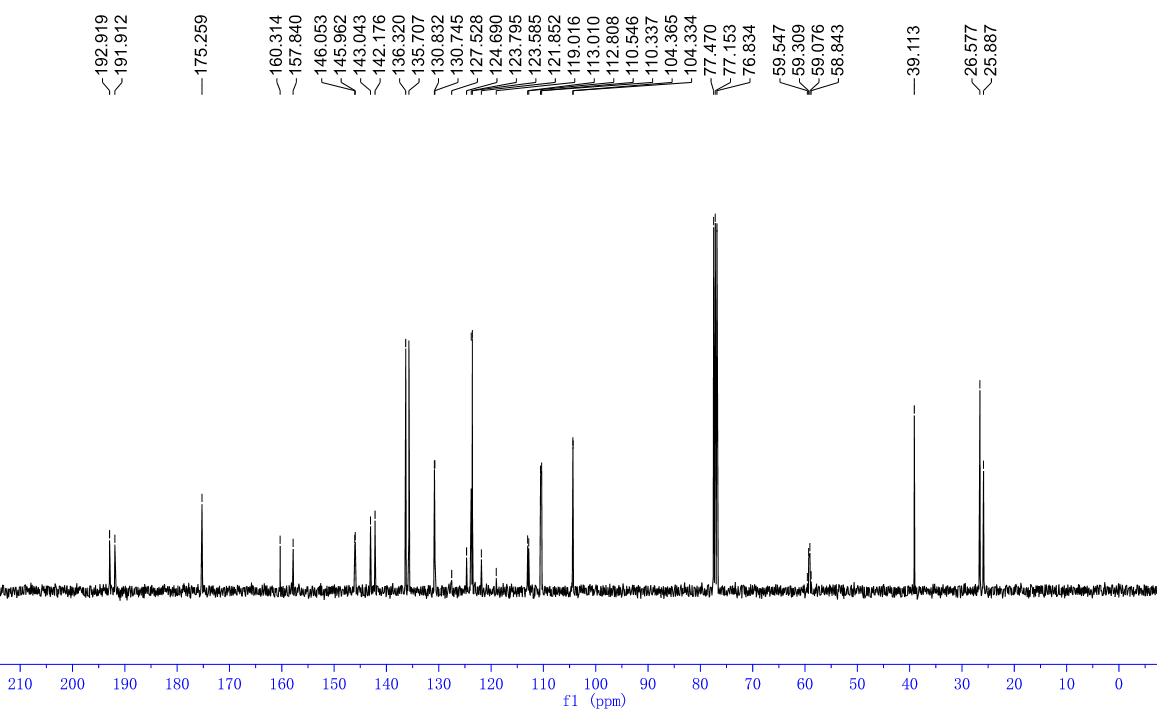

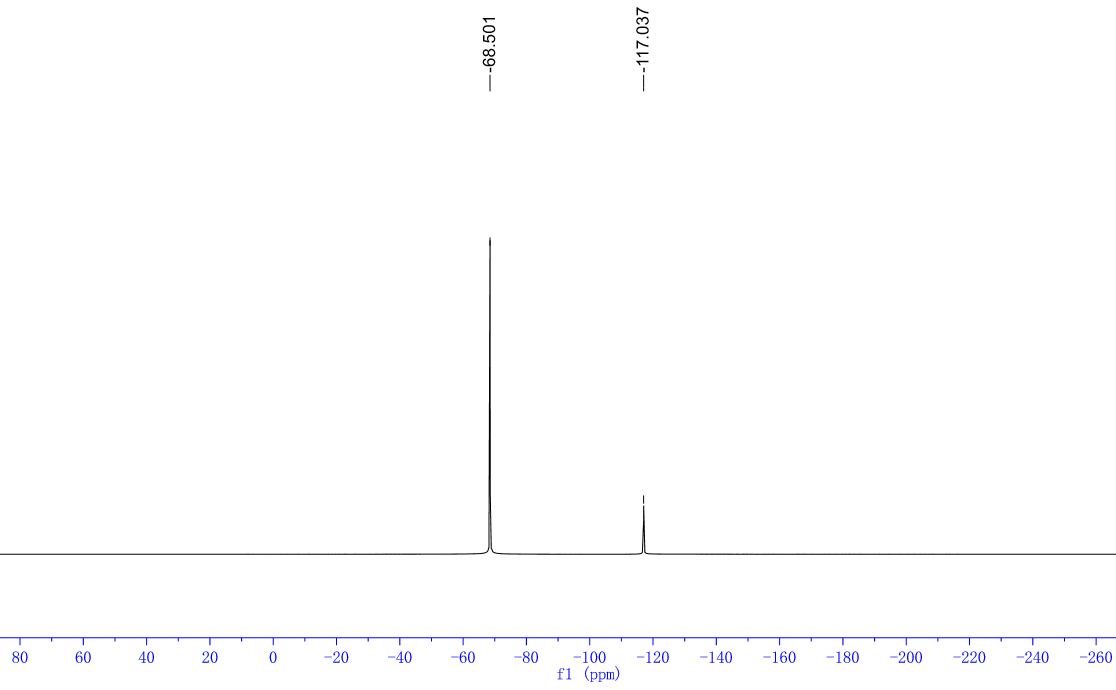


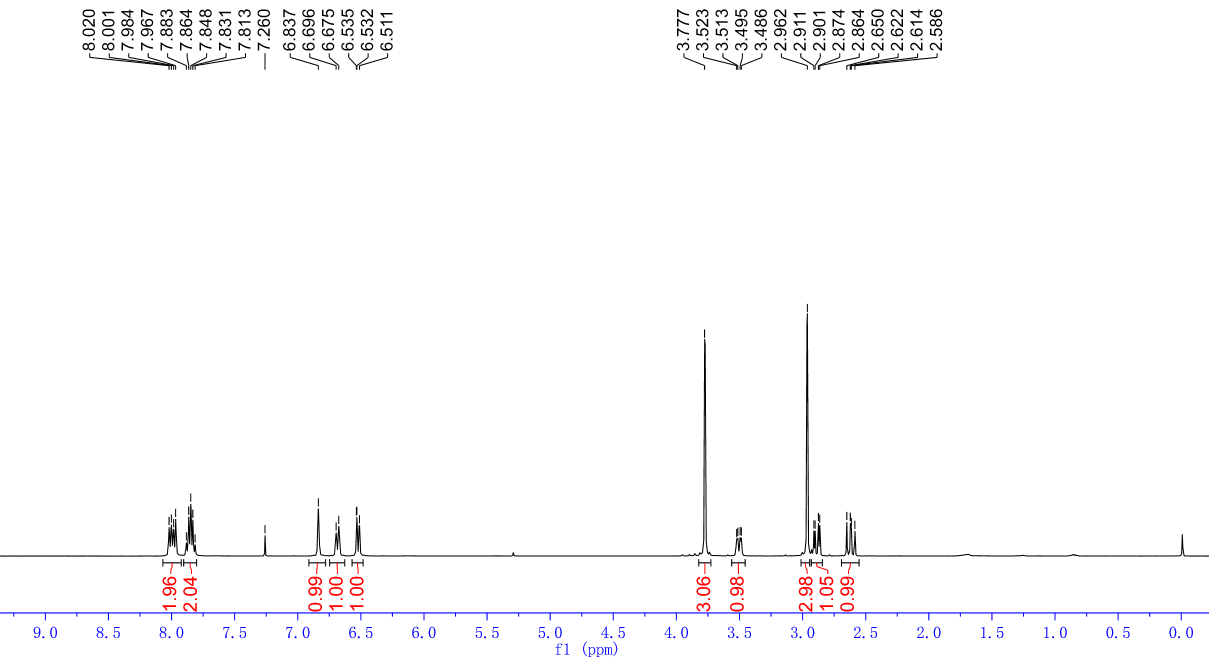

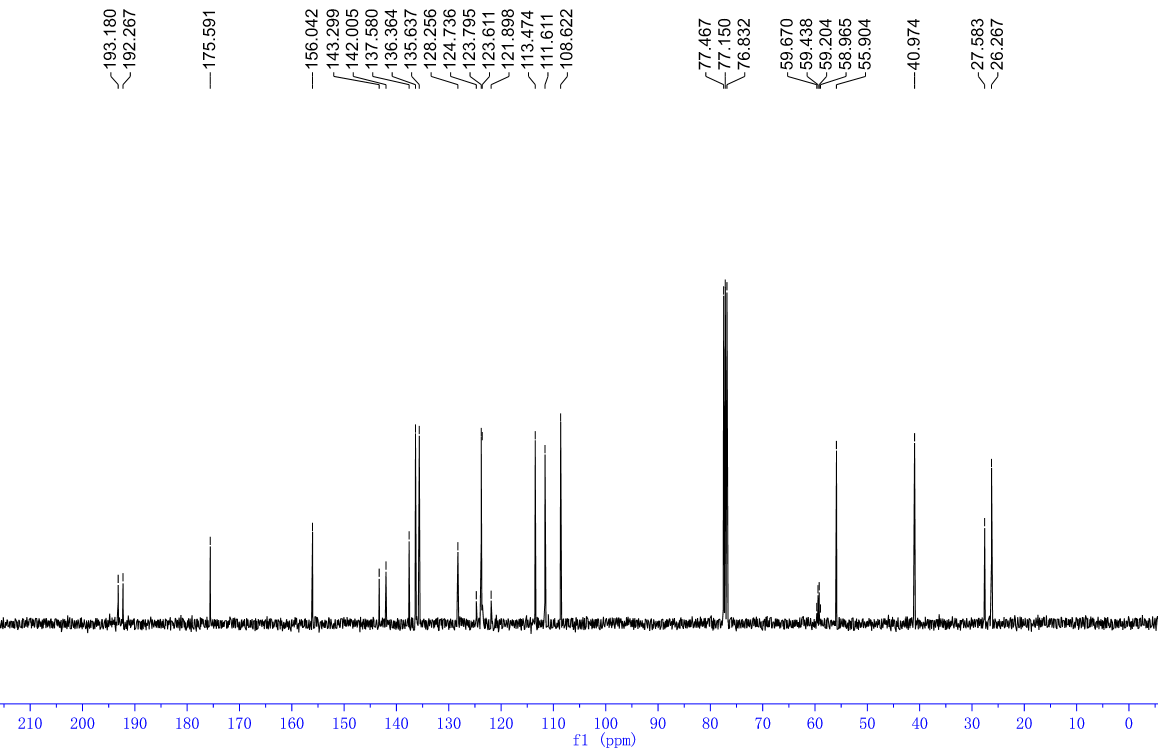

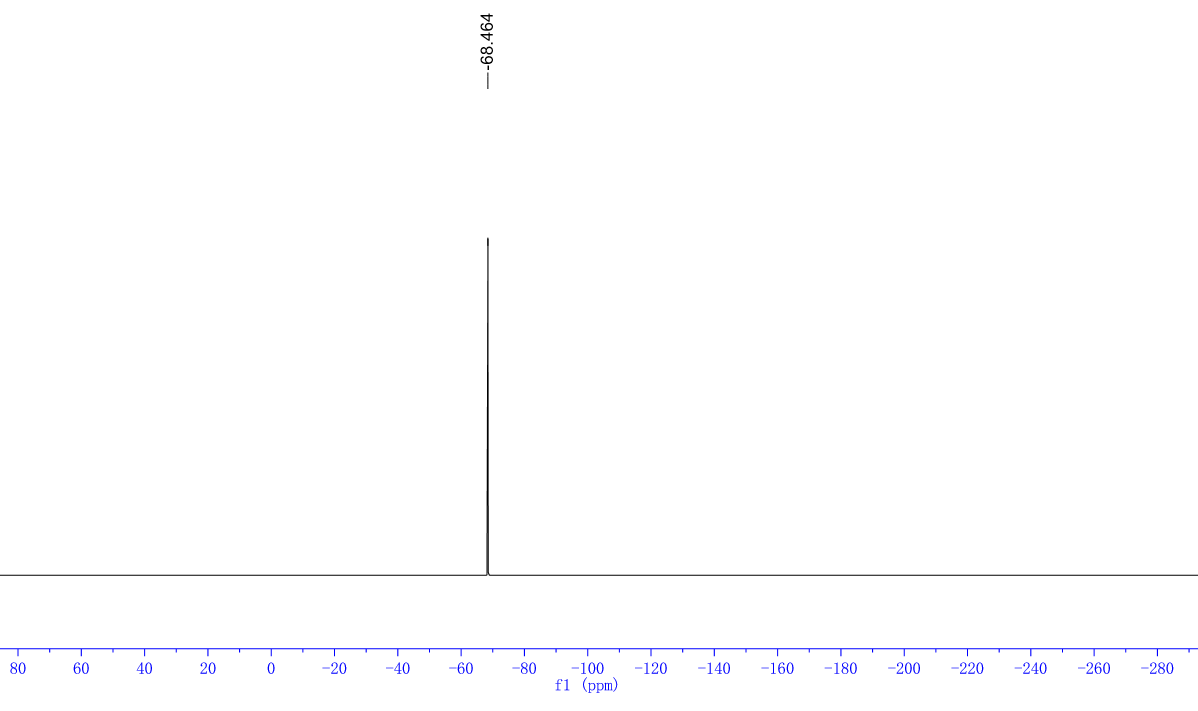


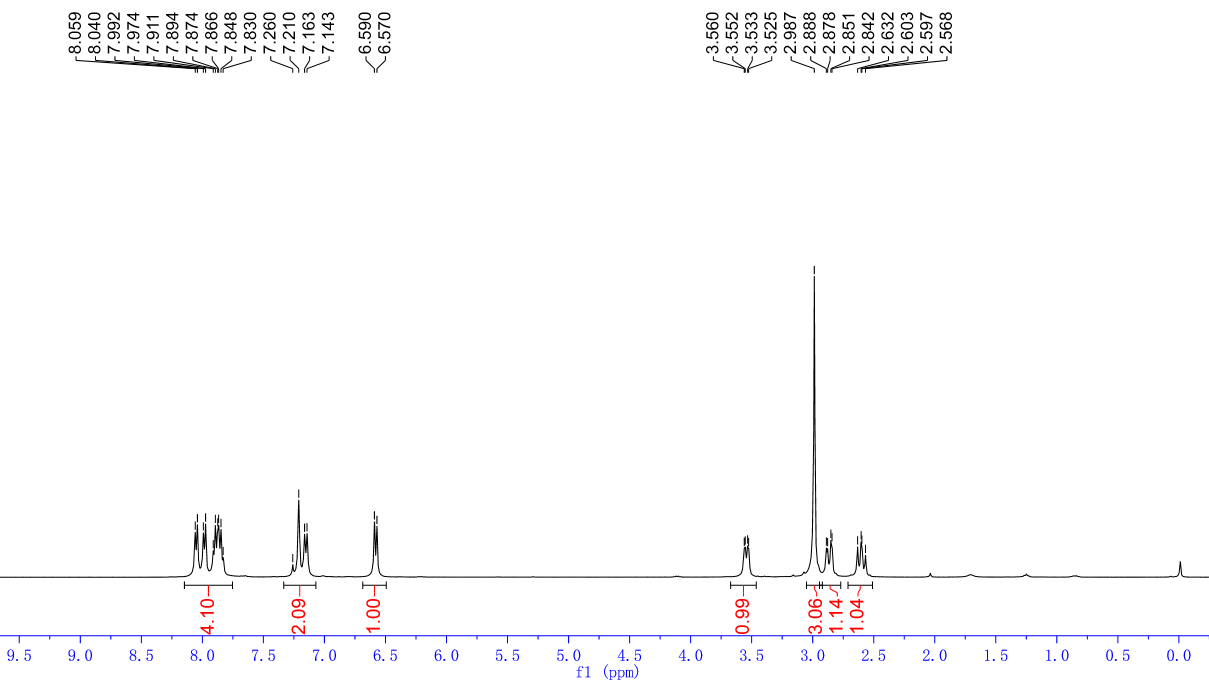

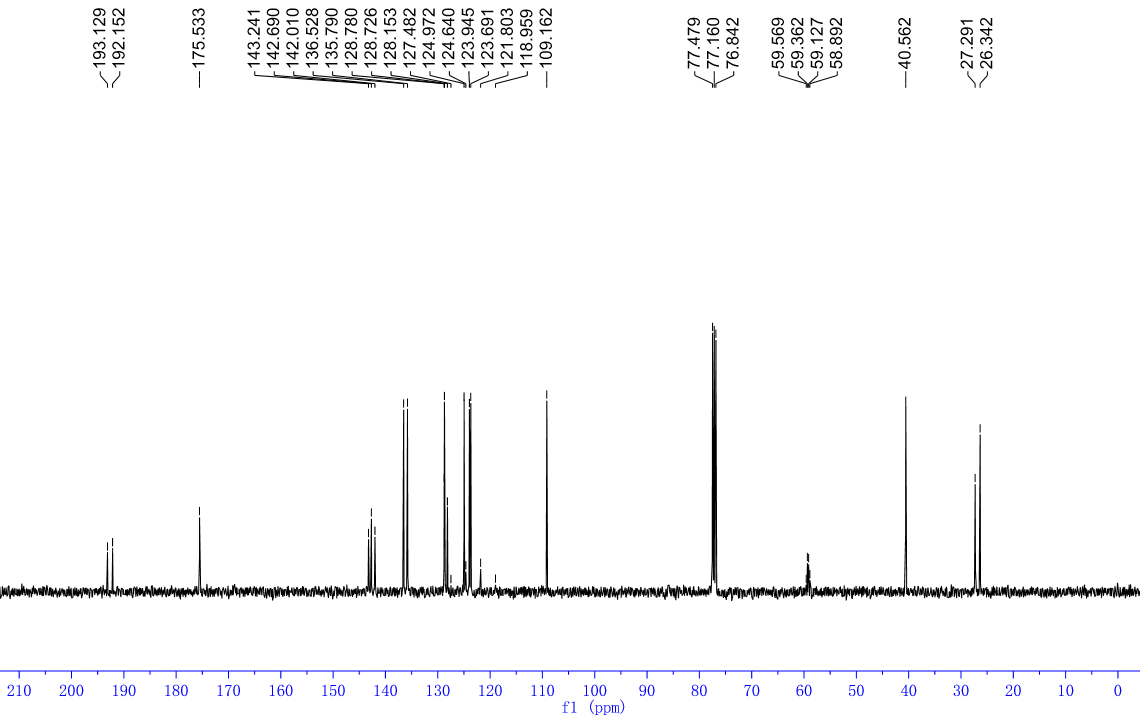

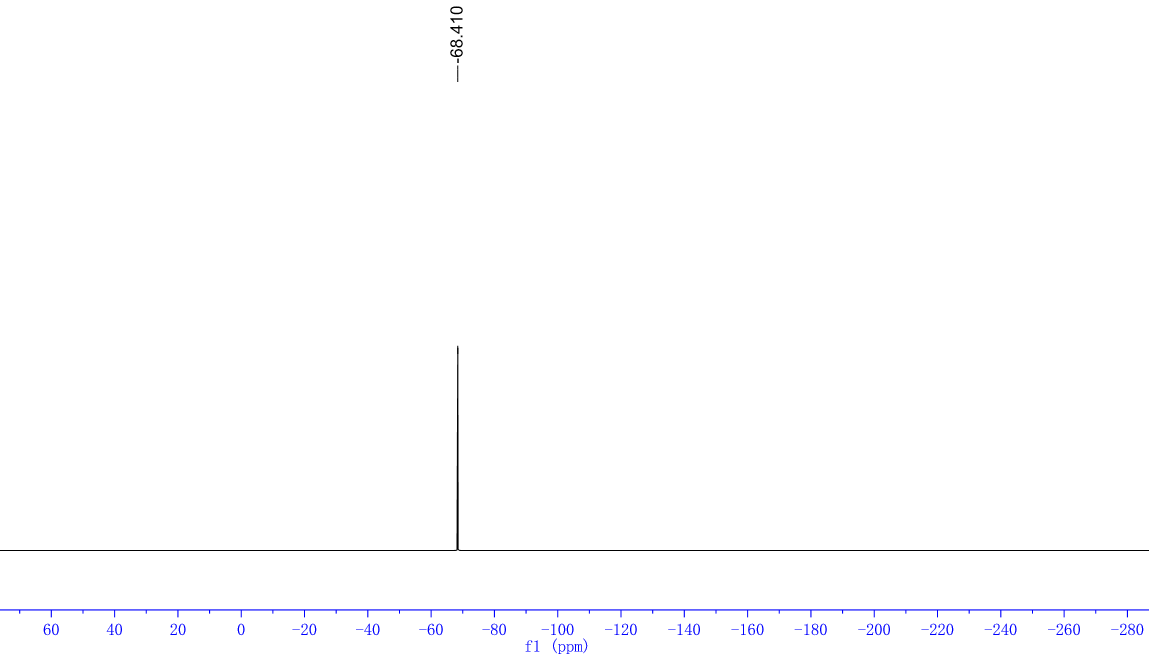


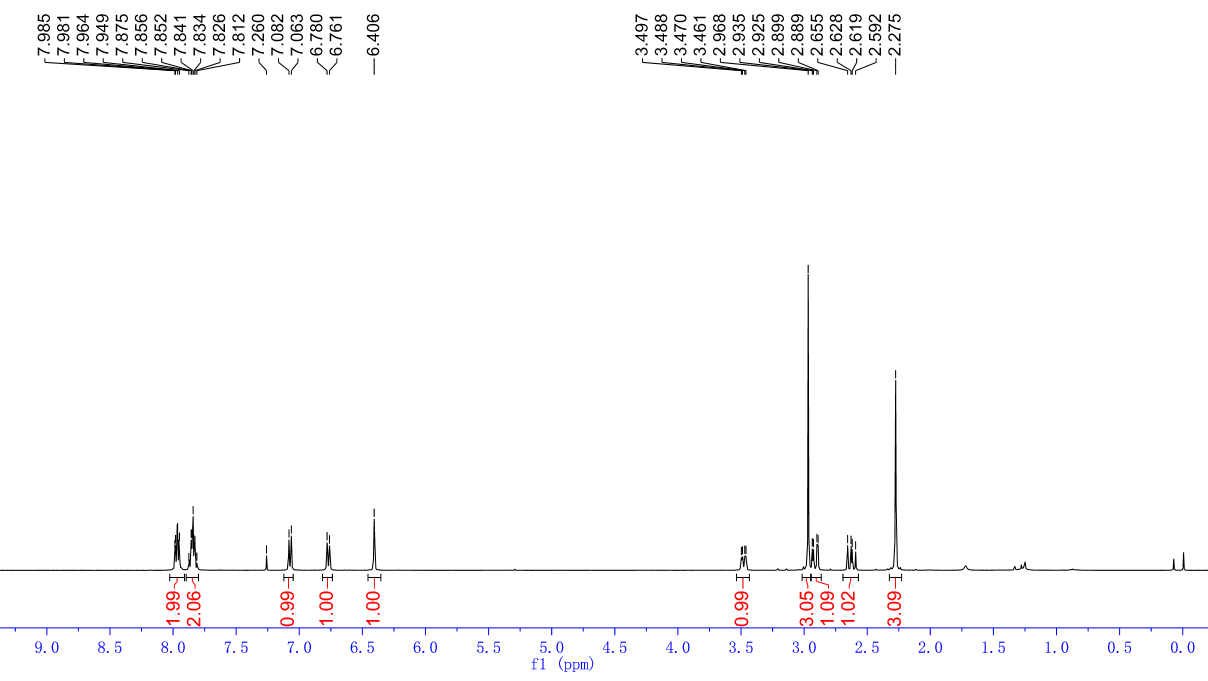

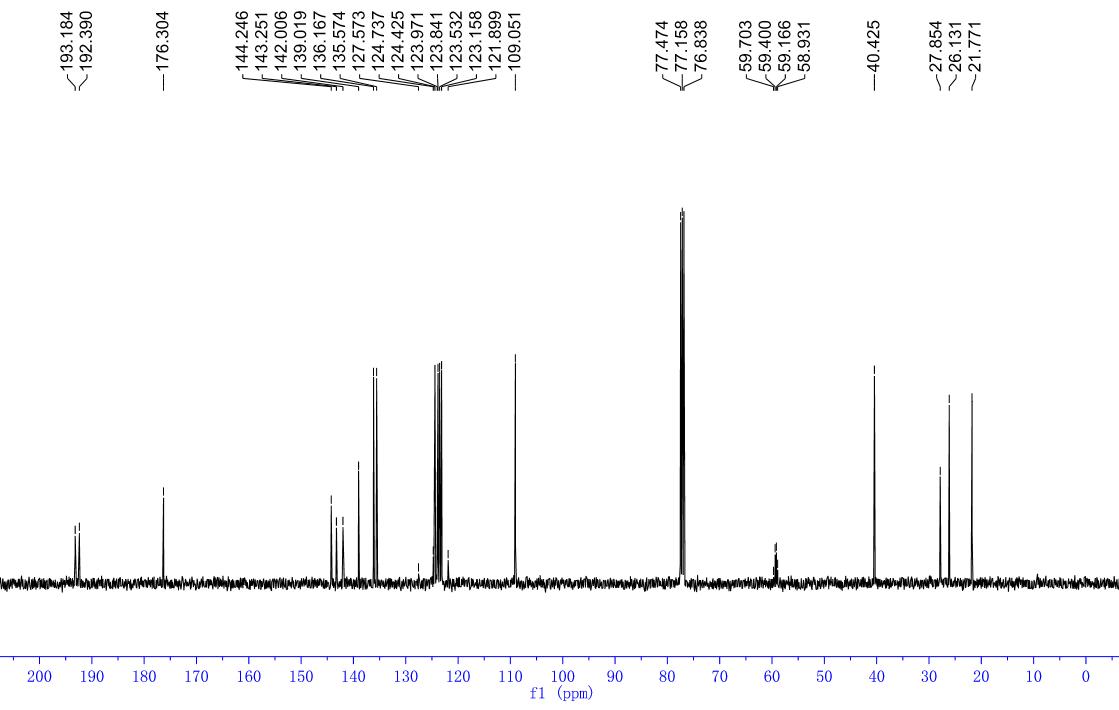

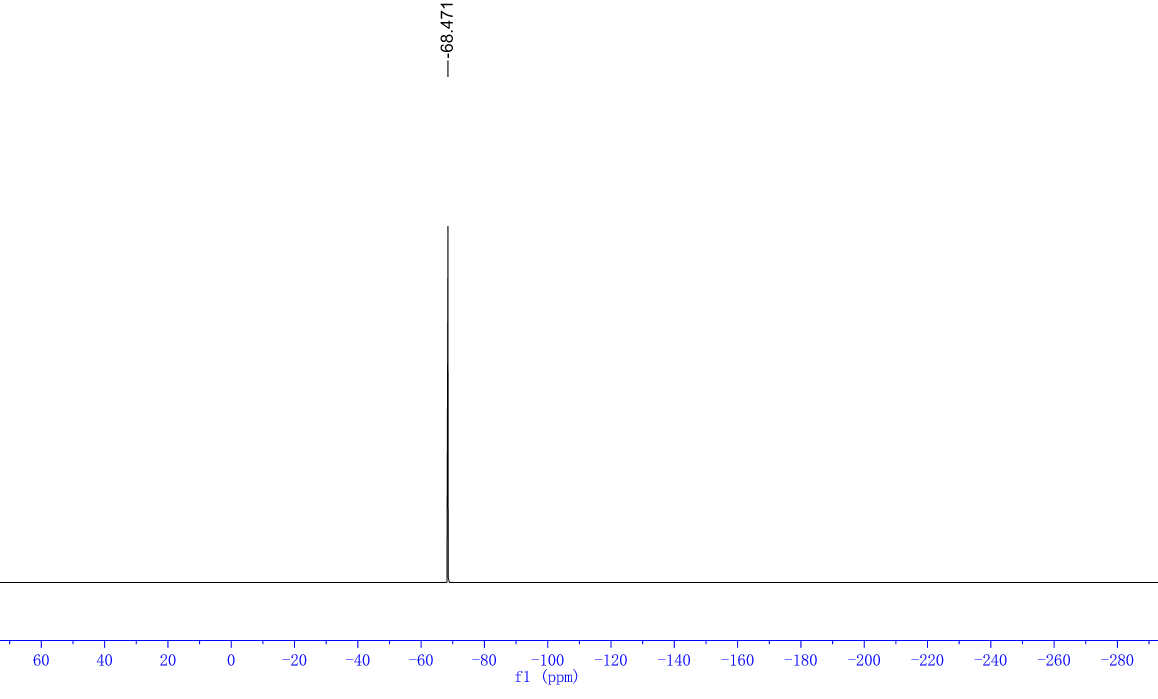


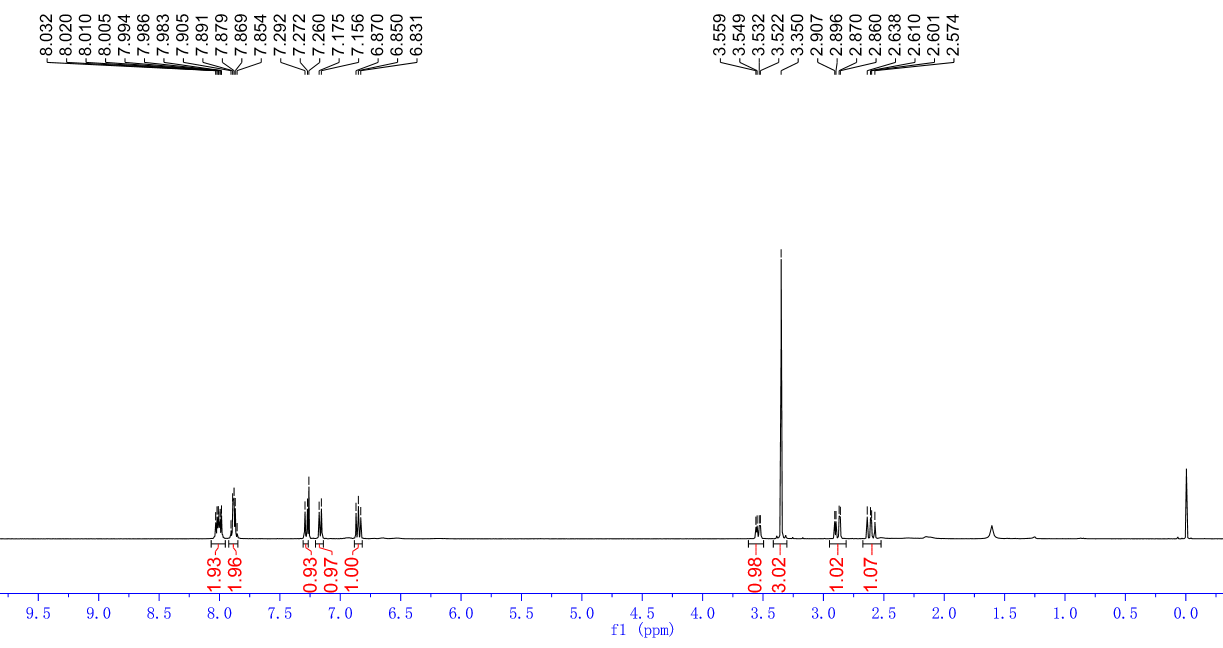

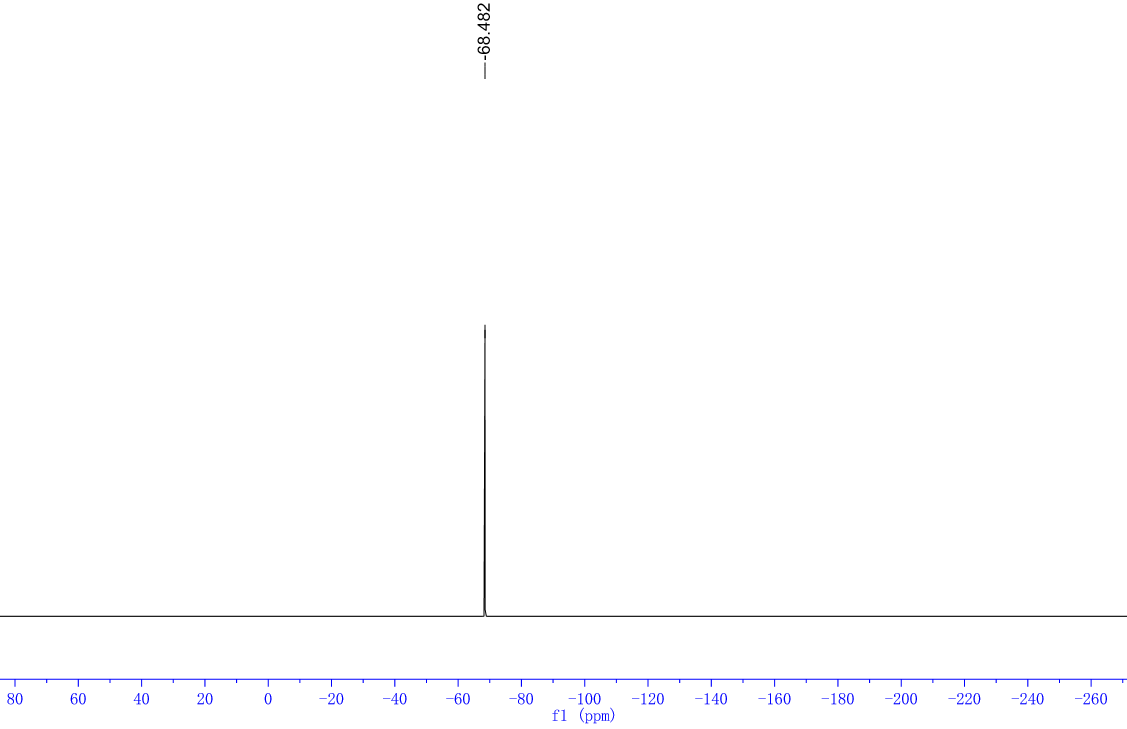


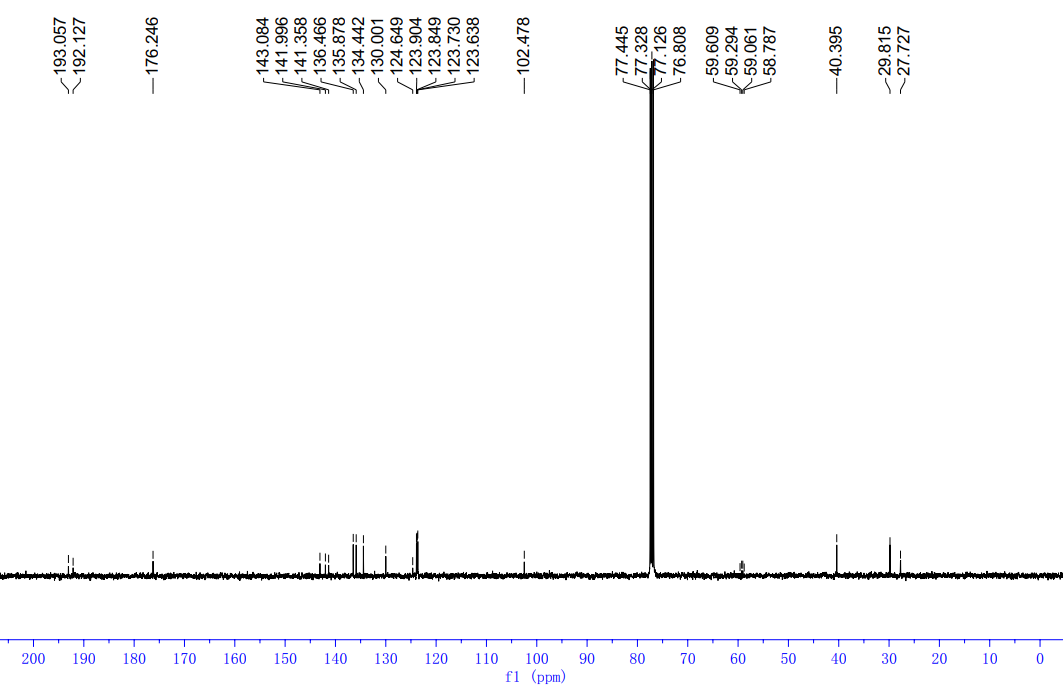


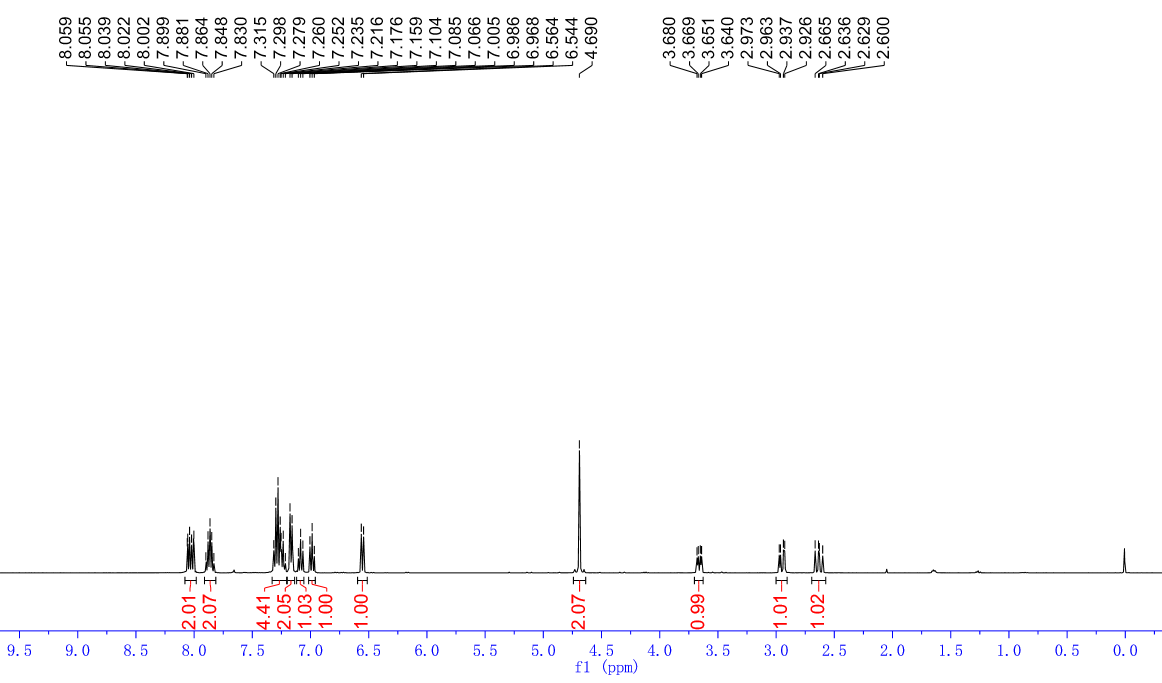

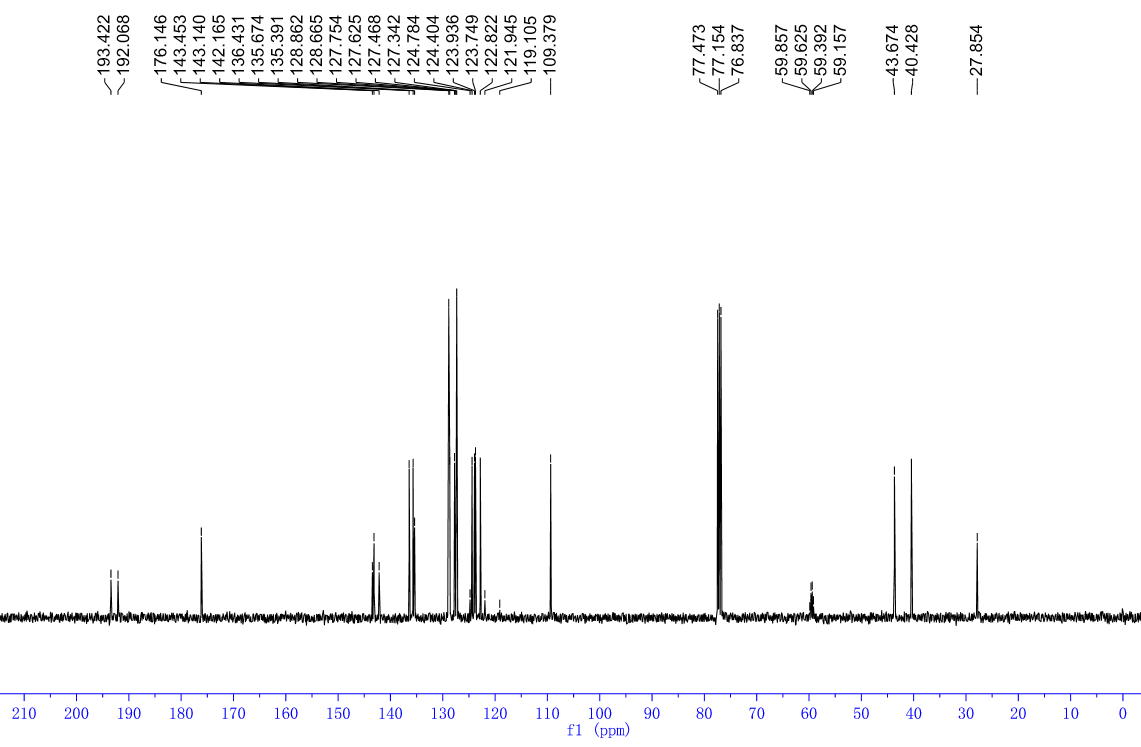

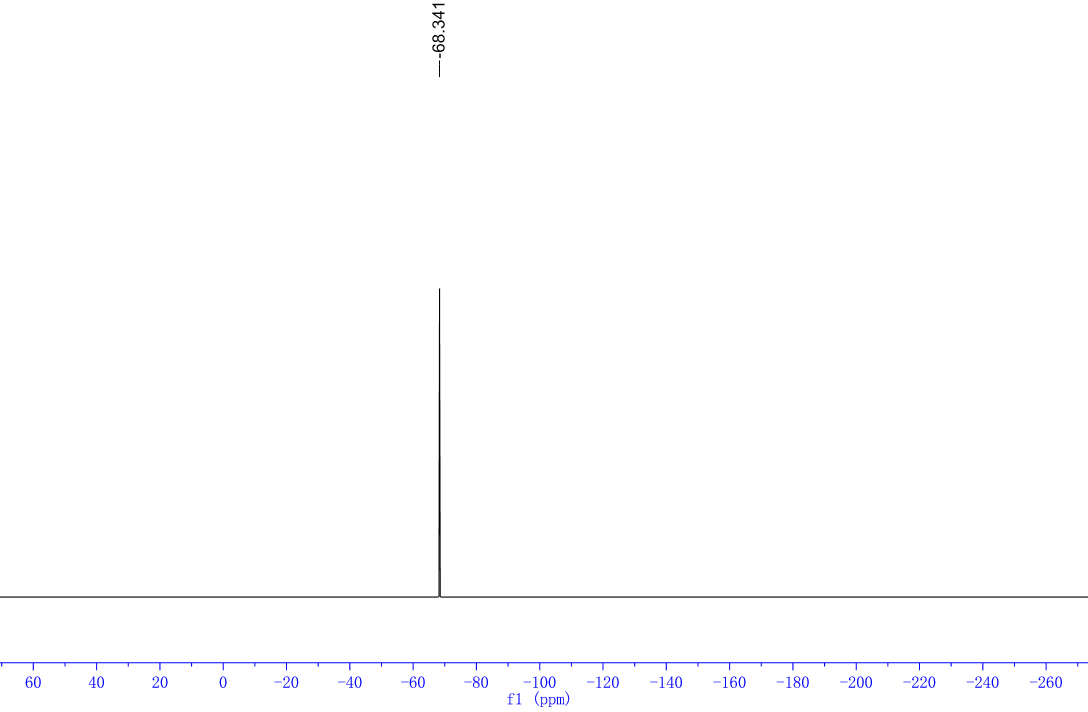


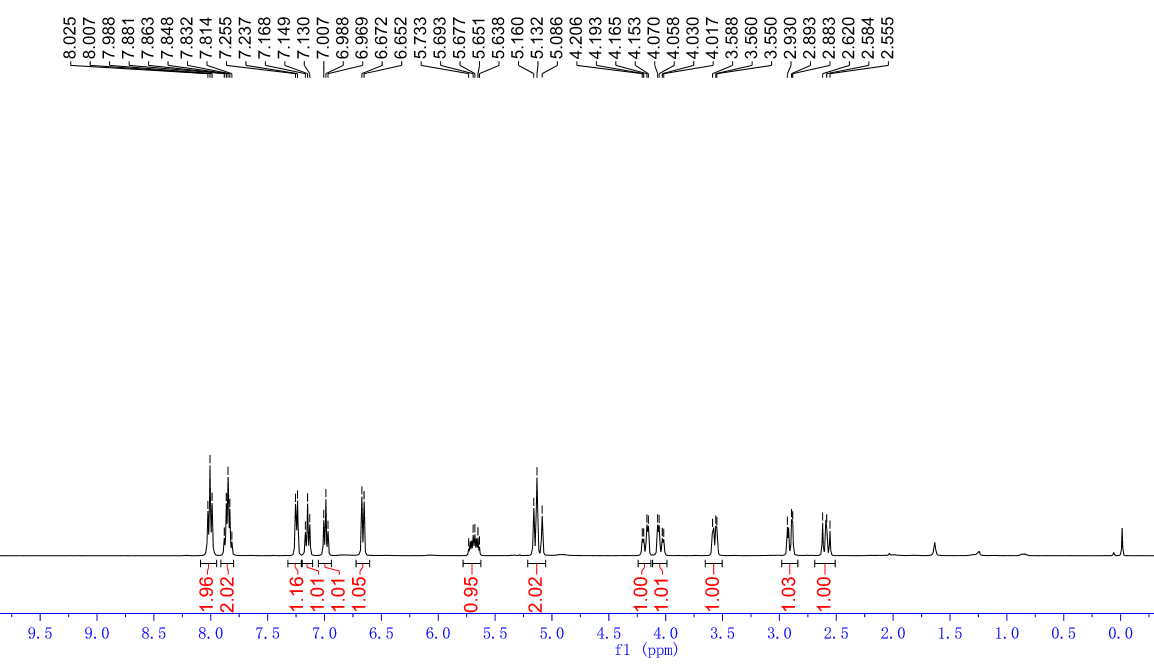

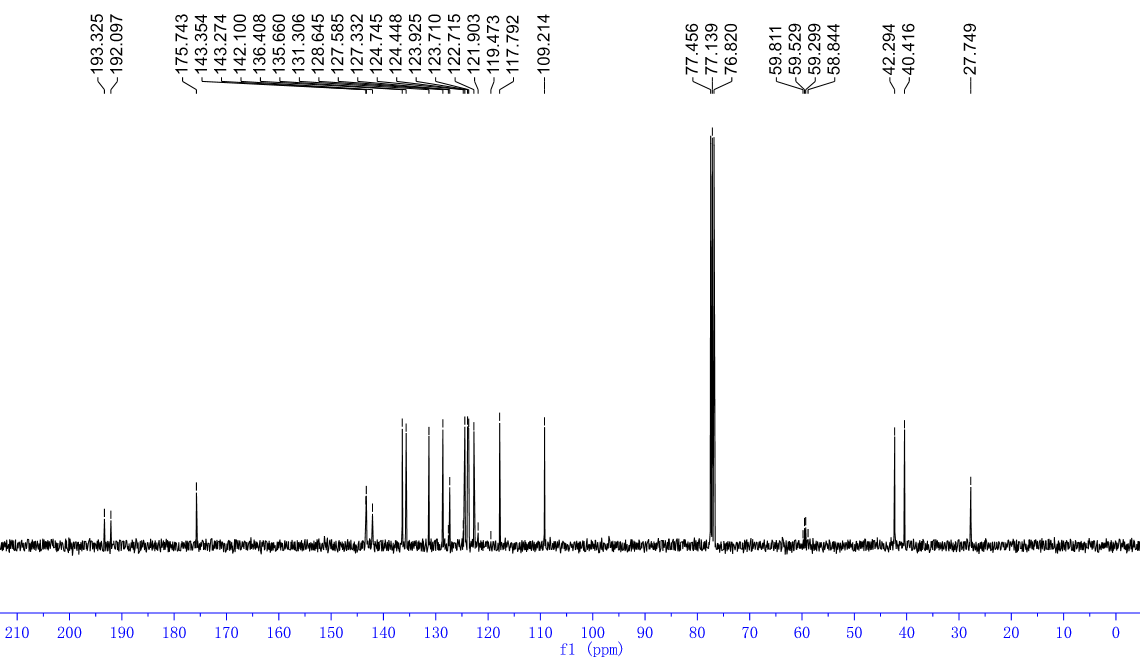

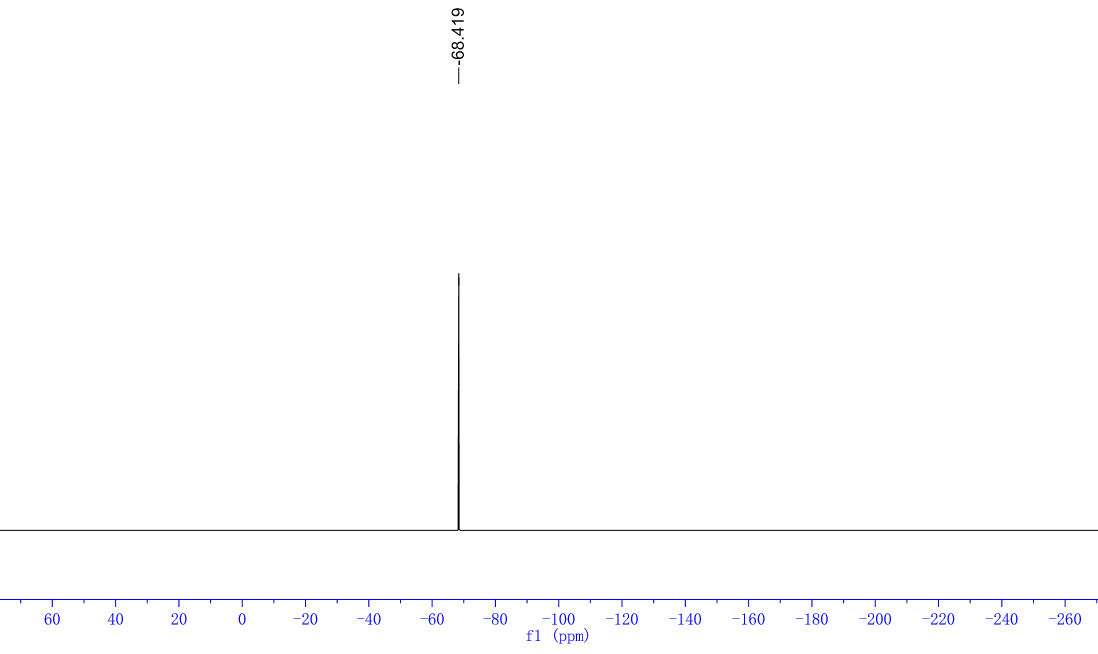


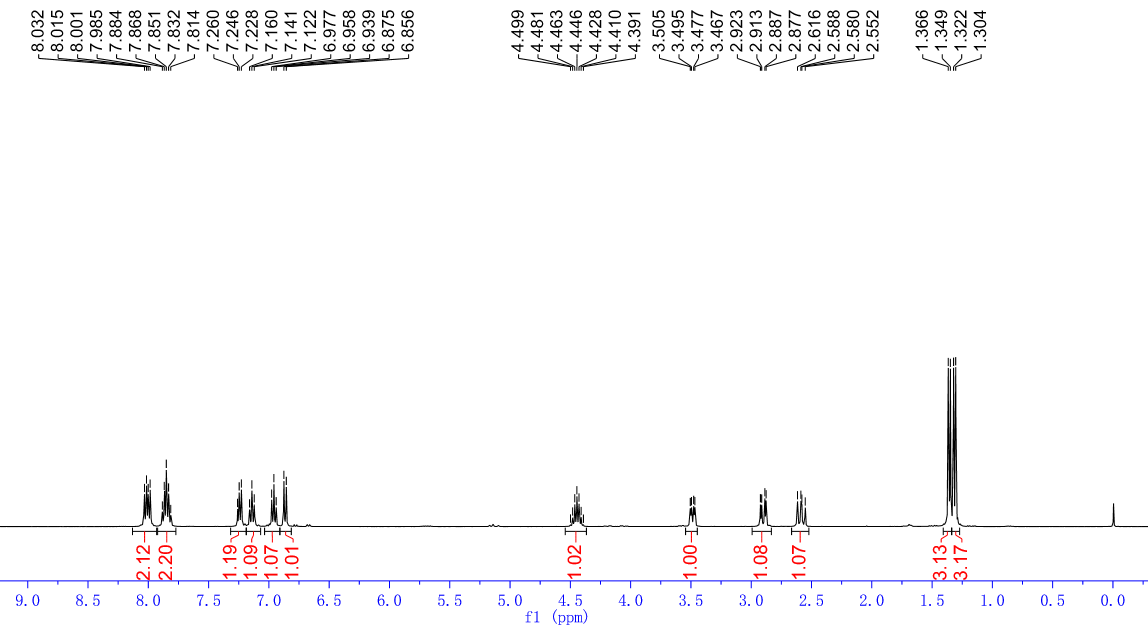

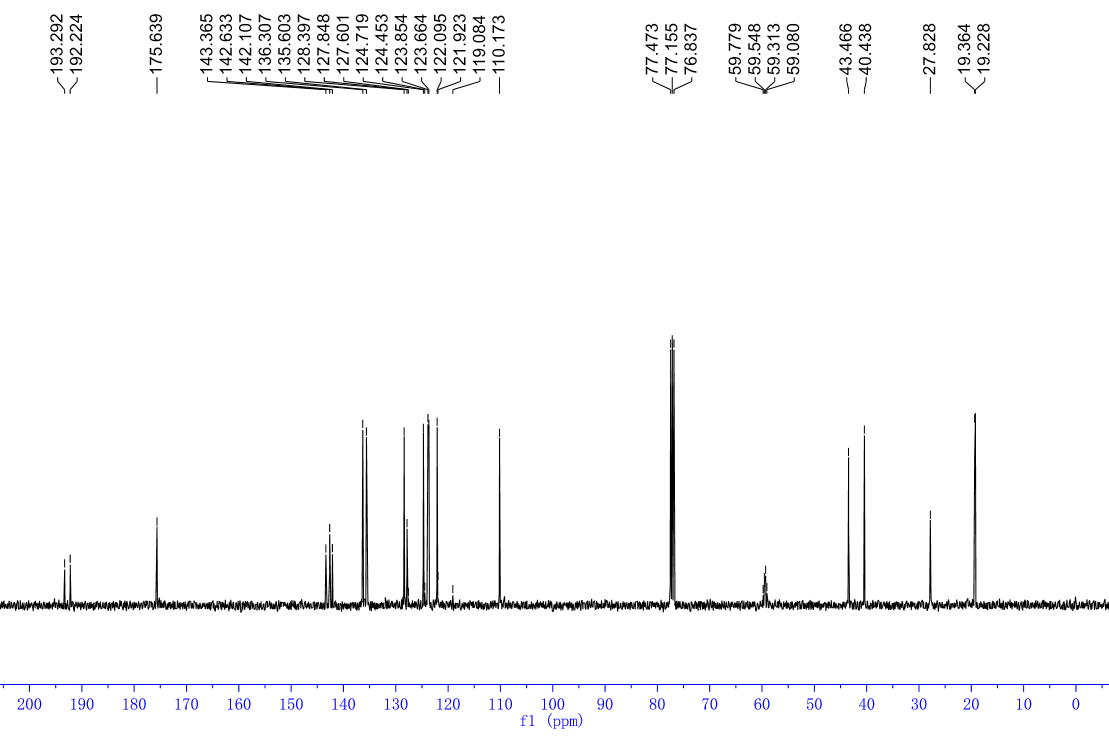

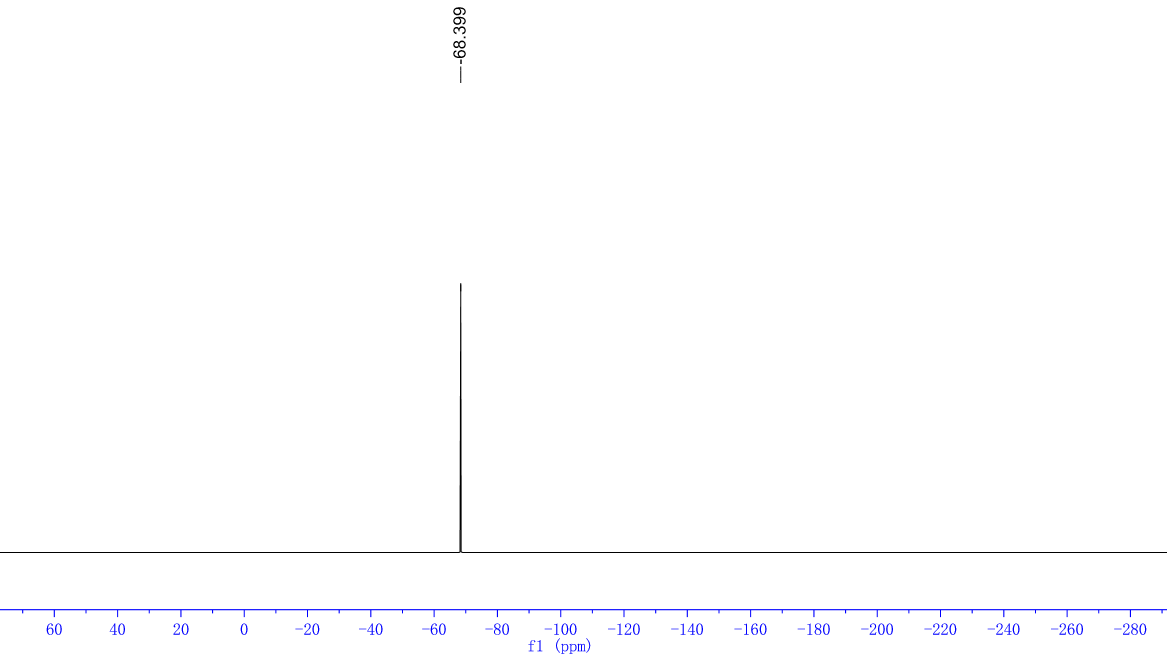


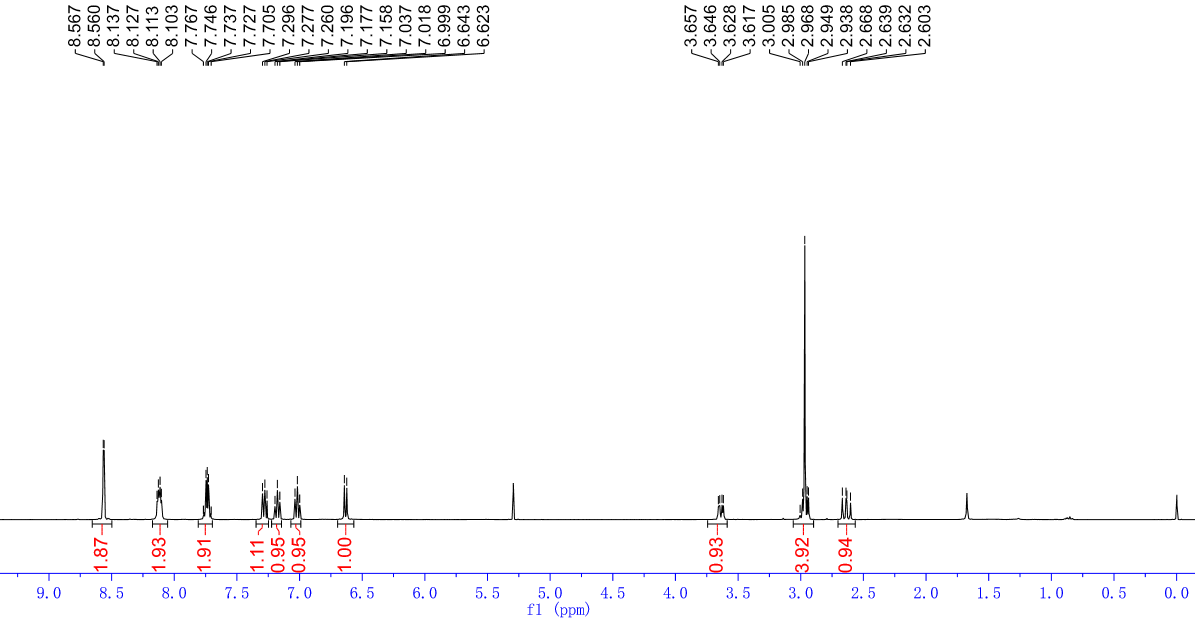

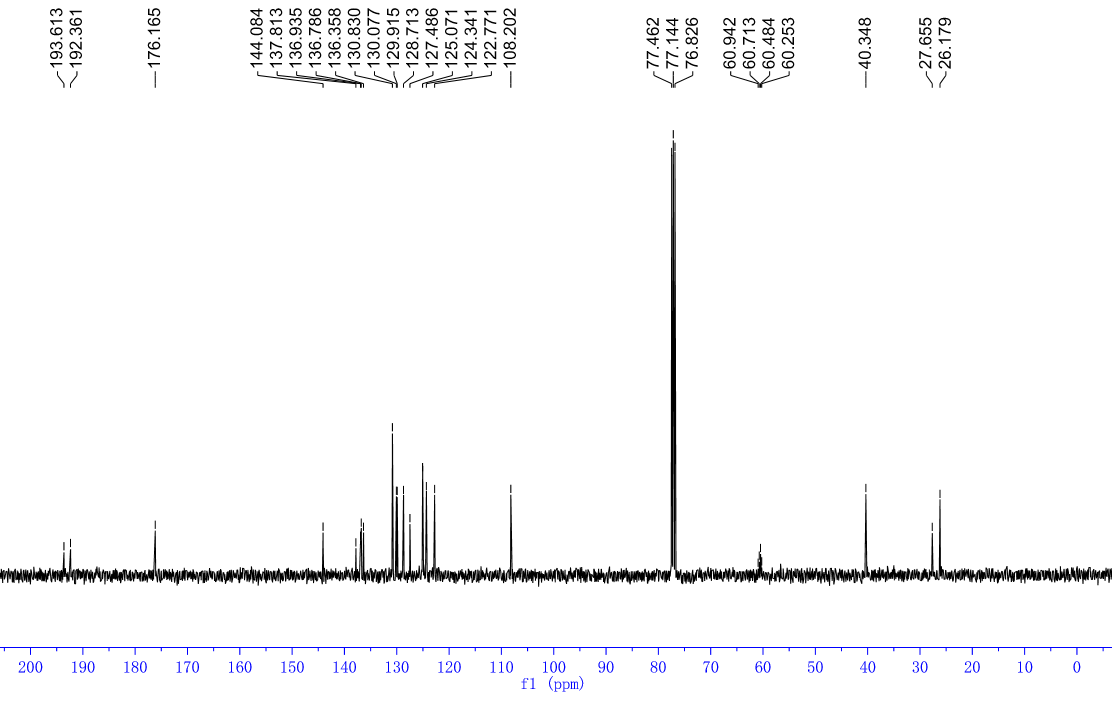

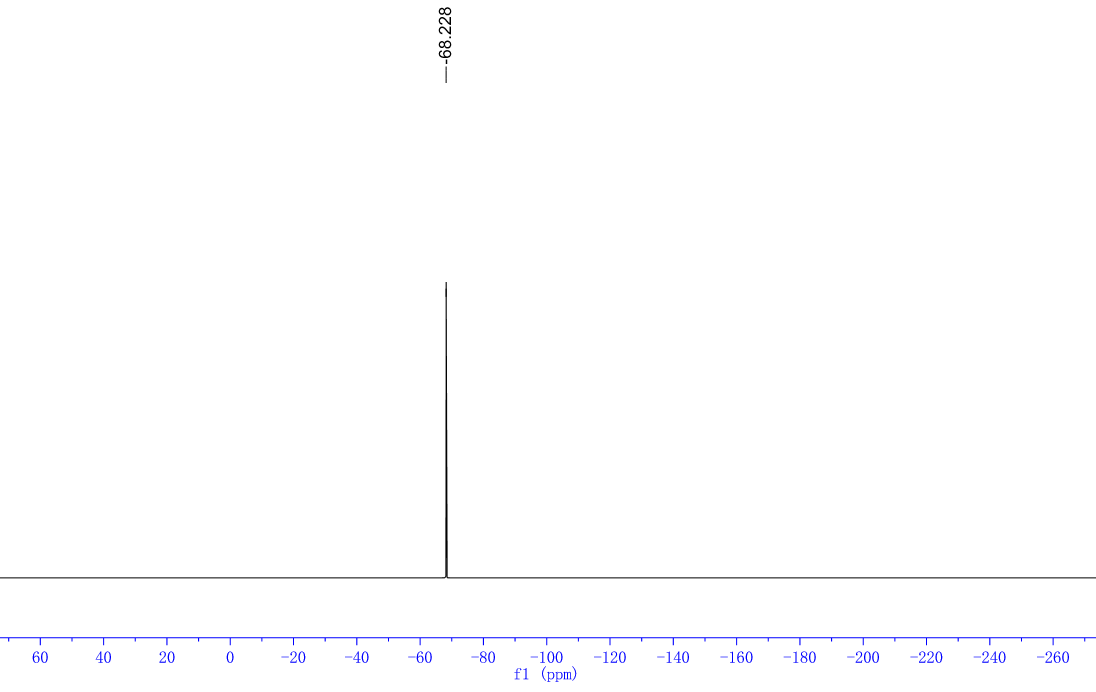


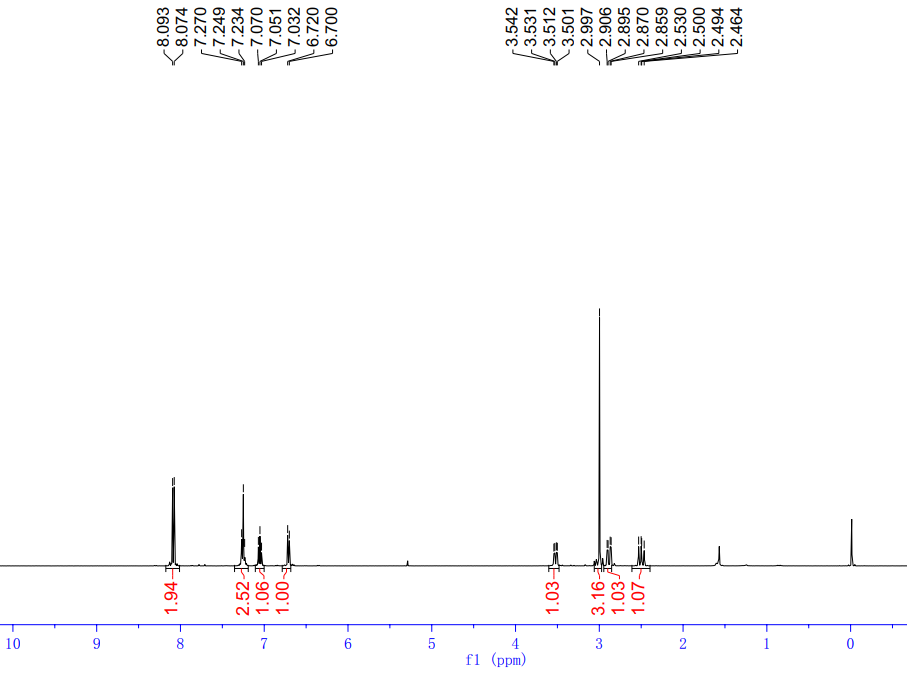


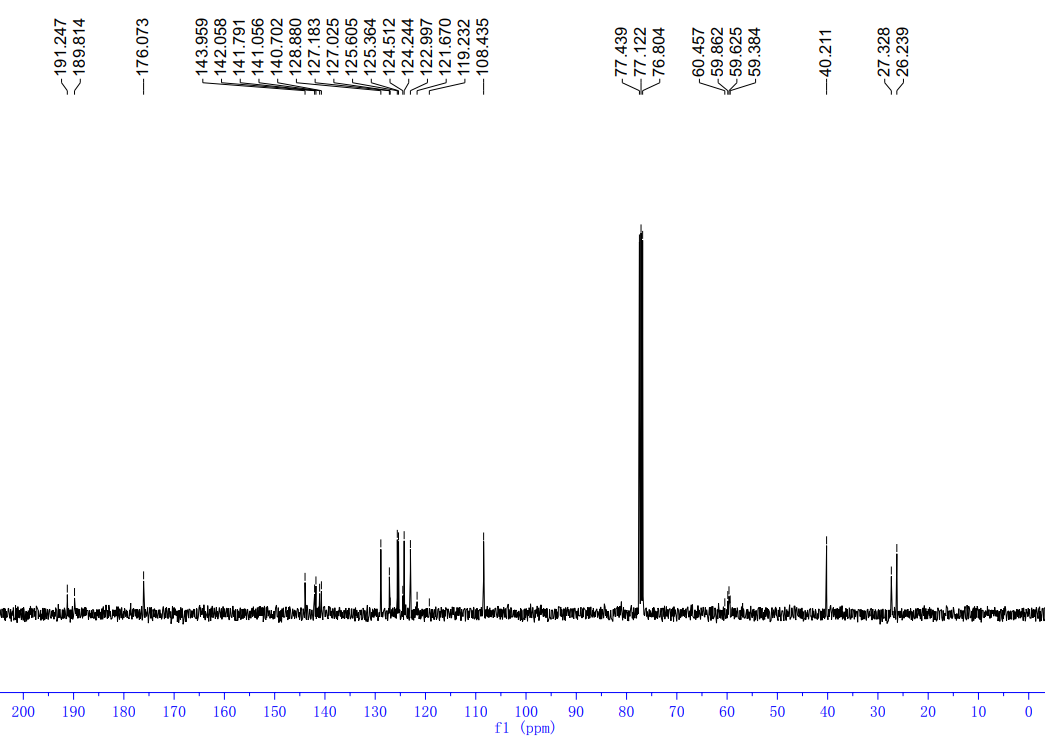

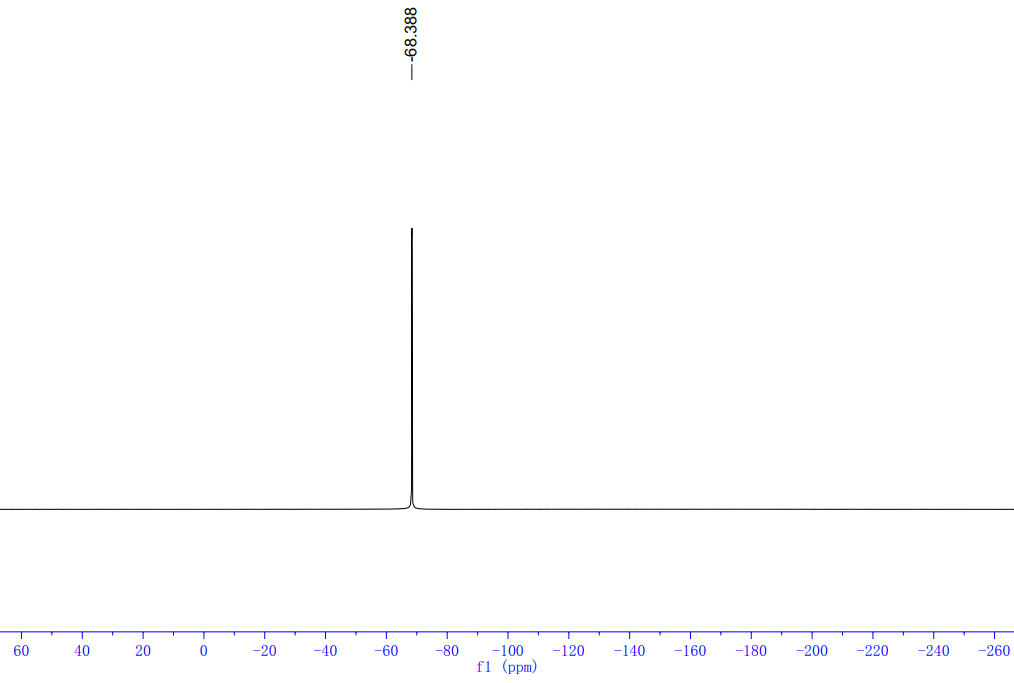


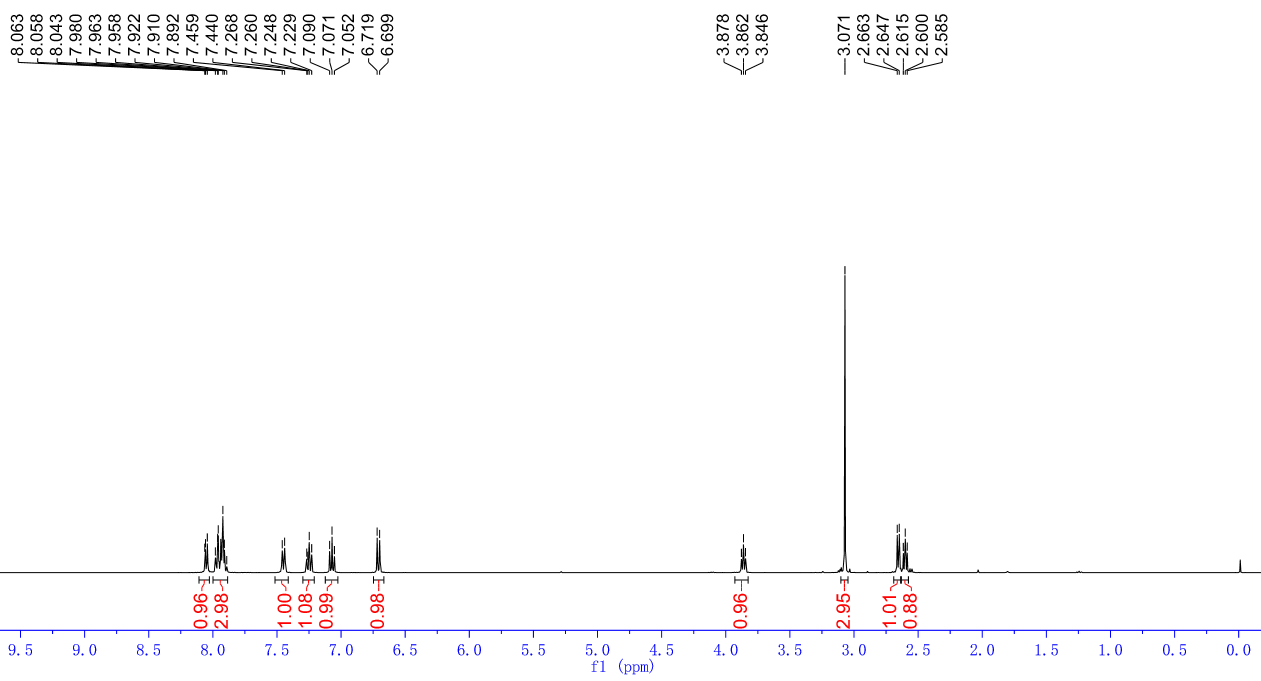

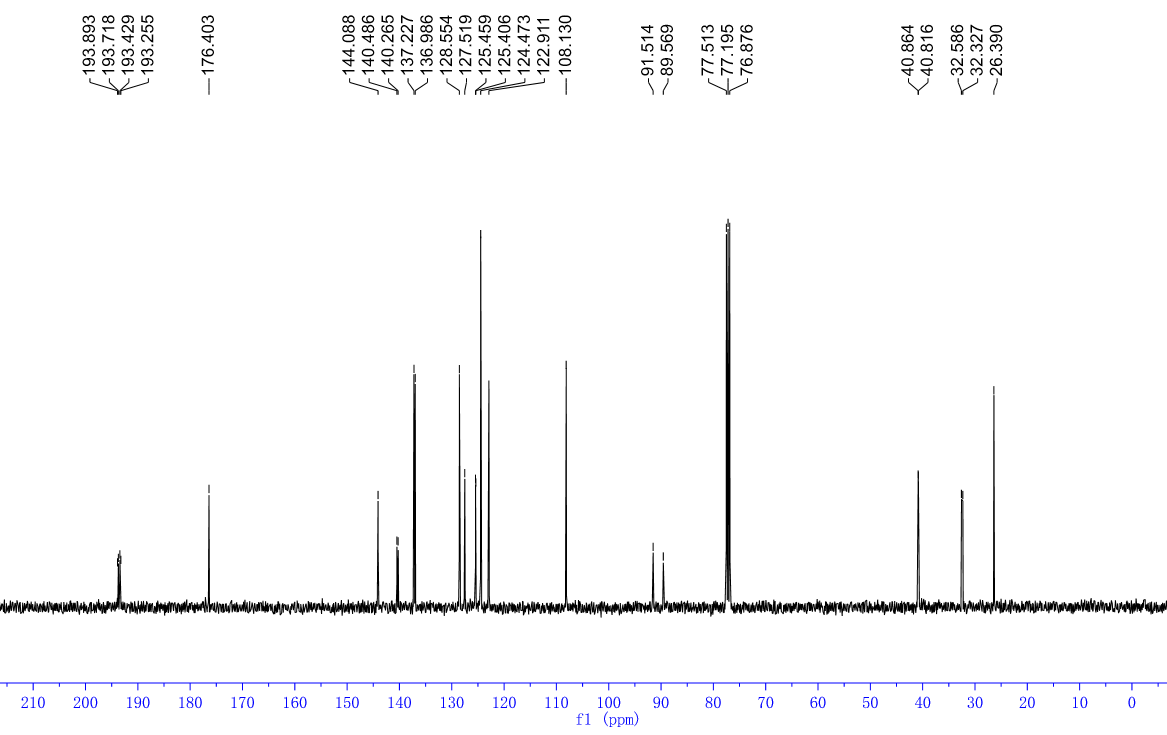

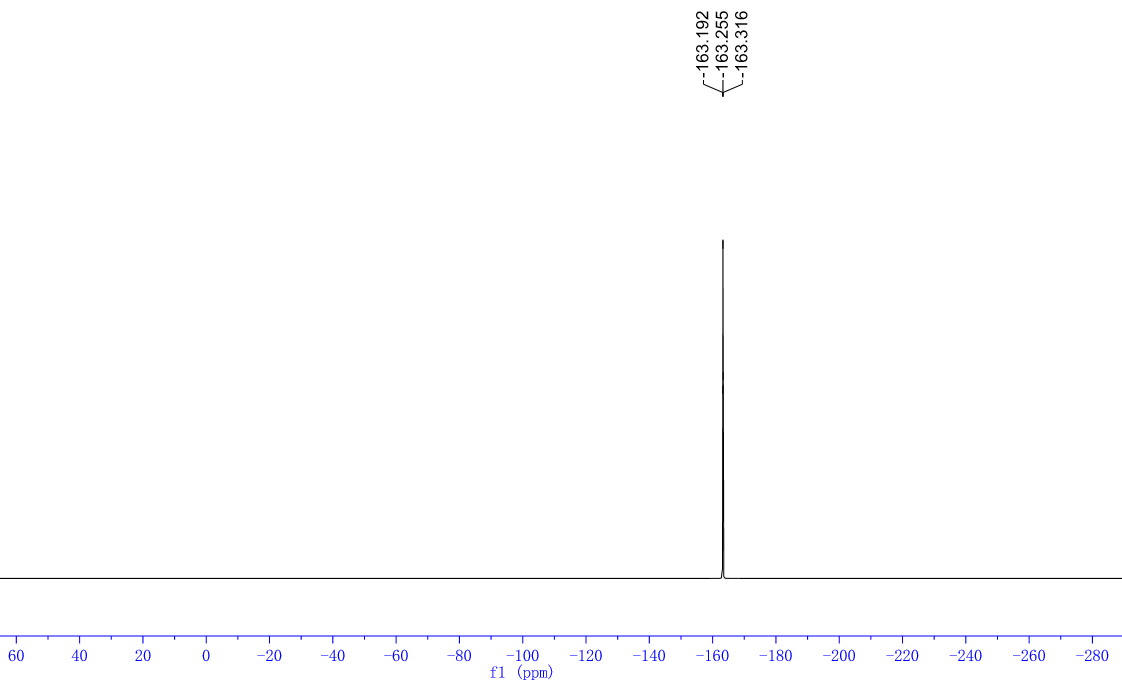


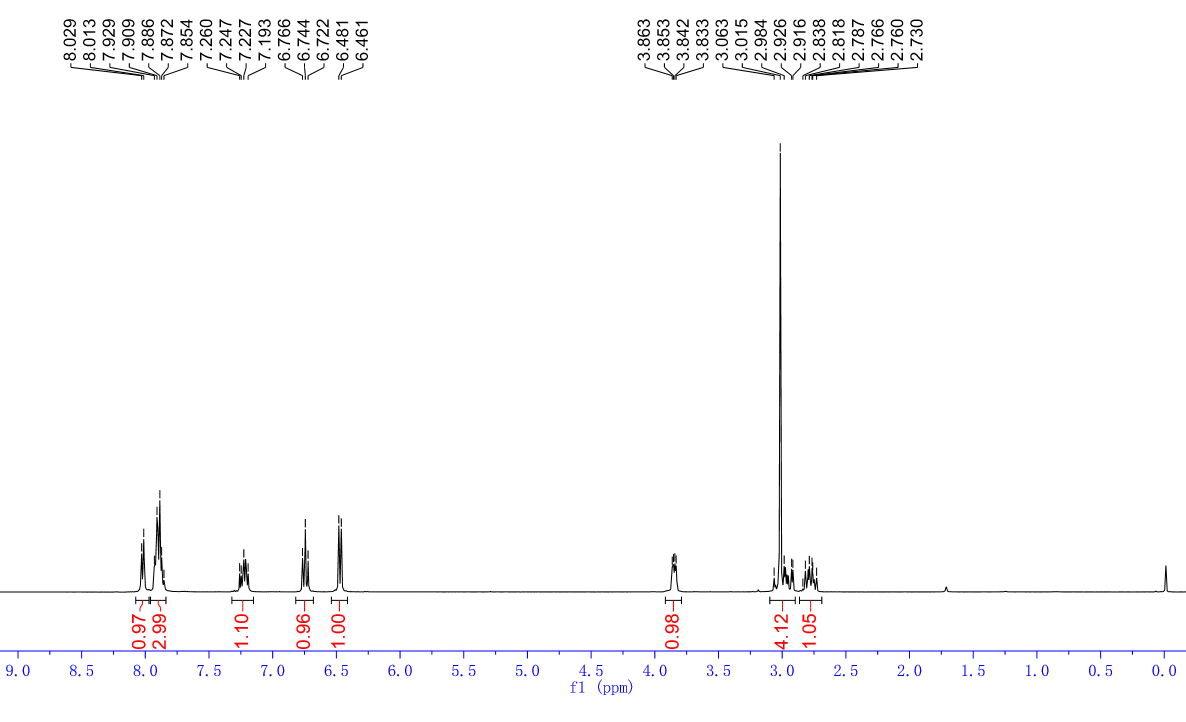

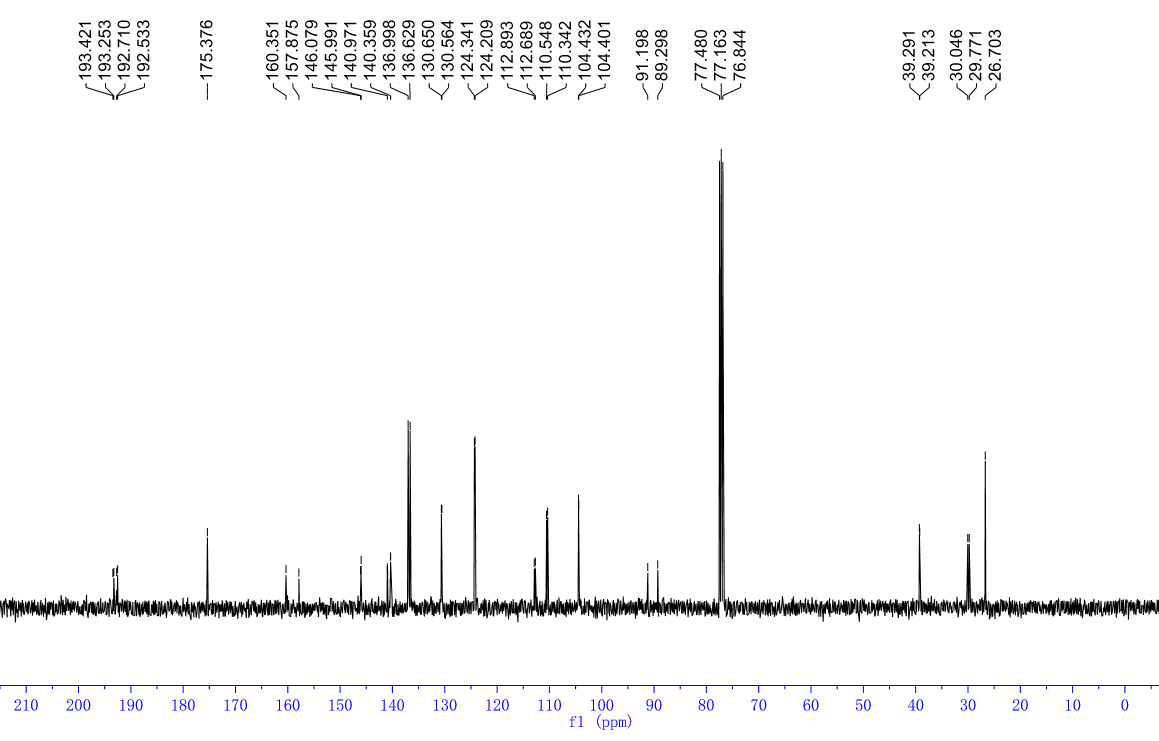

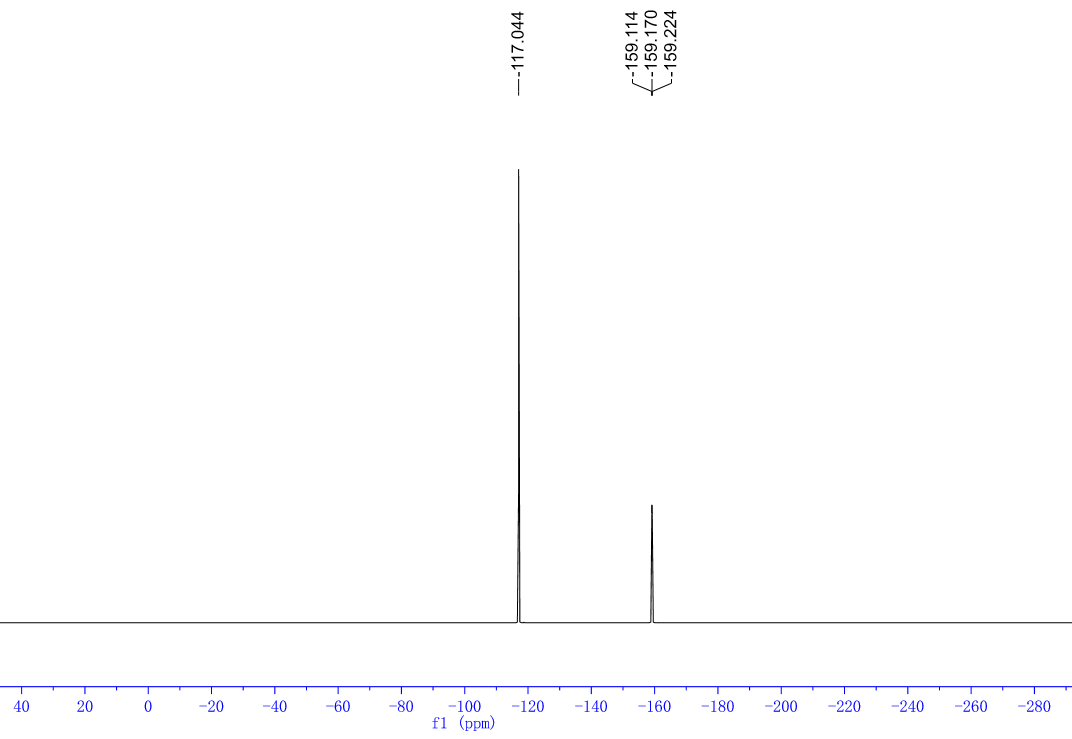


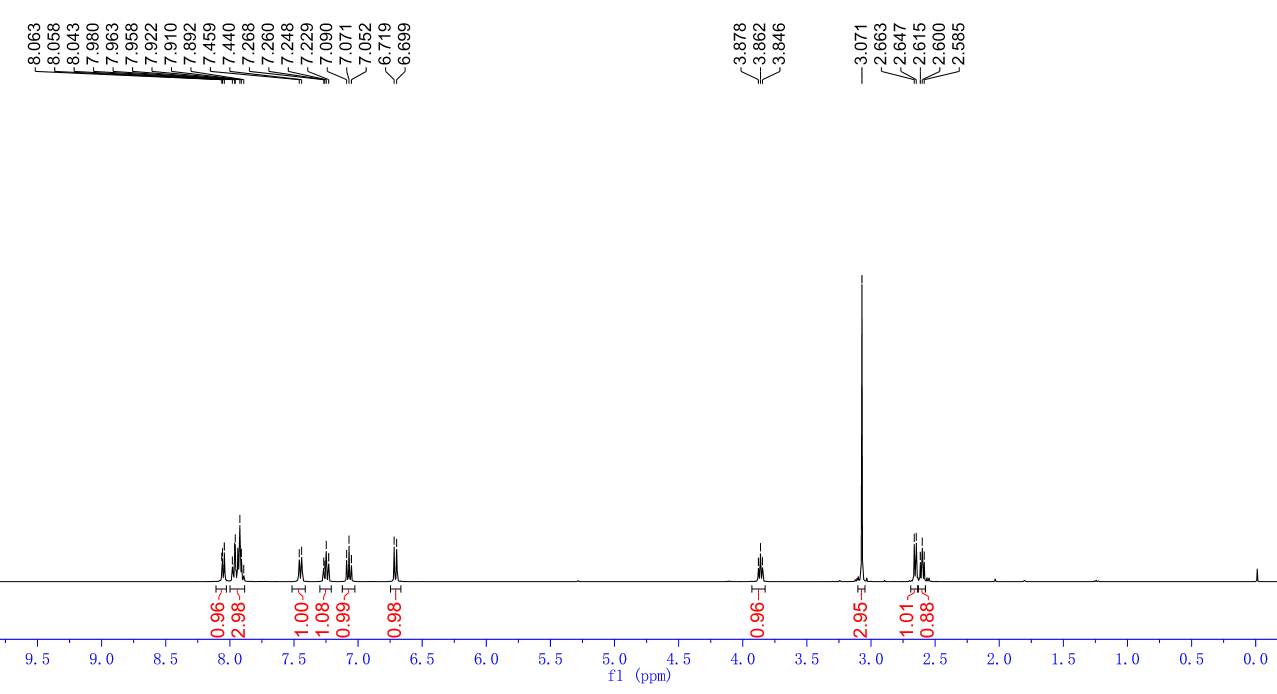

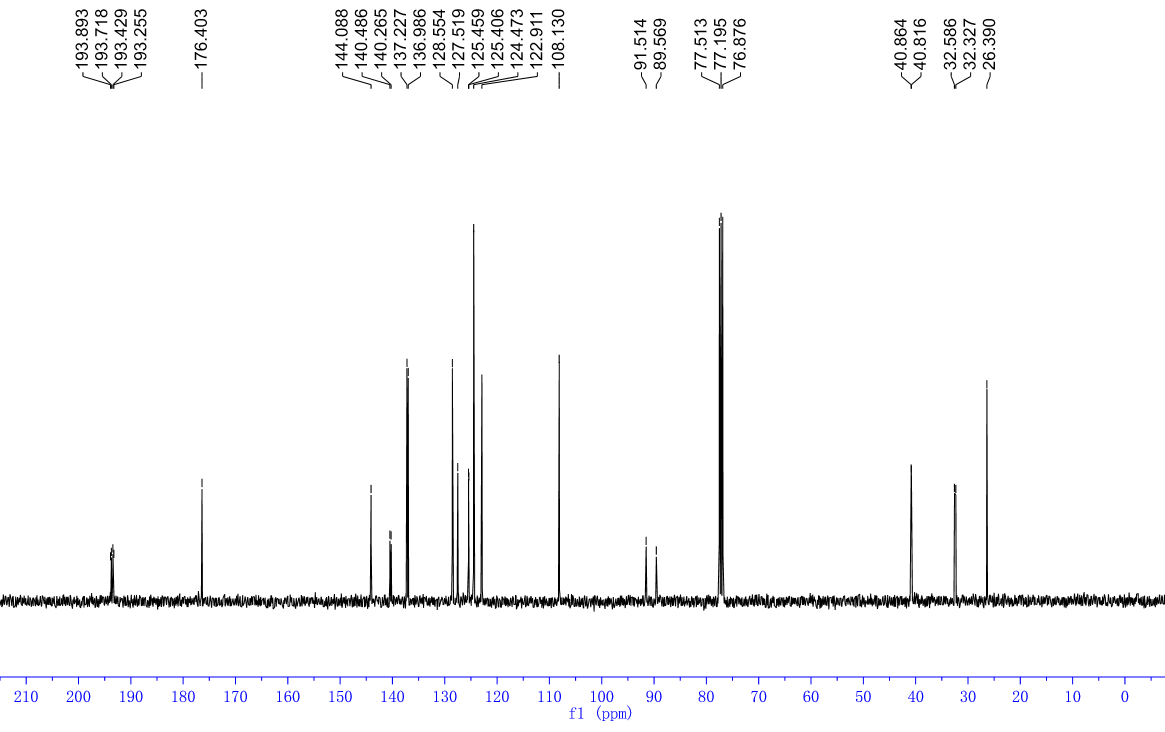

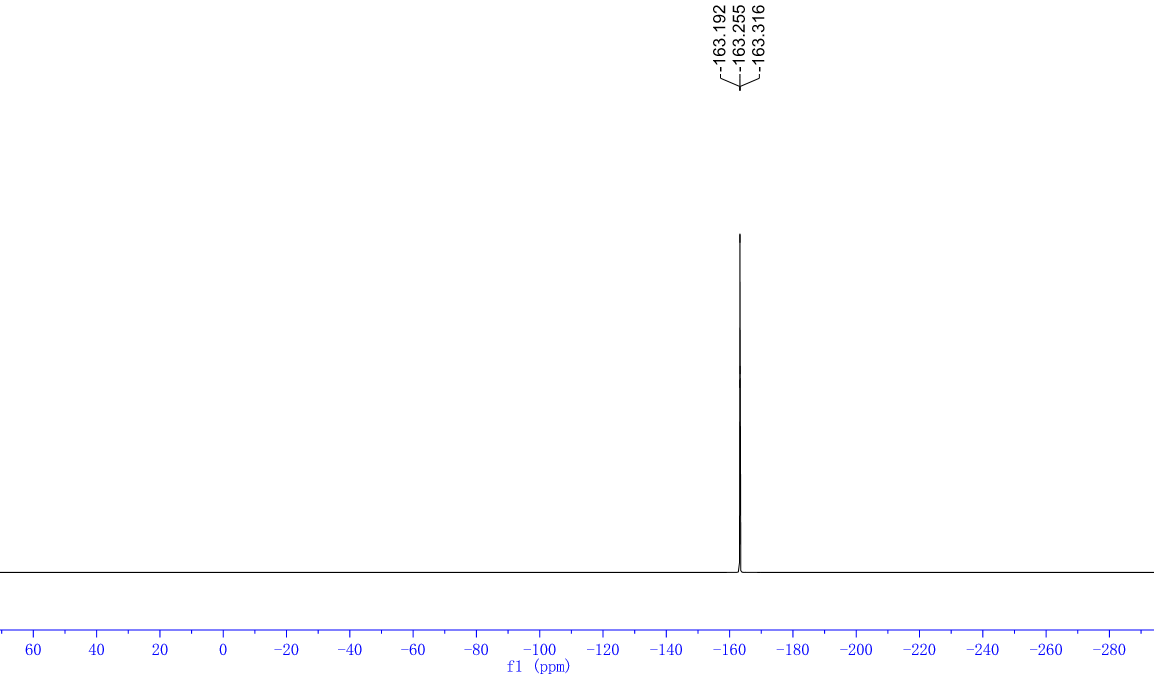


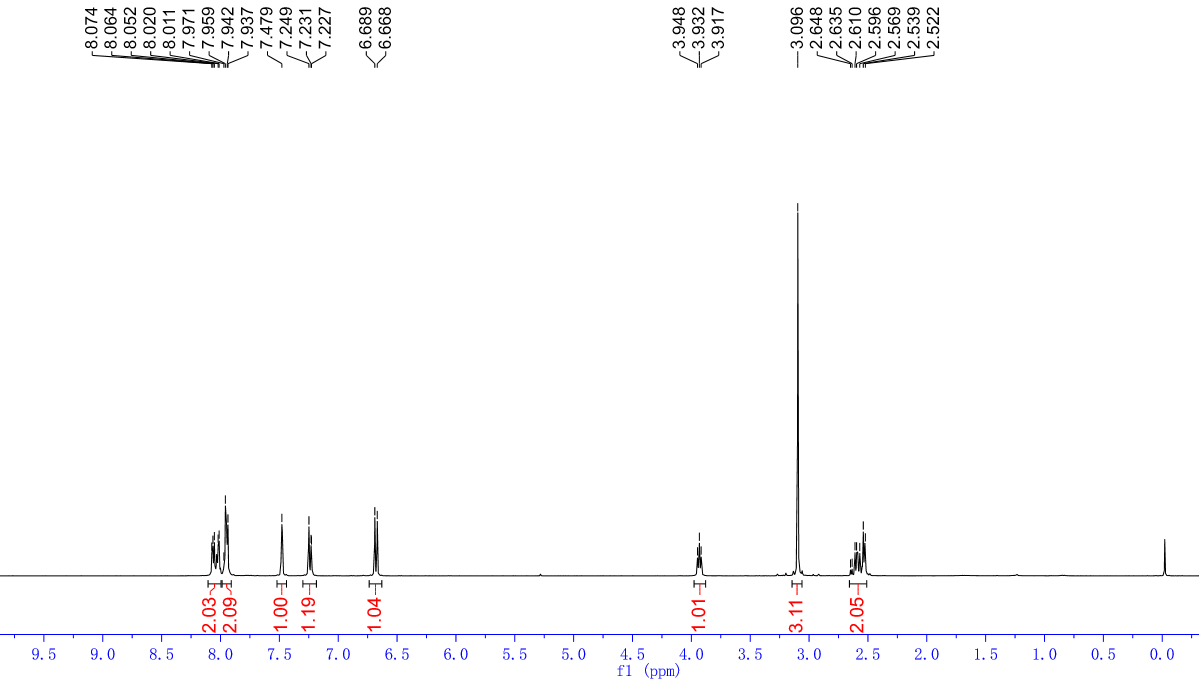

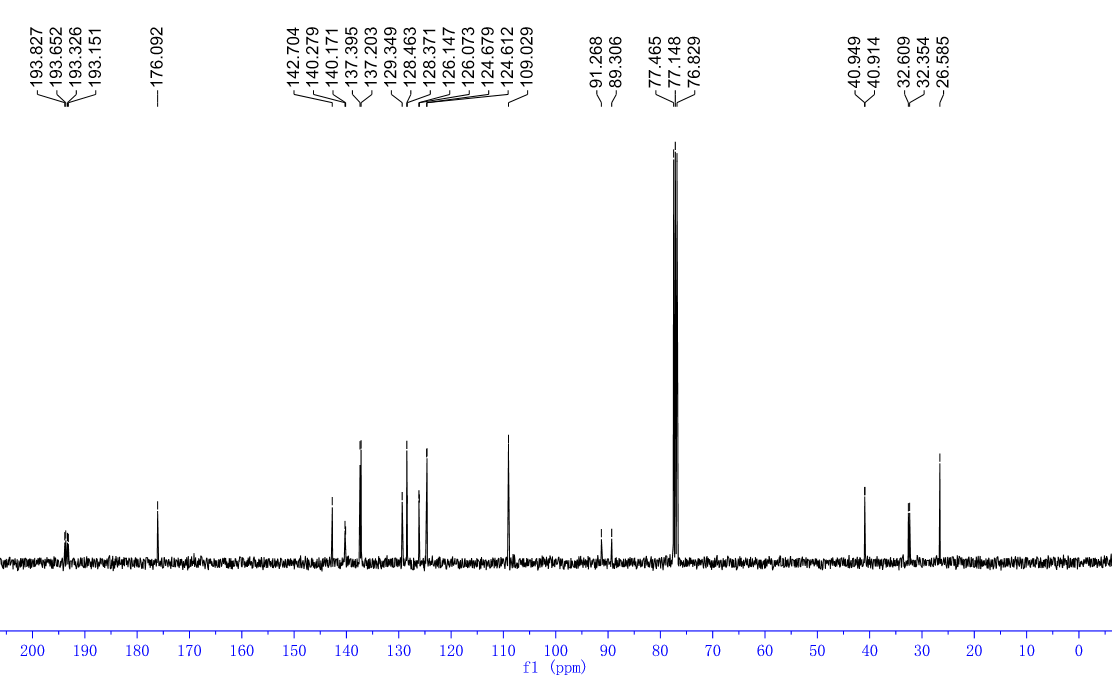

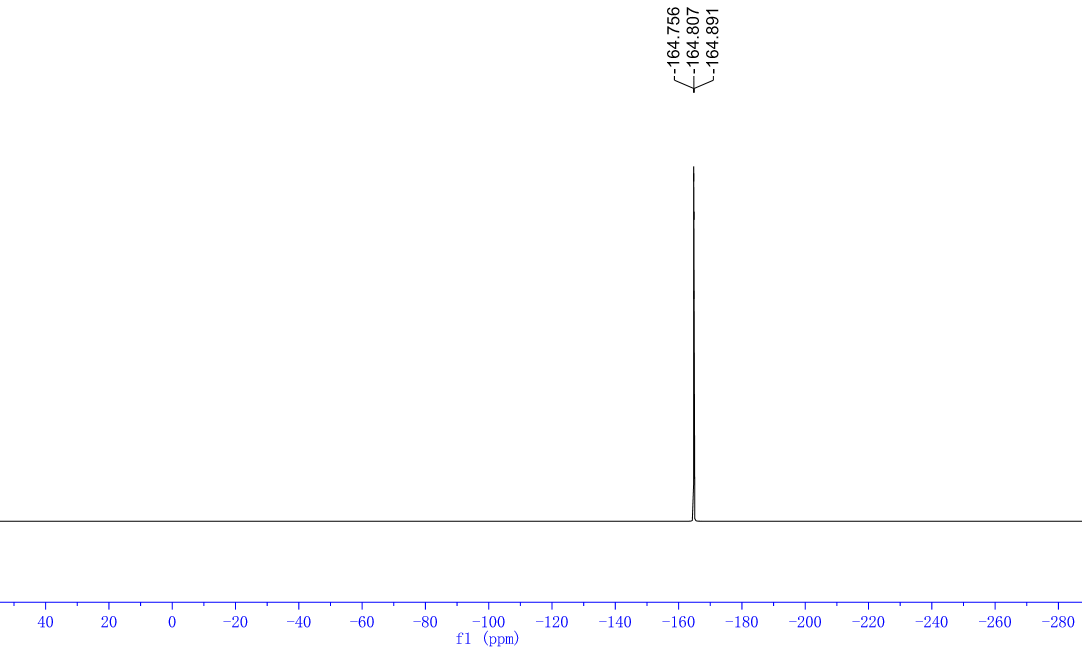


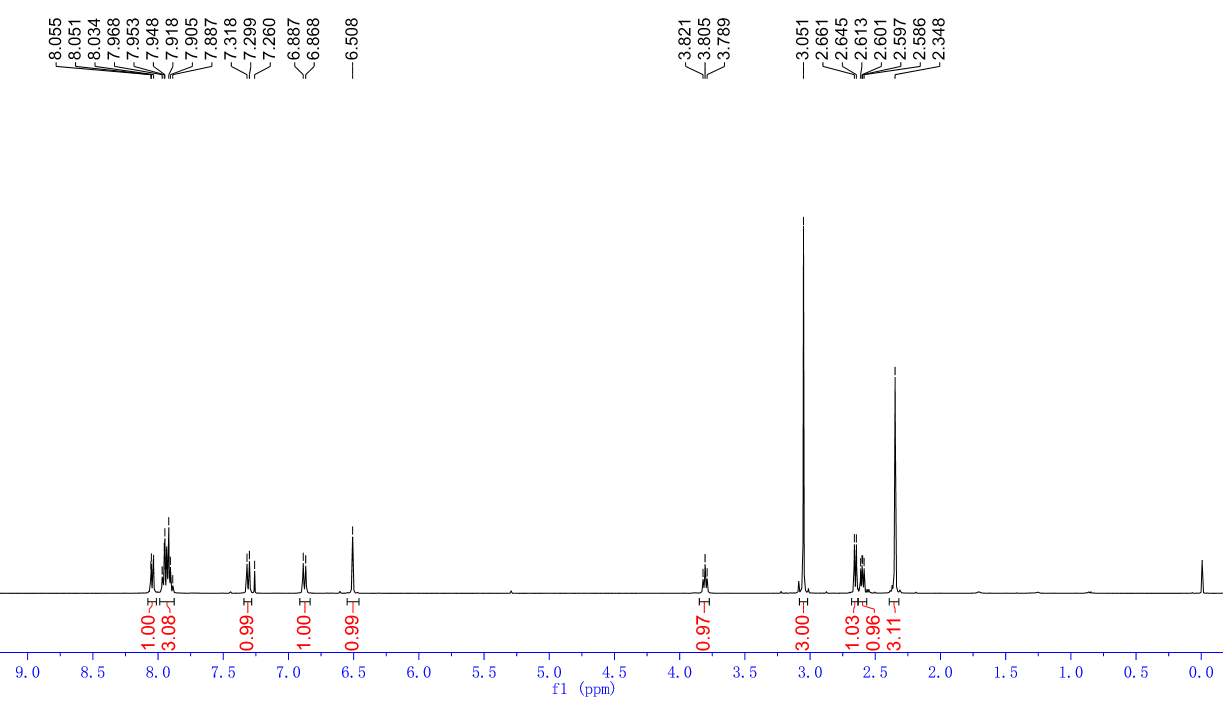

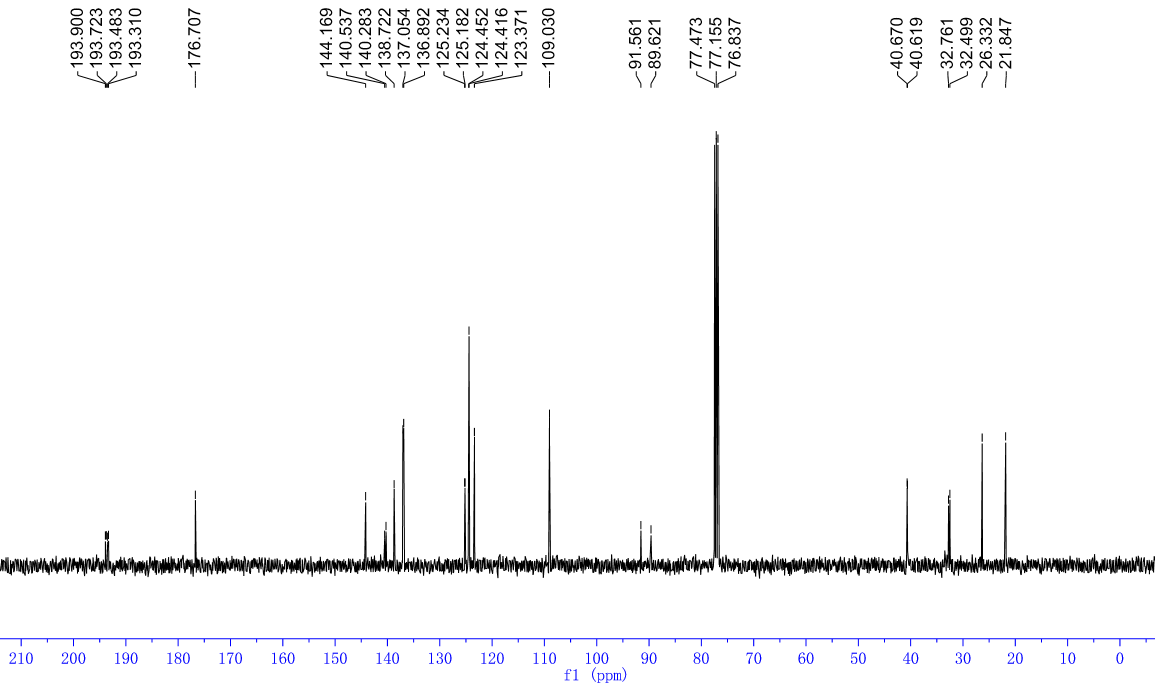

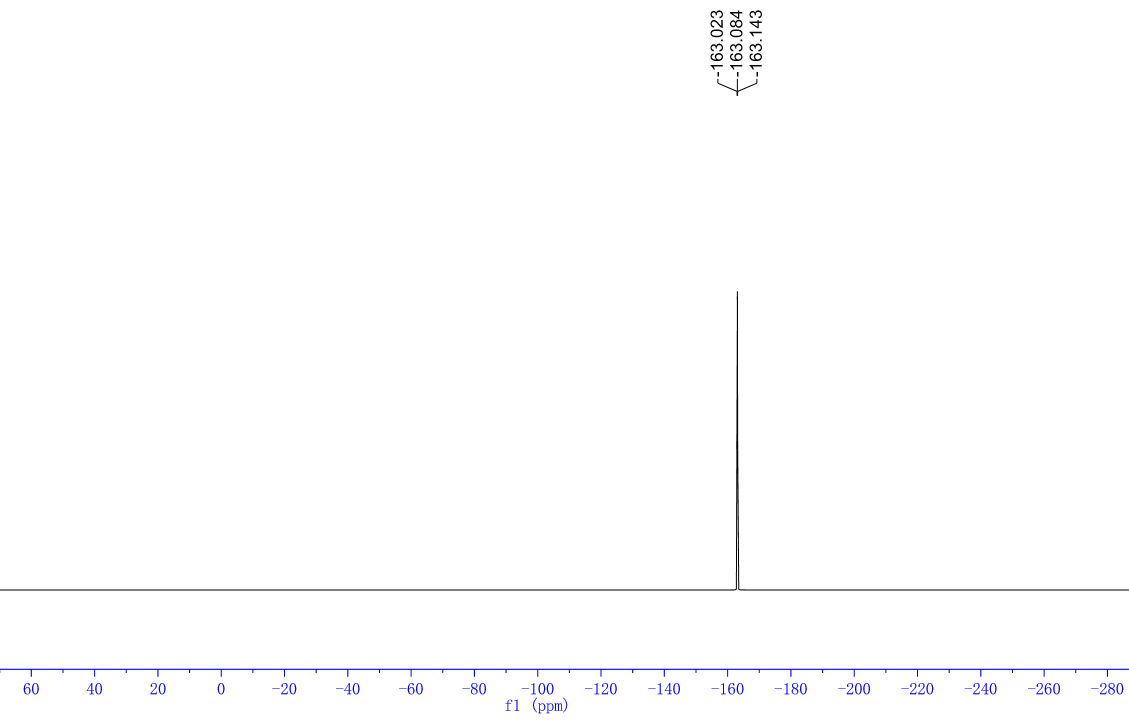


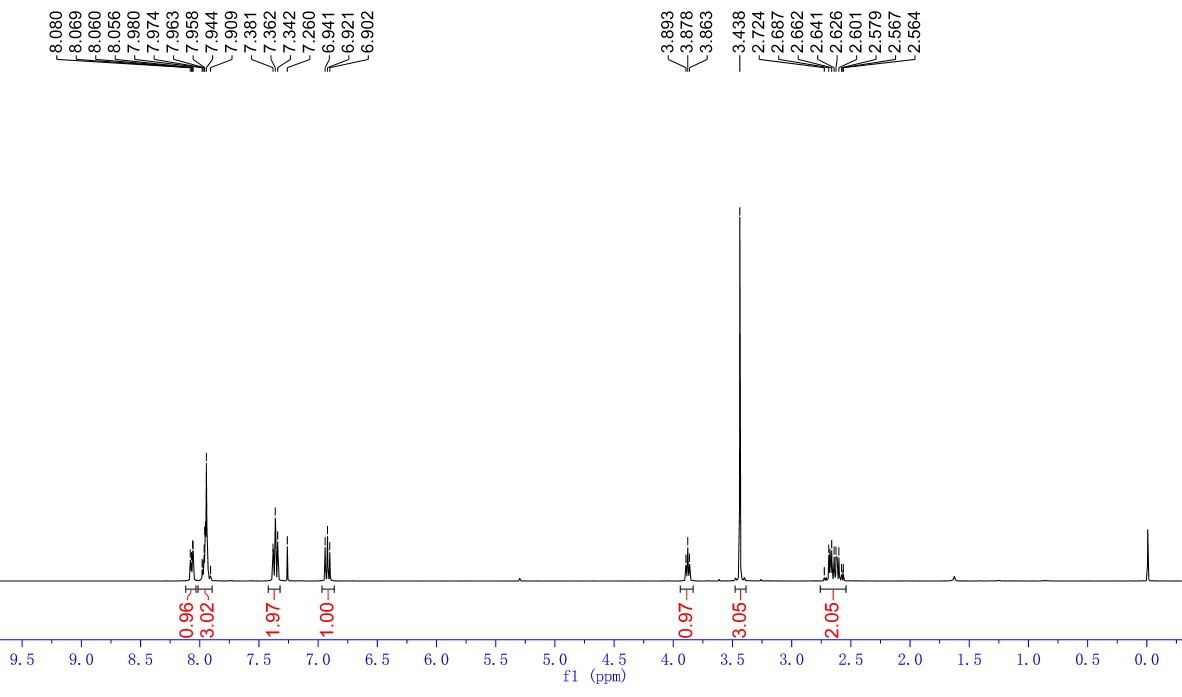

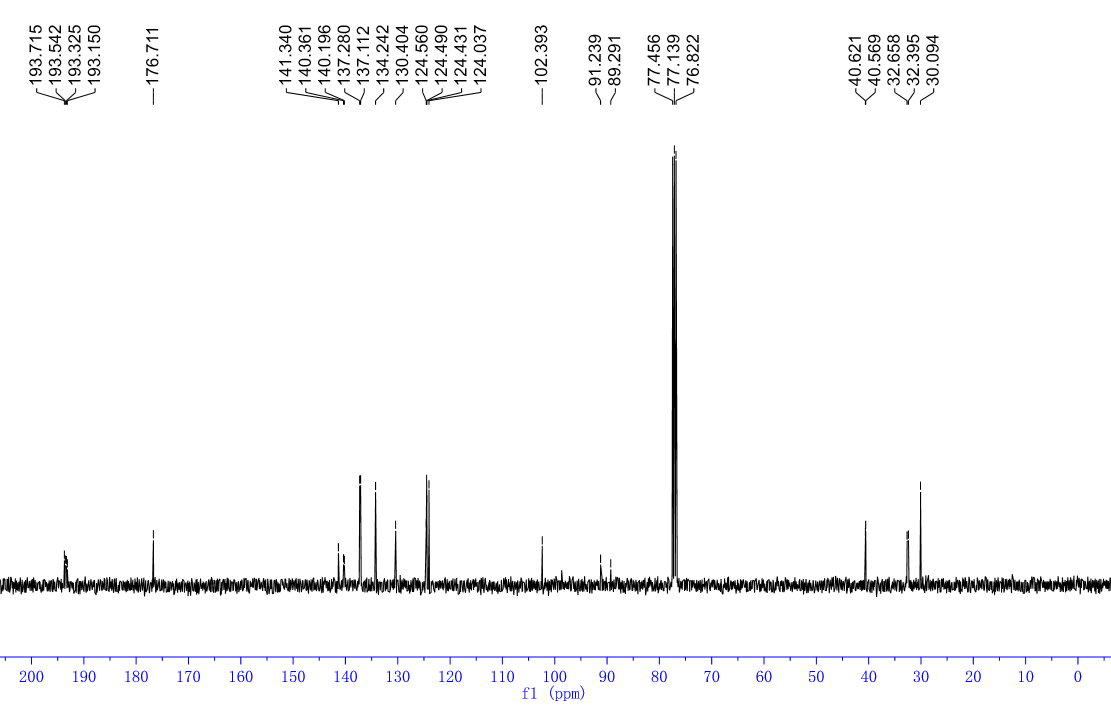


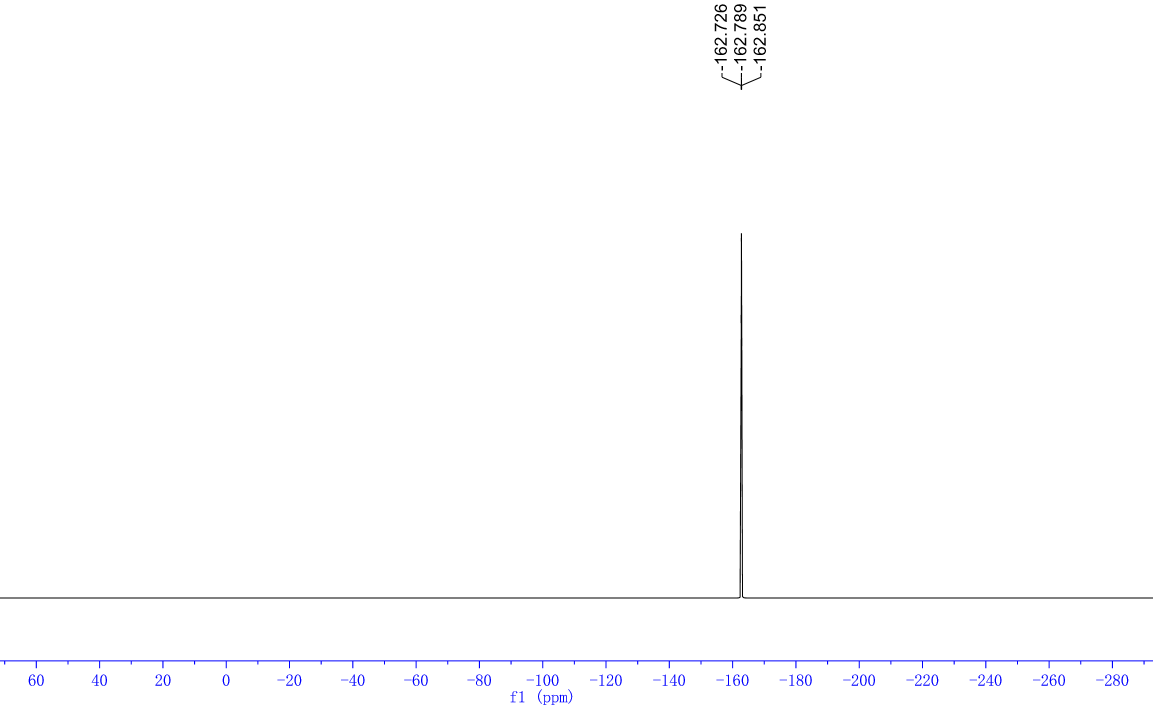


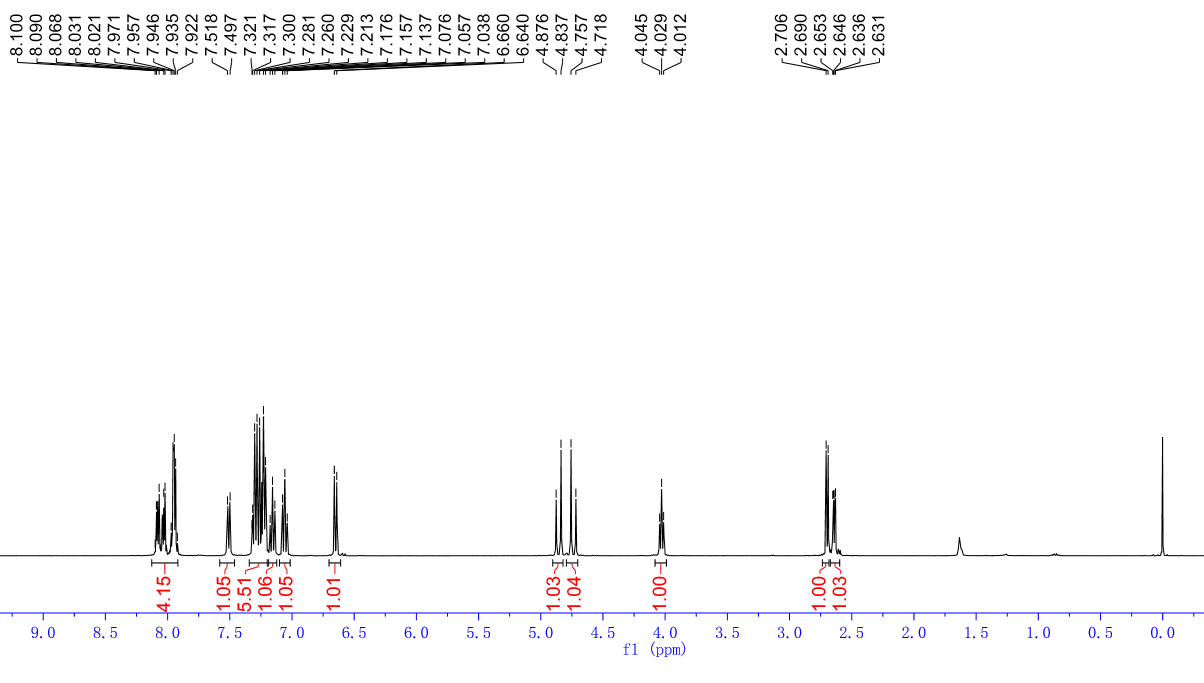

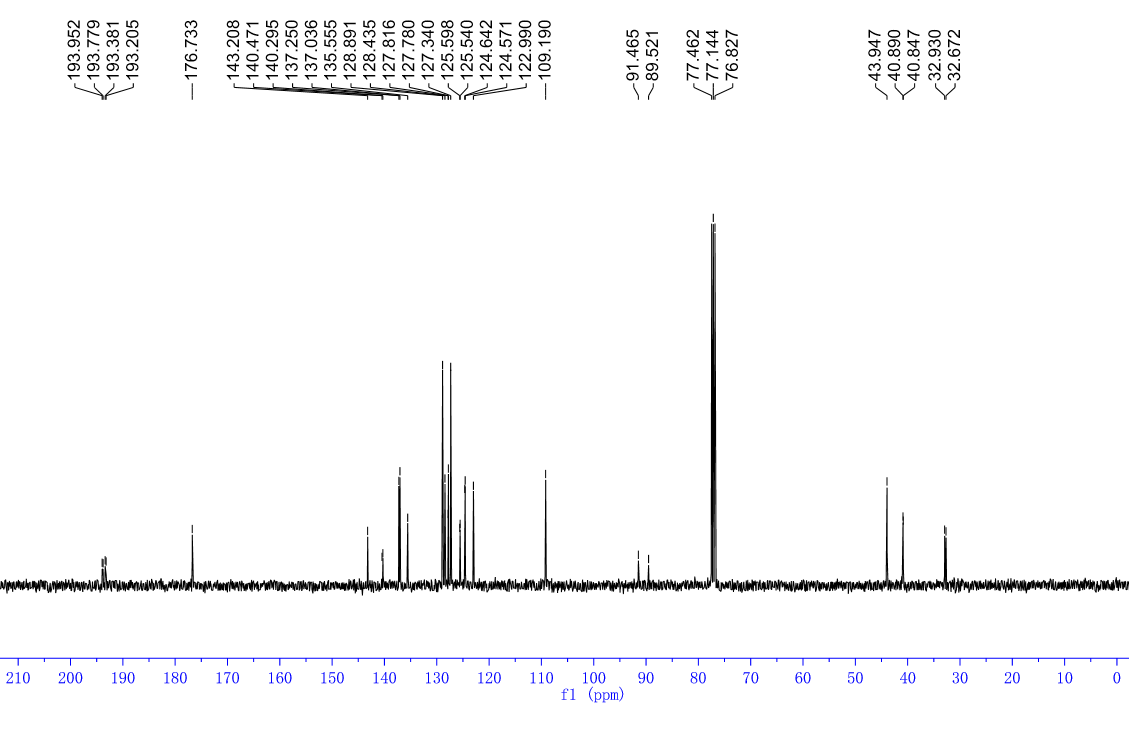

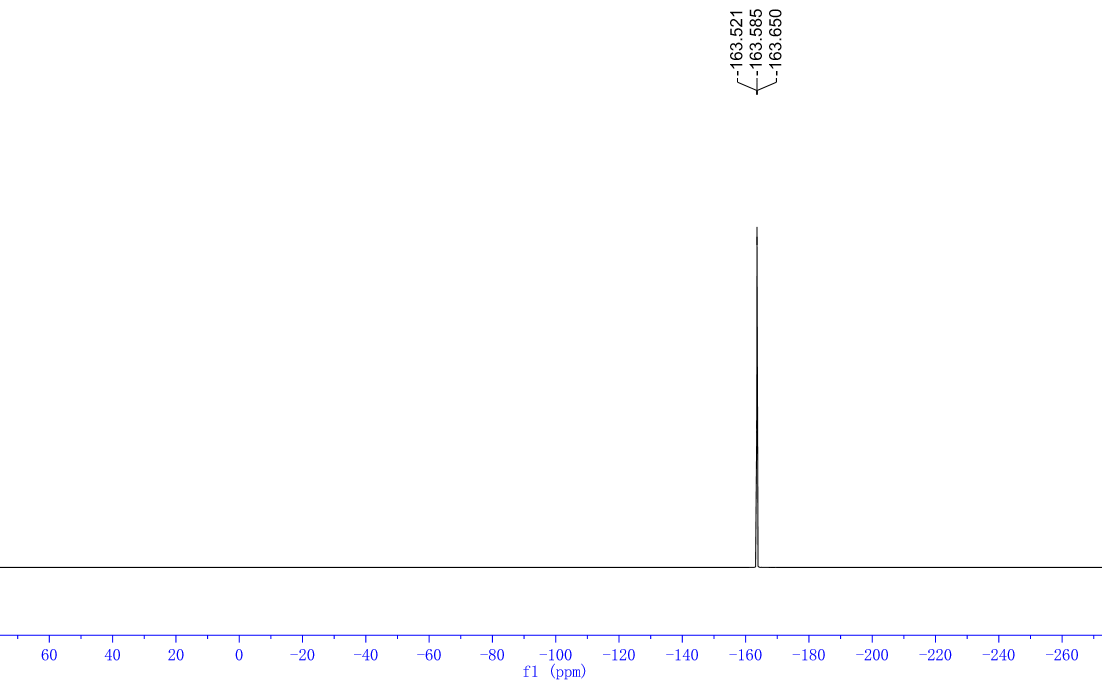


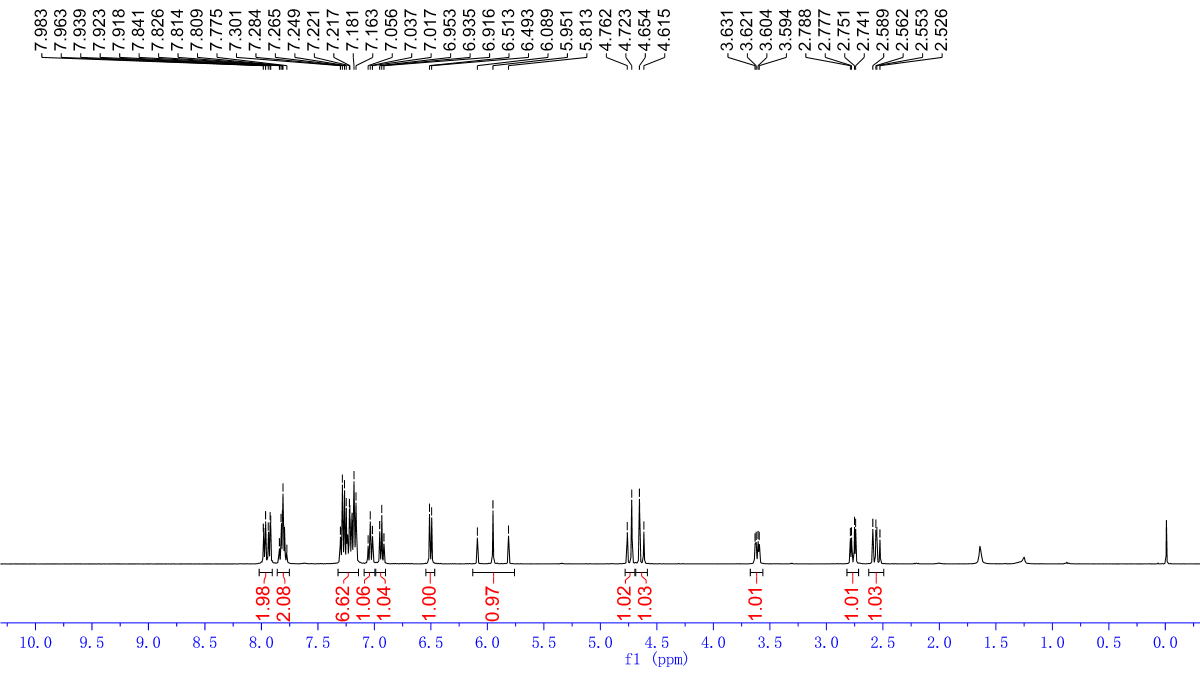

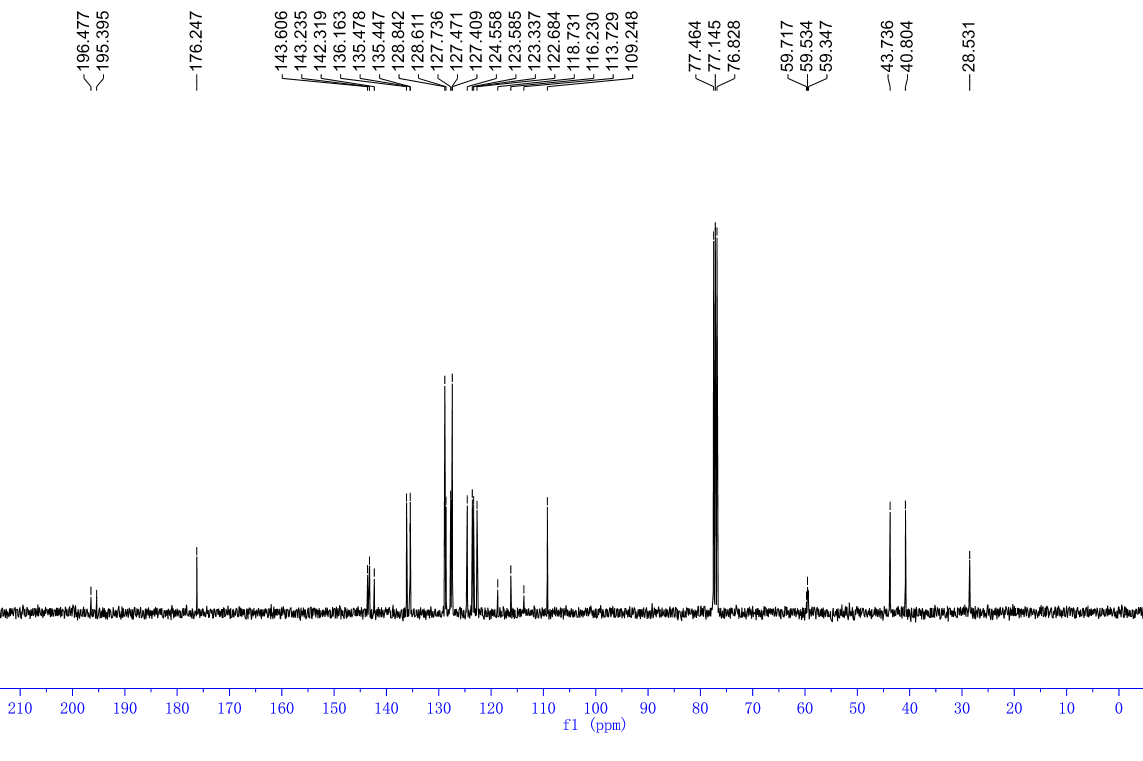

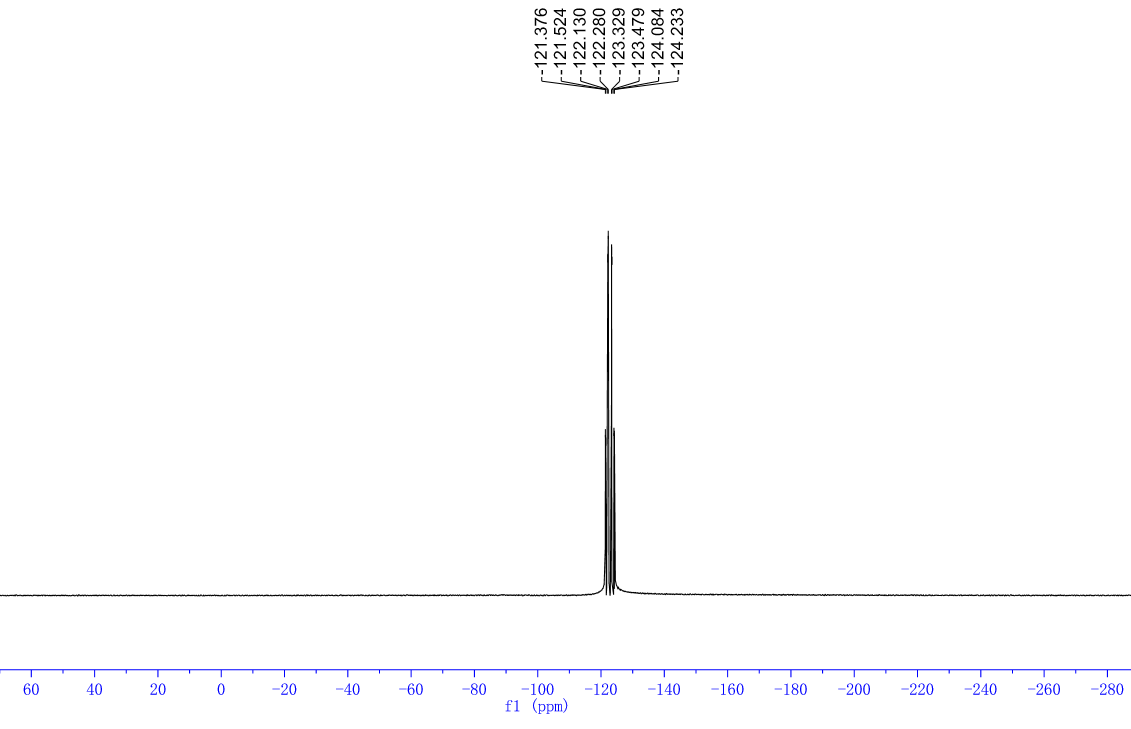


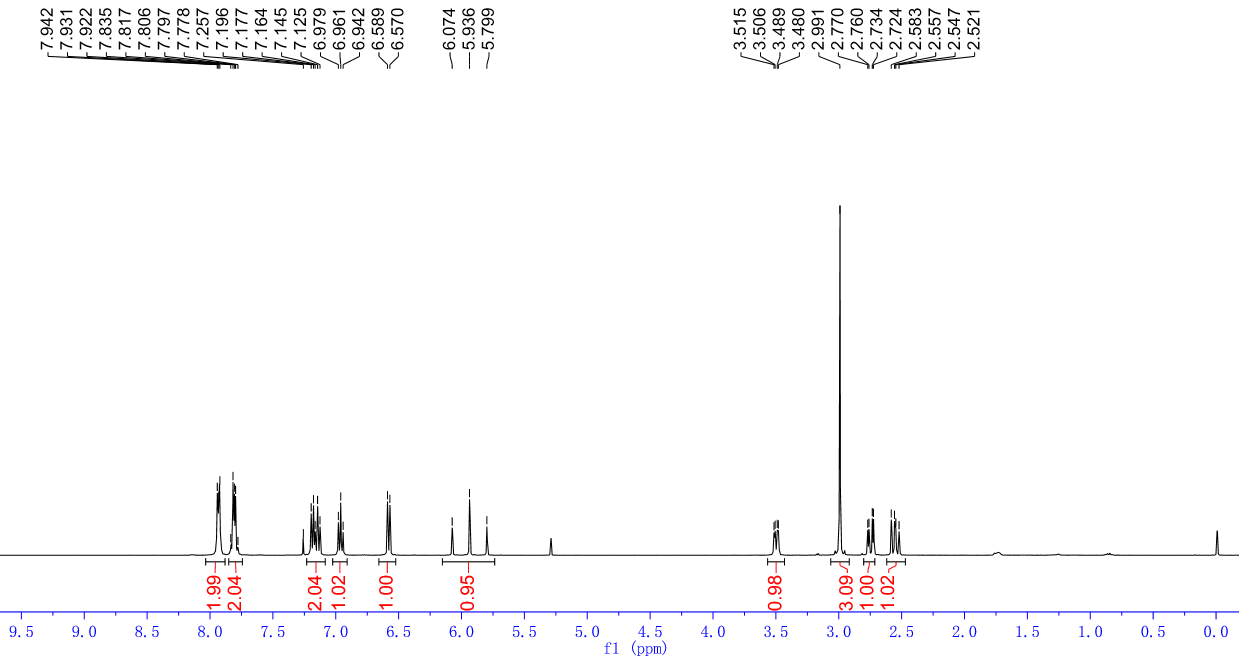

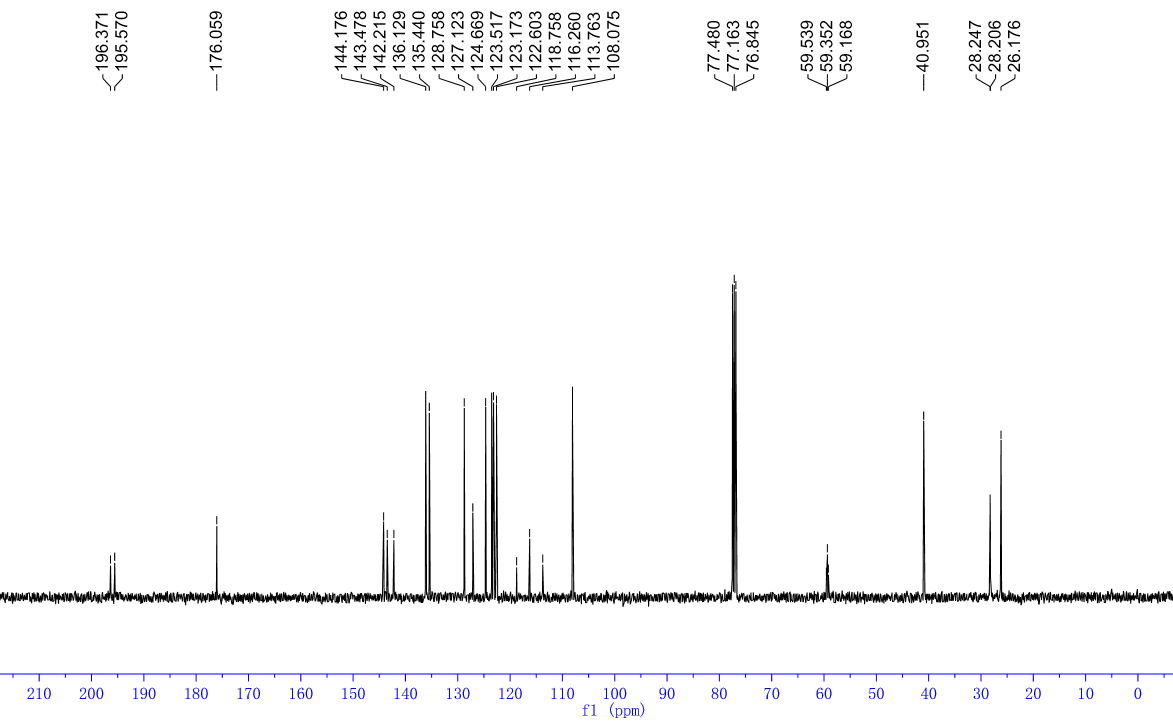

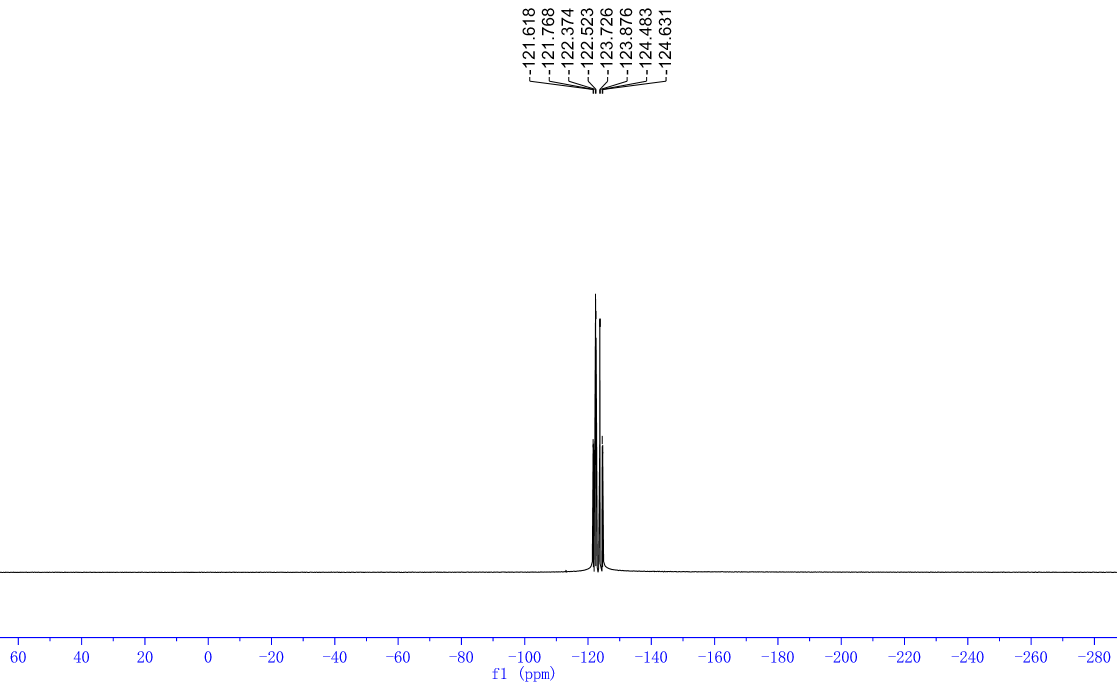


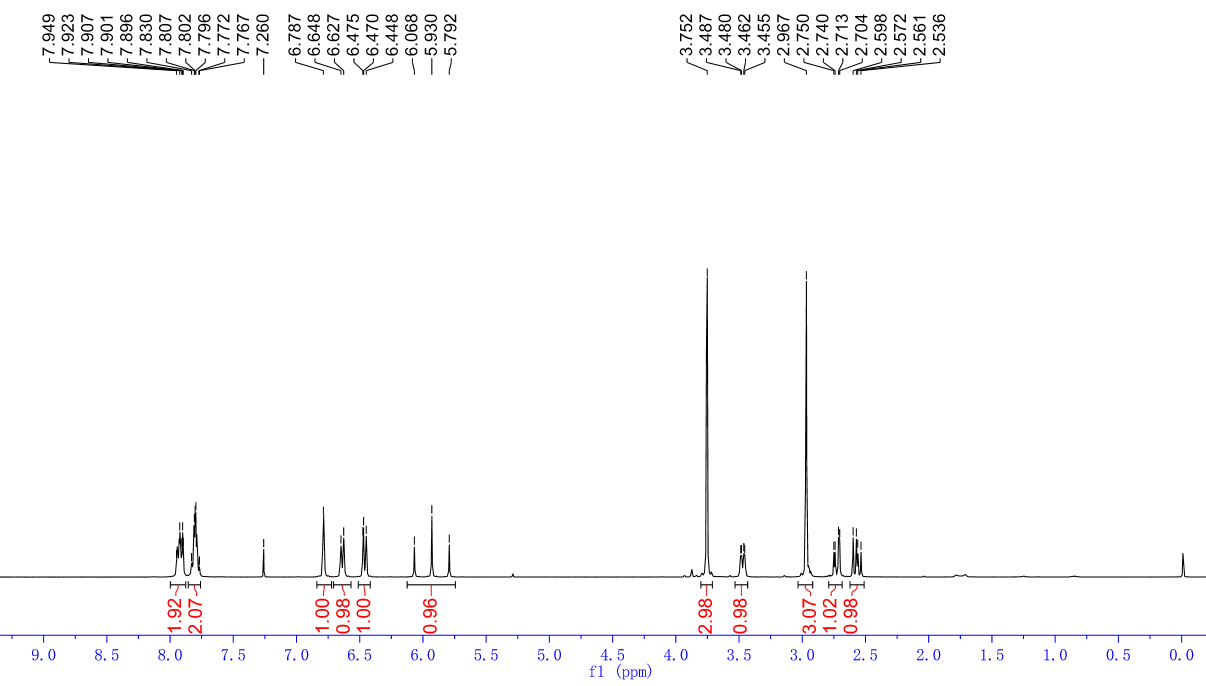

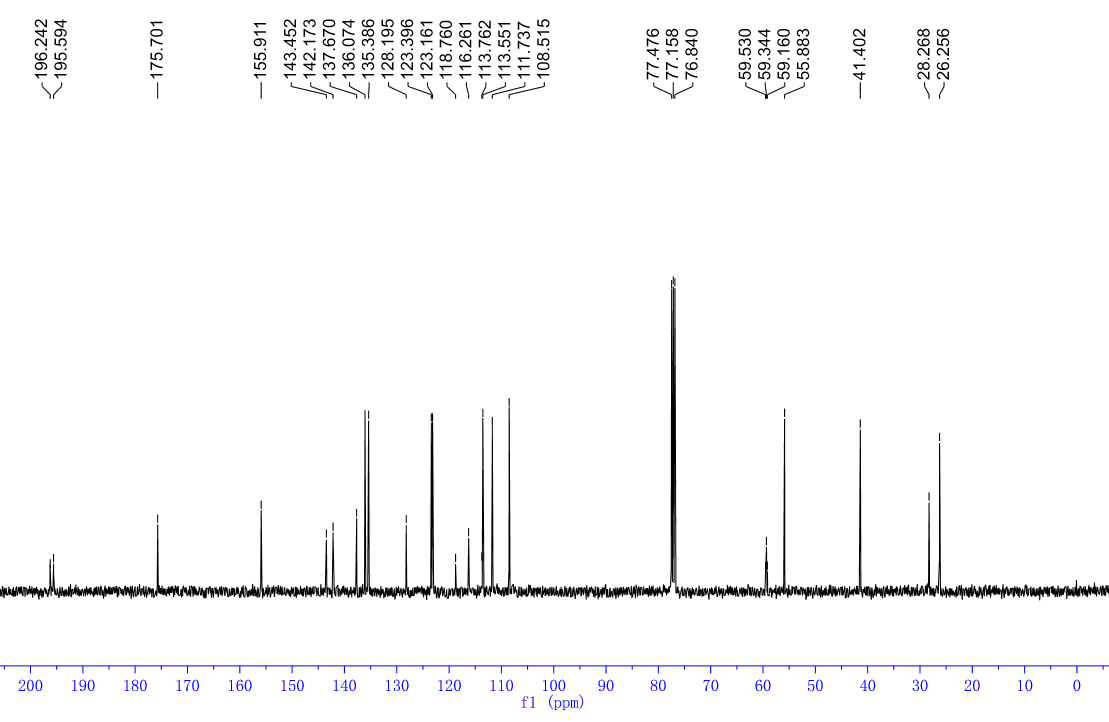

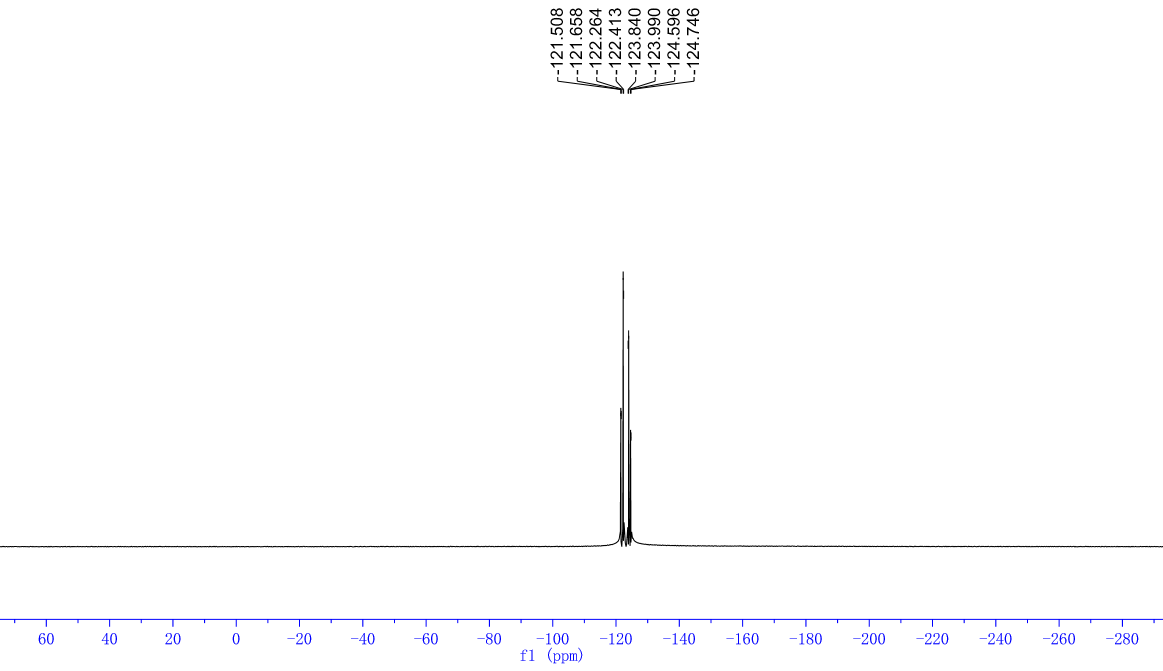


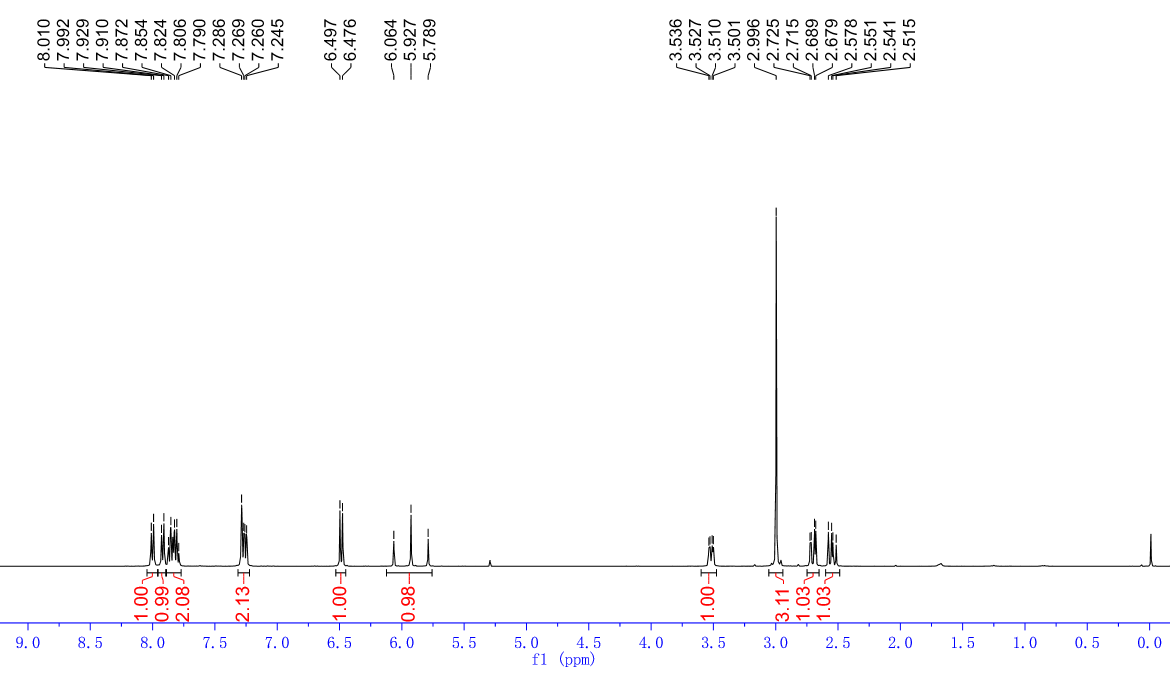

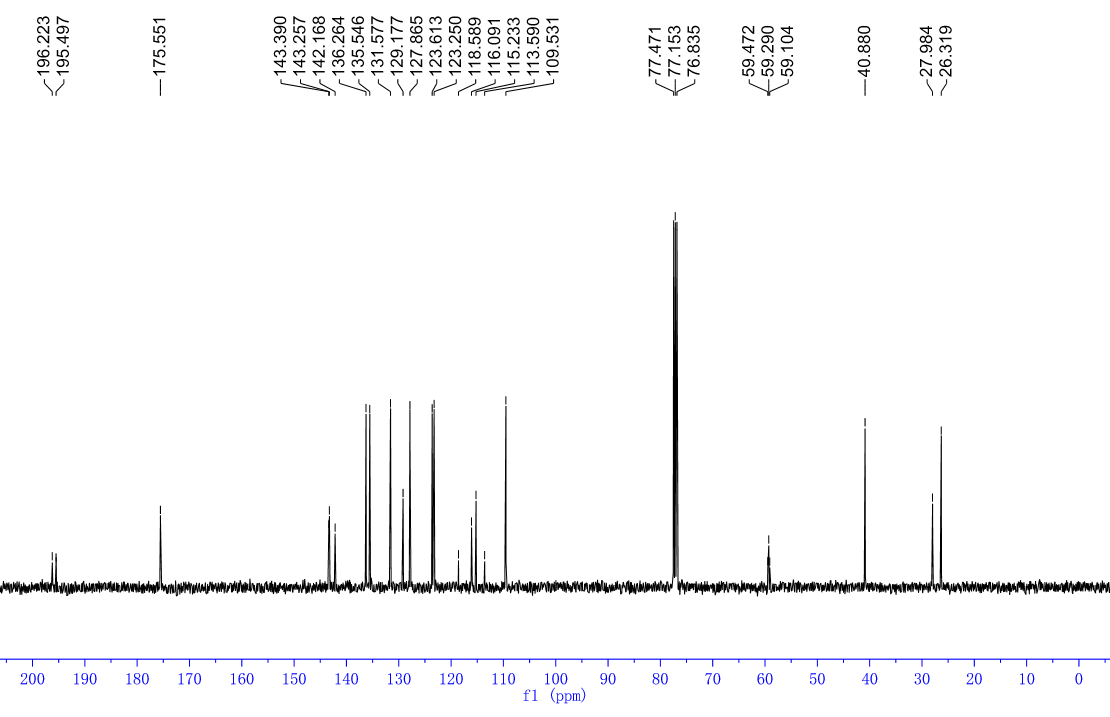

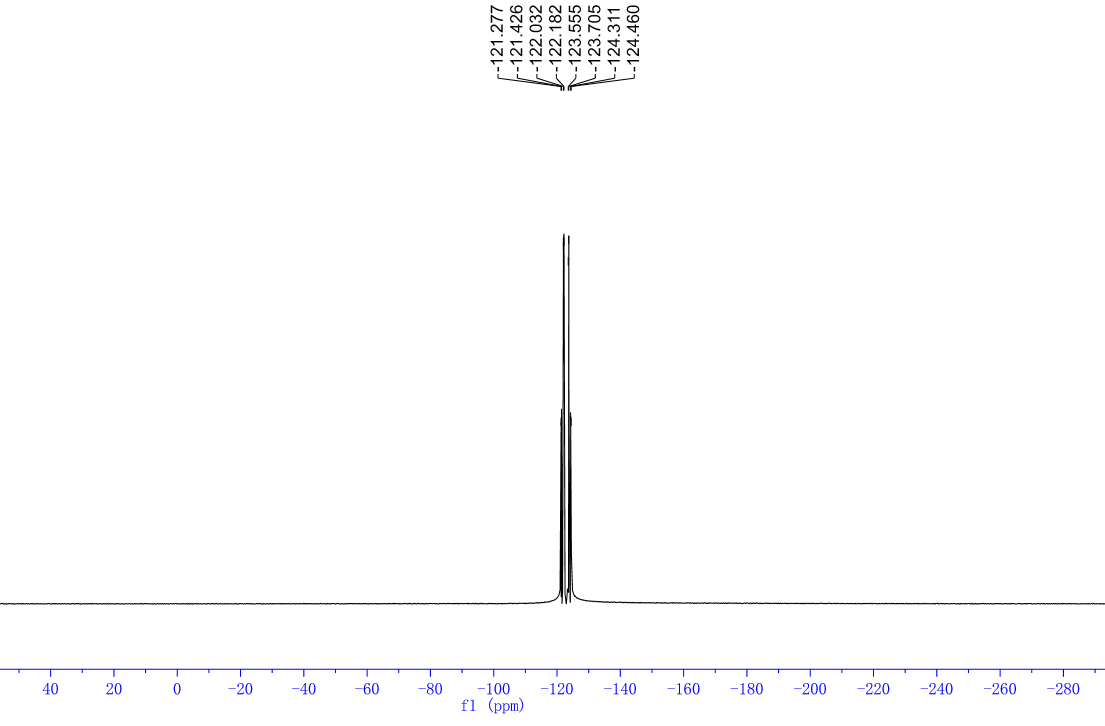


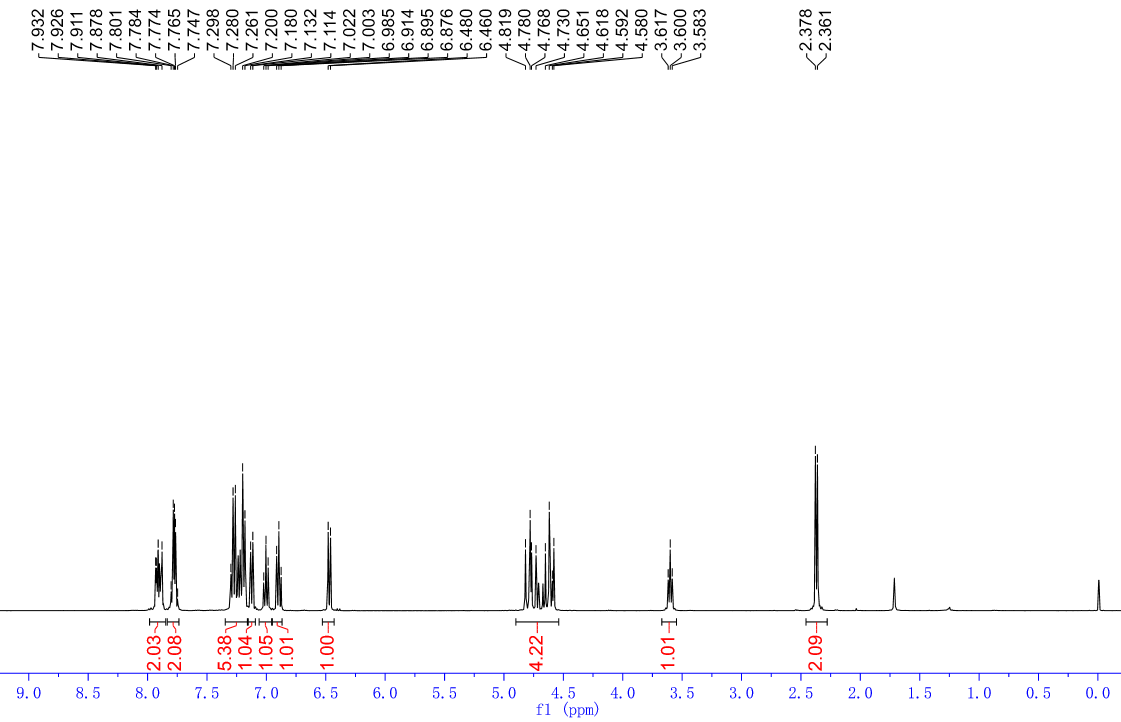

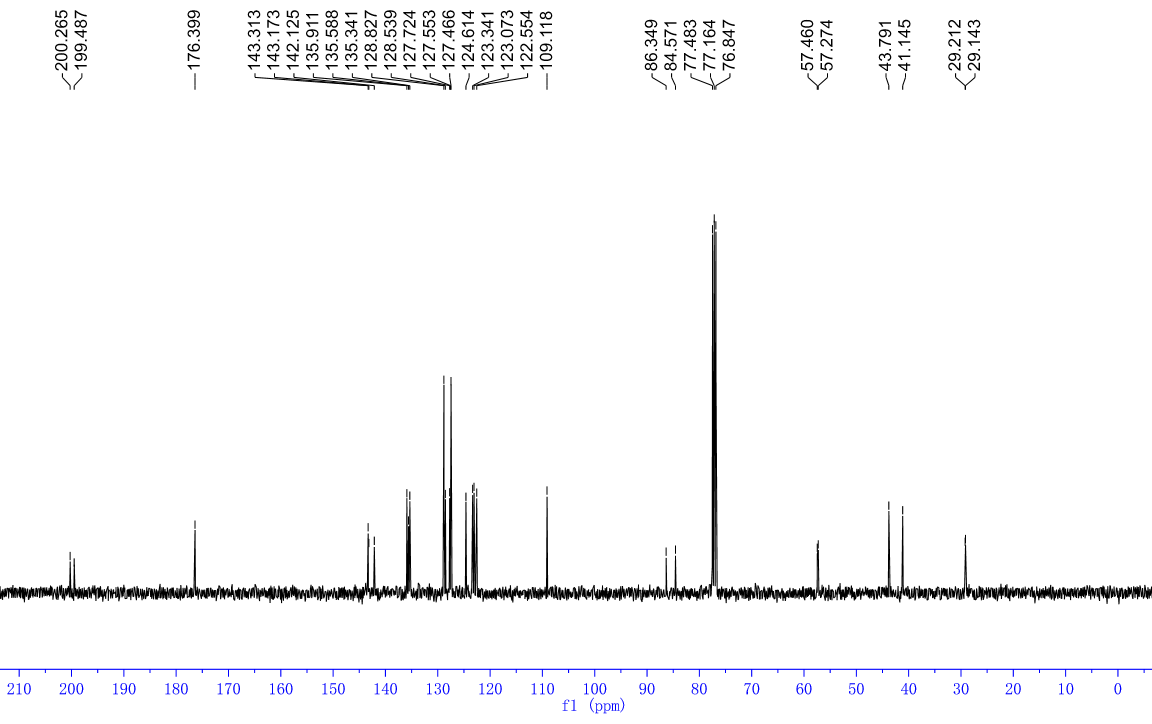

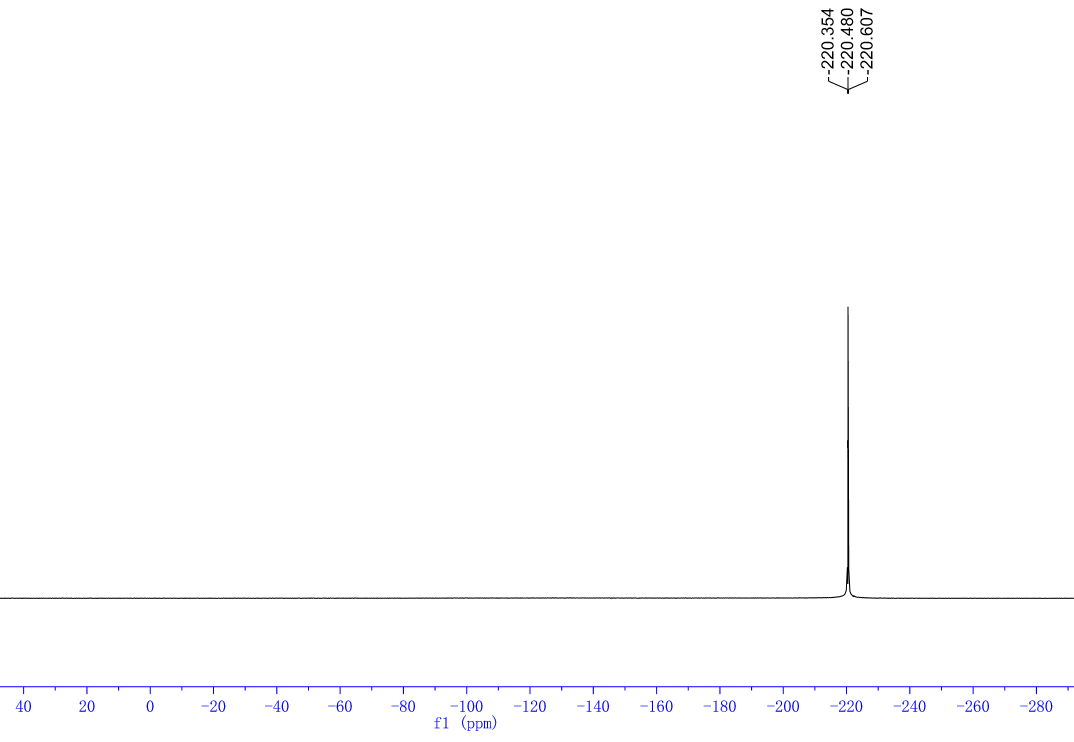


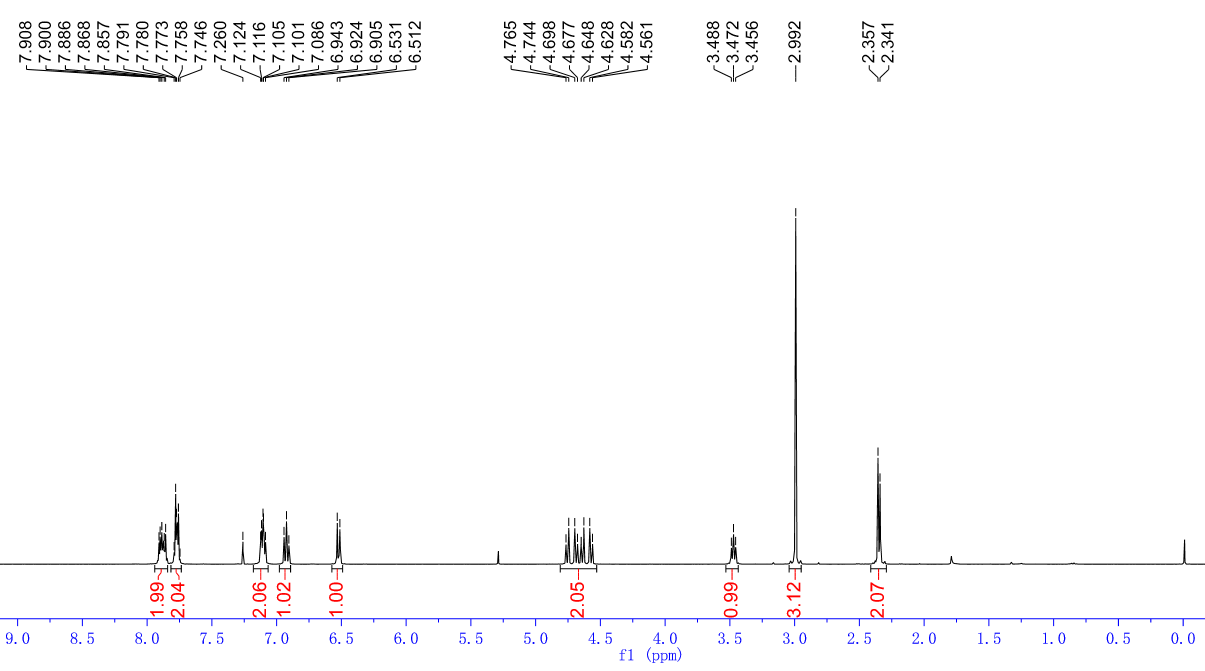

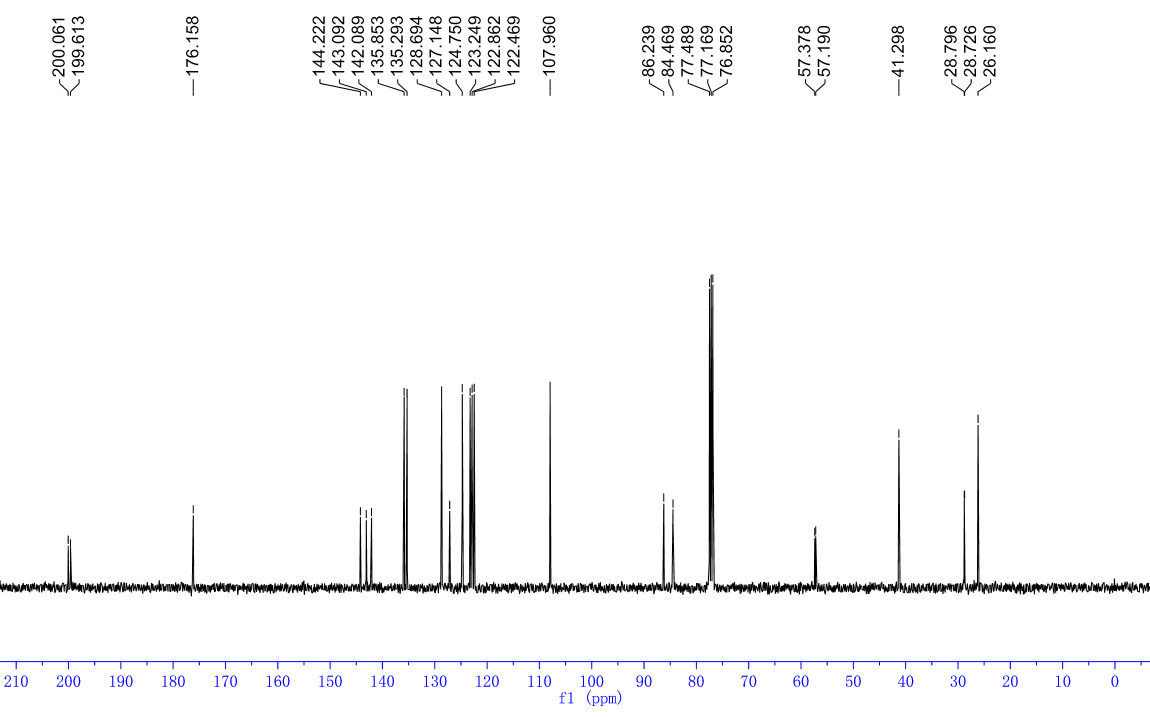

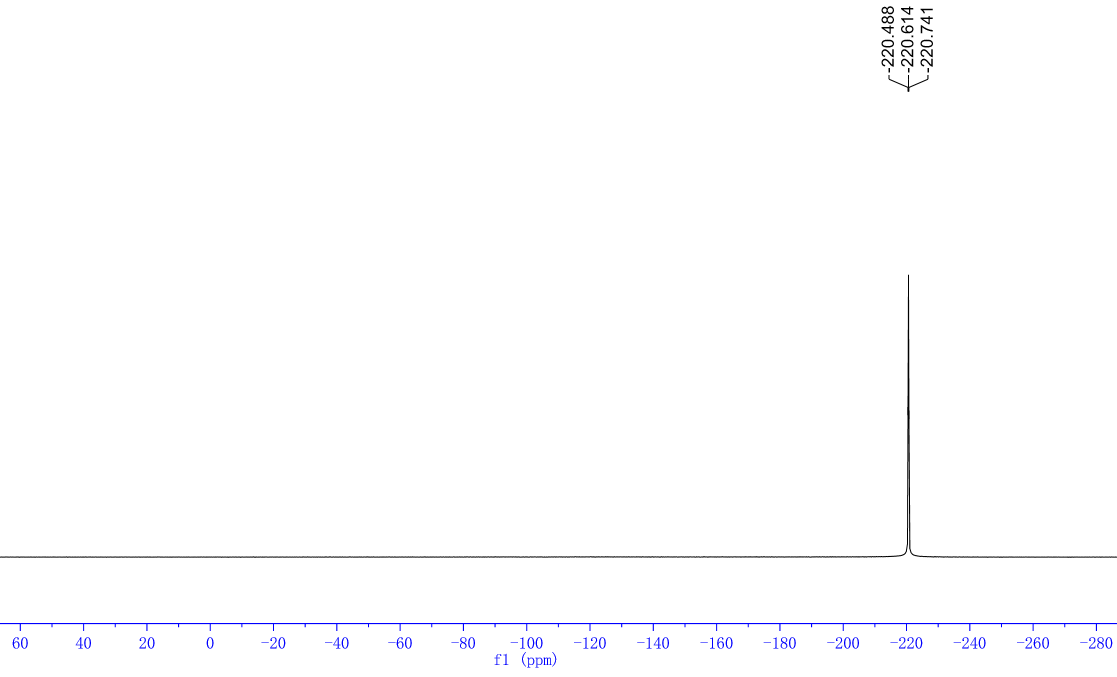


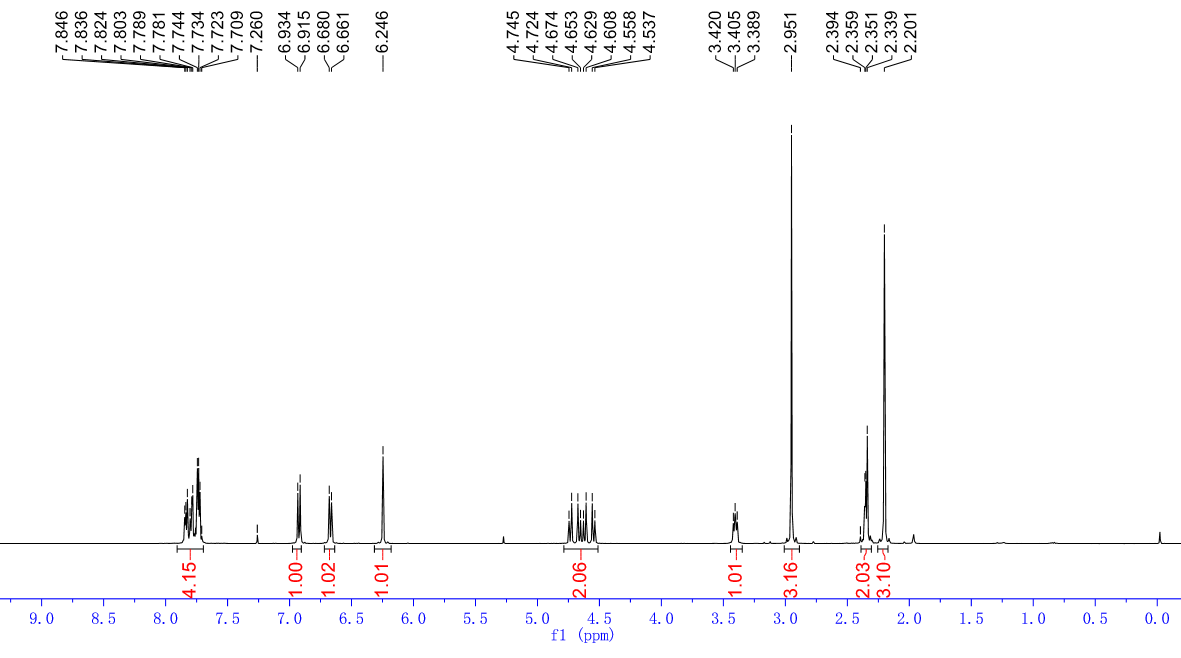

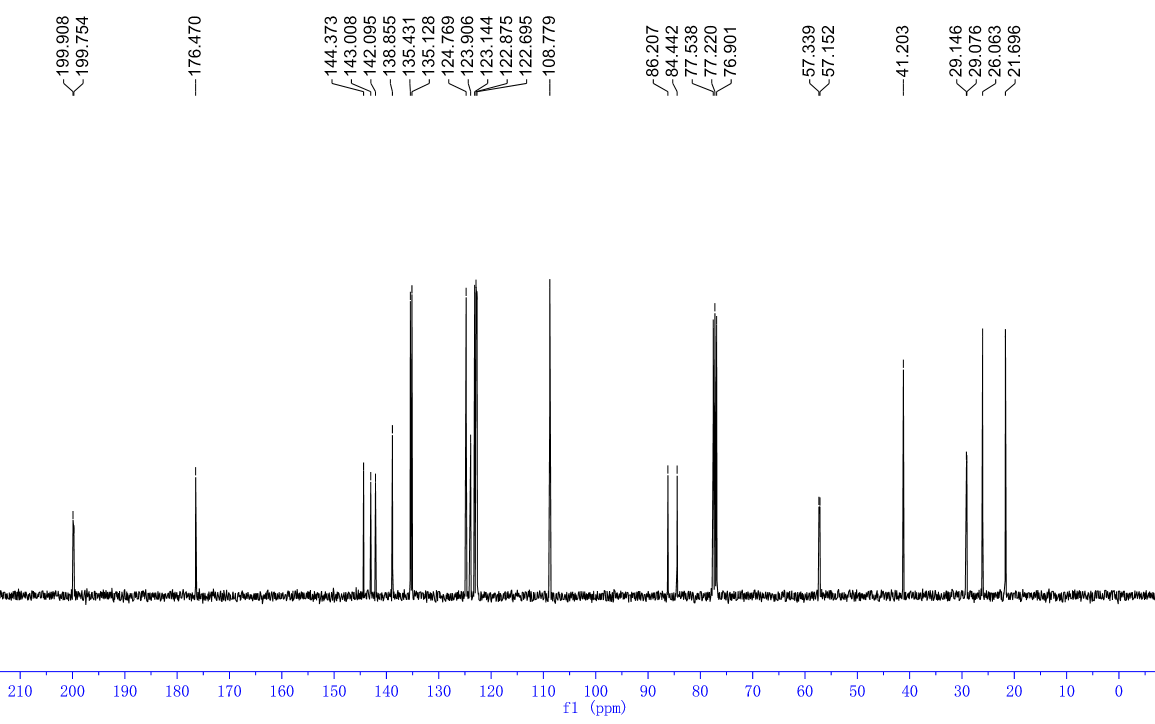

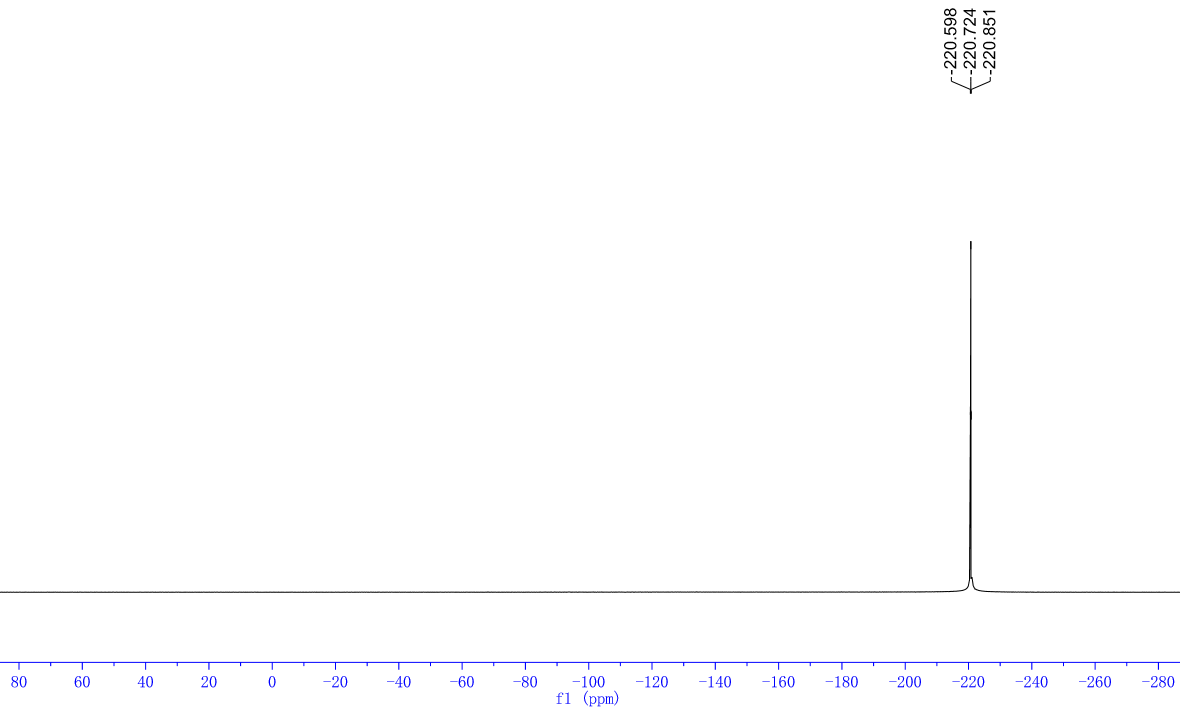


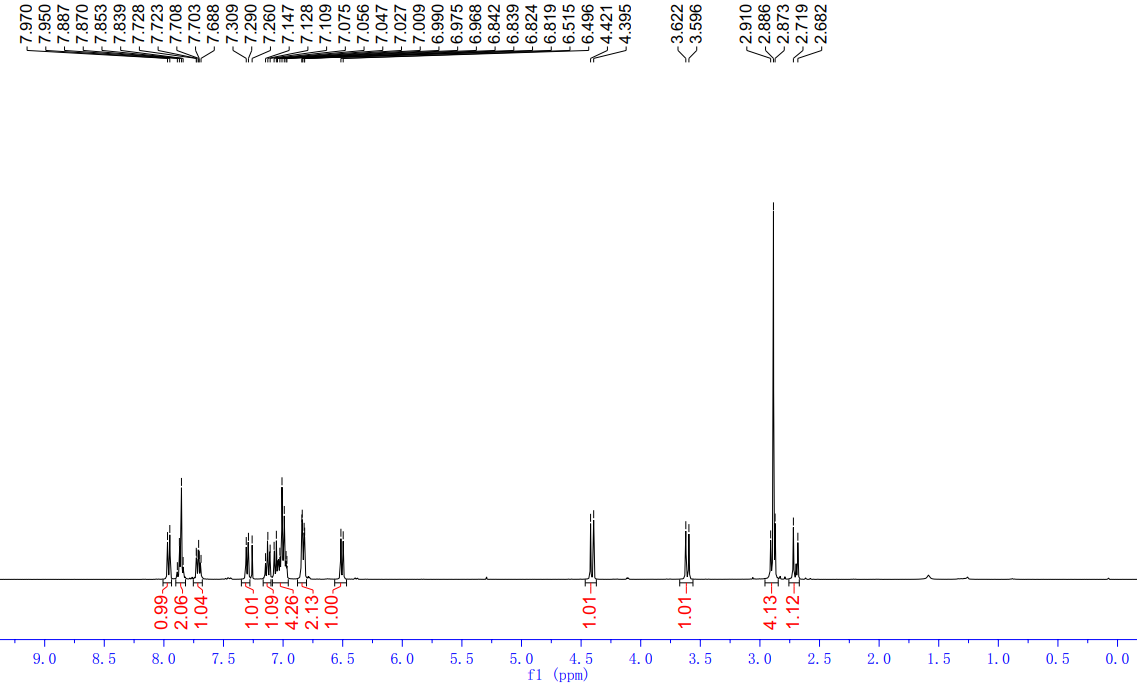


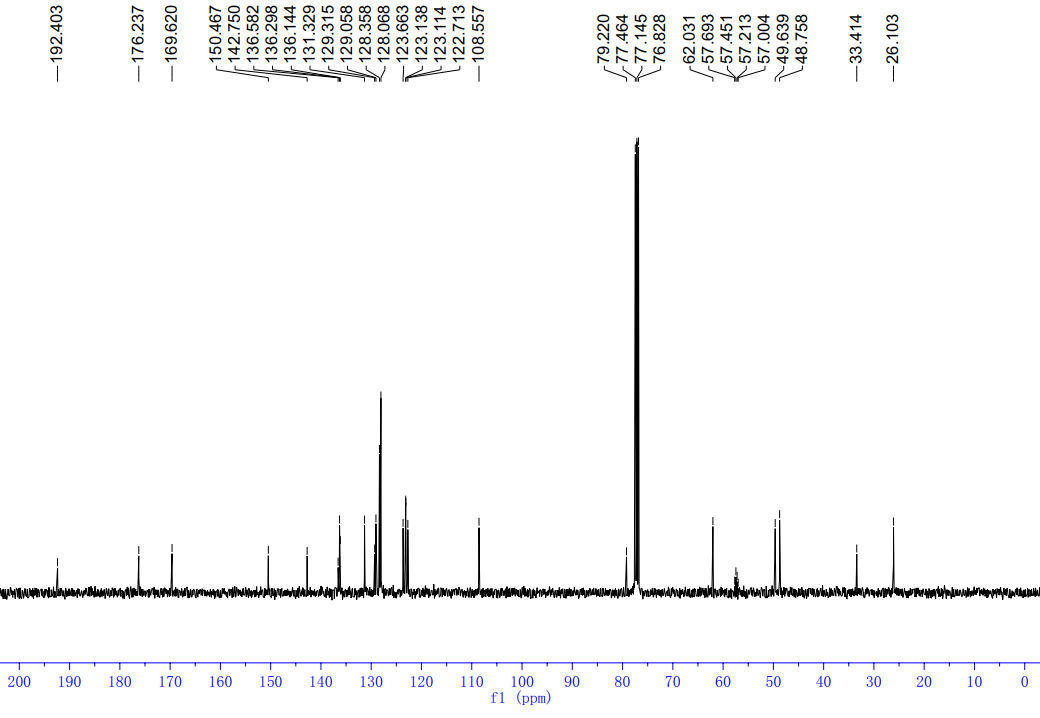

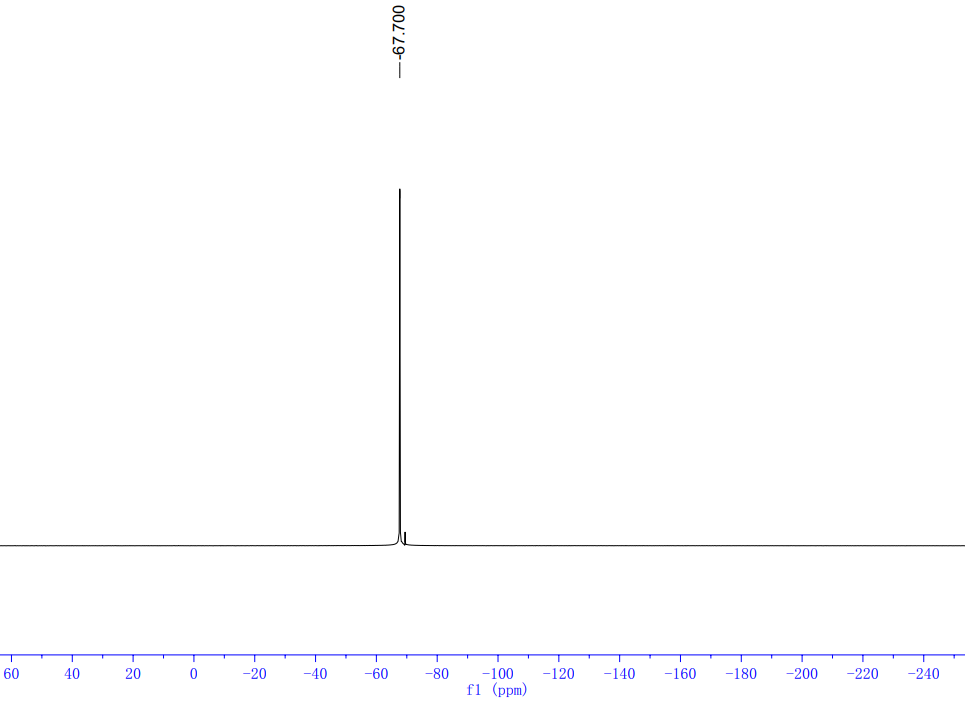


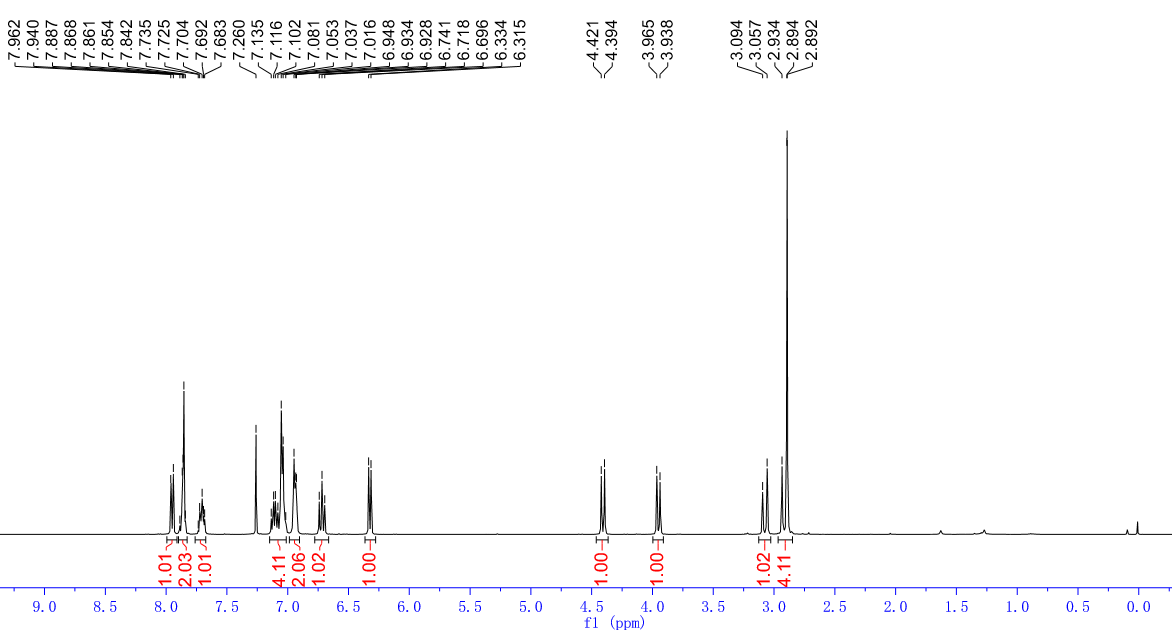

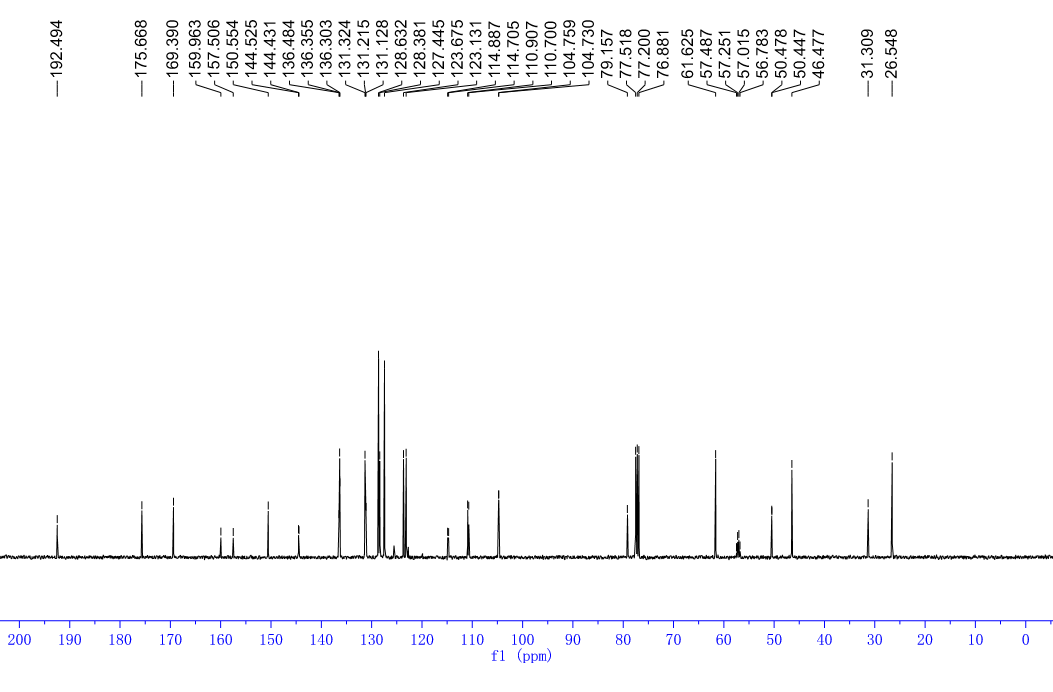

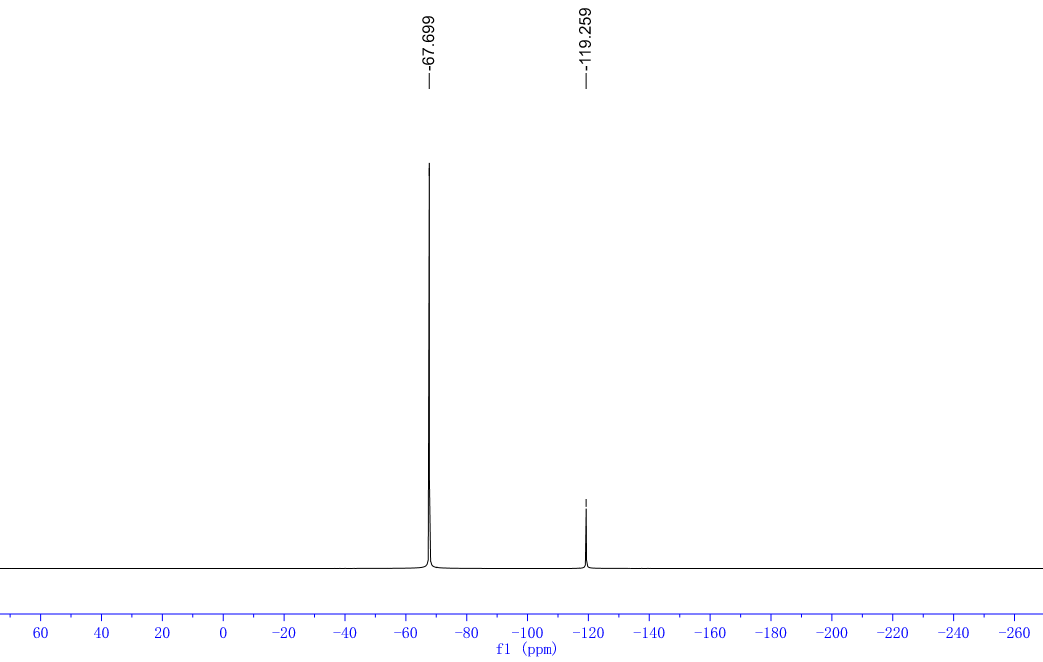


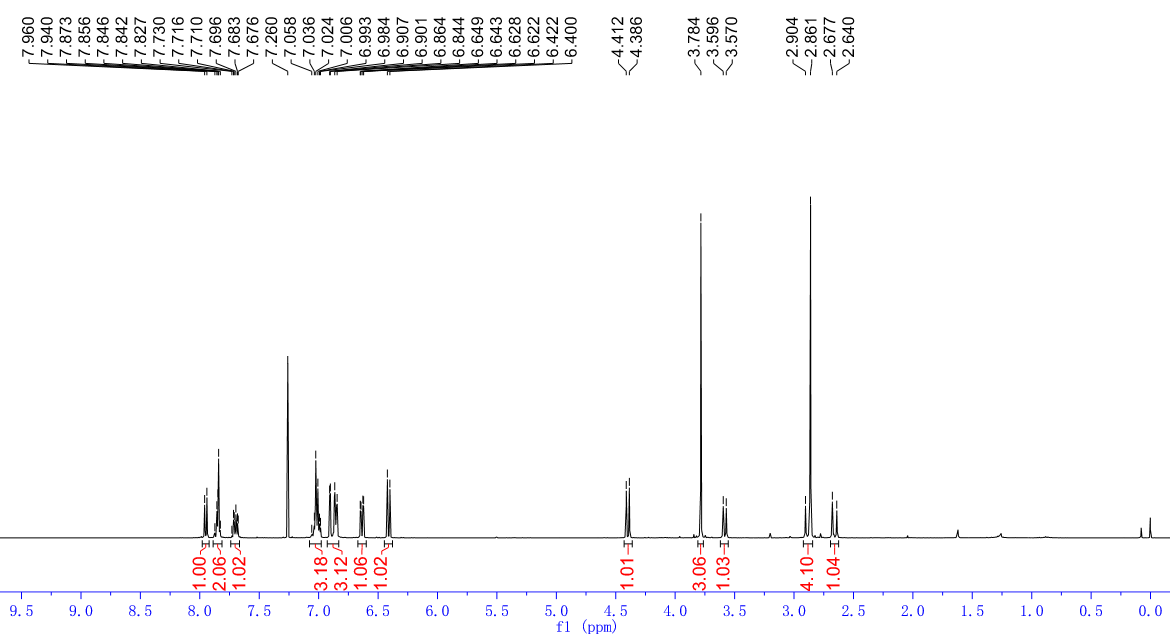

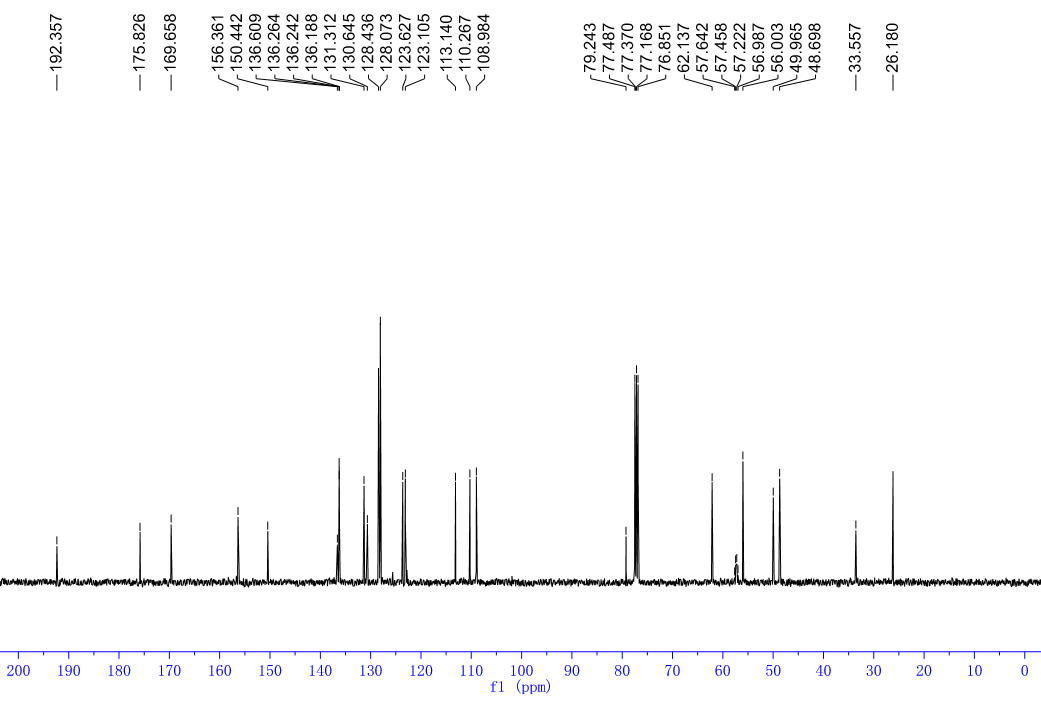

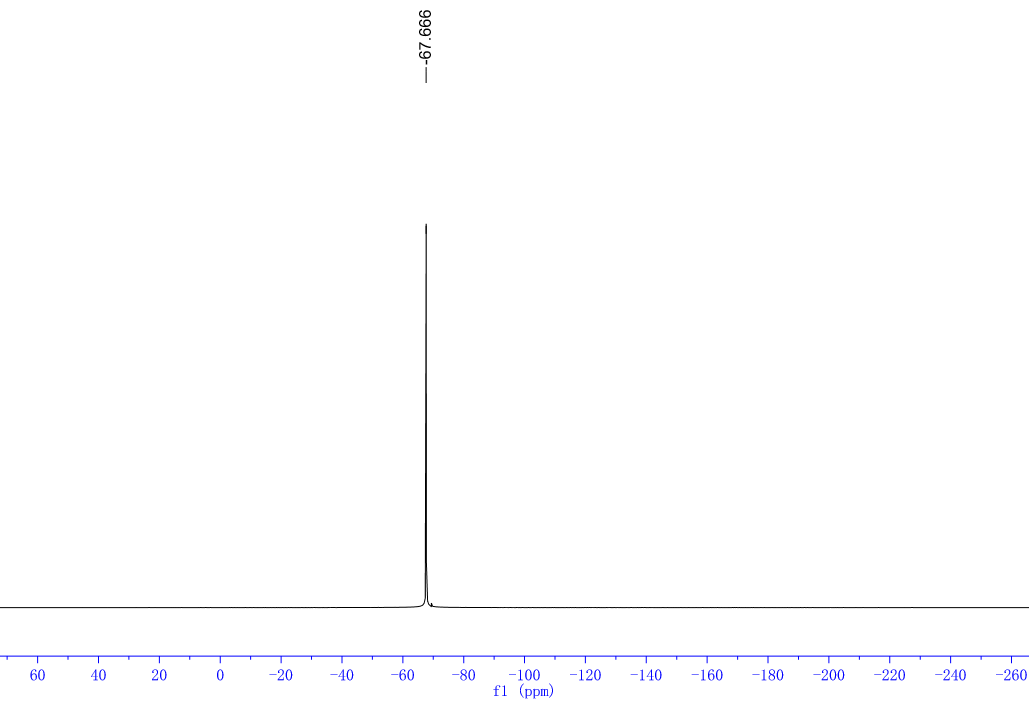


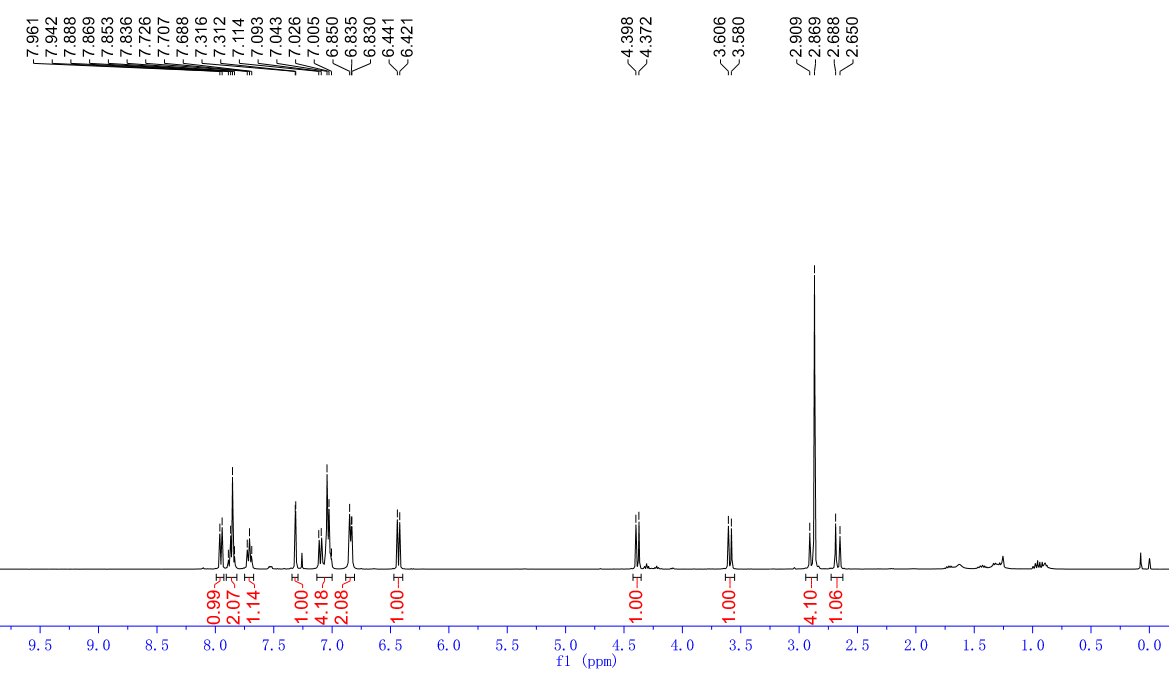

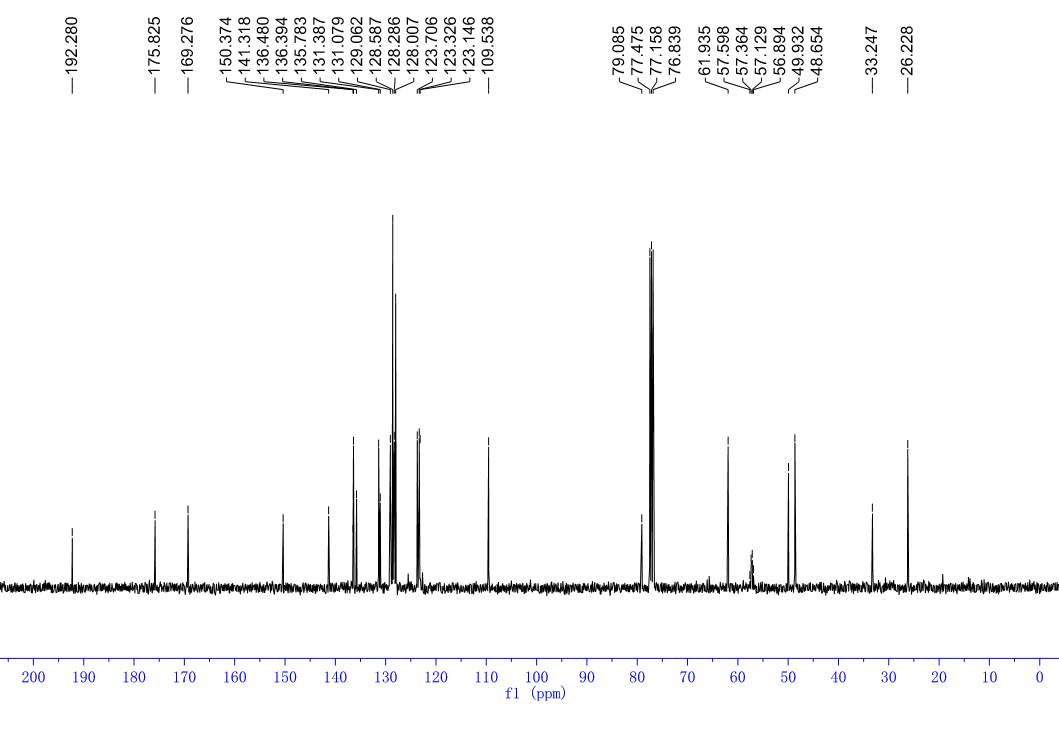

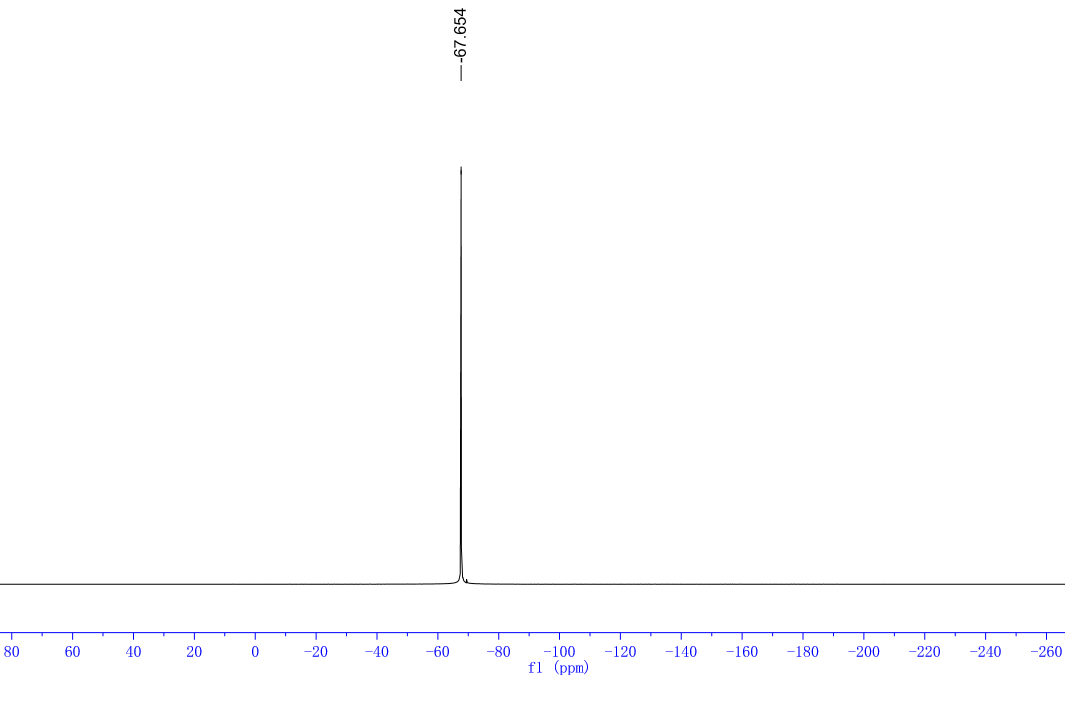


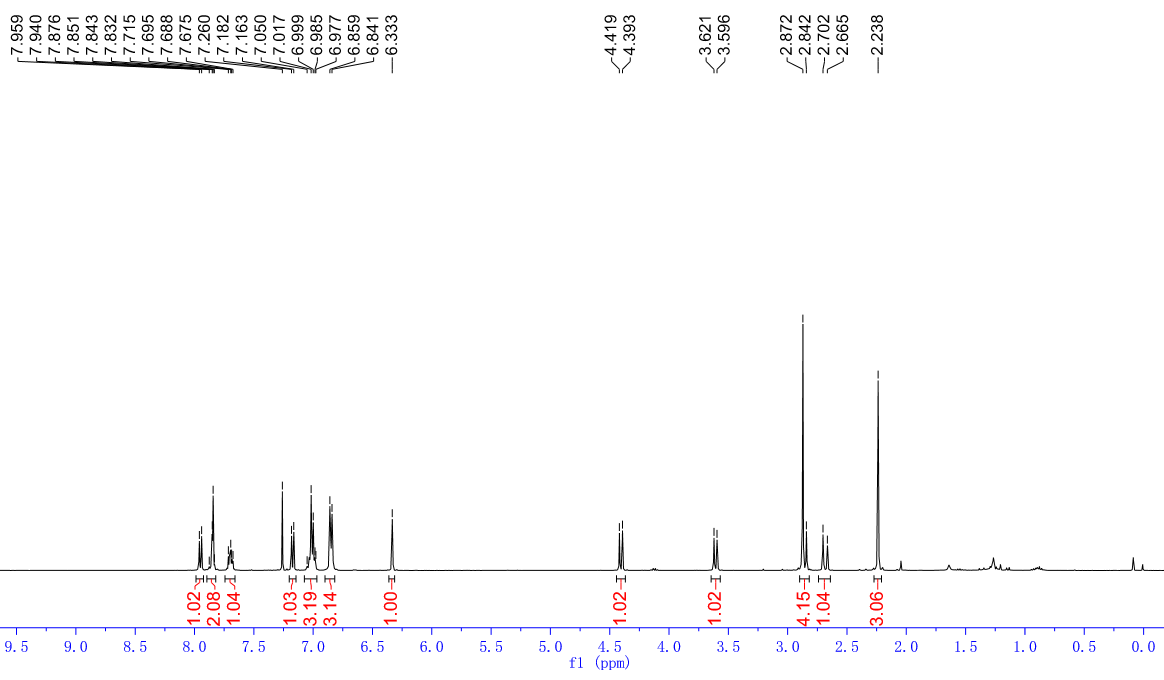

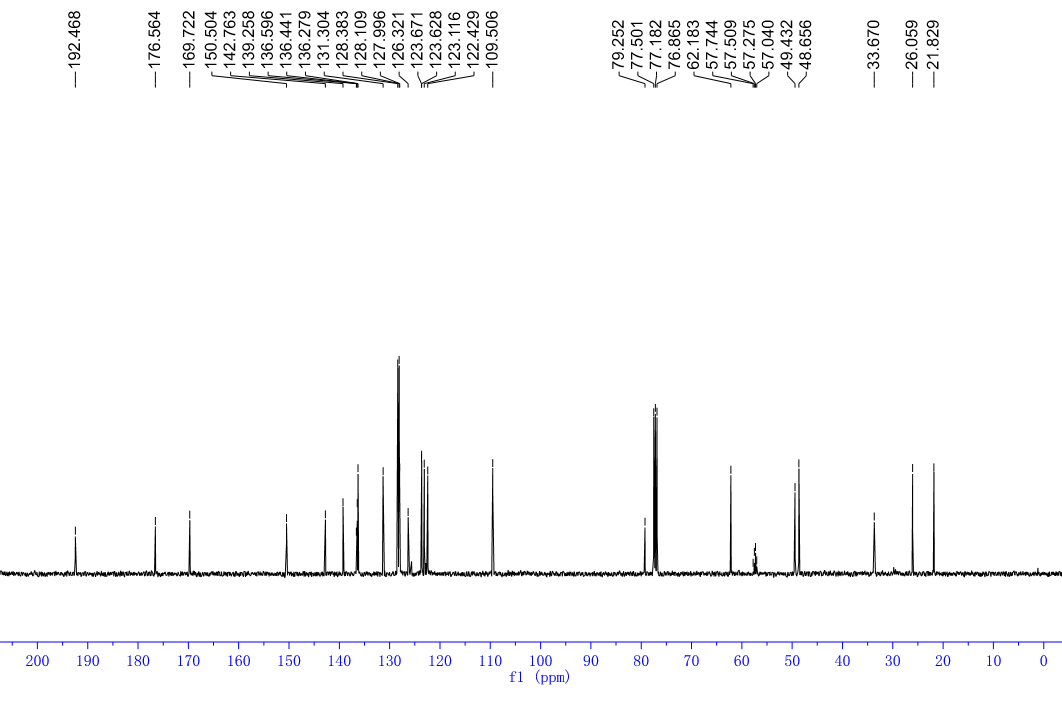

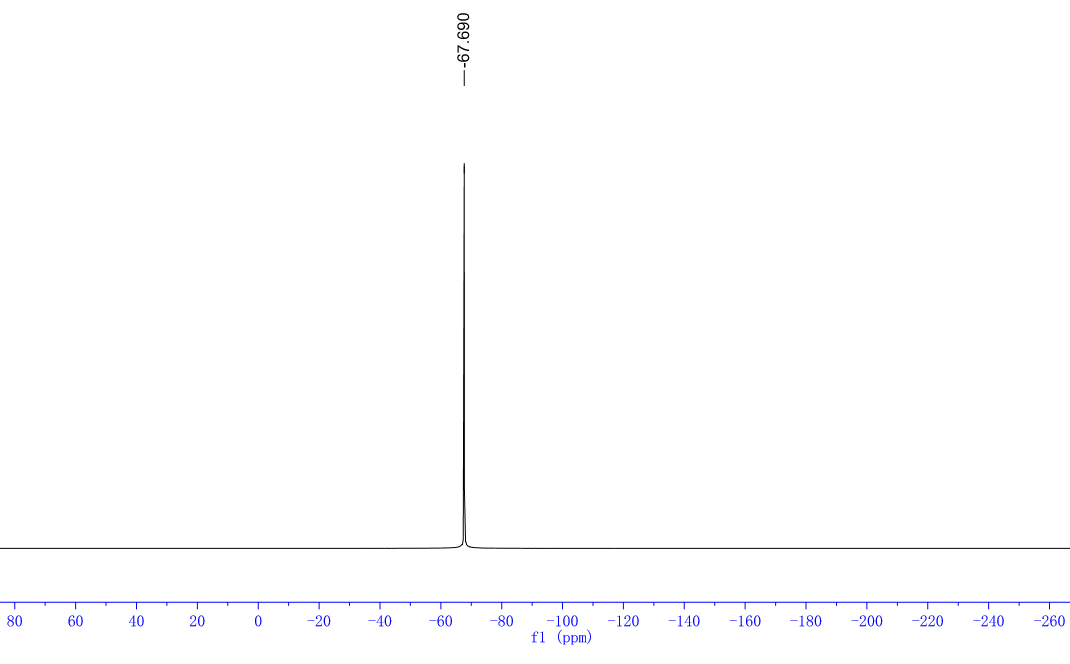


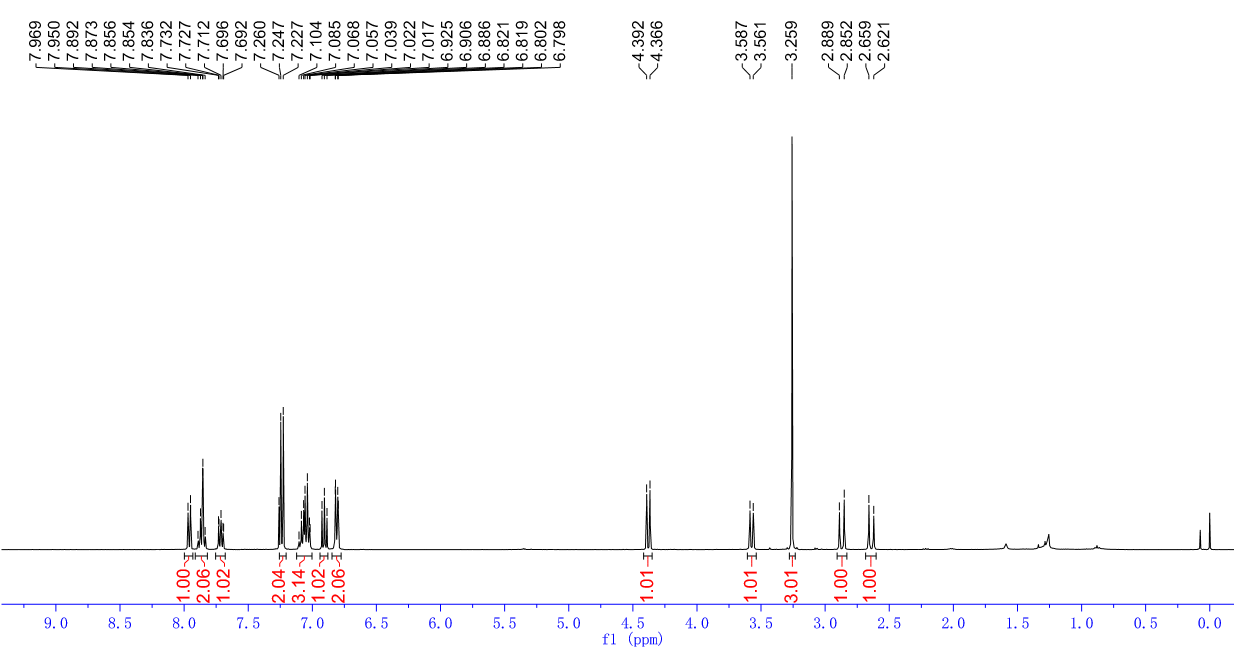

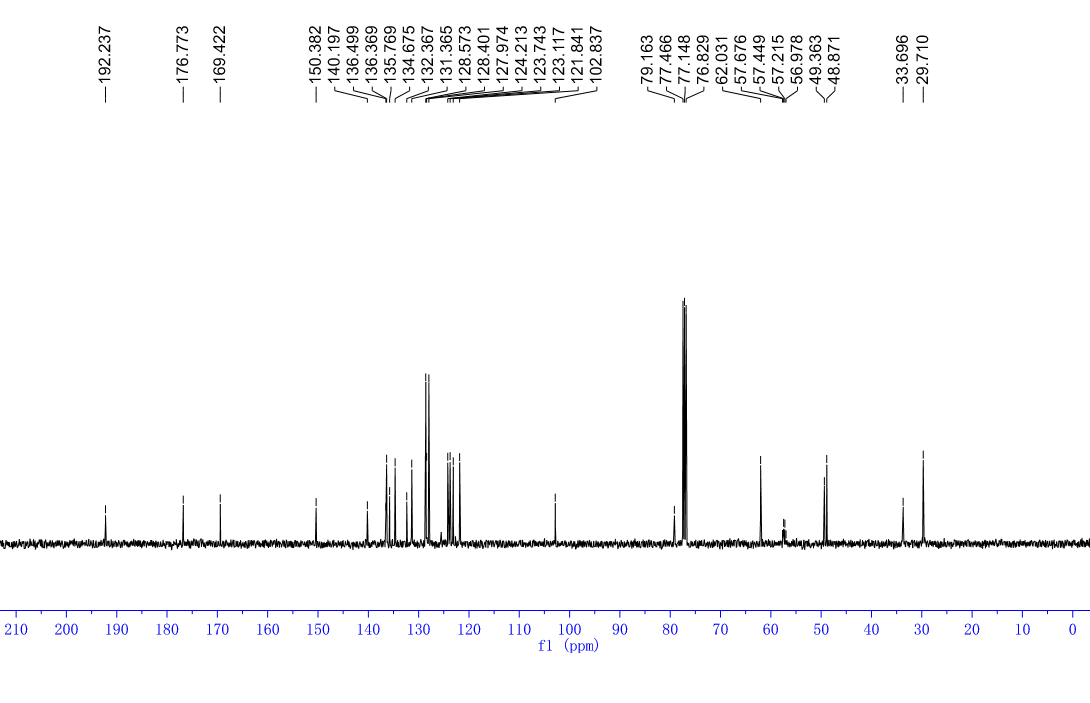

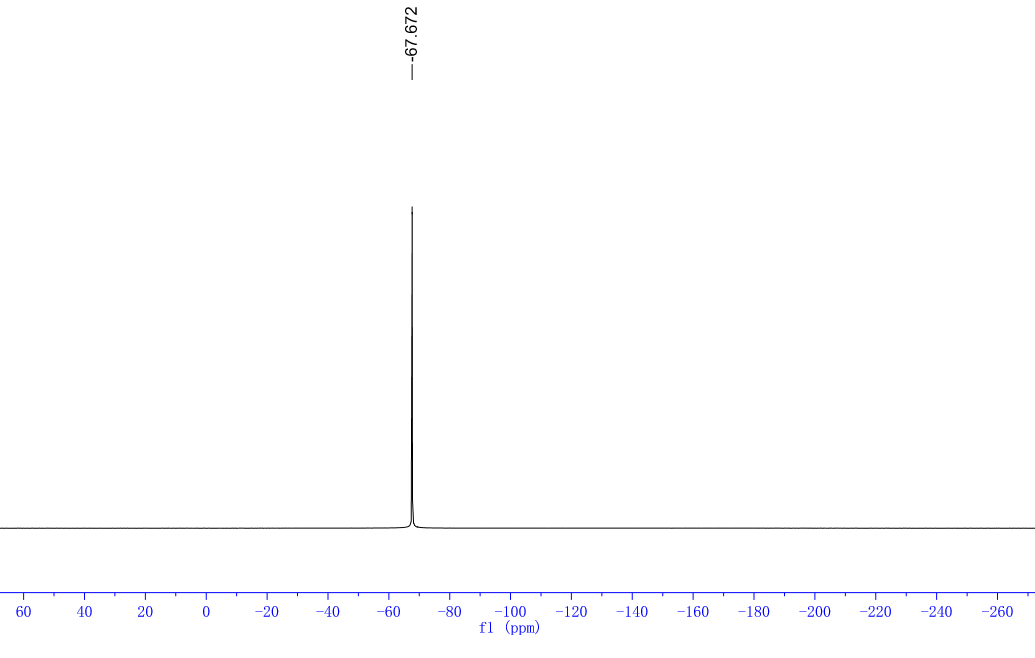


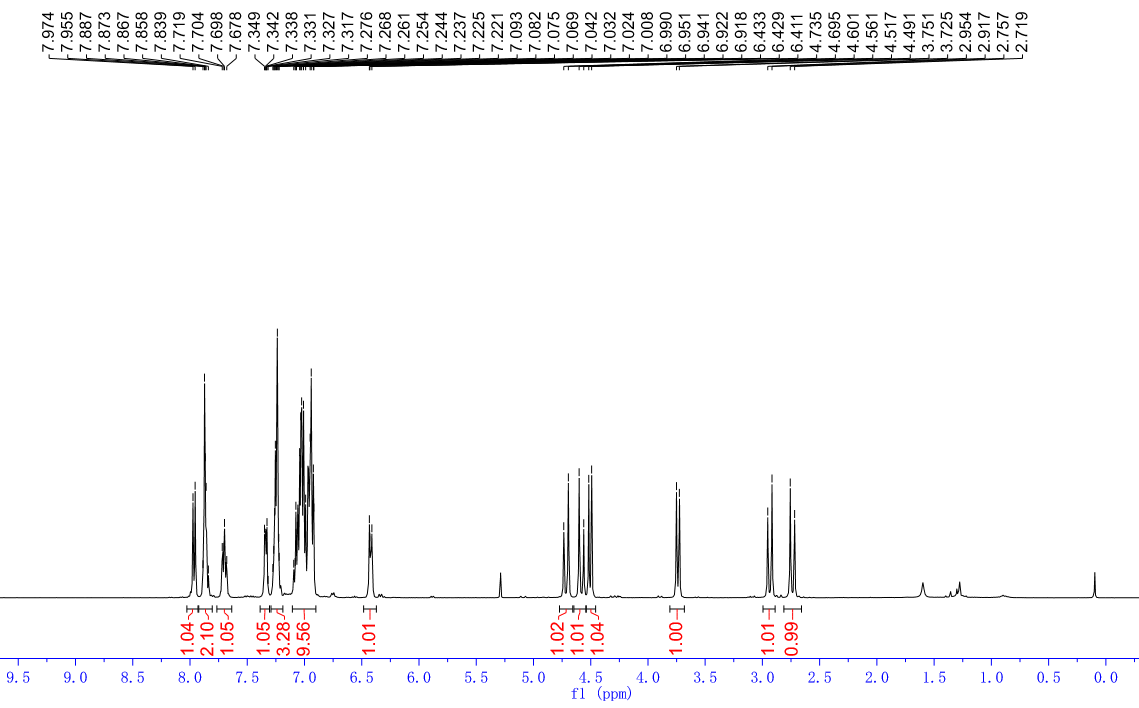

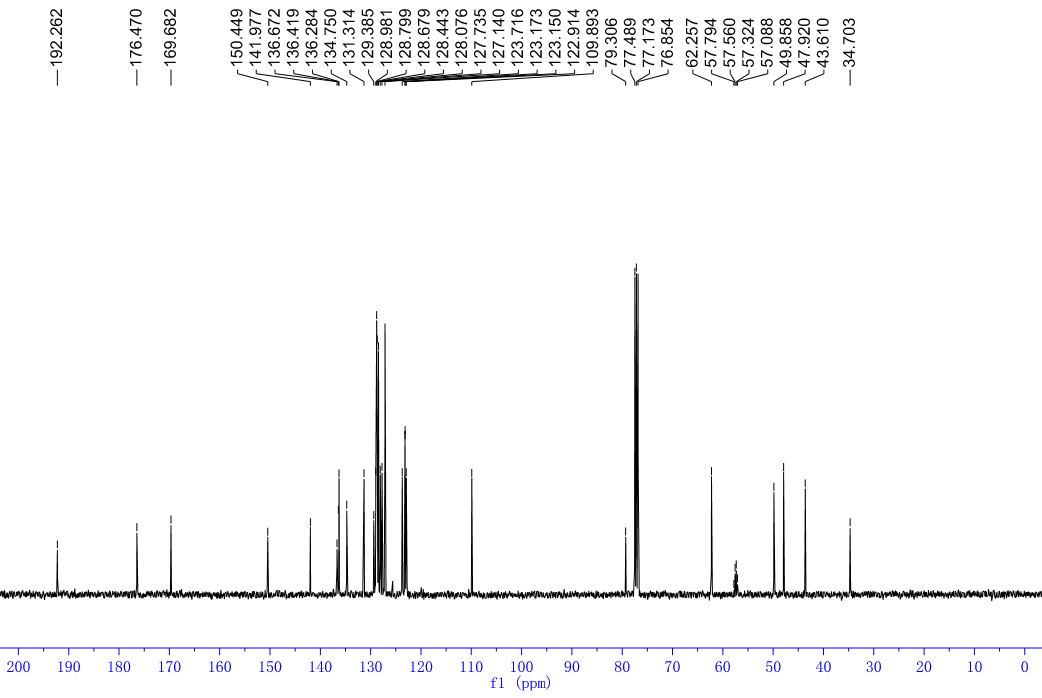

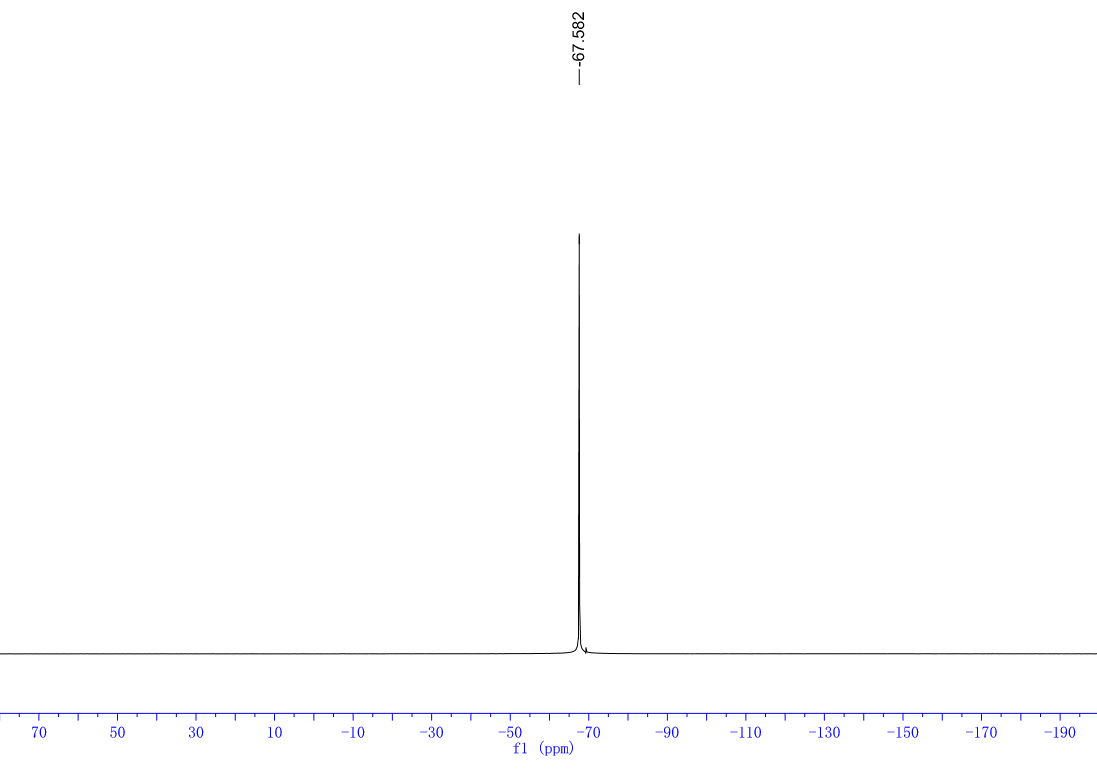


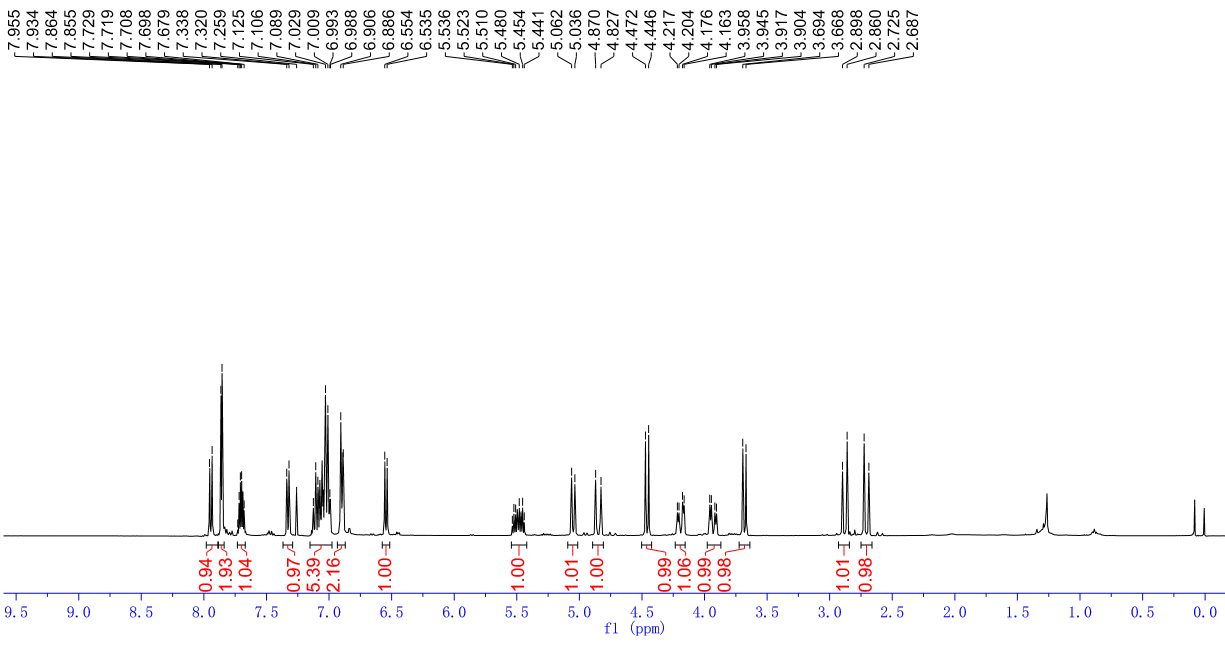

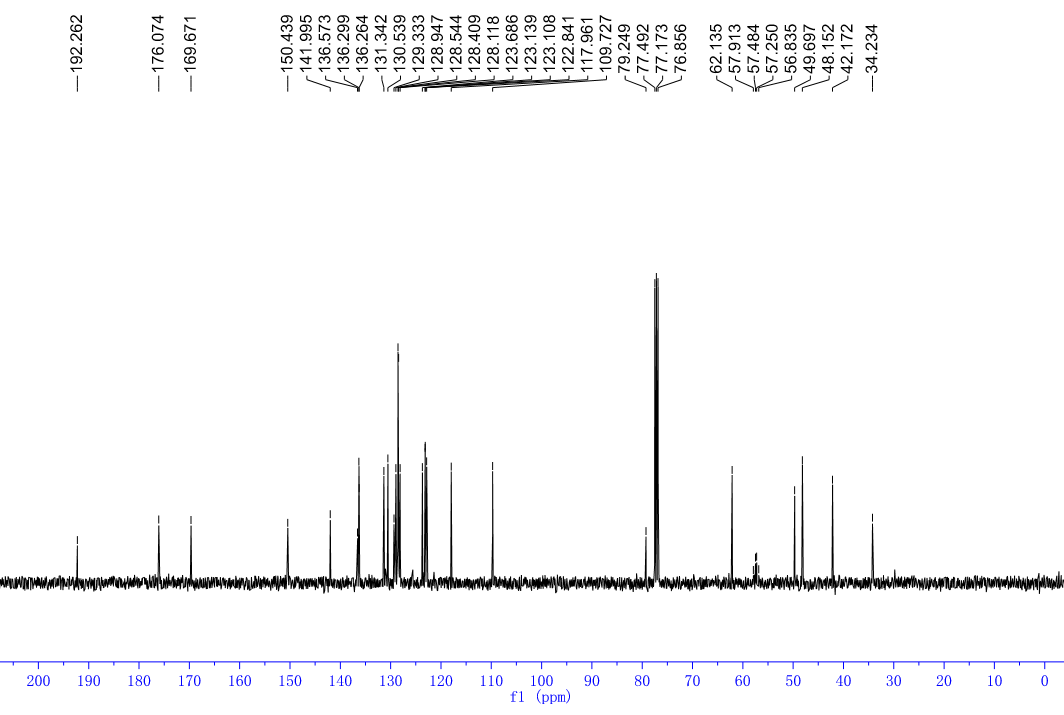

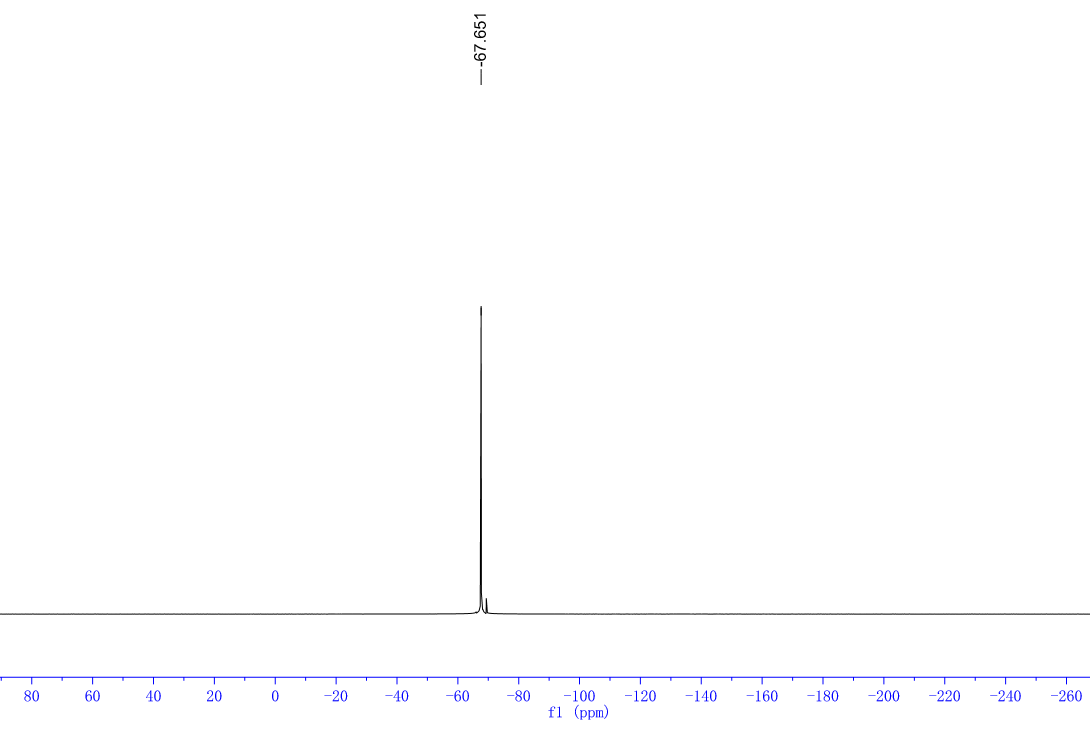


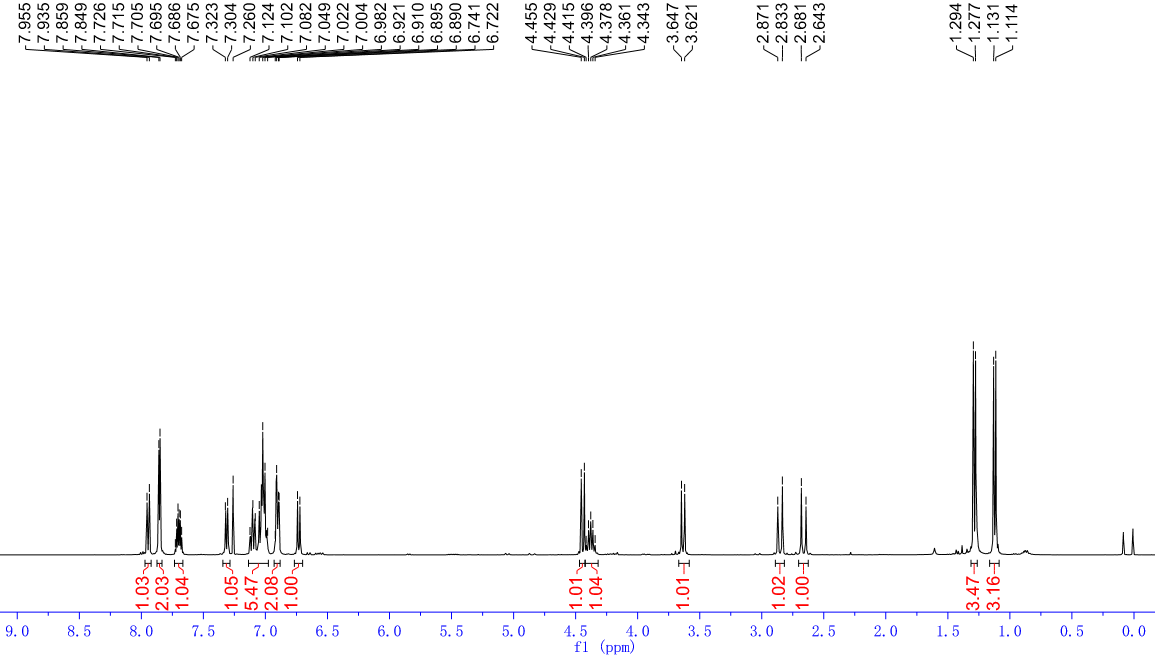


**IV Energies, Cartesian coordinates, and frequencies of all the optimized stationary points**

**Table S1.** Absolute energies, Gibbs free energy corrections (GFEC), and Gibbs free energies (GFE) of the optimized structures obtained at the M062X/6-31G(d, p)/ IEFPCM_mesitylene_ level.

|  | **energies (a.u.)** | **GFEC (a.u.)** | **GFE (a.u.)** |
| --- | --- | --- | --- |
| **NHC** | -3623.092451 | 0.320126 | -3622.772325 |
| **1a** | -2993.977408 | 0.097129 | -2993.880279 |
| **2a** | -1350.065772 | 0.255519 | -1349.810253 |
| **2a^-^** | -1349.539427 | 0.241780 | -1349.297647 |
| **TS_2a→2a-_** | -3852.110716 | 0.376058 | -3851.734658 |
| **OAc-** | -228.462588 | 0.022167 | -228.440421 |
| **HOAc** | -228.994287 | 0.035944 | -228.958343 |
| **HBr** | -2572.370532 | -0.013119 | -2572.383651 |
| **Br-** | -2571.895503 | -0.016176 | -2571.911679 |
| **M1** | -6846.126965 | 0.503360 | -6845.623605 |
| **TS1** | -6617.092814 | 0.447110 | -6616.645704 |
| **M2** | -6617.106143 | 0.446331 | -6616.659812 |
| **TS2** | -6846.113542 | 0.499951 | -6845.613591 |
| **M3** | -6617.114661 | 0.444282 | -6616.670379 |
| **TS3** | -6617.102612 | 0.445600 | -6616.657012 |
| **M4** | -6617.556586 | 0.452726 | -6617.103860 |
| **TS4** | -6846.096342 | 0.496616 | -6845.599726 |
| **M5** | -5394.723139 | 0.725625 | -5393.997514 |
| **TS5RR** | -5394.793612 | 0.715869 | -5394.077743 |
| **TS5RR**_Φ= 51º_ | -5157.050072 | 0.717783 | -5156.332289 |
| **TS5RR**_Φ= 70º_ | -5157.044125 | 0.716877 | -5156.327248 |
| **TS5RR**_Φ= 157º_ | -5157.050084 | 0.71639 | -5156.333694 |
| **TS5RR**_Φ= 152º_ | -5157.051072 | 0.717778 | -5156.333294 |
| **TS5SS** | -5394.794745 | 0.721938 | -5394.072807 |
| **TS5SS**_Φ= -21º_ | -5157.053177 | 0.711426 | -5156.341751 |
| **TS5SS**_Φ= 80º_ | -5157.057081 | 0.714208 | -5156.342873 |
| **TS5SS**_Φ= 152º_ | -5157.052407 | 0.711671 | -5156.340736 |
| **TS5SS**_Φ=135º_ | -5157.057541 | 0.715616 | -5156.341925 |
| **TS5RR'** | -5157.061071 | 0.707748 | -5156.353323 |
| **TS5RR**_Φ= 51º_ | -5157.050072 | 0.717783 | -5156.332289 |
| **TS5RR**_Φ= 70º_ | -5157.044125 | 0.716877 | -5156.327248 |
| **TS5RR**_Φ= 157º_ | -5157.050084 | 0.71639 | -5156.333694 |
| **TS5RR**_Φ= 152º_ | -5157.051072 | 0.717778 | -5156.333294 |
| **TS5SS'** | -5157.057540 | 0.713159 | -5156.344381 |
| **TS5SS'**_Φ= -22º_ | -5394.785253 | 0.721910 | -5394.063343 |
| **TS5SS'**_Φ= 73º_ | -5394.780482 | 0.720712 | -5394.05977 |
| **TS5SS'**_Φ= -82º_ | -5394.782439 | 0.723144 | -5394.059295 |
| **TS5SS'**_Φ= 152º_ | -5394.792123 | 0.721074 | -5394.071049 |
| **M6SS** | -5394.834432 | 0.727599 | -5394.106833 |
| **TS6SS** | -5394.802199 | 0.725850 | -5394.076349 |
| **TS7SS** | -5394.821922 | 0.725633 | -5394.096289 |
| **PSS** | -1771.717785 | 0.377635 | -1771.340150 |

**NHC**

Zero-point correction= 0.374168

Thermal correction to Energy= 0.396504

Thermal correction to Enthalpy= 0.397448

Thermal correction to Gibbs Free Energy= 0.320126

Sum of electronic and zero-point Energies= -3622.718283

Sum of electronic and thermal Energies= -3622.695948

Sum of electronic and thermal Enthalpies= -3622.695003

Sum of electronic and thermal Free Energies= -3622.772325

Cartesian coordinates

C -2.994330 1.470080 0.664213

C -2.284271 0.860182 -0.370580

C -2.517839 -0.459846 -0.736302

C -3.507746 -1.149238 -0.044482

C -4.242522 -0.556010 0.980501

C -3.982277 0.763250 1.341380

C -2.506412 2.876178 0.898410

C -1.743662 3.186924 -0.408963

C -1.271012 1.813063 -0.959129

H -1.940988 -0.938654 -1.519593

H -5.010029 -1.127276 1.489926

H -4.547748 1.226531 2.144008

H -1.839351 2.899752 1.767958

H -2.451985 3.597644 -1.132411

H -3.306799 3.596445 1.082138

H -1.251468 1.793966 -2.051360

N 0.081862 1.531431 -0.481518

O -0.715123 4.148278 -0.319037

C 0.760156 0.358082 -0.692003

C 0.854208 2.429282 0.209506

N 1.919817 0.651993 -0.066732

N 2.009544 1.913461 0.491697

C 0.348796 3.797741 0.544996

H 0.043333 3.844411 1.598434

H 1.138571 4.534553 0.391924

C 3.048803 -0.221741 0.033914

C 2.921001 -1.416078 0.746980

C 4.245413 0.151894 -0.589217

C 4.038525 -2.249444 0.823903

C 5.334423 -0.707902 -0.476158

C 5.250275 -1.912302 0.225383

H 3.956981 -3.184255 1.373311

H 6.271202 -0.434406 -0.957075

Br -3.877910 -2.950960 -0.505261

C 4.347572 1.434892 -1.370841

H 3.493092 1.548501 -2.045045

H 4.348250 2.301501 -0.704909

H 5.263620 1.447737 -1.964375

C 6.449693 -2.819757 0.325636

H 6.802253 -3.114440 -0.667214

H 7.279876 -2.316456 0.830219

H 6.213931 -3.727452 0.884945

C 1.624533 -1.794957 1.413031

H 1.278209 -0.994287 2.074062

H 0.838020 -1.956728 0.671664

H 1.750330 -2.703652 2.004620

Vibrational frequencies

17.5088 26.8377 30.8255

41.2590 53.9403 69.5381

106.9891 135.3234 142.7229

153.8634 167.5797 172.2072

189.6022 198.4903 221.6712

237.8075 261.5488 276.9147

279.3911 303.0451 309.7007

336.2069 343.5335 362.4328

395.3079 429.1005 456.2669

482.7028 497.7169 514.4391

522.6284 533.5611 559.7111

566.3821 588.2188 602.0603

621.9079 636.6502 656.6652

667.6999 689.5373 706.8954

733.9282 759.7921 797.2733

829.3549 847.4236 856.4359

879.6801 900.8517 908.9861

911.5471 937.9290 963.5318

978.6852 992.2235 995.4881

1009.9532 1028.6956 1033.9918

1041.1707 1053.1617 1062.3032

1065.7226 1066.5382 1069.1738

1084.3767 1101.0596 1106.3251

1143.7418 1165.4364 1186.8699

1198.2363 1204.2184 1233.4554

1242.7485 1260.2784 1272.2729

1277.7860 1288.8603 1302.4293

1308.4393 1331.6533 1337.1075

1349.0223 1358.8477 1360.9064

1376.6614 1381.9198 1417.5344

1419.4654 1423.5824 1431.2652

1444.4090 1471.0522 1477.8476

1479.9098 1485.1775 1490.8107

1494.7521 1497.8344 1500.7879

1504.5920 1514.5376 1527.4869

1533.3717 1568.3036 1671.1302

1686.4242 1694.0927 1696.6218

1704.5703 3070.0710 3071.2503

3071.8768 3072.9853 3086.7752

3122.7331 3135.0297 3140.6555

3143.6907 3144.3779 3147.6809

3163.2739 3164.1934 3166.2235

3169.1548 3181.2918 3189.8346

3217.0800 3241.2134 3243.0232

**HOAc**

Zero-point correction= 0.062825

Thermal correction to Energy= 0.067298

Thermal correction to Enthalpy= 0.068243

Thermal correction to Gibbs Free Energy= 0.035944

Sum of electronic and zero-point Energies= -228.931462

Sum of electronic and thermal Energies= -228.926989

Sum of electronic and thermal Enthalpies= -228.926045

Sum of electronic and thermal Free Energies= -228.958344

Cartesian coordinates

C -0.091870 0.122425 -0.000270

O -0.637792 1.197999 0.000065

O -0.778455 -1.035937 0.000023

H -1.720478 -0.804975 0.000238

C 1.391820 -0.113158 0.000034

H 1.670045 -0.694391 0.881649

H 1.670319 -0.695568 -0.880700

H 1.910390 0.842837 -0.000467

Vibrational frequencies

105.5233 429.3961 550.9712

594.1640 672.2584 894.0089

1015.2074 1076.7102 1235.3347

1366.5003 1442.7493 1483.0560

1485.2196 1895.9886 3102.2110

3182.3484 3214.5846 3830.8571

**HBr**

Zero-point correction= 0.006091

Thermal correction to Energy= 0.008451

Thermal correction to Enthalpy= 0.009395

Thermal correction to Gibbs Free Energy= -0.013119

Sum of electronic and zero-point Energies= -2572.364442

Sum of electronic and thermal Energies= -2572.362081

Sum of electronic and thermal Enthalpies= -2572.361137

Sum of electronic and thermal Free Energies= -2572.383652

Cartesian coordinates

Br 0.000000 0.000000 0.039237

H 0.000000 0.000000 -1.373278

Vibrational frequencies

2673.4683

**OAc-**

Zero-point correction= 0.049107

Thermal correction to Energy= 0.053446

Thermal correction to Enthalpy= 0.054390

Thermal correction to Gibbs Free Energy= 0.022167

Sum of electronic and zero-point Energies= -228.413480

Sum of electronic and thermal Energies= -228.409142

Sum of electronic and thermal Enthalpies= -228.408198

Sum of electronic and thermal Free Energies= -228.440421

Cartesian coordinates

C 0.208693 0.000983 -0.006130

O 0.693209 1.157050 0.001271

O 0.807982 -1.098321 0.001448

C -1.346018 -0.055485 -0.001840

H -1.744952 0.599024 -0.783168

H -1.722111 -1.071558 -0.144649

H -1.718517 0.329723 0.953892

Vibrational frequencies

82.1770 421.3227 606.5871

639.5147 899.0284 992.6970

1039.1646 1333.9241 1440.5276

1481.9484 1499.7635 1781.7002

3055.2807 3133.6330 3148.3909

**1a**

Zero-point correction= 0.135410

Thermal correction to Energy= 0.145105

Thermal correction to Enthalpy= 0.146049

Thermal correction to Gibbs Free Energy= 0.097129

Sum of electronic and zero-point Energies= -2993.841997

Sum of electronic and thermal Energies= -2993.832303

Sum of electronic and thermal Enthalpies= -2993.831359

Sum of electronic and thermal Free Energies= -2993.880279

Cartesian coordinates

C 0.046947 1.167806 0.000160

C -1.215459 0.704383 0.000089

H 0.092401 2.258340 0.000214

C 1.368520 0.540636 0.000108

C 2.470481 1.413173 -0.000268

C 1.612802 -0.842874 0.000444

C 3.771254 0.929380 -0.000355

H 2.295783 2.485557 -0.000502

C 2.916583 -1.323353 0.000374

H 0.789833 -1.543117 0.000788

C 3.997886 -0.444948 -0.000037

H 4.606181 1.621953 -0.000660

H 3.088223 -2.394657 0.000647

H 5.012416 -0.830042 -0.000096

Br -1.762195 -1.096118 -0.000155

C -2.333009 1.677054 0.000153

O -3.503858 1.388366 0.000094

H -1.993172 2.731633 0.000265

Vibrational frequencies

10.5129 71.5014 122.8826

179.5849 194.2689 238.2364

333.4079 342.0175 411.0484

454.5233 544.6083 563.8571

622.0325 628.5917 705.8969

711.9122 783.5768 864.0689

870.5276 921.5575 966.8317

1005.5730 1017.7137 1032.7763

1034.9710 1073.9989 1128.1205

1151.2642 1184.8989 1219.9754

1255.0403 1334.6283 1370.3227

1381.2253 1440.5808 1503.9371

1551.0027 1667.7177 1694.5565

1713.7338 1859.6499 3002.1294

3150.6785 3197.1986 3207.5518

3218.2114 3228.2170 3271.1760

**2a**

Zero-point correction= 0.307142

Thermal correction to Energy= 0.329071

Thermal correction to Enthalpy= 0.330015

Thermal correction to Gibbs Free Energy= 0.255519

Sum of electronic and zero-point Energies= -1349.758630

Sum of electronic and thermal Energies= -1349.736701

Sum of electronic and thermal Enthalpies= -1349.735757

Sum of electronic and thermal Free Energies= -1349.810253

Cartesian coordinates

O -1.162998 0.774951 2.286452

O 0.131824 -1.826597 0.160796

O -1.051155 -0.093297 -2.327215

N 2.418005 -1.811021 -0.133688

C 2.577333 -3.165247 -0.611796

H 1.587424 -3.620637 -0.635669

H 3.228052 -3.734973 0.057742

C 1.451286 0.184388 0.643338

C 3.455564 -0.877735 -0.000827

C 2.937960 0.340202 0.458678

C 3.782416 1.416600 0.654952

H 3.395153 2.366710 1.011526

C 1.210715 -1.259463 0.207557

C 0.624426 1.183445 -0.174462

H 0.853307 2.184620 0.201246

H 0.929936 1.148168 -1.225704

C 5.148896 1.261066 0.390831

H 5.824673 2.095886 0.540837

C 5.646195 0.042214 -0.061102

H 6.707684 -0.062927 -0.260422

C -1.506518 0.442644 1.177406

C 4.803684 -1.055145 -0.265902

H 5.191006 -2.004250 -0.620823

C -0.898209 0.978204 -0.136292

C -2.575126 -0.522911 0.816128

C -1.447556 -0.013117 -1.191919

C -3.434086 -1.675603 -1.137104

H -3.394107 -1.877425 -2.202197

C -3.495690 -1.142909 1.650707

H -3.503600 -0.935613 2.715551

C -2.544571 -0.784866 -0.552089

C -4.368319 -2.291536 -0.307469

H -5.083312 -2.990671 -0.728603

C -4.398947 -2.028834 1.068379

H -5.137345 -2.528382 1.686889

H 3.005800 -3.166141 -1.618182

C -1.551899 2.329172 -0.422651

F -1.120923 2.836316 -1.582504

F -1.274030 3.220543 0.536908

F -2.887544 2.224855 -0.497020

H 1.165071 0.263480 1.698730

Vibrational frequencies

30.3860 34.7802 44.7035

60.9166 75.7125 93.5364

118.0018 118.7538 133.1575

140.2762 145.1028 157.3638

164.3056 187.7342 228.5313

239.5427 258.5600 262.7248

303.7255 321.8988 333.1934

351.4092 359.4132 379.0163

420.3208 455.7810 463.7558

494.8448 509.1354 536.0830

548.0282 552.7422 555.5556

566.4622 581.7844 607.4151

649.7907 658.5165 683.4653

712.2126 715.1778 726.0196

751.9427 758.4615 766.0351

777.3922 810.0211 812.5598

866.0969 881.5295 896.7193

917.7661 925.5623 937.5029

968.1825 982.4328 999.1861

1007.4438 1022.0424 1039.4785

1051.1378 1063.1408 1070.1593

1097.6002 1116.7115 1120.1316

1151.6838 1153.5971 1163.1099

1177.2855 1180.8162 1210.2432

1219.9847 1223.9353 1241.3062

1264.3838 1272.3705 1282.1363

1296.6769 1313.1555 1317.0819

1331.9656 1345.7433 1362.5991

1380.8505 1399.5776 1409.8516

1433.6458 1460.4278 1492.9288

1497.7666 1516.6182 1523.2536

1524.6362 1530.7938 1562.2642

1690.5212 1692.9963 1694.0213

1714.0452 1827.1220 1870.4064

1911.3916 3068.5710 3092.9636

3102.9022 3140.8083 3148.8592

3191.1625 3207.1510 3212.8536

3219.1499 3226.6550 3229.2476

3233.1522 3240.5221 3245.3085

**2a^-^**

Zero-point correction= 0.292759

Thermal correction to Energy= 0.314566

Thermal correction to Enthalpy= 0.315510

Thermal correction to Gibbs Free Energy= 0.241780

Sum of electronic and zero-point Energies= -1349.246668

Sum of electronic and thermal Energies= -1349.224860

Sum of electronic and thermal Enthalpies= -1349.223916

Sum of electronic and thermal Free Energies= -1349.297647

Cartesian coordinates

O 0.925718 0.320505 -2.307392

O -0.099416 -1.619494 0.159221

O 1.223954 0.429649 2.384972

N -2.436260 -1.750332 0.065688

C -2.512523 -3.182656 0.058556

H -1.487089 -3.550593 0.124641

H -2.973419 -3.558554 -0.863325

C -1.537223 0.332641 0.092775

C -3.494260 -0.873459 0.016856

C -2.952717 0.451917 0.034944

C -3.851224 1.526515 -0.003104

H -3.480151 2.548636 0.012569

C -1.216805 -1.032463 0.114382

C -0.554790 1.454793 0.129560

H -0.740133 2.155434 -0.697177

H -0.642318 2.038010 1.057900

C -5.223965 1.279245 -0.060699

H -5.914481 2.118289 -0.090487

C -5.727992 -0.024318 -0.080138

H -6.799940 -0.191608 -0.125622

C 1.328288 0.173075 -1.179342

C -4.856948 -1.120418 -0.040905

H -5.236538 -2.139092 -0.055441

C 0.921545 1.039696 0.033030

C 2.380255 -0.777703 -0.715046

C 1.479886 0.227028 1.222641

C 3.374726 -1.539901 1.357630

H 3.426150 -1.509207 2.441199

C 3.197031 -1.606921 -1.467931

H 3.111718 -1.628507 -2.549601

C 2.468015 -0.745782 0.674299

C 4.205828 -2.371974 0.607081

H 4.930364 -3.005568 1.109333

C 4.117376 -2.404976 -0.788478

H 4.774298 -3.063820 -1.348109

H -3.089374 -3.558375 0.912585

C 1.756172 2.316145 -0.048033

F 1.560041 3.112885 1.011983

F 1.469187 3.036722 -1.142236

F 3.078400 2.053626 -0.095432

Vibrational frequencies

27.4873 44.8286 51.9491

56.7277 89.8402 105.2271

118.0816 135.6119 139.0443

148.4686 151.0832 160.5559

191.7778 197.0448 215.7547

238.2615 247.6638 265.1709

276.4526 317.0583 334.9387

348.1814 358.5762 373.7354

424.5276 462.3485 469.6634

498.3689 522.4368 536.6151

554.0947 556.0085 556.4871

580.3252 592.6388 595.6861

656.9355 667.3041 675.3421

712.7382 716.7292 725.2086

729.9201 744.2457 746.5104

755.0463 797.7613 799.4216

841.6689 850.9352 873.2581

895.3509 914.3303 924.4495

944.5495 951.0916 980.7361

998.1138 1017.4376 1025.5207

1051.3708 1052.1304 1064.8483

1093.8213 1109.9621 1116.2085

1149.8818 1155.0349 1166.5767

1167.6110 1203.7299 1211.7606

1252.9303 1256.5430 1273.9089

1289.8077 1296.6203 1305.2280

1313.8762 1328.5738 1346.2443

1376.8540 1388.7600 1419.2095

1425.0312 1448.2340 1485.1299

1497.3180 1507.0124 1519.4421

1523.4301 1525.6587 1533.9731

1559.4618 1645.0492 1669.9648

1691.9927 1704.0626 1716.9304

1869.7086 1906.8431 3029.5708

3036.4278 3061.6850 3091.7118

3167.6221 3170.9604 3182.3602

3189.6566 3198.0528 3205.8851

3211.8382 3224.3830 3227.9205

**Br^-^**

Zero-point correction= 0.000000

Thermal correction to Energy= 0.001416

Thermal correction to Enthalpy= 0.002360

Thermal correction to Gibbs Free Energy= -0.016176

Sum of electronic and zero-point Energies= -2571.895503

Sum of electronic and thermal Energies= -2571.894086

Sum of electronic and thermal Enthalpies= -2571.893142

Sum of electronic and thermal Free Energies= -2571.911678

Cartesian coordinates

Br 0.000000 0.000000 0.000000

Vibrational frequencies

**TS_2a→2a-_**

Zero-point correction= 0.434422

Thermal correction to Energy= 0.461079

Thermal correction to Enthalpy= 0.462023

Thermal correction to Gibbs Free Energy= 0.376058

Sum of electronic and zero-point Energies= -3851.676293

Sum of electronic and thermal Energies= -3851.649637

Sum of electronic and thermal Enthalpies= -3851.648693

Sum of electronic and thermal Free Energies= -3851.734658

Cartesian coordinates

C -3.140094 1.495528 0.667074

C -2.433634 0.967448 -0.413669

C -2.438057 -0.389055 -0.705077

C -3.175223 -1.215632 0.132901

C -3.912844 -0.713981 1.202531

C -3.899272 0.653179 1.471694

C -2.883913 2.974534 0.814744

C -2.249003 3.350702 -0.543676

C -1.624354 2.041146 -1.094420

H -1.844365 -0.811274 -1.510311

H -4.480839 -1.393266 1.827921

H -4.462021 1.044019 2.313754

H -2.192153 3.152681 1.646357

H -3.043222 3.641197 -1.235070

H -3.784051 3.560829 1.012857

H -1.638324 1.995518 -2.185309

N -0.219090 1.972874 -0.660658

O -1.351231 4.441742 -0.544292

C 0.602681 0.909609 -0.759781

C 0.450702 2.970675 -0.003551

N 1.717483 1.306925 -0.158863

N 1.652509 2.588968 0.317354

C -0.211200 4.285005 0.274307

H -0.460225 4.356717 1.340619

H 0.470679 5.101471 0.031456

C 2.883192 0.493760 0.051985

C 3.147196 0.053289 1.352818

C 3.674131 0.155050 -1.045245

C 4.287830 -0.720195 1.542081

C 4.802721 -0.628813 -0.801939

C 5.127597 -1.066251 0.480210

H 4.519329 -1.075299 2.543462

H 5.439205 -0.903363 -1.639166

Br -3.139903 -3.078315 -0.185143

C 3.305368 0.580113 -2.441694

H 2.451410 -0.005857 -2.798050

H 3.030076 1.637953 -2.482232

H 4.140029 0.414263 -3.124691

C 6.343443 -1.922324 0.723136

H 6.051367 -2.951096 0.955861

H 6.992880 -1.947313 -0.154255

H 6.924215 -1.548035 1.570542

C 2.196531 0.356518 2.478126

H 2.070113 1.433508 2.617379

H 1.221372 -0.084675 2.243067

H 2.562416 -0.074668 3.411496

C 0.273418 -2.147285 -0.333638

O 0.116750 -1.542329 0.735130

O 0.388033 -1.579889 -1.487481

H 0.437192 -0.242597 -1.190040

C 0.369744 -3.666230 -0.344132

H -0.142570 -4.071116 -1.218620

H 1.424721 -3.950547 -0.408706

H -0.057122 -4.076325 0.571265

Vibrational frequencies

-606.1878 -8.1452 22.7845

25.0803 31.2735 43.2217

57.8240 65.3069 81.1363

90.5168 94.5838 117.4887

120.6742 143.9662 149.2824

157.6970 168.3199 182.5836

197.3546 205.8138 217.5847

226.0230 242.6180 256.0805

280.8184 283.7799 302.4458

306.5590 319.7701 336.3001

352.0422 383.5305 399.8193

431.5381 456.9196 481.3038

491.9229 502.6778 510.6571

524.8960 537.9408 564.6550

570.8113 589.0267 599.6714

608.2883 627.3880 634.3330

658.4798 670.7870 692.5230

710.1875 722.0017 734.9479

754.6600 804.9497 835.9336

847.9212 853.0541 882.2274

898.8508 908.5450 913.3584

941.2719 962.6308 967.5729

984.6918 988.2894 1005.4716

1024.8072 1026.8985 1036.8181

1045.4563 1051.2910 1062.3693

1063.1330 1065.9737 1071.4389

1074.2591 1078.5865 1101.3461

1112.4494 1122.0786 1148.6233

1171.8125 1195.0896 1196.6818

1208.0862 1245.3309 1254.9909

1267.2143 1276.2813 1284.0186

1307.7462 1311.8867 1331.4350

1338.8070 1340.3234 1357.3341

1362.5580 1366.8645 1370.4806

1379.2053 1386.7973 1416.6766

1417.7486 1419.0451 1430.5232

1445.4090 1458.2899 1470.6317

1475.5585 1479.7785 1481.4355

1488.3167 1494.1197 1496.9806

1497.9872 1498.5092 1506.5463

1510.0561 1510.7477 1528.8453

1536.3773 1541.2444 1560.6495

1655.6502 1672.5585 1674.0544

1693.1661 1698.2884 1702.2815

1791.3800 3065.8770 3069.3649

3072.4374 3075.5544 3076.9182

3085.4500 3131.6208 3137.9042

3140.8799 3142.7517 3145.8660

3146.6173 3156.2923 3161.0751

3164.5479 3168.0040 3172.2748

3190.0482 3193.7559 3203.2489

3205.8610 3222.2342 3236.8355

**M1**

Zero-point correction= 0.576548

Thermal correction to Energy= 0.614276

Thermal correction to Enthalpy= 0.615221

Thermal correction to Gibbs Free Energy= 0.503360

Sum of electronic and zero-point Energies= -6845.550417

Sum of electronic and thermal Energies= -6845.512688

Sum of electronic and thermal Enthalpies= -6845.511744

Sum of electronic and thermal Free Energies= -6845.623605

Cartesian coordinates

C -3.093107 -2.780935 1.019816

C -2.131337 -1.929329 0.488427

C -1.249605 -2.327848 -0.511822

C -1.359331 -3.637641 -0.958498

C -2.329160 -4.510424 -0.461937

C -3.206898 -4.079953 0.528918

C -3.864188 -2.104610 2.125334

C -3.058676 -0.822698 2.455243

C -2.108843 -0.607796 1.222982

H -0.496442 -1.656951 -0.926204

H -2.380622 -5.524616 -0.841360

H -3.950319 -4.763416 0.926898

H -3.967953 -2.720340 3.024135

H -3.709452 0.042865 2.579181

H -4.874180 -1.840001 1.796121

H -2.442084 0.229350 0.611851

N -0.729714 -0.352781 1.661617

O -2.362546 -0.912431 3.687259

C 0.323248 0.149926 0.986888

C -0.226007 -1.037569 2.738353

N 1.394441 -0.231441 1.679591

N 1.069466 -0.967200 2.782072

C -1.198504 -1.711304 3.651317

H -1.407976 -2.730286 3.295306

H -0.793168 -1.766219 4.661708

C 2.775615 -0.051994 1.306231

C 3.376744 -1.087748 0.580024

C 3.435172 1.114190 1.683776

C 4.712543 -0.920681 0.237007

C 4.773545 1.231261 1.306794

C 5.422136 0.231274 0.587025

H 5.207085 -1.699004 -0.338288

H 5.311373 2.139005 1.568985

C -1.090812 2.896142 -0.164734

C -0.840042 1.681452 -0.663852

H -0.280146 3.274175 0.461041

C -2.209937 3.838129 -0.317953

C -1.954062 5.179024 0.008126

C -3.506407 3.487941 -0.722905

C -2.946257 6.145678 -0.092567

H -0.956474 5.461327 0.332941

C -4.500883 4.456054 -0.813234

H -3.740819 2.461354 -0.971008

C -4.226873 5.786308 -0.505624

H -2.720362 7.178091 0.153691

H -5.498651 4.166173 -1.126889

H -5.006694 6.536797 -0.585057

Br -1.947159 0.791609 -1.918012

C 0.435649 0.930863 -0.312430

O 0.889179 0.078744 -1.292493

H 1.859739 0.487010 -1.602057

Br -0.119324 -4.264107 -2.241585

C 2.735046 2.242291 2.388207

H 1.896841 1.897470 2.998743

H 3.431172 2.778835 3.036171

H 2.369450 2.943877 1.629572

C 6.871135 0.378125 0.201883

H 7.518090 -0.136022 0.920412

H 7.059440 -0.057323 -0.782711

H 7.167260 1.429407 0.176037

C 2.592972 -2.295261 0.141528

H 2.016108 -2.727802 0.965269

H 1.893862 -2.004085 -0.649978

H 3.260346 -3.063971 -0.250915

C 3.377337 2.143771 -1.424536

O 3.066687 0.990325 -1.877170

O 2.655887 2.884423 -0.730965

H 1.188462 1.719367 -0.113665

C 4.762875 2.635256 -1.825344

H 4.693617 3.136960 -2.795626

H 5.137448 3.351982 -1.092551

H 5.450438 1.793527 -1.926530

Vibrational frequencies

13.3070 18.9877 21.5709

28.6342 35.6468 42.1686

45.2019 57.7178 60.5786

71.7777 86.0771 92.0539

96.6101 100.6933 107.5346

113.4428 118.1011 122.9390

134.7707 141.9808 146.2114

147.8343 162.0786 165.5403

171.6283 178.3583 192.5626

208.9199 211.7727 223.9623

239.3455 244.9994 268.2432

281.5149 286.4107 290.7925

295.2899 301.8817 326.2177

336.7794 352.4250 370.7184

382.0202 416.3899 418.6706

441.4025 449.0012 465.9882

482.8734 488.4422 505.0840

511.7506 517.9006 521.8830

526.7886 540.5392 560.0348

570.4539 575.5118 591.4352

609.7765 614.8385 617.4896

620.5296 624.4855 642.6699

664.5425 682.2883 696.5365

710.3646 714.8203 721.7592

744.0906 753.4441 765.2522

780.8378 794.4416 823.9712

842.2678 852.0840 865.8355

873.2971 876.6899 877.6251

897.8889 901.4651 909.7448

915.7742 949.8639 960.5050

961.9954 966.8708 977.3916

980.2433 1004.6468 1006.9276

1017.6608 1022.8250 1029.1488

1030.1423 1040.9309 1044.6963

1050.5400 1056.7304 1058.0896

1062.0926 1063.5823 1064.3236

1065.8520 1069.1245 1074.3052

1099.4276 1128.9372 1130.6456

1135.6612 1152.5401 1154.3984

1173.8363 1182.7310 1198.1595

1204.2587 1210.6012 1217.2119

1224.3910 1232.2035 1249.8892

1254.0959 1265.5940 1281.1332

1287.1062 1307.3026 1314.2875

1321.1414 1327.4645 1331.1516

1336.1687 1341.7895 1354.4381

1360.8586 1365.4146 1366.8704

1369.0720 1376.4095 1379.6163

1400.3427 1411.7183 1416.2907

1419.6661 1427.7656 1456.0227

1460.5669 1470.4526 1476.0209

1480.3779 1482.6985 1488.4921

1491.8744 1492.7124 1497.4880

1500.0169 1502.2350 1503.8645

1509.8708 1525.5795 1526.4837

1544.9417 1548.1761 1553.2058

1587.1302 1593.5516 1669.8721

1676.8461 1690.6831 1692.9949

1694.3814 1695.3669 1697.4450

1702.0292 1741.2144 1933.8465

2944.9827 3066.0916 3066.9115

3068.1673 3068.8192 3076.0367

3084.8122 3132.7875 3138.5636

3141.5458 3146.9669 3148.5764

3155.3810 3160.4699 3162.9431

3168.7270 3170.4478 3176.4022

3179.8420 3183.3488 3197.6345

3198.4271 3201.0433 3202.4628

3208.6573 3223.5296 3223.5363

3236.5820 3238.2377 3273.5645

**TS1**

Zero-point correction= 0.512258

Thermal correction to Energy= 0.544227

Thermal correction to Enthalpy= 0.545171

Thermal correction to Gibbs Free Energy= 0.447110

Sum of electronic and zero-point Energies= -6616.580556

Sum of electronic and thermal Energies= -6616.548587

Sum of electronic and thermal Enthalpies= -6616.547643

Sum of electronic and thermal Free Energies= -6616.645703

Cartesian coordinates

C -2.540850 0.283519 -1.775531

C -1.255297 0.506080 -1.489098

H -2.669030 -0.501926 -2.523164

C -3.815724 0.838917 -1.301913

C -4.976320 0.356183 -1.927777

C -3.965027 1.773486 -0.266172

C -6.239660 0.794810 -1.551829

H -4.877189 -0.377288 -2.723848

C -5.230932 2.208200 0.112683

H -3.097463 2.152118 0.255882

C -6.371464 1.729373 -0.527243

H -7.118909 0.408206 -2.057430

H -5.325038 2.931137 0.917180

H -7.354572 2.076997 -0.225959

Br -0.591135 1.788297 -0.273138

C -0.144446 -0.294881 -2.138296

H -0.585488 -1.117820 -2.739253

O 0.926894 0.237918 -2.472149

C 4.872568 -0.776474 -0.425888

C 3.535387 -0.454704 -0.631493

C 3.024639 0.801556 -0.334792

C 3.902215 1.731464 0.204073

C 5.249089 1.441297 0.420975

C 5.740829 0.179089 0.096949

C 5.151030 -2.199857 -0.837199

C 3.755042 -2.843324 -1.025468

C 2.755292 -1.636499 -1.162209

H 1.984768 1.040087 -0.526957

H 5.901469 2.196742 0.843985

H 6.787835 -0.052527 0.267122

H 5.724917 -2.766601 -0.097667

H 3.717292 -3.469291 -1.917611

H 5.712444 -2.229708 -1.777059

H 2.433703 -1.468822 -2.190854

N 1.555679 -1.866962 -0.356016

O 3.432201 -3.730126 0.036673

C 0.278704 -1.458041 -0.545642

C 1.657129 -2.472581 0.869663

N -0.318230 -1.848303 0.589476

N 0.512897 -2.489511 1.480383

C 2.965758 -3.100706 1.217623

H 3.678340 -2.351485 1.589666

H 2.829992 -3.871098 1.976513

C -1.688584 -1.616245 0.942793

C -2.686883 -2.295995 0.239815

C -1.966877 -0.701779 1.960583

C -4.010824 -2.019693 0.571450

C -3.309421 -0.462029 2.255847

C -4.340334 -1.106429 1.573835

H -4.806649 -2.523529 0.027610

H -3.552855 0.257133 3.034687

Br 3.229319 3.441840 0.674378

C -0.865763 -0.023256 2.731356

H -1.259797 0.839403 3.272596

H -0.420174 -0.713791 3.453190

H -0.072316 0.323275 2.065635

C -5.782329 -0.839575 1.916598

H -5.896638 0.125960 2.414535

H -6.399429 -0.830821 1.014710

H -6.171775 -1.613739 2.586345

C -2.342531 -3.271754 -0.853586

H -1.578756 -3.982435 -0.524325

H -3.228252 -3.831240 -1.159646

H -1.944550 -2.753538 -1.733007

Vibrational frequencies

-177.0980 13.3200 20.7249

23.9752 35.1669 46.4279

50.6976 60.7408 71.3903

89.1023 95.6974 104.5964

113.1759 129.4052 141.5129

145.7032 158.2210 166.3328

168.2924 179.0234 186.3170

198.2677 209.7343 219.5556

231.6441 240.8953 252.5516

270.6381 275.2947 281.7635

285.1954 295.7726 312.6084

332.1278 353.2188 366.9489

375.4673 409.0113 416.5186

419.1246 443.7758 456.4075

500.1662 510.0730 516.9499

520.0444 524.4683 536.2274

554.4378 558.5483 574.2009

587.6261 591.0524 608.3461

616.3943 619.5964 630.7148

635.0329 659.2924 696.8218

707.1958 717.4377 733.4955

740.1007 750.0682 759.6937

782.3510 794.3122 840.8357

846.4767 854.3033 864.5045

864.7722 882.5531 888.9056

902.6606 915.4650 922.2037

951.9021 956.5274 966.6255

980.0178 981.3960 994.2830

999.3575 1007.5673 1016.8489

1020.9123 1027.7423 1039.7427

1042.4576 1047.9506 1055.8904

1059.9406 1064.3955 1066.5475

1070.5897 1075.1677 1085.7215

1097.6988 1114.6488 1121.3467

1131.5809 1132.5321 1149.8607

1179.9474 1193.5062 1201.9543

1215.2260 1219.3560 1228.0788

1246.6871 1248.9411 1265.3448

1280.8445 1282.8766 1297.7674

1305.2644 1307.8188 1325.2293

1328.1961 1341.3662 1346.6088

1356.8186 1367.0839 1367.2319

1370.7343 1383.6516 1412.1909

1419.2676 1420.1327 1422.9651

1431.9593 1434.2108 1464.4801

1470.3369 1475.2595 1481.9328

1484.4208 1492.2170 1497.1983

1499.1225 1501.5321 1503.0179

1505.7521 1509.0517 1524.0863

1538.3117 1552.9263 1567.1348

1635.6185 1669.0588 1676.6341

1689.2861 1695.6946 1697.4640

1698.1154 1703.7127 1746.3475

2958.3771 3065.8685 3066.6926

3067.5587 3078.8807 3079.5755

3128.0640 3134.1234 3141.2024

3153.4596 3157.4197 3157.5813

3168.3018 3168.4111 3170.0027

3172.1047 3184.3049 3185.6674

3195.6221 3204.8151 3205.1680

3212.7276 3213.0666 3223.9228

3234.7585 3262.3185 3272.2188

**M2**

Zero-point correction= 0.512920

Thermal correction to Energy= 0.545737

Thermal correction to Enthalpy= 0.546681

Thermal correction to Gibbs Free Energy= 0.446331

Sum of electronic and zero-point Energies= -6616.593222

Sum of electronic and thermal Energies= -6616.560406

Sum of electronic and thermal Enthalpies= -6616.559461

Sum of electronic and thermal Free Energies= -6616.659812

Cartesian coordinates

C 0.091368 0.243877 3.341781

C 0.687372 0.202561 2.078487

C 0.816308 1.352245 1.311510

C 0.270449 2.529413 1.808086

C -0.337517 2.592820 3.057807

C -0.410132 1.441343 3.840582

C 0.074759 -1.119127 3.980049

C 1.029635 -1.936585 3.076777

C 1.044781 -1.217034 1.696605

H 1.286877 1.351032 0.339405

H -0.749495 3.531101 3.411915

H -0.869455 1.484516 4.823415

H -0.940694 -1.530620 3.973960

H 2.042583 -1.877937 3.481432

H 0.412998 -1.113026 5.019057

H 2.008407 -1.310372 1.189516

N -0.008912 -1.826455 0.851555

O 0.743210 -3.312620 2.978110

C -0.651758 -1.215396 -0.234646

C -0.965388 -2.642983 1.439075

N -1.974305 -1.622401 -0.124706

N -2.127431 -2.539496 0.904559

C -0.566681 -3.592150 2.529574

H -1.300585 -3.559357 3.343732

H -0.545671 -4.611527 2.135373

C -3.149731 -1.028736 -0.673031

C -3.437456 0.318858 -0.415268

C -4.012708 -1.842837 -1.414350

C -4.616049 0.841660 -0.944840

C -5.186118 -1.276668 -1.909472

C -5.500791 0.064473 -1.692415

H -4.854146 1.886062 -0.755242

H -5.866207 -1.899077 -2.486792

C 2.257415 -1.228792 -1.257689

C 1.304516 -0.299538 -1.475492

H 1.846542 -2.206835 -1.003222

C 3.722709 -1.195549 -1.309136

C 4.377731 -2.434179 -1.422445

C 4.514976 -0.039743 -1.208272

C 5.762736 -2.519429 -1.467692

H 3.780649 -3.340277 -1.482425

C 5.902069 -0.129014 -1.248875

H 4.050249 0.930719 -1.095524

C 6.534375 -1.362714 -1.383800

H 6.239655 -3.489502 -1.565455

H 6.493798 0.777483 -1.167834

H 7.617361 -1.422485 -1.416546

Br 1.726025 1.434420 -2.220940

C -0.109357 -0.470761 -1.247646

O -1.004179 0.160394 -2.092985

H -0.740256 -0.012412 -3.005450

Br 0.295285 4.057035 0.692717

C -3.676436 -3.287632 -1.669536

H -2.659022 -3.384804 -2.060624

H -3.718993 -3.869598 -0.744763

H -4.371318 -3.722548 -2.390459

C -6.755364 0.668017 -2.270079

H -6.555588 1.123898 -3.245521

H -7.530159 -0.089120 -2.411650

H -7.153454 1.449573 -1.618167

C -2.514199 1.184417 0.399292

H -2.119397 0.645059 1.268405

H -1.657313 1.518008 -0.197228

H -3.040030 2.072496 0.757996

Vibrational frequencies

15.7077 21.5585 29.5118

32.7105 33.7748 43.0483

50.5010 59.9157 65.2050

67.3650 92.0646 106.9222

122.5988 124.9217 151.1636

160.3178 169.7867 174.0642

179.6230 198.1834 201.2794

223.0499 226.9846 232.6412

242.2309 249.1015 266.1637

284.4971 284.6663 287.0227

297.2155 306.7225 325.1831

339.4569 341.2015 357.4692

364.1966 377.8596 405.7081

414.8586 416.5374 450.8849

482.9953 490.8575 509.3947

516.9005 527.2204 531.9010

549.3178 556.6701 567.0095

584.7603 586.2837 591.4107

601.3864 624.5924 628.3635

632.9509 643.8036 653.8128

670.5195 679.8107 698.7722

712.4941 737.6133 755.4198

773.6802 790.4518 818.7702

844.4306 848.8009 860.3050

867.1125 869.8724 883.1543

884.4215 905.6257 910.1213

914.1643 950.8326 961.6733

968.1292 982.6387 989.8268

1002.6143 1007.4221 1014.9148

1016.2809 1022.4702 1037.4347

1041.0389 1043.5587 1050.4491

1062.0897 1064.9282 1066.0317

1070.6741 1075.2086 1093.0967

1107.3967 1118.2776 1129.5383

1134.8035 1163.3711 1179.4593

1181.7095 1198.1642 1204.1801

1212.4072 1219.5052 1224.5219

1231.1711 1246.6488 1260.4983

1272.4103 1279.1384 1285.4948

1295.5994 1303.3592 1314.2447

1322.3852 1340.1236 1341.5455

1348.1401 1356.6283 1360.6843

1367.4593 1369.2120 1379.7737

1381.9093 1410.2581 1417.8321

1421.7368 1427.9893 1431.7113

1460.0510 1470.1664 1481.2451

1486.7335 1490.2970 1492.4652

1493.1240 1500.9911 1501.8475

1503.5427 1518.3818 1526.3321

1535.0862 1552.4597 1553.1623

1659.1075 1668.3945 1671.9817

1681.8891 1695.2200 1697.6979

1702.2796 1720.7372 1747.2147

3058.9248 3068.6187 3073.6079

3081.1113 3090.4399 3130.2158

3133.3187 3135.6926 3137.1664

3137.4168 3142.2576 3146.4963

3150.7175 3152.5978 3164.4801

3172.1978 3190.0586 3193.4276

3200.7312 3206.3029 3208.6033

3218.4254 3231.4209 3246.4767

3269.6959 3280.0936 3855.4692

**TS2**

Zero-point correction= 0.572630

Thermal correction to Energy= 0.610262

Thermal correction to Enthalpy= 0.611207

Thermal correction to Gibbs Free Energy= 0.499951

Sum of electronic and zero-point Energies= -6845.540912

Sum of electronic and thermal Energies= -6845.503279

Sum of electronic and thermal Enthalpies= -6845.502335

Sum of electronic and thermal Free Energies= -6845.613590

Cartesian coordinates

C 2.662757 2.967552 1.197116

C 1.825615 2.043888 0.580973

C 0.914334 2.409444 -0.406055

C 0.862190 3.753685 -0.748651

C 1.702987 4.700775 -0.161928

C 2.614330 4.306072 0.812975

C 3.504212 2.309736 2.262267

C 2.865212 0.914900 2.477685

C 1.956661 0.679136 1.221011

H 0.255624 1.689369 -0.890316

H 1.628905 5.740229 -0.460591

H 3.259472 5.043426 1.280561

H 3.524594 2.863691 3.205827

H 3.619866 0.131245 2.545062

H 4.541592 2.200520 1.929556

H 2.393738 -0.065604 0.558316

N 0.616997 0.232470 1.626348

O 2.154878 0.815407 3.701258

C -0.384234 -0.221280 0.824729

C 0.025207 0.837203 2.708735

N -1.510262 0.111826 1.474895

N -1.266168 0.770174 2.653192

C 0.915368 1.489004 3.718874

H 1.024756 2.558956 3.488887

H 0.492190 1.388892 4.718895

C -2.858801 -0.165299 1.065207

C -3.493969 0.728404 0.198390

C -3.478579 -1.298539 1.596240

C -4.792960 0.409063 -0.191179

C -4.779226 -1.567570 1.175551

C -5.438421 -0.739372 0.266621

H -5.313121 1.076153 -0.874470

H -5.287867 -2.445662 1.566508

C 1.493548 -2.612026 -0.286865

C 0.995085 -1.484063 -0.814529

H 0.772858 -3.139858 0.338809

C 2.795301 -3.287882 -0.398088

C 2.829638 -4.651161 -0.063512

C 4.000343 -2.666188 -0.759276

C 4.011777 -5.378098 -0.117407

H 1.907087 -5.142354 0.233822

C 5.185134 -3.393936 -0.803635

H 4.015887 -1.614582 -1.014083

C 5.198152 -4.750429 -0.489975

H 4.007728 -6.433356 0.136139

H 6.106388 -2.893267 -1.084594

H 6.125508 -5.312631 -0.530208

Br 1.942913 -0.446499 -2.122352

C -0.342781 -0.956648 -0.442164

O -0.998649 -0.243111 -1.455839

H -1.777406 -0.799384 -1.723745

Br -0.420591 4.318062 -2.020864

C -2.760087 -2.190068 2.572347

H -2.335213 -1.611176 3.397363

H -3.446979 -2.930373 2.987131

H -1.950544 -2.725422 2.066329

C -6.818565 -1.083599 -0.227768

H -7.453008 -0.195365 -0.284486

H -6.761443 -1.511605 -1.234038

H -7.303698 -1.815071 0.422147

C -2.816457 1.976685 -0.301456

H -2.160715 2.413867 0.458301

H -2.200516 1.746015 -1.174338

H -3.559000 2.725626 -0.584208

C -2.830121 -2.927694 -1.027563

O -3.006277 -1.941639 -1.755012

O -1.787730 -3.122129 -0.287522

H -1.084530 -2.104673 -0.273978

C -3.863126 -4.031722 -0.973257

H -3.504634 -4.883451 -1.558411

H -3.995173 -4.365989 0.057571

H -4.808500 -3.682242 -1.387820

Vibrational frequencies

-1134.5205 15.8665 19.6371

25.3472 29.9421 35.0257

44.0536 49.6325 53.4308

59.0483 61.8675 75.8203

84.6901 94.7649 100.0973

105.6604 114.3860 119.7713

123.7494 133.1079 147.0306

159.3181 163.9328 172.1723

181.7406 190.3148 192.2813

206.4024 211.7670 217.6320

229.3419 246.9129 253.2485

266.9505 279.1403 282.8501

285.7219 295.4824 298.3827

316.1420 333.9711 351.4201

358.5644 394.0670 415.3705

416.3898 442.4047 446.8839

464.0416 466.7727 494.6748

507.9370 511.6881 514.1825

523.1755 529.4292 548.2304

561.0552 572.1920 579.8552

590.3003 609.2370 614.3126

616.3772 624.3518 629.3483

641.3692 662.0899 667.7681

700.7686 713.3171 718.8632

730.1896 745.4337 760.6433

769.8078 783.3847 798.4178

821.0729 827.0762 841.7091

849.3249 872.0880 873.5007

876.5826 878.3426 895.6622

899.1643 909.9978 911.6095

949.9315 952.5570 957.2017

966.4880 970.0475 982.8760

1000.6327 1003.0591 1017.2719

1024.6929 1026.3420 1030.6899

1038.9879 1043.6635 1047.6328

1059.3902 1062.2763 1063.2269

1065.1427 1065.6172 1068.3596

1070.9070 1074.0792 1104.2081

1120.5496 1127.7339 1131.8041

1155.6830 1170.4017 1181.6914

1193.0577 1200.0506 1212.3353

1218.0575 1223.9326 1233.5323

1246.8012 1260.9744 1272.6680

1276.3680 1283.0904 1303.3690

1310.0830 1312.2657 1319.3218

1331.3969 1346.6123 1348.9682

1358.6064 1363.5940 1366.3855

1370.1618 1373.9831 1384.1593

1403.3594 1408.6530 1418.7419

1425.3392 1427.8031 1458.7119

1467.7757 1471.6189 1472.7854

1476.5704 1482.8004 1485.6299

1489.4063 1490.3319 1496.3471

1497.0953 1500.5272 1502.5755

1504.1304 1504.7647 1516.9558

1526.8860 1539.5050 1549.0269

1552.0981 1584.1889 1615.7704

1667.5622 1673.8954 1688.1464

1689.6100 1696.5558 1697.5156

1702.9656 1730.3918 1766.4817

3060.2136 3068.6569 3071.6051

3077.3721 3081.4296 3082.7051

3130.1984 3141.2959 3147.9515

3153.1072 3156.1991 3160.4349

3162.3615 3166.3494 3167.6781

3173.9256 3176.9589 3186.2074

3187.7965 3188.4995 3190.8522

3196.5215 3196.5753 3203.4057

3211.5332 3217.9810 3230.0666

3233.0206 3235.5561 3266.5945

**M3**

Zero-point correction= 0.512528

Thermal correction to Energy= 0.545727

Thermal correction to Enthalpy= 0.546672

Thermal correction to Gibbs Free Energy= 0.444282

Sum of electronic and zero-point Energies= -6616.602133

Sum of electronic and thermal Energies= -6616.568934

Sum of electronic and thermal Enthalpies= -6616.567989

Sum of electronic and thermal Free Energies= -6616.670379

Cartesian coordinates

C -3.761766 -0.963734 0.497113

C -2.651177 -0.167194 0.774577

C -2.515378 1.107904 0.242173

C -3.499475 1.551731 -0.628971

C -4.605539 0.765840 -0.944036

C -4.742662 -0.495590 -0.370280

C -3.704648 -2.274221 1.230623

C -2.608356 -2.051365 2.281847

C -1.746416 -0.834698 1.773138

H -1.659330 1.736423 0.463677

H -5.353700 1.145245 -1.630701

H -5.608636 -1.105154 -0.606768

H -3.378877 -3.066054 0.548879

H -3.046541 -1.725831 3.236328

H -4.656692 -2.550566 1.689638

H -1.504995 -0.164325 2.604758

N -0.431155 -1.319082 1.295161

O -1.891287 -3.249804 2.479832

C 0.437105 -0.907761 0.354019

C 0.187068 -2.325590 1.982580

N 1.544094 -1.629493 0.545676

N 1.397354 -2.530260 1.552375

C -0.602680 -3.060761 3.015054

H -0.156930 -4.039079 3.192724

H -0.630192 -2.499000 3.961040

C 2.831078 -1.457035 -0.084498

C 3.048232 -1.971882 -1.362538

C 3.805942 -0.774871 0.649771

C 4.319344 -1.785686 -1.907183

C 5.057984 -0.620515 0.059142

C 5.330547 -1.117865 -1.216752

H 4.520726 -2.181124 -2.899592

H 5.837135 -0.098446 0.608836

C 2.132720 2.018581 -0.465065

C 1.180808 1.125509 -0.592198

H 3.071916 1.831480 -0.991052

C 2.067662 3.258531 0.332034

C 3.097022 4.197234 0.217998

C 1.009055 3.514894 1.211042

C 3.062726 5.376592 0.956052

H 3.923404 4.001110 -0.459599

C 0.974834 4.690304 1.948665

H 0.218212 2.776796 1.314375

C 2.001983 5.626268 1.821871

H 3.864515 6.100579 0.854111

H 0.147988 4.879749 2.625510

H 1.974348 6.544856 2.398667

Br -1.415024 -2.919294 -1.299102

C 0.309840 0.142395 -0.690431

O -0.637915 0.026582 -1.631972

H -1.014814 -0.911146 -1.617529

C 3.505314 -0.216820 2.015092

H 3.291988 -1.015408 2.731479

H 4.352270 0.362871 2.385086

H 2.632463 0.445792 1.980401

C 6.682920 -0.914328 -1.849149

H 7.456319 -0.767389 -1.092324

H 6.961376 -1.771337 -2.466511

H 6.676568 -0.030853 -2.495617

C 1.961142 -2.672481 -2.125119

H 1.398758 -3.368375 -1.499118

H 1.222323 -1.959004 -2.506033

H 2.383280 -3.211167 -2.975277

Br -3.317910 3.267771 -1.405189

Vibrational frequencies

14.2951 16.8715 25.3821

30.7906 36.2316 44.2688

53.3028 55.2832 62.9348

74.2120 80.1233 89.5539

93.1405 114.1842 124.4359

138.3943 152.1130 154.7060

163.3788 168.1891 184.1318

187.6008 203.7058 214.6639

238.6408 259.8869 262.2090

271.7627 276.2392 284.0600

296.3049 306.5972 319.6691

321.5086 336.6778 357.1876

385.2360 391.4797 414.2716

429.8319 435.3761 462.3966

473.0382 495.7323 500.0743

513.4563 522.4623 528.9998

534.8009 562.5109 574.8694

589.7973 596.6598 603.8112

617.9572 627.8583 631.9758

658.1031 671.9762 681.5231

697.4018 710.4398 720.2638

736.0827 751.6622 763.1880

772.3989 790.1594 811.4585

832.0922 841.8731 844.3404

859.9104 867.6621 888.0605

901.1072 908.1885 920.3299

922.6458 939.8272 954.6244

967.4451 980.9417 989.4830

996.1124 1013.8417 1018.9357

1022.3685 1029.7402 1039.2178

1042.1778 1046.3164 1056.6302

1058.8623 1065.3941 1067.3989

1071.0178 1073.5388 1109.1406

1115.6178 1118.9132 1125.0984

1140.5451 1169.2819 1188.5648

1189.0825 1201.2843 1206.4339

1209.5880 1212.6623 1233.2119

1244.8632 1248.1186 1258.5422

1282.5204 1288.2794 1297.5090

1306.1037 1316.0643 1337.5577

1343.2196 1349.1058 1360.4609

1362.3529 1367.3751 1370.4028

1379.4880 1390.5600 1409.5891

1413.1968 1420.2006 1420.9839

1432.3080 1445.0721 1467.2921

1472.6616 1480.8144 1484.8300

1491.5823 1497.2826 1500.1896

1500.9475 1503.5332 1503.9514

1522.6309 1527.6936 1541.3446

1543.7318 1548.7656 1566.6834

1588.5108 1672.6428 1681.3541

1688.2467 1691.6506 1696.2630

1700.3377 1700.6014 2092.4718

2997.8666 3044.9372 3063.2305

3066.0191 3078.4918 3079.1179

3093.8264 3097.4948 3136.1351

3137.5266 3149.5135 3150.3315

3156.1983 3171.2540 3174.9541

3179.0613 3193.6743 3198.5239

3198.8909 3201.2291 3207.3249

3214.2011 3221.2891 3229.6605

3230.9428 3234.1490 3248.7903

**TS3**

Zero-point correction= 0.512240

Thermal correction to Energy= 0.544680

Thermal correction to Enthalpy= 0.545624

Thermal correction to Gibbs Free Energy= 0.445600

Sum of electronic and zero-point Energies= -6616.590373

Sum of electronic and thermal Energies= -6616.557932

Sum of electronic and thermal Enthalpies= -6616.556988

Sum of electronic and thermal Free Energies= -6616.657012

Cartesian coordinates

C 2.384335 2.899151 -0.701226

C 1.556643 1.843136 -0.333361

C 1.627872 0.602470 -0.956558

C 2.606408 0.432983 -1.928081

C 3.455028 1.470617 -2.311386

C 3.335432 2.716849 -1.702398

C 2.099116 4.134277 0.113166

C 0.868602 3.783300 0.987175

C 0.655502 2.230714 0.816405

H 0.981691 -0.228582 -0.684523

H 4.202440 1.298427 -3.077344

H 3.987934 3.530731 -2.003151

H 1.874580 5.010409 -0.504186

H 1.053577 4.007496 2.037921

H 2.953522 4.398743 0.743548

H 0.944314 1.712274 1.731224

N -0.742787 1.913777 0.557680

O -0.272331 4.558174 0.664201

C -1.468559 0.774560 0.779118

C -1.536923 2.775741 -0.154184

N -2.672002 1.037858 0.207367

N -2.712748 2.288063 -0.368375

C -0.966453 4.113437 -0.485350

H -0.302024 4.044884 -1.359149

H -1.764362 4.824123 -0.698772

C -3.778470 0.154030 -0.041013

C -3.679054 -0.751661 -1.096879

C -4.917923 0.281960 0.751878

C -4.786297 -1.561564 -1.347876

C -5.998892 -0.545823 0.460648

C -5.948103 -1.474317 -0.581166

H -4.737528 -2.276630 -2.165483

H -6.900545 -0.467644 1.063517

C 1.273895 -0.674923 2.389723

C 0.171737 -0.829677 1.666181

H 1.092635 -0.097771 3.299528

C 2.669417 -1.117020 2.235219

C 3.488832 -1.053281 3.372064

C 3.241457 -1.529549 1.024246

C 4.829241 -1.415123 3.310900

H 3.062750 -0.721611 4.315560

C 4.584483 -1.879445 0.961804

H 2.638730 -1.591156 0.130687

C 5.383909 -1.828273 2.101609

H 5.441401 -1.369986 4.205895

H 5.000641 -2.184264 0.006233

H 6.432343 -2.104077 2.048447

Br 0.273485 -2.696323 0.167071

C -1.120010 -0.403122 1.488930

O -2.146468 -1.311927 1.631849

H -1.826883 -2.136474 1.212489

Br 2.818304 -1.282957 -2.697800

C -4.948322 1.266927 1.888357

H -4.167818 1.029741 2.618892

H -4.768058 2.285536 1.532172

H -5.913543 1.241904 2.396646

C -7.119562 -2.382674 -0.852089

H -7.128732 -2.718125 -1.891488

H -7.072302 -3.272836 -0.216426

H -8.066650 -1.879241 -0.643514

C -2.417181 -0.867547 -1.908171

H -2.008664 0.117724 -2.155170

H -1.646717 -1.422365 -1.358423

H -2.609139 -1.401205 -2.840816

Vibrational frequencies

-151.5905 12.8179 18.9804

20.0265 34.8001 41.5366

46.6042 56.3519 66.3810

72.3293 77.4553 87.5330

103.9762 110.8200 131.7801

142.5250 145.3257 163.4750

167.5670 177.7077 183.3386

194.3084 197.2391 216.2942

220.6799 237.8191 256.0979

276.9155 280.8606 286.0937

294.6327 304.7314 315.9550

333.3155 337.5284 364.5470

394.0832 400.0662 419.7446

423.7525 451.2770 466.5511

470.2088 499.7778 509.3018

512.5230 519.3947 524.3660

525.7649 537.8413 553.0532

564.2726 585.0964 589.9173

605.4329 622.4817 626.3246

631.0147 649.4003 663.7777

693.1338 714.1719 715.6289

719.2136 742.6664 750.2183

770.6200 783.4204 809.7956

831.6535 844.0333 855.4428

870.2386 878.9209 880.3755

884.3687 894.0256 908.8541

910.2769 955.9565 965.5908

968.6405 980.7637 999.3560

1007.2605 1012.7881 1018.5336

1021.7820 1032.2930 1035.0190

1039.0773 1040.3650 1057.9496

1060.1350 1062.4671 1066.2912

1068.9540 1070.6981 1071.8577

1107.1685 1116.6019 1125.6975

1134.5625 1156.6725 1178.9708

1187.5233 1200.8429 1203.4089

1211.8884 1217.9816 1230.8325

1239.4199 1246.4478 1248.1159

1265.1994 1283.4468 1283.9465

1302.5948 1306.5274 1319.4940

1334.5147 1341.8200 1346.3615

1355.6528 1360.3517 1362.0510

1367.2733 1386.4194 1392.0378

1416.0633 1422.2892 1424.0482

1429.3904 1436.0420 1454.9662

1472.0368 1476.2173 1487.3151

1494.0587 1496.5726 1497.3091

1499.8003 1502.8386 1504.2904

1507.0398 1511.4161 1528.2975

1538.5019 1550.3850 1559.4506

1570.7732 1668.2388 1676.0449

1690.6547 1694.7874 1695.1724

1700.2206 1723.0900 1864.4610

3055.8285 3062.1365 3064.7748

3071.6014 3089.8412 3127.1419

3131.9791 3132.0352 3135.2291

3141.4956 3152.7633 3158.9568

3164.4177 3164.6333 3164.8782

3185.5048 3190.7820 3192.3658

3197.8760 3204.6318 3205.0069

3210.3741 3218.7953 3232.5999

3249.3746 3300.4961 3619.5323

**M4**

Zero-point correction= 0.522427

Thermal correction to Energy= 0.556233

Thermal correction to Enthalpy= 0.557177

Thermal correction to Gibbs Free Energy= 0.452726

Sum of electronic and zero-point Energies= -6617.034159

Sum of electronic and thermal Energies= -6617.000353

Sum of electronic and thermal Enthalpies= -6616.999409

Sum of electronic and thermal Free Energies= -6617.103861

Cartesian coordinates

C -3.300450 -0.243432 1.211241

C -2.101841 0.475119 1.196147

C -1.951751 1.599167 0.390626

C -3.003854 1.976379 -0.439870

C -4.186181 1.236292 -0.430724

C -4.357808 0.128798 0.394114

C -3.225720 -1.408750 2.158019

C -2.047315 -1.004170 3.066597

C -1.122612 -0.140618 2.163430

H -1.039507 2.188119 0.403036

H -2.918305 2.845547 -1.081314

H -5.291950 -0.421313 0.389262

H -3.005492 -2.318126 1.585819

H -2.416618 -0.342446 3.853068

H -4.141364 -1.580311 2.726160

H -0.528924 0.579713 2.731254

N -0.146871 -1.067671 1.511429

O -1.370566 -2.039358 3.739274

C 0.764556 -0.807230 0.564748

C 0.028878 -2.385843 1.854606

N 1.449782 -1.924647 0.372320

N 1.003640 -2.923405 1.176572

C -0.823544 -3.031204 2.902396

H -1.594939 -3.646707 2.423559

H -0.203952 -3.679154 3.523232

C 2.676773 -2.056209 -0.383905

C 2.625111 -2.366842 -1.741485

C 3.868913 -1.870395 0.327790

C 3.847873 -2.471171 -2.406929

C 5.055648 -1.986212 -0.385807

C 5.063547 -2.282349 -1.752844

H 3.842712 -2.714746 -3.465607

H 5.999101 -1.848167 0.135915

C 1.965274 2.662018 -0.307867

C 1.750671 1.509687 0.372459

H 1.494647 2.737315 -1.288602

C 2.750440 3.806140 0.114016

C 2.787806 4.928084 -0.729008

C 3.472092 3.830932 1.320422

C 3.521616 6.052932 -0.375115

H 2.234759 4.909181 -1.663629

C 4.205070 4.953206 1.670062

H 3.467547 2.968365 1.979235

C 4.229410 6.065326 0.824080

H 3.543525 6.915869 -1.031142

H 4.763456 4.967515 2.599536

H 4.806279 6.940985 1.102589

C 0.949793 0.469735 -0.240303

O 0.401488 0.503539 -1.328385

H 2.160970 1.319128 1.359524

Br -5.607771 1.763218 -1.553358

C 3.863558 -1.590897 1.807345

H 3.203796 -0.754008 2.061689

H 3.515083 -2.463951 2.367243

H 4.867347 -1.341608 2.152312

C 6.370780 -2.401967 -2.489954

H 6.940771 -1.471482 -2.418291

H 6.985594 -3.196454 -2.057763

H 6.213285 -2.626666 -3.545931

C 1.322577 -2.578068 -2.459517

H 0.662006 -3.244001 -1.900035

H 0.794703 -1.628445 -2.592375

H 1.500888 -3.010916 -3.444480

Br -1.902061 -2.105659 -1.169381

H -1.400391 -0.811939 -1.486358

Vibrational frequencies

9.7338 19.2232 24.6239

29.1009 36.1688 39.5769

42.9793 53.0262 64.9722

71.2755 75.4241 85.9377

89.3605 109.1779 117.4329

125.4761 139.0312 145.7869

155.6696 176.6268 188.0567

200.7605 208.6769 219.5502

233.6807 239.7493 244.3035

250.6466 267.5393 281.4325

289.2418 291.6649 304.4667

321.2484 330.6675 362.6586

364.4299 395.4654 408.6509

409.8508 437.3625 449.3577

466.3542 478.0301 484.6366

493.9771 509.6208 520.1732

525.1619 542.4403 565.6134

580.6285 583.3038 590.9087

592.1703 614.0393 626.5948

628.4807 633.7896 641.0244

682.1707 695.5527 707.7542

720.9680 737.7065 746.9700

773.9066 784.1994 807.0146

817.6321 841.7465 858.3180

864.7927 881.5978 888.9811

891.3314 899.1806 908.5507

912.5970 922.9300 926.7230

962.0352 965.0443 981.7381

998.4118 1005.7624 1017.6151

1026.5452 1030.8751 1037.1044

1038.7845 1040.8833 1047.2096

1048.4834 1059.3706 1063.1513

1064.8485 1069.3744 1070.5517

1078.5916 1100.1178 1111.7519

1120.8069 1129.6529 1147.8351

1166.5798 1176.7864 1196.7418

1204.6750 1210.2199 1212.5336

1214.1718 1240.3810 1250.1661

1252.6527 1267.6929 1280.7429

1286.2665 1302.2442 1317.0290

1324.5933 1335.5463 1340.6915

1341.8989 1357.7603 1360.4810

1367.3747 1370.6308 1374.7673

1380.3043 1390.5837 1420.2757

1420.9571 1422.2800 1432.4360

1442.3579 1469.7130 1481.0053

1482.9196 1488.9511 1495.1152

1495.7038 1501.4737 1502.9901

1508.8533 1511.1928 1526.2127

1528.7136 1535.8932 1542.8047

1553.6187 1612.2047 1648.3295

1666.2971 1668.4535 1672.7075

1690.9879 1693.5273 1695.8978

1697.5266 1794.3490 2637.0430

3064.5011 3073.9137 3083.2478

3083.7895 3096.5018 3131.8449

3135.1268 3144.7518 3145.1938

3158.8191 3159.0699 3172.8726

3176.8243 3183.9219 3186.4075

3197.2166 3199.5590 3209.0297

3215.1988 3217.1521 3222.3290

3224.5873 3231.0225 3233.3052

3238.8447 3243.8617 3251.6343

**TS4**

Zero-point correction= 0.570141

Thermal correction to Energy= 0.607813

Thermal correction to Enthalpy= 0.608758

Thermal correction to Gibbs Free Energy= 0.496616

Sum of electronic and zero-point Energies= -6845.526201

Sum of electronic and thermal Energies= -6845.488529

Sum of electronic and thermal Enthalpies= -6845.487584

Sum of electronic and thermal Free Energies= -6845.599726

Cartesian coordinates

C -3.299892 -0.429032 0.929078

C -2.173226 0.397206 0.936825

C -2.063561 1.488822 0.085547

C -3.076207 1.705490 -0.846394

C -4.172877 0.846970 -0.875738

C -4.318050 -0.214270 0.013058

C -3.193854 -1.481974 2.001303

C -2.173711 -0.828334 2.958105

C -1.227934 -0.042037 2.020397

H -1.219115 2.167242 0.166772

H -3.026009 2.539811 -1.535629

H -5.196203 -0.848469 -0.023208

H -2.793838 -2.395998 1.543772

H -2.685941 -0.086814 3.575459

H -4.138714 -1.715427 2.495357

H -0.694710 0.778183 2.500621

N -0.197477 -1.015527 1.547010

O -1.501315 -1.667322 3.872162

C 0.652846 -0.929758 0.518898

C 0.083756 -2.202421 2.166937

N 1.445075 -1.992023 0.580169

N 1.098757 -2.803395 1.610092

C -0.767659 -2.715139 3.285804

H -1.422384 -3.500086 2.884184

H -0.137180 -3.151839 4.061753

C 2.627630 -2.238029 -0.213004

C 2.611434 -3.266673 -1.164943

C 3.760736 -1.462470 0.059927

C 3.775129 -3.454741 -1.905701

C 4.893174 -1.695214 -0.721969

C 4.914740 -2.671290 -1.714243

H 3.791418 -4.245209 -2.652101

H 5.786331 -1.106398 -0.528322

C 1.885186 2.061903 -1.288553

C 1.319412 1.308434 -0.302228

H 2.112821 1.591032 -2.249334

C 2.314625 3.439046 -1.144508

C 3.167298 3.996907 -2.110668

C 1.877634 4.228018 -0.065205

C 3.599752 5.310363 -1.989895

H 3.491961 3.387668 -2.949750

C 2.305914 5.542723 0.044217

H 1.180438 3.813221 0.658919

C 3.170986 6.081873 -0.910494

H 4.265711 5.734968 -2.733338

H 1.963219 6.153553 0.872659

H 3.505822 7.110083 -0.816339

Br -1.614802 -2.554667 -1.038351

C 0.691466 0.103709 -0.570353

O 0.052967 -0.182164 -1.647119

H -0.567175 -1.044058 -1.543319

H 1.481077 1.553123 0.995396

C 1.022627 2.484850 2.852115

O -0.015281 2.962094 2.384807

O 1.782186 1.637025 2.233234

C 1.508621 2.865367 4.238583

H 1.678772 1.962962 4.830091

H 2.468086 3.381451 4.150445

H 0.783923 3.511141 4.732293

Br -5.536039 1.151715 -2.155382

C 3.815893 -0.435457 1.160172

H 3.056227 -0.585374 1.929035

H 4.795486 -0.471754 1.642067

H 3.674245 0.576865 0.771831

C 6.138779 -2.887819 -2.564068

H 6.008748 -2.423884 -3.546985

H 7.026072 -2.450577 -2.102026

H 6.320998 -3.952873 -2.726567

C 1.412705 -4.141329 -1.403290

H 0.865834 -4.349026 -0.482499

H 0.697643 -3.659938 -2.074812

H 1.732682 -5.084867 -1.849594

Vibrational frequencies

-1236.6861 -13.3043 12.6358

19.3122 26.1617 31.2433

42.7164 45.4684 47.7249

58.5715 60.3099 67.1056

70.2354 75.6655 78.4085

86.9930 103.7166 108.1210

112.4778 118.6519 126.4144

127.4958 132.2283 142.6745

155.7016 178.0772 182.4957

187.4370 210.3949 211.6111

217.9479 225.7611 230.8979

244.6680 253.8294 275.5459

286.2489 292.9854 303.4636

332.4370 341.2365 343.0238

357.5935 366.9837 377.3950

409.5351 419.1477 428.7847

451.2636 461.8764 473.6100

496.4738 504.1577 513.6731

517.5044 526.8485 533.6880

545.5315 569.3519 579.2743

585.7806 591.4110 601.2196

612.6539 613.6648 627.4778

632.4565 639.3767 676.8979

684.5697 699.9989 708.7278

719.3651 735.9423 748.1264

764.1834 773.7895 803.2586

819.3167 833.4014 849.6747

871.2415 881.1250 886.9597

888.1434 899.7980 911.8448

918.3807 919.1119 943.1534

966.5485 968.8609 977.9111

979.6823 990.9351 997.7071

1009.4524 1016.3317 1023.4113

1027.1769 1031.6735 1041.2651

1048.2549 1050.2558 1052.2060

1053.7948 1055.3454 1061.7503

1066.5902 1068.8975 1069.2940

1071.5765 1099.6652 1113.7462

1130.5998 1135.5354 1151.2681

1167.7001 1186.3408 1190.1340

1201.6592 1205.9541 1215.9081

1222.3616 1242.5480 1252.4643

1266.5008 1269.4776 1277.6735

1280.0515 1306.9242 1313.7934

1322.1899 1325.9231 1335.0046

1337.9897 1346.9023 1354.6643

1366.2349 1368.6903 1370.7754

1374.1105 1377.0239 1379.3035

1418.4581 1420.6681 1421.7908

1430.4301 1434.9046 1444.6479

1452.9736 1465.1577 1474.8659

1477.4808 1482.5712 1486.4453

1490.5217 1492.9379 1497.1558

1500.0985 1503.4373 1504.9907

1512.4126 1515.4258 1521.0895

1532.4701 1537.3782 1544.4495

1545.5463 1583.9779 1614.0130

1661.2036 1667.1585 1673.3965

1681.4785 1688.8441 1694.1529

1702.4299 1703.6229 1770.2645

2209.4020 3072.7521 3074.6170

3081.8853 3082.6695 3086.8439

3088.3275 3139.3326 3141.9072

3146.9160 3155.4703 3157.2740

3160.4148 3164.9422 3165.3794

3166.9814 3180.4087 3184.9782

3190.8389 3195.4598 3196.6524

3200.3824 3206.7327 3211.7281

3216.3980 3222.3847 3230.8156

3235.8189 3239.3911 3247.2859

**M5SS**

Zero-point correction= 0.812606

Thermal correction to Energy= 0.864598

Thermal correction to Enthalpy= 0.865542

Thermal correction to Gibbs Free Energy= 0.725625

Sum of electronic and zero-point Energies= -5393.910533

Sum of electronic and thermal Energies= -5393.858541

Sum of electronic and thermal Enthalpies= -5393.857597

Sum of electronic and thermal Free Energies= -5393.997514

Cartesian coordinates

O 0.427683 -2.470220 -0.552704

O 3.151169 0.632059 1.849961

O -0.748723 -0.557471 -1.586328

O 4.803462 -1.780925 1.217779

N 4.372529 2.152404 0.635562

C 5.019490 2.812291 1.744439

H 4.757414 2.261834 2.648251

H 4.672658 3.847813 1.828398

C 2.938071 0.748626 -0.605878

C -0.487820 -0.592581 -0.390876

C -0.176580 4.411286 -1.091948

H -0.651789 4.967680 -1.894230

C 0.656546 -0.011136 0.243291

H 0.642199 0.180798 1.306764

C 4.472462 2.552780 -0.702701

C 3.646000 1.747605 -1.492341

C 1.402914 0.968400 -0.627388

H 1.110262 0.750742 -1.660791

C 0.948175 2.392956 -0.335223

C 3.578038 1.958363 -2.856687

H 2.938588 1.340271 -3.482451

C -0.134983 4.935534 0.195926

C 0.460009 4.196441 1.217541

H 0.502502 4.599433 2.225771

C 0.360181 3.151129 -1.351194

H 0.300936 2.736368 -2.355088

C 3.488195 1.114146 0.780199

C 3.273782 -0.683563 -1.073338

H 2.832899 -0.797580 -2.070925

H 4.360410 -0.769159 -1.183317

C 4.343134 2.986736 -3.420925

H 4.299203 3.166252 -4.490074

C 0.996015 2.938583 0.956042

H 1.443069 2.367558 1.766940

C 5.158067 3.781112 -2.618921

H 5.744009 4.575870 -3.070241

C 1.322326 -2.009111 0.201432

C 5.237571 3.576568 -1.237487

H 5.871573 4.196919 -0.612101

C 2.825039 -1.876245 -0.212942

C 1.353501 -2.290085 1.686075

C 3.607520 -1.931113 1.119761

C 2.924733 -2.459601 3.543366

H 3.947093 -2.399629 3.903981

C 0.300587 -2.654417 2.525096

H -0.703381 -2.795233 2.137238

C 2.645911 -2.228739 2.200154

C 1.865921 -2.775813 4.386096

H 2.043805 -2.963074 5.440328

C 0.565976 -2.881477 3.873132

H -0.247555 -3.159141 4.537249

C 3.174685 -3.150066 -0.981332

F 2.564932 -3.214342 -2.167169

F 2.832818 -4.245698 -0.281904

F 4.495195 -3.237238 -1.220583

H -0.563011 5.911728 0.403518

H 6.105351 2.804052 1.611994

C -3.345691 2.644594 2.297961

C -2.566523 1.869605 1.445910

C -2.530163 2.083704 0.073265

C -3.308411 3.115723 -0.430322

C -4.085247 3.923966 0.399958

C -4.104183 3.688773 1.772767

C -3.221666 2.181878 3.728178

C -2.367627 0.886707 3.676660

C -1.824524 0.797764 2.203626

H -1.920777 1.482080 -0.594858

H -4.677529 4.723227 -0.031944

H -4.717865 4.310374 2.417942

H -4.190094 1.973796 4.196227

H -1.524214 0.940085 4.364638

H -2.726167 2.935996 4.348080

H -0.750864 0.964262 2.184312

N -2.108255 -0.520145 1.607776

O -3.073745 -0.268000 4.093876

C -1.625494 -1.101352 0.486159

C -3.243649 -1.201625 1.951111

N -2.479727 -2.081598 0.198643

N -3.484804 -2.169889 1.118624

C -4.007707 -0.747244 3.152144

H -4.733505 0.027923 2.864181

H -4.544544 -1.583262 3.601038

C -2.581510 -2.875396 -1.006044

C -3.248297 -2.288167 -2.085932

C -2.127091 -4.193092 -1.001808

C -3.410599 -3.068832 -3.227364

C -2.317282 -4.926885 -2.171597

C -2.940538 -4.380907 -3.292795

H -3.921898 -2.639666 -4.085986

H -1.967817 -5.956140 -2.202752

Br -3.311754 3.403567 -2.297228

C -1.452207 -4.801275 0.195398

H -0.436073 -4.401975 0.272710

H -1.990052 -4.568953 1.120848

H -1.404335 -5.887906 0.094620

C -3.093455 -5.187632 -4.556209

H -2.242365 -5.016650 -5.224556

H -3.134723 -6.258522 -4.340819

H -4.000551 -4.908306 -5.099005

C -3.777626 -0.878306 -2.032170

H -4.224717 -0.648362 -1.058317

H -2.970368 -0.160909 -2.211177

H -4.545726 -0.732550 -2.794992

Vibrational frequencies

-518.4503 13.2494 18.5008

23.4508 25.6743 31.7282

34.0402 39.6380 45.7170

46.6771 51.1551 54.4916

56.5279 63.8554 71.1568

74.5830 78.9932 86.5101

94.6429 106.8177 116.6475

123.6366 125.1214 132.2152

138.9344 143.2235 152.3144

156.4937 165.1532 170.3959

175.9857 178.9441 188.5786

189.2497 194.4895 199.6016

206.6578 207.5519 221.8595

226.7320 233.5812 237.9903

243.5922 246.4859 254.2002

263.4500 280.3184 282.7179

284.4817 290.8991 301.4540

309.2394 312.9459 317.1317

325.0258 331.2695 334.5669

347.5862 350.9195 360.8155

375.6492 396.0679 405.1496

414.8931 425.5070 435.6614

440.2271 454.8349 463.8429

470.4891 480.4051 491.6198

499.9416 504.4209 510.5491

512.5057 515.9391 524.5472

527.0653 534.2821 541.8584

549.7942 552.9153 554.3899

559.2334 565.9060 573.0390

586.3010 589.5843 599.5634

614.6964 623.4478 628.6043

632.3624 637.4905 648.4514

661.0083 663.3695 677.7473

688.4810 710.3790 714.7457

718.2139 721.6319 723.5917

727.3667 748.5487 753.5175

755.2644 772.2518 776.5198

779.6038 781.2522 786.1199

789.3893 796.6129 806.0930

812.5962 838.0518 841.5953

855.8173 858.9205 879.6092

880.4049 883.2775 888.8060

894.3202 894.8190 908.4638

910.7452 912.8459 914.9062

918.9751 941.3368 948.3828

962.1895 966.4027 971.1888

983.3303 983.6648 986.2513

987.3273 993.3598 1002.8744

1007.6881 1015.6980 1018.1300

1020.4269 1028.0560 1029.8312

1039.8303 1048.0502 1049.6044

1051.5556 1060.4613 1063.6771

1067.5670 1067.8619 1070.9299

1071.7124 1075.2745 1077.4933

1078.4261 1085.7798 1091.7452

1114.1021 1120.0031 1123.2995

1127.3705 1138.3761 1145.1337

1158.0492 1163.1670 1164.0438

1165.2940 1169.4299 1176.0350

1184.6578 1186.0315 1186.6211

1200.9777 1202.7047 1210.7524

1212.9885 1221.6839 1222.0754

1232.6648 1243.3714 1253.3495

1257.2182 1262.3479 1265.3227

1275.2195 1279.6108 1288.6076

1290.6608 1299.8769 1302.2532

1308.8362 1315.3637 1317.9003

1319.9002 1324.0788 1325.0528

1336.4428 1346.3622 1346.8219

1349.3099 1360.3487 1368.8319

1370.4695 1371.2924 1378.2643

1390.5922 1393.5993 1398.8513

1412.2106 1418.1828 1427.8679

1433.1337 1438.9517 1440.5685

1448.5824 1462.9317 1467.6981

1480.5116 1481.6981 1500.0399

1501.7500 1505.0165 1505.8422

1508.2239 1513.5238 1515.0021

1517.9130 1518.0684 1518.5772

1520.5624 1521.1241 1522.0751

1523.0358 1536.8671 1539.5018

1547.2392 1552.3480 1558.0101

1566.1917 1574.9720 1583.7637

1676.0687 1676.7991 1683.2889

1685.0738 1695.2427 1696.3861

1697.4827 1697.7517 1698.5275

1702.4843 1712.9006 1748.0464

1815.7054 1872.2938 3076.1535

3077.6991 3077.7791 3078.3506

3080.4618 3095.1321 3098.1941

3110.5074 3136.2382 3145.2271

3146.3133 3148.8656 3150.3791

3150.7439 3168.8275 3174.8222

3176.0539 3179.0172 3196.5051

3200.2971 3200.4079 3202.9356

3207.0010 3208.2326 3209.6738

3210.6108 3215.2226 3217.7685

3223.9250 3225.1465 3225.6288

3235.7448 3235.8714 3237.6623

3239.6911 3244.2341 3248.6037

3252.1959 3254.8320 3271.0417

**TS5SS**

Zero-point correction= 0.808125

Thermal correction to Energy= 0.860885

Thermal correction to Enthalpy= 0.861829

Thermal correction to Gibbs Free Energy= 0.721938

Sum of electronic and zero-point Energies= -5393.986620

Sum of electronic and thermal Energies= -5393.933860

Sum of electronic and thermal Enthalpies= -5393.932915

Sum of electronic and thermal Free Energies= -5394.072807

Cartesian coordinates

O 5.623315 0.334609 -0.714618

O 0.026314 -0.210503 0.653275

O -0.267222 2.004060 -2.301483

O 2.105680 1.729518 2.042389

N 0.991525 -2.315958 0.399180

C 0.127278 -3.012718 1.308207

H -0.520492 -2.266366 1.766096

H 0.708752 -3.511710 2.092753

C 1.949197 -0.619830 -0.785718

C -1.035845 1.181728 -1.800424

C 1.431212 -4.155087 -3.662635

H 2.259840 -4.516530 -4.262743

C -0.997915 -0.233611 -1.906726

H -1.682535 -0.812972 -1.301458

C 2.073821 -2.854601 -0.261589

C 2.709465 -1.815788 -0.994869

C 0.070717 -0.847865 -2.522423

H 0.742259 -0.215047 -3.097665

C 0.197969 -2.289531 -2.710185

C 3.851920 -2.125287 -1.742126

H 4.365184 -1.350690 -2.305397

C 0.573164 -5.054027 -3.036770

C -0.477056 -4.575936 -2.251543

H -1.149496 -5.272683 -1.759983

C 1.243174 -2.787778 -3.500149

H 1.924597 -2.086148 -3.972671

C 0.907712 -0.937921 0.152256

C 2.423199 0.784440 -0.999498

H 2.880313 0.921611 -1.986476

H 1.581834 1.477496 -0.914225

C 4.341958 -3.429616 -1.729047

H 5.234404 -3.671270 -2.298152

C -0.661088 -3.210152 -2.086808

H -1.472441 -2.858945 -1.453841

C 3.698353 -4.433978 -0.997004

H 4.094974 -5.444613 -1.008632

C 4.702806 0.296401 0.067524

C 2.547534 -4.158256 -0.256421

H 2.037110 -4.937174 0.302315

C 3.480534 1.231744 0.068835

C 4.514160 -0.671343 1.179586

C 2.876203 1.000445 1.467873

C 2.977636 -1.085487 3.010576

H 2.117401 -0.784660 3.599785

C 5.204540 -1.855197 1.420816

H 6.033921 -2.148062 0.786061

C 3.429119 -0.288402 1.963189

C 3.650174 -2.280502 3.243470

H 3.318085 -2.937532 4.041218

C 4.754319 -2.658415 2.461509

H 5.250491 -3.601366 2.666889

C 3.881046 2.669323 -0.165624

F 4.501299 2.826371 -1.342177

F 4.720199 3.104209 0.792880

F 2.821726 3.487960 -0.165706

H 0.722246 -6.122787 -3.154358

H -0.486280 -3.761137 0.790627

C -5.606558 -1.407588 -0.268611

C -4.311905 -0.893198 -0.213872

C -3.424922 -1.220432 0.810800

C -3.897348 -2.096693 1.787203

C -5.178553 -2.644382 1.738430

C -6.041249 -2.301343 0.702206

C -6.369008 -0.834740 -1.435324

C -5.555767 0.408609 -1.846214

C -4.104052 0.088772 -1.353068

H -2.415872 -0.805390 0.868701

H -5.499946 -3.318327 2.524138

H -7.046969 -2.708517 0.674193

H -7.396174 -0.551648 -1.192375

H -5.565265 0.569505 -2.925143

H -6.407206 -1.548892 -2.265492

H -3.512592 -0.327800 -2.171446

N -3.443640 1.304101 -0.888304

O -6.088757 1.606443 -1.298841

C -2.178555 1.758405 -0.989848

C -4.148147 2.217233 -0.154422

N -2.169653 2.913621 -0.323215

N -3.393133 3.213189 0.201125

C -5.618122 1.990544 -0.017998

H -5.827876 1.231864 0.746951

H -6.117258 2.920987 0.251143

C -1.054260 3.779529 -0.012292

C -0.424494 3.587067 1.219212

C -0.730985 4.801628 -0.903627

C 0.608652 4.464543 1.535859

C 0.309370 5.650905 -0.533987

C 0.991211 5.490883 0.673014

H 1.142526 4.319606 2.470798

H 0.596483 6.452723 -1.209418

Br -2.809933 -2.532833 3.273326

C -1.472272 4.964214 -2.201525

H -1.271994 4.104829 -2.847957

H -2.552696 5.026215 -2.035137

H -1.153362 5.872748 -2.714638

C 2.160774 6.371394 1.023939

H 3.092980 5.815831 0.879840

H 2.194645 7.263216 0.394412

H 2.121145 6.685524 2.070152

C -0.841266 2.484088 2.152164

H -1.922195 2.505949 2.327197

H -0.564256 1.509247 1.736572

H -0.333065 2.594423 3.110767

Vibrational frequencies

-39.6033 8.7799 23.3073

35.2426 36.8444 39.3706

46.6870 47.9801 52.7178

54.9326 58.6380 61.2467

66.9953 72.5141 78.9318

81.9856 83.1397 91.3712

94.8852 104.4285 108.6910

117.5026 119.9887 123.5924

130.0206 139.6257 140.7130

146.3368 152.1695 154.7399

158.3347 161.9760 165.3229

181.5923 190.6047 196.2496

206.5329 207.2022 211.0443

217.5359 223.0690 231.4578

232.8028 247.8751 254.9829

265.2157 269.2845 276.2780

283.8492 290.0983 294.5055

298.9271 299.8382 310.4651

319.2010 328.3905 330.6730

332.0326 346.1935 348.6041

357.4450 361.2182 384.7393

394.2496 404.8811 417.3868

419.5266 421.3098 430.3232

451.5505 460.5320 469.3100

478.0387 502.1355 504.5314

512.9591 518.0734 524.1413

525.1406 529.7274 538.5384

541.5743 544.2144 547.6683

559.0656 561.5514 576.5903

579.0329 582.1534 590.4609

595.7677 607.9886 613.7283

619.3724 627.9271 638.3260

649.5867 663.9249 671.5068

681.0544 684.3304 702.4834

719.5979 724.3800 727.6441

728.8261 739.1250 744.5467

749.9024 751.6961 755.9712

758.2537 761.2282 776.8575

786.5410 800.5623 801.7003

803.3557 806.7252 815.1887

836.0635 849.4217 851.4600

858.4094 860.0586 868.3224

869.5812 876.6947 887.9931

902.0863 903.5319 906.9087

910.3598 915.3869 918.8368

924.3485 937.0612 960.7130

969.4401 971.9267 977.5475

983.3373 985.9444 986.2322

991.2331 993.2930 1006.1438

1006.7484 1015.9766 1020.0819

1020.7596 1035.8934 1039.9621

1043.9753 1049.0900 1054.0743

1056.8054 1057.5081 1058.8598

1059.8911 1063.2260 1066.9031

1069.4752 1071.6202 1077.2695

1088.5072 1115.6533 1120.4569

1120.7840 1121.8865 1128.5319

1133.6628 1144.7390 1152.1026

1153.7989 1164.4240 1177.6906

1178.6178 1188.1014 1188.4845

1194.9148 1205.9845 1209.9843

1210.7232 1224.6491 1238.8982

1242.5871 1246.0229 1250.7523

1266.1455 1271.3801 1274.6977

1277.3387 1279.9722 1284.3258

1284.7201 1296.4432 1303.2592

1313.5439 1315.2173 1317.3603

1324.1030 1331.4741 1333.5789

1339.8253 1349.7354 1353.8322

1362.6526 1367.4585 1368.2063

1372.5614 1378.3210 1389.1406

1398.0546 1399.2544 1410.0779

1410.9347 1420.4572 1420.9626

1429.4026 1434.0551 1445.4968

1467.1484 1468.2364 1470.9331

1474.3851 1480.5824 1488.2293

1489.9765 1492.2901 1496.5000

1496.7266 1497.3814 1500.8473

1502.3041 1509.3209 1520.2148

1524.7630 1525.4426 1530.0843

1531.2031 1534.5657 1537.7626

1542.8854 1544.1203 1549.9086

1563.6889 1596.4675 1658.1317

1668.8255 1671.2027 1688.0772

1689.0418 1689.4234 1690.6555

1694.1162 1695.5596 1696.0551

1696.8432 1702.1567 1740.8999

1861.5939 1900.7669 3042.4714

3062.0537 3069.1371 3069.5020

3083.7445 3084.3174 3087.5895

3100.5547 3141.2113 3141.4880

3144.7520 3147.3183 3149.2635

3151.4374 3154.6180 3164.4858

3168.0682 3173.5105 3174.7488

3184.0686 3187.6996 3190.0641

3194.6532 3201.5871 3204.4563

3204.6163 3204.9198 3206.2965

3206.8970 3211.0550 3212.9706

3215.7505 3217.2477 3221.1394

3225.2771 3226.8252 3233.5521

3239.4339 3244.6107 3257.0537

**TS5RR**

Zero-point correction= 0.806574

Thermal correction to Energy= 0.860454

Thermal correction to Enthalpy= 0.861398

Thermal correction to Gibbs Free Energy= 0.715869

Sum of electronic and zero-point Energies= -5393.987038

Sum of electronic and thermal Energies= -5393.933158

Sum of electronic and thermal Enthalpies= -5393.932213

Sum of electronic and thermal Free Energies= -5394.077743

Cartesian coordinates

O -0.918525 -2.058633 1.476449

O 1.485488 -0.403241 0.911694

O -2.304021 -0.849178 -1.650254

O 2.716550 -4.564473 -0.262882

N 3.342384 -0.022554 -0.431822

C 3.937847 1.000054 0.386756

H 3.223376 1.236693 1.178606

H 4.133459 1.899761 -0.206895

C 1.820512 -1.528335 -1.214648

C -1.760061 0.017804 -0.969666

C 3.057533 1.617719 -4.451961

H 3.511294 1.344397 -5.398676

C -0.511973 0.677142 -1.180937

H -0.230655 1.467910 -0.501620

C 3.851878 -0.527710 -1.613199

C 2.941839 -1.506573 -2.104714

C 0.318563 0.353685 -2.228589

H -0.009194 -0.444625 -2.892173

C 1.420018 1.175371 -2.712182

C 3.255647 -2.163850 -3.299057

H 2.587996 -2.921413 -3.701191

C 3.530568 2.714218 -3.736877

C 2.953451 3.044446 -2.508645

H 3.302017 3.905705 -1.946082

C 2.011854 0.856303 -3.943564

H 1.647687 -0.004113 -4.496457

C 2.128977 -0.642269 -0.137922

C 0.679534 -2.494704 -1.187378

H -0.294511 -2.004029 -1.260067

H 0.768193 -3.174189 -2.041591

C 4.437473 -1.842981 -3.965833

H 4.685006 -2.357491 -4.889277

C 1.913075 2.280605 -1.998825

H 1.495460 2.539946 -1.029580

C 5.312649 -0.874594 -3.462492

H 6.226135 -0.643095 -4.001105

C 0.138167 -2.641700 1.356097

C 5.028265 -0.204251 -2.270979

H 5.700274 0.551582 -1.875397

C 0.611442 -3.371884 0.081621

C 1.156719 -2.886362 2.415304

C 1.991619 -3.935153 0.468098

C 3.340526 -3.924272 2.671962

H 4.173067 -4.468455 2.238726

C 1.124118 -2.536404 3.761828

H 0.263698 -2.031391 4.187242

C 2.239689 -3.588282 1.890250

C 3.322677 -3.549739 4.010558

H 4.161951 -3.795331 4.652700

C 2.220178 -2.871218 4.550632

H 2.222677 -2.610719 5.604170

C -0.353306 -4.522483 -0.156700

F -1.576028 -4.082652 -0.477926

F -0.476059 -5.284464 0.947057

F 0.057338 -5.322690 -1.146115

H 4.346985 3.311018 -4.131141

H 4.876442 0.657451 0.835827

C 0.775448 2.288152 3.217602

C 0.015092 2.310920 2.046003

C 0.150449 3.327413 1.112091

C 1.126989 4.292047 1.348381

C 1.904979 4.286365 2.501517

C 1.716490 3.282245 3.452501

C 0.450025 1.055602 4.031099

C -0.887884 0.582926 3.442644

C -0.769957 1.023120 1.972240

H -0.453364 3.376753 0.211239

H 2.651429 5.057907 2.650421

H 2.315820 3.277535 4.357515

H 0.370331 1.234838 5.105453

H -1.033827 -0.498619 3.485144

H 1.212696 0.287173 3.851656

H -0.167258 0.282956 1.427729

N -2.087961 1.073665 1.362347

O -1.977020 1.184581 4.152170

C -2.550233 0.555384 0.211195

C -3.165638 1.466215 2.098869

N -3.876858 0.662374 0.289940

N -4.279448 1.228684 1.465057

C -2.884640 2.019708 3.457329

H -2.478710 3.034556 3.343984

H -3.805072 2.068426 4.039591

C -4.863686 0.249648 -0.676651

C -5.409477 1.223772 -1.509429

C -5.241484 -1.094317 -0.694437

C -6.389194 0.808805 -2.410382

C -6.224611 -1.454483 -1.611472

C -6.803905 -0.521360 -2.475393

H -6.835783 1.542979 -3.075693

H -6.544517 -2.492389 -1.654943

Br 1.435835 5.603496 0.018136

C -4.597950 -2.094615 0.225525

H -3.549655 -2.244123 -0.051568

H -4.627919 -1.753588 1.265254

H -5.112841 -3.054517 0.165963

C -7.842635 -0.956856 -3.475834

H -7.367676 -1.443600 -4.333393

H -8.537458 -1.675196 -3.033981

H -8.415846 -0.105871 -3.848999

C -4.949941 2.655676 -1.435469

H -5.133214 3.077217 -0.442627

H -3.876354 2.735116 -1.637359

H -5.476524 3.265425 -2.170581

Vibrational frequencies

-33.6267 9.6721 16.2250

23.1314 25.2771 32.4205

33.8460 36.9188 41.2520

41.6267 46.0767 51.8017

56.3946 61.8725 66.4701

67.3293 70.5087 71.7792

79.5145 99.3985 105.5821

111.1942 117.8409 122.3650

125.9253 130.5090 133.1793

140.6361 146.1523 153.0344

156.2298 160.1047 169.6235

172.3514 173.6942 184.8017

189.5999 193.0105 195.9142

198.5361 202.1009 219.3533

228.5742 237.1793 241.2809

242.8841 250.0932 262.9199

271.5243 274.6745 280.1562

285.4834 297.1458 303.5069

314.3969 317.9947 326.2568

329.6583 331.1906 347.9326

348.4231 354.6118 358.9453

373.2083 394.0086 414.6694

416.9700 420.8736 422.6303

446.5592 460.5489 462.8621

473.5401 499.5127 502.8315

510.1212 516.8702 518.6639

522.7037 523.8256 535.3348

548.5045 549.0862 550.2531

558.8766 559.2621 570.7705

574.2418 580.8000 586.7105

587.9130 603.3772 605.1274

616.7444 628.3577 642.5139

647.3549 661.9181 667.9713

677.3636 677.7776 700.1257

713.4844 720.0820 723.6553

732.1491 735.8927 744.4148

747.6455 751.3546 755.3085

756.8073 759.9141 765.4395

784.9818 791.0512 793.6752

804.6394 805.1505 818.2111

834.9695 849.9310 856.5901

857.3489 858.7977 869.2567

873.8818 881.9753 884.1125

909.3687 914.9613 915.6735

918.7888 919.8253 924.2363

927.1860 930.4020 944.5485

963.5883 971.9404 973.3607

979.7073 982.2331 991.7673

994.5913 1001.5203 1001.7581

1009.0365 1013.2424 1014.3183

1033.5989 1036.4755 1037.8966

1039.2674 1040.9556 1051.3161

1054.9774 1058.2187 1059.4009

1064.8204 1066.0192 1066.8262

1072.7880 1074.6505 1076.5562

1095.1890 1108.2947 1113.3332

1116.8418 1119.0881 1124.2950

1128.9101 1154.9150 1156.6836

1157.5965 1168.9252 1173.2957

1178.0054 1182.6018 1190.3377

1197.4414 1203.1210 1206.3259

1207.2005 1227.9620 1239.9433

1241.9664 1250.5312 1259.2464

1262.9757 1276.6529 1280.3840

1280.9694 1282.5354 1288.3441

1298.5848 1301.0401 1308.0739

1311.8586 1318.0675 1319.7916

1329.2963 1332.9589 1337.4227

1340.9542 1343.6784 1358.0918

1360.3055 1364.7736 1368.9047

1374.5863 1381.1764 1386.7906

1387.7180 1401.8343 1402.6106

1411.6418 1416.7429 1420.8974

1425.7399 1429.1340 1446.1954

1459.9232 1468.2946 1469.4902

1471.8319 1478.0286 1488.6822

1493.4596 1495.8946 1497.3251

1497.8839 1497.9122 1503.4993

1504.8309 1505.4475 1516.9770

1520.1347 1520.9471 1525.2022

1525.8139 1532.6063 1534.4744

1538.5860 1542.6134 1545.9562

1569.7244 1594.3290 1655.6717

1662.0590 1664.0309 1674.4406

1679.6213 1683.7017 1685.0132

1686.8894 1689.2064 1689.9031

1691.4954 1692.3077 1746.2784

1843.9064 1896.6477 3038.1068

3053.0882 3054.1793 3072.7479

3075.3584 3075.6757 3078.5303

3099.0409 3119.1666 3138.7726

3146.8262 3147.0646 3148.7639

3156.1480 3163.4193 3166.2908

3171.6597 3173.8716 3178.1377

3179.5386 3199.8638 3200.3999

3207.7461 3209.4191 3209.6415

3209.7622 3218.6135 3219.5700

3222.0328 3224.5040 3226.0115

3227.4037 3229.9111 3230.8297

3233.3146 3240.9429 3243.3240

3247.3310 3249.7601 3309.0250

**TS5SS'**

Zero-point correction= 0.794506

Thermal correction to Energy= 0.844394

Thermal correction to Enthalpy= 0.845339

Thermal correction to Gibbs Free Energy= 0.713159

Sum of electronic and zero-point Energies= -5156.263034

Sum of electronic and thermal Energies= -5156.213145

Sum of electronic and thermal Enthalpies= -5156.212201

Sum of electronic and thermal Free Energies= -5156.344381

Cartesian coordinates

O 4.816146 2.894146 -0.278259

O 0.222024 -0.324925 0.624090

O -1.020487 1.544205 -2.649779

O 1.075608 2.163643 2.348772

N 2.016752 -1.762620 0.239370

C 1.597708 -2.830930 1.104403

H 0.687450 -2.507855 1.613699

H 2.370314 -3.036323 1.855656

C 2.102637 0.279148 -0.775495

C -1.404503 0.535929 -2.060041

C 3.250659 -3.100472 -3.721680

H 4.175314 -3.024775 -4.284074

C -0.756575 -0.734988 -2.036680

H -1.148350 -1.531122 -1.417059

C 3.232127 -1.688406 -0.407470

C 3.322869 -0.415193 -1.041394

C 0.470652 -0.831859 -2.644238

H 0.807918 0.045104 -3.191146

C 1.262383 -2.041173 -2.810097

C 4.495043 -0.102364 -1.741386

H 4.604869 0.866628 -2.221595

C 2.877181 -4.310546 -3.146009

C 1.696910 -4.392531 -2.403483

H 1.402790 -5.335441 -1.952200

C 2.451153 -1.976636 -3.552502

H 2.751226 -1.026112 -3.983684

C 1.323840 -0.554864 0.081623

C 1.852839 1.756175 -0.825937

H 2.251385 2.225742 -1.732964

H 0.777889 1.966792 -0.773624

C 5.531154 -1.033358 -1.787969

H 6.443619 -0.788836 -2.323445

C 0.898548 -3.270397 -2.236818

H -0.009830 -3.343885 -1.644461

C 5.416630 -2.276591 -1.154649

H 6.237277 -2.985286 -1.210032

C 4.020711 2.287255 0.398991

C 4.257791 -2.619863 -0.455676

H 4.157710 -3.585580 0.031215

C 2.494402 2.493072 0.372806

C 4.289881 1.197143 1.373714

C 2.057827 1.872120 1.710595

C 3.068387 -0.150227 2.987156

H 2.135590 -0.388427 3.490210

C 5.450922 0.451626 1.554800

H 6.342567 0.674210 0.978602

C 3.126006 0.911631 2.090063

C 4.222158 -0.908162 3.156603

H 4.215318 -1.754542 3.836568

C 5.402855 -0.603120 2.458453

H 6.282802 -1.218860 2.614308

H 3.503758 -5.188638 -3.267640

H 1.403379 -3.750089 0.538694

C -4.445050 -2.957857 0.732949

C -3.472810 -2.087348 0.252402

C -2.436714 -1.614030 1.048502

C -2.408033 -2.037867 2.370515

C -3.348892 -2.936700 2.874978

C -4.370148 -3.405765 2.050234

C -5.493488 -3.224259 -0.317782

C -5.269023 -2.130882 -1.392371

C -3.802808 -1.616856 -1.149337

H -1.663330 -0.937964 0.687497

H -3.294410 -3.245810 3.912724

H -5.115541 -4.086746 2.448939

H -6.517606 -3.166928 0.062645

H -5.366345 -2.528923 -2.402323

H -5.366370 -4.217320 -0.761284

H -3.124679 -1.980678 -1.923102

N -3.754625 -0.148861 -1.151538

O -6.234443 -1.094761 -1.331146

C -2.726841 0.707156 -1.327849

C -4.769119 0.560103 -0.566925

N -3.159392 1.878146 -0.857186

N -4.432946 1.802823 -0.384278

C -6.047525 -0.161226 -0.285649

H -6.005122 -0.645574 0.699925

H -6.884977 0.536099 -0.303390

C -2.358456 3.053275 -0.597492

C -1.668338 3.083864 0.616326

C -2.310330 4.064608 -1.554851

C -0.841327 4.186917 0.832367

C -1.480916 5.147249 -1.280556

C -0.725284 5.211199 -0.106705

H -0.251931 4.219010 1.743270

H -1.407554 5.950270 -2.009647

Br -1.091576 -1.302913 3.515218

C -3.092274 3.953245 -2.833820

H -2.692335 3.130309 -3.434532

H -4.149755 3.751403 -2.636773

H -3.020804 4.876485 -3.410866

C 0.230161 6.350087 0.131283

H 1.244491 6.043767 -0.140638

H -0.035262 7.226227 -0.464592

H 0.246064 6.637383 1.185437

C -1.772202 1.969875 1.625247

H -2.783661 1.551800 1.665584

H -1.059130 1.172026 1.387223

H -1.513396 2.338918 2.618294

F 2.177540 3.830819 0.310784

Vibrational frequencies

-34.6471 15.1400 32.1888

34.7876 38.5315 45.0940

48.7914 52.7253 55.4280

63.1922 67.6115 70.9154

74.8109 84.0212 86.8238

91.3205 97.3515 101.8292

112.1763 121.3945 122.2181

125.4940 126.8941 135.1141

138.9846 143.8945 149.9566

151.8815 158.9971 173.0547

176.7820 198.4875 203.7744

208.4585 211.1004 218.5991

221.8912 227.0306 231.8704

233.1472 240.3602 249.3312

254.9181 262.1478 265.4337

272.1013 283.7098 286.7217

288.3804 291.6417 298.2888

299.7660 328.7012 332.6216

341.2940 344.1330 353.8104

363.3031 365.4588 385.8036

412.5676 416.7811 423.1474

426.1940 443.5770 446.4624

454.6010 461.0460 472.4001

482.9386 494.6446 500.9724

515.0240 516.7795 518.1835

526.3852 527.0169 533.6373

541.7201 559.4693 560.1647

569.4285 576.9350 580.4127

590.6836 593.2090 611.8416

620.4522 625.8328 630.5014

631.2268 652.6010 662.7855

673.9962 680.8086 697.5038

703.4409 717.7114 721.7497

728.3689 732.3462 743.8039

749.5864 753.2290 756.4284

759.6490 760.9341 772.1251

789.8252 795.9292 800.0306

803.3897 817.6759 824.2753

834.3476 845.4160 851.3071

853.4494 866.9901 868.7342

870.1748 870.9350 890.9911

899.1412 903.8799 912.6893

916.9401 921.3065 924.4204

925.6467 956.3258 962.9424

966.2841 968.2406 977.2243

981.6015 982.6637 985.0907

990.2751 991.7179 1003.9232

1007.1458 1016.3074 1020.9474

1026.9972 1039.1793 1045.3630

1046.9689 1048.9344 1050.2091

1054.1933 1055.3028 1059.0829

1064.4675 1066.4831 1067.7587

1068.4482 1072.8483 1084.8676

1113.2347 1114.1309 1114.8257

1118.0438 1127.9021 1129.1732

1131.1781 1152.3483 1154.0874

1157.0151 1167.7100 1174.5503

1175.3144 1183.0463 1195.8794

1203.3101 1207.4124 1214.0536

1220.8521 1228.8460 1236.8262

1238.2630 1240.6177 1250.8563

1267.8052 1272.4918 1276.3117

1280.2403 1291.1791 1303.0765

1306.2670 1312.9236 1317.7744

1320.5015 1327.7245 1330.8626

1336.4222 1345.0380 1351.0877

1358.6329 1363.0265 1367.4536

1367.6561 1371.9842 1379.9302

1388.7757 1394.5005 1408.0473

1411.2193 1418.2498 1420.5809

1426.8715 1427.5758 1443.9047

1462.0831 1462.2215 1474.0858

1474.6528 1478.8558 1484.8300

1487.0648 1491.6273 1495.7610

1497.7332 1498.4697 1498.9050

1503.1142 1508.0697 1513.3118

1515.1615 1517.0397 1523.8013

1528.1756 1536.3322 1538.3263

1538.9513 1544.6619 1548.4154

1559.8406 1591.2414 1655.5011

1665.3193 1673.3028 1678.6060

1684.1694 1691.4501 1692.2189

1693.0629 1693.3442 1694.3760

1697.9476 1700.8514 1740.0408

1870.6713 1910.6295 3046.0267

3062.7832 3065.4005 3071.0398

3073.8689 3079.0256 3091.4386

3107.0848 3128.0908 3137.4239

3140.4627 3145.1395 3149.6273

3153.5045 3162.1433 3169.1013

3170.0890 3170.3629 3173.7726

3180.1377 3188.3673 3189.2240

3192.2204 3195.3510 3198.5644

3202.2199 3203.2195 3206.8429

3211.4933 3212.1997 3212.7905

3218.7829 3220.1778 3221.8186

3224.6477 3225.4755 3227.4803

3241.3785 3242.7405 3244.0375

**TS5RR'**

Zero-point correction= 0.793642

Thermal correction to Energy= 0.844469

Thermal correction to Enthalpy= 0.845414

Thermal correction to Gibbs Free Energy= 0.707748

Sum of electronic and zero-point Energies= -5156.267430

Sum of electronic and thermal Energies= -5156.216602

Sum of electronic and thermal Enthalpies= -5156.215658

Sum of electronic and thermal Free Energies= -5156.353323

Cartesian coordinates

O -1.519093 -0.197899 -3.418533

O -1.517698 0.824745 -0.490948

O 2.063589 -2.561154 0.140729

O -3.666224 -3.996445 -1.792520

N -3.235078 0.211934 0.937563

C -3.732739 1.534697 1.217975

H -3.090462 2.238869 0.686117

H -3.686103 1.740230 2.292348

C -1.846815 -1.480182 0.233498

C 1.826542 -1.341559 0.158352

C -2.114633 -0.422121 4.761980

H -2.661144 -0.973752 5.520258

C 0.859337 -0.596331 0.838422

H 0.852363 0.472934 0.696877

C -3.724389 -0.968221 1.482357

C -2.926411 -2.043167 1.030418

C -0.162148 -1.183449 1.621841

H -0.074511 -2.256699 1.787663

C -0.796987 -0.427245 2.720622

C -3.255995 -3.341021 1.412201

H -2.672036 -4.185223 1.056069

C -2.029226 0.965227 4.830144

C -1.332523 1.661425 3.840432

H -1.253086 2.744208 3.873988

C -1.502587 -1.111400 3.717934

H -1.579916 -2.193942 3.663362

C -2.134646 -0.044286 0.134291

C -1.177312 -2.198801 -0.899527

H -0.227905 -1.714589 -1.154531

H -0.957843 -3.237608 -0.627832

C -4.359991 -3.543461 2.243689

H -4.626792 -4.551420 2.543435

C -0.728147 0.974446 2.796410

H -0.217961 1.543740 2.025692

C -5.126229 -2.464521 2.690557

H -5.977667 -2.644295 3.339219

C -2.290703 -0.861145 -2.764689

C -4.820258 -1.155102 2.310842

H -5.416509 -0.314881 2.652454

C -1.995618 -2.259965 -2.199778

C -3.678710 -0.522682 -2.361120

C -3.398189 -2.828688 -1.930059

C -5.599619 -1.594405 -1.326055

H -6.067989 -2.479988 -0.909215

C -4.308206 0.718339 -2.361199

H -3.791312 1.597461 -2.730949

C -4.315413 -1.659860 -1.856738

C -6.230219 -0.355100 -1.321177

H -7.228052 -0.261310 -0.905180

C -5.594517 0.786011 -1.837600

H -6.115809 1.737682 -1.812029

H -2.499372 1.502448 5.647825

H -4.764463 1.643020 0.867021

C 0.270899 3.278023 -2.277415

C 0.879312 2.461554 -1.327124

C 1.075776 2.883685 -0.020585

C 0.570428 4.128255 0.335721

C -0.057155 4.959840 -0.588017

C -0.192462 4.537026 -1.910010

C 0.183913 2.570176 -3.608385

C 1.116493 1.351601 -3.462556

C 1.145607 1.099905 -1.926967

H 1.591346 2.276622 0.716606

H -0.432375 5.926964 -0.273986

H -0.667750 5.187761 -2.637430

H 0.485494 3.186315 -4.459180

H 0.723096 0.478210 -3.981559

H -0.835749 2.209703 -3.781913

H 0.332510 0.428996 -1.627471

N 2.424819 0.525226 -1.526510

O 2.408943 1.581108 -4.028227

C 2.748500 -0.467998 -0.678603

C 3.585254 0.977771 -2.091250

N 4.072878 -0.573127 -0.759547

N 4.615369 0.322345 -1.639387

C 3.442574 2.010486 -3.163132

H 3.230322 2.991107 -2.717025

H 4.358974 2.074101 -3.749515

C 4.920986 -1.415610 0.042758

C 5.336845 -0.921437 1.278003

C 5.285028 -2.665650 -0.456389

C 6.184586 -1.731421 2.031902

C 6.134441 -3.435166 0.333606

C 6.592515 -2.984728 1.574454

H 6.529977 -1.375003 2.998786

H 6.439241 -4.415135 -0.024717

Br 0.708180 4.665415 2.148068

C 4.735001 -3.154725 -1.766295

H 3.649232 -3.263211 -1.674554

H 4.943517 -2.447761 -2.574962

H 5.166709 -4.120873 -2.030936

C 7.485511 -3.857901 2.417211

H 6.893998 -4.609020 2.950118

H 8.213035 -4.389885 1.799556

H 8.027350 -3.270253 3.161053

C 4.863048 0.419798 1.772923

H 5.077265 1.209664 1.046148

H 3.780346 0.406557 1.945573

H 5.351057 0.677447 2.713706

F -1.305988 -3.016856 -3.113926

Vibrational frequencies

-226.6158 14.1669 20.0206

25.6030 30.5783 33.5032

36.4477 40.6799 47.2518

50.3499 52.9507 59.7593

61.3019 63.6831 65.9379

74.2465 82.5322 86.2218

92.3863 100.5242 103.3176

113.4789 116.4678 129.0828

133.1587 135.5678 142.2474

145.1071 165.1320 174.1977

179.3251 186.1674 189.0662

196.9981 199.6500 200.9499

203.6002 216.7949 227.2468

235.4476 237.6224 238.7206

250.4678 257.1895 260.9599

266.3413 276.7680 281.7752

288.3652 293.9396 298.0483

303.5081 308.6782 322.6986

325.4997 339.8167 354.8926

361.3186 364.7839 383.3118

403.2741 414.0734 416.7983

421.5448 428.6760 444.2365

447.8622 457.7857 474.9140

484.2154 494.8034 499.7572

508.2144 515.6341 520.5945

524.0556 534.2689 540.4221

545.0000 553.9481 559.3483

564.8657 583.0949 585.8945

587.9345 590.8593 609.0997

617.6886 628.9056 630.5704

638.1925 658.2512 663.5025

673.6192 687.2536 699.7307

705.8315 715.4672 718.7884

725.5891 734.9057 744.7323

750.1219 752.3514 756.7095

759.9004 767.4959 779.0243

787.0774 791.8714 793.7996

797.3771 808.9052 824.9863

835.4283 845.4322 846.9307

861.1389 864.2927 873.2102

874.4474 881.7194 883.6805

905.6664 906.5889 910.5633

915.8901 922.8424 930.2379

941.9265 942.3513 964.6740

967.3861 968.2206 981.3996

987.4873 988.3571 988.4373

990.6450 999.2918 1012.3316

1014.9495 1021.4567 1023.7783

1026.2698 1037.1321 1040.7368

1044.6084 1048.8798 1053.6090

1056.6542 1058.9054 1063.0774

1064.0093 1066.8915 1067.3898

1070.3195 1073.9668 1094.4921

1111.3872 1114.4089 1115.5042

1121.4638 1125.7031 1131.4304

1142.2101 1150.4125 1154.8973

1156.1365 1167.7257 1175.0097

1176.6519 1177.1882 1188.8986

1202.9984 1204.7286 1205.2227

1224.5869 1226.3977 1227.3763

1229.4007 1235.4909 1248.0069

1259.6371 1269.8895 1274.6803

1277.7681 1281.8001 1283.1126

1293.3205 1299.9535 1303.4874

1315.0534 1318.8482 1322.3084

1333.7695 1338.7547 1352.8453

1355.8666 1358.9960 1362.8227

1363.7059 1367.6858 1377.7576

1381.3902 1384.1272 1398.2937

1412.0270 1412.5235 1420.2497

1421.5430 1429.1301 1433.1108

1462.5528 1463.3529 1467.2032

1469.6141 1470.7684 1474.9762

1490.5179 1493.9258 1496.7643

1497.7297 1499.2431 1500.0552

1501.5310 1506.7033 1511.1861

1514.3409 1520.5626 1522.5373

1527.3887 1529.6129 1530.0491

1537.2721 1543.5425 1547.2185

1567.3914 1583.7988 1662.1502

1667.6328 1669.1666 1672.3819

1678.2205 1686.2235 1687.2100

1689.3944 1689.7410 1690.2210

1693.7128 1711.5448 1751.7244

1870.3096 1912.9148 3061.7077

3064.2366 3066.4519 3073.5721

3078.0818 3078.5147 3094.8918

3112.6665 3130.8327 3131.2715

3136.1314 3146.2493 3148.6418

3151.6655 3171.2718 3174.5467

3178.2030 3179.1372 3179.2479

3180.7640 3184.2948 3206.5096

3207.7384 3207.9734 3208.7546

3213.2851 3214.9569 3221.2573

3222.6570 3223.6942 3226.6931

3230.7029 3232.5094 3233.0586

3236.3931 3237.6880 3241.7514

3245.7174 3247.3099 3319.9081

**M6SS**

Zero-point correction= 0.813212

Thermal correction to Energy= 0.864645

Thermal correction to Enthalpy= 0.865589

Thermal correction to Gibbs Free Energy= 0.727599

Sum of electronic and zero-point Energies= -5394.021220

Sum of electronic and thermal Energies= -5393.969787

Sum of electronic and thermal Enthalpies= -5393.968843

Sum of electronic and thermal Free Energies= -5394.106833

Cartesian coordinates

O 1.890786 -1.026965 -0.397832

O 1.683649 2.962142 1.761591

O -0.271090 -0.950599 -1.401744

O 4.613402 2.807582 0.541995

N 0.780261 4.787551 0.689334

C 0.746289 5.689522 1.816277

H 1.213771 5.176662 2.656651

H -0.287639 5.945316 2.068154

C 1.025829 2.833765 -0.605358

C 0.399482 -0.882343 -0.314569

C -3.687633 2.387245 -0.528119

H -4.491898 2.351745 -1.257183

C 0.624177 0.514773 0.401558

H 0.322039 0.604776 1.445307

C 0.288654 5.080867 -0.590482

C 0.424657 3.958153 -1.412688

C 0.047179 1.640117 -0.458108

H -0.062036 1.217038 -1.464336

C -1.342349 2.044704 0.000231

C 0.007183 4.012053 -2.728990

H 0.100963 3.144418 -3.377026

C -3.954213 2.705760 0.799781

C -2.913108 2.715355 1.726670

H -3.109760 2.974453 2.763101

C -2.393332 2.055184 -0.919897

H -2.199718 1.759623 -1.948624

C 1.236897 3.498892 0.759365

C 2.334172 2.331452 -1.234690

H 2.082554 1.960985 -2.233938

H 3.043437 3.157238 -1.351882

C -0.549358 5.202941 -3.211996

H -0.883251 5.261850 -4.242172

C -1.616709 2.395665 1.329111

H -0.811518 2.413414 2.061786

C -0.679615 6.310030 -2.378535

H -1.113410 7.225609 -2.767375

C 2.127982 0.196911 0.298662

C -0.259912 6.268666 -1.045141

H -0.360507 7.131860 -0.395701

C 3.034259 1.218196 -0.446652

C 2.823016 0.004145 1.633439

C 3.948034 1.807738 0.664175

C 4.584251 0.962646 3.016661

H 5.367471 1.703014 3.143077

C 2.520812 -0.922456 2.626354

H 1.732971 -1.658212 2.486685

C 3.843796 0.922728 1.839637

C 4.287444 0.030685 4.003072

H 4.847821 0.022728 4.932018

C 3.262964 -0.903823 3.805421

H 3.045462 -1.626728 4.585703

C 3.962118 0.465478 -1.400099

F 3.324486 -0.030677 -2.462049

F 4.584314 -0.549622 -0.778914

F 4.918269 1.284676 -1.872634

H -4.966107 2.943891 1.112207

H 1.301288 6.604448 1.591465

C -4.289214 -0.980536 2.052492

C -3.164225 -0.973234 1.234095

C -3.243029 -0.867735 -0.148745

C -4.515559 -0.739689 -0.689011

C -5.664990 -0.740683 0.099193

C -5.554002 -0.869077 1.482006

C -3.904756 -1.090089 3.506540

C -2.381273 -1.370587 3.511628

C -1.898615 -1.042174 2.052406

H -2.348077 -0.845149 -0.773033

H -6.637021 -0.636399 -0.369627

H -6.447159 -0.874681 2.098964

H -4.431105 -1.889125 4.037967

H -1.859180 -0.733378 4.226062

H -4.116827 -0.155107 4.035671

H -1.373519 -0.087914 2.032163

N -0.999898 -2.083405 1.550857

O -2.063478 -2.688983 3.928238

C -0.053583 -2.055241 0.591030

C -1.227979 -3.399704 1.864636

N 0.258298 -3.335343 0.378427

N -0.464506 -4.187389 1.173901

C -2.229614 -3.682984 2.935442

H -3.246841 -3.678293 2.518016

H -2.036813 -4.653546 3.392008

C 1.053904 -3.888423 -0.692735

C 0.373210 -4.224483 -1.868099

C 2.414136 -4.119295 -0.500860

C 1.120348 -4.799999 -2.891604

C 3.114747 -4.698458 -1.558689

C 2.489758 -5.036572 -2.757233

H 0.618645 -5.069446 -3.817662

H 4.178503 -4.887141 -1.437479

Br -4.671602 -0.463665 -2.555229

C 3.117119 -3.733834 0.768507

H 3.374484 -2.671387 0.729196

H 2.485864 -3.897129 1.646957

H 4.034060 -4.313474 0.891048

C 3.279118 -5.626276 -3.896821

H 3.610547 -4.837968 -4.580306

H 4.168515 -6.147292 -3.535307

H 2.675068 -6.330562 -4.473886

C -1.100865 -3.962673 -2.012229

H -1.668843 -4.457211 -1.217298

H -1.277265 -2.883367 -1.950912

H -1.463375 -4.331838 -2.972903

Vibrational frequencies

13.1864 20.1881 24.7943

25.4437 29.4840 37.3328

45.1184 50.7704 53.3294

57.2559 59.1666 62.3030

71.3376 72.5341 81.4095

84.5919 92.2254 98.2524

101.9818 116.5641 118.8616

126.1540 131.7868 139.2445

145.2880 162.4008 165.9442

169.1143 171.1831 176.2536

180.5758 185.7751 190.0863

193.0405 197.6712 202.6824

208.8446 223.0869 233.4665

239.4169 247.3055 250.3447

255.8892 269.8581 272.3998

280.1939 283.0159 283.8767

302.9760 305.3337 317.6836

318.8925 324.9938 331.8037

336.2745 341.4509 354.5179

365.0232 381.3026 393.8997

398.6202 407.7870 418.1420

421.1784 437.8667 443.4411

461.9090 466.8230 476.3012

487.8540 497.7982 502.8031

512.5632 515.6619 520.4779

522.5976 525.0720 530.0340

540.4654 551.2229 552.7949

558.5707 564.6977 572.7876

590.7959 591.1766 593.1281

600.9976 617.0936 622.3495

623.8858 627.5477 629.3435

636.1872 660.1908 663.1091

672.7370 697.1786 712.0402

715.9265 719.1044 720.3915

727.6292 734.8073 748.3810

754.0023 754.9349 768.4623

772.1472 775.5547 780.5694

788.4512 789.8542 800.5449

807.2875 833.8766 839.0376

847.3033 855.1490 858.1583

872.0395 873.1850 880.5281

880.9253 883.1400 891.6829

906.4279 906.7693 912.3793

914.2487 931.5112 939.9991

950.9411 957.6073 962.4220

965.4559 970.6815 982.3773

984.0782 985.7627 999.4043

1004.7962 1005.0893 1013.3165

1016.0653 1017.3383 1023.0607

1026.5175 1029.2421 1038.9203

1042.0168 1044.3699 1054.8832

1057.8431 1061.0412 1061.9587

1063.8793 1065.0032 1068.7053

1072.1938 1073.4463 1074.5481

1078.0013 1096.9337 1113.6223

1121.5096 1122.0336 1126.0163

1132.5765 1137.8968 1146.0853

1154.1620 1156.3707 1157.2464

1167.6461 1174.8527 1177.2121

1180.8491 1183.7236 1195.2653

1196.1437 1203.7557 1207.8989

1211.9135 1217.8300 1220.2574

1233.3465 1243.3986 1247.6472

1250.5434 1258.9268 1265.5141

1268.6059 1276.9820 1282.2527

1283.6859 1286.4543 1296.9997

1301.2827 1303.5961 1317.3284

1318.4493 1322.4106 1323.4805

1328.5210 1331.2572 1339.9636

1340.9663 1350.6064 1354.5119

1358.3274 1364.1155 1369.7054

1371.4232 1383.7937 1385.3145

1386.2201 1403.1174 1412.2386

1413.1717 1419.6035 1419.8104

1427.6336 1437.1269 1452.9949

1459.2479 1462.8947 1472.4507

1476.2815 1484.9745 1487.7169

1489.2467 1492.3285 1497.6451

1498.2143 1503.2562 1506.0402

1506.6281 1509.9866 1513.2071

1515.8687 1523.8139 1527.6615

1531.1502 1532.4011 1545.1016

1546.6607 1549.7549 1560.0046

1574.1880 1672.3208 1675.5981

1689.5117 1693.0492 1693.9007

1695.3732 1696.3047 1698.3535

1703.2735 1703.8848 1707.9129

1816.3371 1880.0372 3065.7124

3066.5218 3067.8812 3070.0846

3074.0273 3083.7386 3086.0670

3086.2705 3136.7657 3138.0397

3138.8895 3139.5462 3141.9716

3145.4220 3150.6863 3154.7282

3160.9096 3163.8470 3164.5752

3175.4252 3176.5308 3180.6033

3185.9767 3191.1299 3194.0450

3195.3823 3195.5055 3198.9390

3204.3863 3204.4356 3210.2546

3211.0523 3218.0173 3219.9526

3224.5314 3225.4543 3229.3087

3232.6781 3240.4269 3247.6712

**TS6SS**

Zero-point correction= 0.810935

Thermal correction to Energy= 0.862554

Thermal correction to Enthalpy= 0.863499

Thermal correction to Gibbs Free Energy= 0.725850

Sum of electronic and zero-point Energies= -5393.991265

Sum of electronic and thermal Energies= -5393.939645

Sum of electronic and thermal Enthalpies= -5393.938701

Sum of electronic and thermal Free Energies= -5394.076350

Cartesian coordinates

O -1.294575 2.561551 -1.597456

O -2.516187 -0.755505 1.895135

O 0.663816 0.469891 -2.081231

O -4.231904 1.602675 2.038805

N -3.854499 -2.396470 1.015025

C -4.049880 -3.152841 2.229344

H -3.573859 -2.599979 3.038646

H -3.592227 -4.143116 2.138690

C -3.096623 -0.770529 -0.522130

C 0.441980 0.570054 -0.881383

C 0.045211 -3.708149 -2.580788

H 0.309677 -4.076286 -3.567224

C -0.828693 0.341001 -0.263925

H -0.835973 0.128916 0.795271

C -4.319488 -2.783798 -0.248554

C -3.890000 -1.861592 -1.205454

C -1.660009 -0.641581 -1.076862

H -1.751061 -0.217751 -2.085673

C -0.958991 -1.986933 -1.197070

C -4.226447 -2.039951 -2.534002

H -3.894911 -1.331446 -3.288973

C 0.372451 -4.447605 -1.448485

C 0.038517 -3.956976 -0.188084

H 0.312854 -4.515985 0.702256

C -0.615394 -2.488031 -2.453160

H -0.857895 -1.903894 -3.336469

C -3.100445 -1.255436 0.943501

C -3.836429 0.565348 -0.696598

H -3.977988 0.702427 -1.773436

H -4.829703 0.499099 -0.238879

C -4.993199 -3.155491 -2.892641

H -5.261111 -3.312996 -3.931723

C -0.617380 -2.735632 -0.062909

H -0.838111 -2.348987 0.929539

C -5.412302 -4.064271 -1.925161

H -6.006639 -4.923712 -2.217960

C -1.614125 2.098364 -0.471964

C -5.081655 -3.893142 -0.577384

H -5.408501 -4.601464 0.176539

C -3.147172 1.810368 -0.143502

C -1.138228 2.763520 0.824359

C -3.261432 1.920138 1.390316

C -1.790590 3.091790 3.151821

H -2.517258 2.939250 3.943918

C -0.007593 3.551023 1.013570

H 0.658024 3.757854 0.180310

C -2.028558 2.575639 1.880289

C -0.630543 3.833846 3.350429

H -0.411592 4.257261 4.325711

C 0.245938 4.069128 2.282582

H 1.133419 4.672198 2.450363

C -3.920447 3.009936 -0.705696

F -3.937341 3.030873 -2.040868

F -3.421854 4.181057 -0.284308

F -5.207921 2.972494 -0.307481

H 0.897242 -5.393283 -1.542880

H -5.116750 -3.266457 2.440440

C 2.426800 -1.098719 3.753679

C 1.917305 -0.877759 2.478868

C 2.188284 -1.732579 1.417567

C 3.014772 -2.820584 1.664157

C 3.517575 -3.082588 2.939066

C 3.215226 -2.222539 3.992289

C 2.042289 0.020062 4.687623

C 1.516799 1.152992 3.769988

C 1.210070 0.457119 2.392496

H 1.792918 -1.561073 0.420015

H 4.159914 -3.941784 3.095597

H 3.623828 -2.414358 4.979506

H 2.876292 0.384964 5.294635

H 0.601372 1.600931 4.155757

H 1.253878 -0.298878 5.376806

H 0.131874 0.390336 2.253530

N 1.810699 1.194643 1.267337

O 2.419286 2.239477 3.667443

C 1.591129 1.130535 -0.064922

C 3.018229 1.808331 1.441555

N 2.663072 1.694545 -0.625815

N 3.553516 2.137485 0.302140

C 3.529925 2.007828 2.831144

H 4.113694 1.131661 3.148947

H 4.171676 2.888434 2.866975

C 3.065937 1.728138 -2.016450

C 3.768602 0.622723 -2.496501

C 2.754157 2.846210 -2.783459

C 4.233506 0.696218 -3.807012

C 3.238901 2.865875 -4.089898

C 3.990522 1.812111 -4.610216

H 4.784776 -0.147085 -4.215609

H 3.010064 3.722674 -4.718339

Br 3.510768 -3.915305 0.207347

C 1.867778 3.930704 -2.240015

H 0.861066 3.530362 -2.050197

H 2.267925 4.337103 -1.304662

H 1.781191 4.750760 -2.954506

C 4.536914 1.879004 -6.012633

H 4.620052 0.882644 -6.452520

H 3.898697 2.488671 -6.655838

H 5.536081 2.327049 -6.015719

C 3.922464 -0.629372 -1.673818

H 4.215198 -0.418766 -0.639536

H 2.969650 -1.174535 -1.654675

H 4.672436 -1.292475 -2.108055

Vibrational frequencies

-173.7790 19.5431 20.6513

27.9712 29.2635 34.5649

36.7058 44.2097 46.8785

51.4715 57.5658 62.8714

64.5285 69.1525 75.8349

77.7934 83.8731 85.5554

99.8766 104.6284 115.8912

120.3335 125.7104 129.5598

135.5643 145.8743 155.4550

159.2452 164.9963 175.3318

179.3384 182.9406 188.7328

196.5619 204.5241 209.0825

213.0869 219.9585 230.7175

236.2510 238.9997 240.8664

245.0348 256.1994 265.4668

269.9816 281.3153 283.3939

292.8408 296.5815 297.0018

307.8741 311.6898 321.6600

326.6582 332.6681 337.6845

347.4490 353.2062 357.5543

374.1191 395.3095 399.9047

413.3111 424.0387 427.1461

439.1202 449.1461 459.1391

469.3497 474.5558 487.2368

497.9958 506.3113 515.5143

518.5938 520.6190 529.1477

535.9240 540.9741 549.7428

551.3323 556.2699 558.7494

566.0459 568.9378 573.1549

589.5341 593.1529 609.4923

613.0402 624.0868 628.8228

631.4177 642.3403 652.8657

660.9748 664.0136 683.0034

699.4023 709.8812 715.9875

721.6847 722.7391 729.0056

730.2061 754.2079 759.0198

764.0565 771.9097 776.4816

781.4480 786.3190 795.8600

800.0209 802.2428 815.9238

820.6549 837.2383 848.7822

851.9328 859.8697 872.4250

883.0884 885.6050 888.8371

891.0099 893.6218 901.0953

917.9100 918.6256 925.3807

926.4268 935.9561 937.3796

948.8570 963.9726 967.3488

970.3610 982.4594 988.5322

995.2844 1000.5443 1001.1352

1007.0546 1007.6311 1015.7655

1021.2606 1022.4770 1028.0208

1034.0272 1040.8123 1045.8224

1050.8415 1056.6758 1058.9892

1061.6118 1062.2667 1065.2200

1068.4870 1072.6591 1076.7699

1084.4948 1086.9906 1098.0706

1107.6105 1115.6581 1118.7827

1124.3823 1131.2644 1147.7027

1150.8522 1156.8268 1157.7629

1159.5294 1167.1356 1178.0358

1179.3632 1184.5284 1186.9438

1190.7161 1193.3889 1206.8664

1211.4614 1220.0074 1221.4835

1229.1106 1243.0076 1246.2357

1248.8021 1256.5399 1260.0108

1263.8456 1281.0168 1282.1146

1287.1859 1294.8087 1296.4033

1297.7299 1303.8441 1309.8259

1315.4244 1321.6714 1326.3833

1334.7639 1340.9278 1343.7043

1344.3416 1357.7399 1361.7767

1365.1858 1366.3620 1372.1199

1383.7957 1387.8359 1393.1708

1411.3410 1413.3778 1426.6836

1433.0488 1435.1509 1440.0083

1448.2398 1462.1981 1463.0432

1471.2511 1472.2561 1481.7458

1490.5667 1492.6259 1497.5729

1499.1972 1500.5609 1504.3808

1504.4945 1505.2180 1510.3511

1510.6378 1511.7110 1515.8934

1516.5677 1530.1921 1532.0748

1539.5013 1545.6716 1550.0257

1559.7686 1564.0837 1567.8701

1672.4096 1675.2449 1678.2443

1684.3382 1693.2017 1694.1051

1696.2381 1697.0430 1697.3233

1699.0592 1707.3425 1762.8307

1804.1211 1875.7476 3041.6202

3050.0758 3057.5401 3069.5489

3072.2699 3078.3452 3086.4579

3087.5090 3120.9047 3121.2979

3134.6689 3140.0120 3141.7964

3142.1315 3165.1212 3165.6110

3174.3733 3176.2695 3182.0115

3183.2940 3189.0559 3189.9774

3197.5348 3197.9756 3199.0919

3202.3355 3206.3202 3209.8114

3213.8951 3215.5952 3216.3614

3217.6371 3220.6800 3221.0296

3223.0108 3226.5847 3230.7441

3231.5824 3238.7784 3292.3853

**TS7SS**

Zero-point correction= 0.811518

Thermal correction to Energy= 0.863048

Thermal correction to Enthalpy= 0.863992

Thermal correction to Gibbs Free Energy= 0.725633

Sum of electronic and zero-point Energies= -5394.010403

Sum of electronic and thermal Energies= -5393.958874

Sum of electronic and thermal Enthalpies= -5393.957930

Sum of electronic and thermal Free Energies= -5394.096289

Cartesian coordinates

O -0.302665 -2.177537 -0.444510

O 3.210440 -0.556041 1.887571

O -0.975084 -0.283223 -1.606964

O 4.126512 -3.437174 0.886473

N 4.758203 0.816124 0.873787

C 5.511366 1.183917 2.050088

H 5.085755 0.638873 2.892499

H 5.433034 2.260528 2.229646

C 3.093430 -0.094437 -0.522141

C -0.562974 -0.789880 -0.575899

C 1.299424 4.265075 -0.658072

H 1.138012 5.023955 -1.417728

C 0.677164 -0.411539 0.264184

H 0.469494 -0.004374 1.250266

C 4.995333 1.306116 -0.418640

C 4.042584 0.784022 -1.299152

C 1.650035 0.473921 -0.525039

H 1.329437 0.442727 -1.574227

C 1.570956 1.924300 -0.081075

C 4.078832 1.122760 -2.638330

H 3.342625 0.725126 -3.332238

C 1.345806 4.614903 0.687611

C 1.525133 3.623195 1.650200

H 1.571342 3.888225 2.702528

C 1.409629 2.929625 -1.036238

H 1.330019 2.659456 -2.086226

C 3.671241 -0.013565 0.894939

C 3.077149 -1.527908 -1.073722

H 2.723310 -1.468727 -2.108562

H 4.091759 -1.938587 -1.093420

C 5.078248 1.995252 -3.085888

H 5.120549 2.273416 -4.133247

C 1.642873 2.289229 1.269700

H 1.790654 1.526041 2.030777

C 6.016173 2.509730 -2.195012

H 6.783098 3.186823 -2.556778

C 0.890753 -1.943088 0.337548

C 5.991879 2.171776 -0.837884

H 6.723177 2.572535 -0.144062

C 2.193367 -2.507564 -0.291470

C 0.834519 -2.573156 1.711749

C 2.961706 -3.122981 0.913640

C 2.196147 -3.872335 3.258415

H 3.131515 -4.378411 3.474074

C -0.202748 -2.517844 2.636745

H -1.126191 -1.996880 2.403673

C 2.008340 -3.243909 2.031727

C 1.153802 -3.829253 4.176094

H 1.257160 -4.314141 5.141162

C -0.033627 -3.156350 3.863695

H -0.837824 -3.130578 4.592612

C 1.853875 -3.680474 -1.212319

F 1.246165 -3.293821 -2.336997

F 1.062628 -4.572449 -0.598661

F 2.973747 -4.325078 -1.579862

H 1.239550 5.652885 0.986696

H 6.563743 0.911548 1.931483

C -2.359325 3.791559 1.922849

C -1.964983 2.659489 1.216996

C -1.748996 2.677391 -0.155291

C -1.924748 3.893866 -0.801893

C -2.307515 5.049441 -0.123018

C -2.530237 4.998855 1.251191

C -2.548725 3.477035 3.385442

C -2.489660 1.931491 3.482329

C -1.857895 1.456136 2.124568

H -1.448461 1.789403 -0.710770

H -2.434685 5.976485 -0.670621

H -2.841425 5.893074 1.782658

H -3.498774 3.839941 3.789566

H -1.867989 1.608645 4.318243

H -1.749488 3.923273 3.987118

H -0.818130 1.154427 2.272057

N -2.591154 0.329933 1.563612

O -3.755096 1.347778 3.747182

C -2.172424 -0.603434 0.668186

C -3.957566 0.278060 1.671304

N -3.325902 -1.179324 0.304483

N -4.445749 -0.656172 0.917962

C -4.608744 1.227649 2.622872

H -4.778578 2.200306 2.138866

H -5.563636 0.833429 2.970208

C -3.505162 -2.137131 -0.753973

C -3.936973 -1.643794 -1.991596

C -3.263984 -3.489192 -0.520610

C -4.134344 -2.563420 -3.015921

C -3.474183 -4.371283 -1.582535

C -3.903640 -3.929038 -2.830644

H -4.470094 -2.204897 -3.986021

H -3.294059 -5.431728 -1.423229

Br -1.596328 3.975549 -2.663098

C -2.762086 -3.992560 0.803550

H -1.669424 -3.931569 0.827622

H -3.152316 -3.402197 1.637249

H -3.051235 -5.035030 0.952505

C -4.091795 -4.895822 -3.971075

H -3.215399 -4.891105 -4.627075

H -4.229387 -5.916309 -3.606977

H -4.959066 -4.625738 -4.578873

C -4.156648 -0.170088 -2.201921

H -4.898984 0.224641 -1.502445

H -3.214860 0.366361 -2.045977

H -4.500937 0.023241 -3.219423

Vibrational frequencies

-170.1353 17.2522 18.6939

25.1091 28.1844 33.0080

35.2198 44.6046 49.2157

53.0346 58.1957 58.4037

61.6514 64.3259 71.8756

80.9276 87.0634 92.2274

96.9790 109.4251 114.0200

117.1783 122.9193 126.8227

138.7654 140.8862 143.7623

146.2721 152.5237 157.6624

167.7578 171.4467 176.7825

183.0756 188.3138 195.7625

200.3990 207.8178 210.4346

215.9455 238.5690 238.9601

249.3700 255.3161 264.8170

271.2322 274.8848 285.0350

290.9128 294.6421 307.2069

318.0936 318.9177 329.7307

334.5989 340.1294 349.4533

354.6775 364.1816 380.9555

396.6143 409.3677 415.7356

422.5852 426.4382 439.0448

447.0659 456.8481 475.2992

481.8828 494.9050 499.7076

503.2943 514.2949 516.8584

518.7112 524.3349 526.0371

542.0977 548.4350 551.0924

556.5970 559.6136 569.6801

577.3905 581.4351 590.5285

598.3829 612.1908 618.0100

619.8510 623.9495 626.8946

632.8030 658.6185 659.5310

663.2807 685.2445 702.2556

712.3003 715.7835 717.2418

723.2985 726.4943 731.5485

749.5089 754.6309 758.2029

773.5461 775.2190 779.1796

782.7175 789.1144 792.7776

808.1686 811.4630 840.2971

843.2331 847.3914 859.7608

868.2388 870.8076 882.9187

885.4772 889.2524 897.6327

901.5158 913.1614 913.8993

917.1714 936.5683 952.9381

957.0789 966.3991 969.0228

970.6979 980.7268 988.6617

989.5552 990.7242 996.7094

999.5656 1003.7406 1007.0528

1009.6951 1014.7238 1017.4979

1022.8393 1025.5812 1027.8375

1030.3276 1042.6006 1044.8300

1052.3714 1055.1695 1056.3766

1061.5904 1063.2029 1066.6703

1067.2359 1070.7682 1072.9246

1078.7520 1091.7138 1097.2264

1114.8676 1121.1308 1122.4674

1126.0606 1130.2215 1139.3944

1151.7258 1151.9563 1155.8220

1168.3813 1169.8925 1175.0475

1179.2922 1182.5366 1191.1010

1191.9732 1200.8872 1202.1689

1210.0052 1217.6323 1218.9388

1227.4978 1243.4618 1246.3168

1248.4164 1259.3829 1262.5968

1265.9495 1280.5651 1281.5424

1283.4534 1289.0848 1292.7098

1300.9567 1301.6386 1306.9532

1313.3979 1315.3062 1323.8486

1324.9373 1330.6511 1331.2983

1337.7731 1341.4974 1352.2078

1361.4156 1366.3609 1366.6817

1367.2434 1383.3092 1384.1775

1386.7538 1408.0913 1408.9160

1411.9062 1415.8372 1420.8282

1424.9069 1430.4903 1435.4454

1463.0355 1466.8784 1473.6347

1475.9327 1479.4601 1485.4566

1486.9775 1493.6401 1495.7937

1500.2312 1501.6409 1501.9571

1505.2748 1507.0854 1508.8292

1512.8426 1524.9737 1526.7351

1529.6002 1530.7971 1542.5344

1552.7429 1561.9612 1562.7049

1674.0841 1676.1314 1690.0143

1691.4682 1693.9234 1695.8915

1700.6377 1700.8673 1702.9151

1706.1694 1710.9683 1759.6695

1817.9106 1884.4928 3058.1989

3068.2660 3069.1564 3072.7338

3073.9117 3081.4852 3083.6999

3085.6184 3136.0910 3136.1340

3138.2666 3140.6601 3144.3803

3148.6830 3151.0004 3155.7216

3157.6041 3165.0343 3166.6220

3186.3308 3192.9577 3194.0783

3194.8871 3195.4229 3197.1611

3199.2998 3199.3396 3203.9674

3206.0009 3211.1721 3211.3617

3217.0391 3218.1983 3219.7970

3225.9991 3229.2805 3233.3824

3234.6621 3235.0995 3241.6788

**PSS**

Zero-point correction= 0.436486

Thermal correction to Energy= 0.465134

Thermal correction to Enthalpy= 0.466079

Thermal correction to Gibbs Free Energy= 0.377635

Sum of electronic and zero-point Energies= -1771.281300

Sum of electronic and thermal Energies= -1771.252651

Sum of electronic and thermal Enthalpies= -1771.251707

Sum of electronic and thermal Free Energies= -1771.340150

Cartesian coordinates

O -2.193035 0.880607 -2.023782

O -0.128130 0.298116 1.909142

O -0.787938 2.281635 -3.172006

O -2.110705 -2.204193 1.664300

N 1.958596 -0.674289 2.017542

C 2.258636 -0.374631 3.399067

H 1.385234 0.121411 3.821949

H 3.126666 0.288831 3.461045

C 0.901925 -0.692911 -0.086430

C -1.081264 1.651897 -2.205065

C 4.240422 2.359084 -1.294363

H 5.068239 2.381633 -1.995579

C -0.472414 1.322660 -0.851545

H -0.427071 2.174159 -0.171558

C 2.830634 -1.316941 1.126071

C 2.244316 -1.379913 -0.141641

C 0.881766 0.593614 -0.960699

H 0.994143 0.233712 -1.992212

C 2.053601 1.516506 -0.674733

C 2.915974 -1.986973 -1.186000

H 2.472134 -2.044638 -2.176721

C 4.283410 3.129273 -0.135754

C 3.212467 3.094590 0.753580

H 3.235145 3.694646 1.657692

C 3.133405 1.557442 -1.558026

H 3.105856 0.953084 -2.460820

C 0.804954 -0.297682 1.392211

C -0.233479 -1.630772 -0.515649

H -0.032381 -1.930821 -1.549721

H -0.227502 -2.537814 0.096978

C 4.185845 -2.525806 -0.946982

H 4.726704 -3.007201 -1.754196

C 2.103781 2.297268 0.485580

H 1.276889 2.280766 1.191368

C 4.758071 -2.447776 0.319538

H 5.743816 -2.868799 0.487994

C -1.773314 0.497164 -0.674669

C 4.086633 -1.839607 1.384958

H 4.532675 -1.781087 2.371938

C -1.639297 -1.020892 -0.424112

C -2.757508 0.980691 0.357320

C -2.250268 -1.207768 1.001146

C -3.859002 0.255048 2.397207

H -4.032312 -0.515251 3.141290

C -3.370868 2.225196 0.423959

H -3.198577 2.981167 -0.336476

C -3.010960 0.014719 1.321614

C -4.471544 1.500665 2.471869

H -5.145288 1.725546 3.291789

C -4.231498 2.471969 1.491951

H -4.727848 3.434261 1.565703

C -2.549684 -1.788034 -1.384256

F -2.107173 -1.732594 -2.646910

F -3.799515 -1.309295 -1.366593

F -2.604864 -3.082907 -1.046267

H 5.144785 3.755724 0.072213

H 2.462512 -1.293940 3.954338

Vibrational frequencies

27.2563 32.8519 44.1626

52.3498 56.8083 71.9106

72.8462 77.8513 87.2838

111.3099 127.7119 133.2147

140.3886 154.7248 164.5716

174.1440 177.8476 187.1768

196.4658 231.7979 236.7589

247.0606 264.7798 274.4139

307.9203 318.5904 322.9863

336.6808 342.7339 363.2943

388.7972 395.5283 396.4750

418.6615 435.3240 455.0149

477.8418 499.3264 515.9743

525.2595 542.1672 546.1895

551.3021 559.4460 570.2534

590.3722 599.2793 618.5451

626.3825 633.1620 661.9777

684.8003 696.2065 707.4673

720.2059 721.5229 733.3114

754.8854 771.9174 773.5900

779.1227 783.1040 787.4490

807.7991 822.4775 855.4633

865.1844 877.2016 885.9703

889.6280 914.0374 921.6419

951.1405 953.1971 962.1388

986.5699 991.4120 995.4691

1001.5769 1011.3992 1014.3781

1015.9268 1020.7591 1026.3535

1027.8362 1038.3273 1061.1716

1063.6158 1075.9448 1084.4297

1099.6503 1122.0382 1124.8420

1134.0141 1154.2128 1157.0601

1162.3218 1168.9696 1178.9530

1184.1126 1184.5489 1185.7409

1200.9360 1215.7575 1223.7166

1238.3177 1243.0772 1253.9623

1263.1342 1275.3501 1281.3043

1293.6178 1299.3855 1310.9650

1319.9101 1329.7672 1332.7398

1343.2618 1354.0757 1363.6141

1367.6446 1385.2594 1392.4898

1408.1338 1413.0742 1436.0373

1464.0127 1491.1067 1501.1403

1511.8914 1515.7496 1528.4719

1532.2289 1537.7235 1557.0699

1561.8223 1680.5719 1693.2997

1695.8625 1702.2579 1705.2093

1709.3059 1822.6067 1890.3941

1979.2649 3058.8819 3071.5801

3090.2540 3145.1212 3146.5750

3161.0395 3193.2381 3193.6030

3198.9071 3203.0447 3211.2231

3213.9970 3218.8029 3222.1165

3222.2860 3230.4566 3232.0087

3233.8571 3242.8559 3243.4282

**TS5SS'_Φ= -22º_**

Zero-point correction= 0.807212

Thermal correction to Energy= 0.859405

Thermal correction to Enthalpy= 0.860349

Thermal correction to Gibbs Free Energy= 0.721910

Sum of electronic and zero-point Energies= -5393.978041

Sum of electronic and thermal Energies= -5393.925849

Sum of electronic and thermal Enthalpies= -5393.924904

Sum of electronic and thermal Free Energies= -5394.063343

Cartesian coordinates

O 2.838425 3.407595 0.069875

O 1.518416 -2.146527 0.880276

O -1.238693 1.309985 -2.404909

O 2.206863 -0.048048 3.178670

N 3.410052 -1.934927 -0.456814

C 4.018620 -3.198946 -0.139582

H 3.322629 -3.735331 0.507315

H 4.970142 -3.050863 0.386269

C 1.929439 -0.188169 -0.502608

C -1.606294 0.260473 -1.860220

C 2.803673 -3.530087 -3.695071

H 3.611399 -3.542994 -4.420253

C -0.876072 -0.939526 -1.719467

H -1.296768 -1.789827 -1.208502

C 3.920585 -0.992187 -1.331235

C 3.031401 0.114083 -1.378941

C 0.411259 -1.028563 -2.247398

H 0.752697 -0.176726 -2.831898

C 1.096412 -2.295716 -2.488811

C 3.322771 1.180156 -2.232535

H 2.651684 2.032926 -2.289842

C 2.431660 -4.699448 -3.038051

C 1.409710 -4.665456 -2.086027

H 1.135729 -5.569808 -1.551834

C 2.143697 -2.335685 -3.420153

H 2.438810 -1.418610 -3.924139

C 2.201262 -1.493618 0.088505

C 1.061504 0.771759 0.248630

H 0.572651 1.504266 -0.404914

H 0.295259 0.188846 0.777928

C 4.497449 1.146418 -2.985853

H 4.734410 1.976809 -3.643616

C 0.748923 -3.477736 -1.809758

H -0.010816 -3.452417 -1.034399

C 5.372328 0.059358 -2.904299

H 6.280548 0.055117 -3.498908

C 2.992662 2.377235 0.680384

C 5.090176 -1.031085 -2.076162

H 5.760861 -1.883141 -2.018728

C 1.866287 1.551652 1.339955

C 4.269519 1.652749 0.910706

C 2.651035 0.575224 2.246017

C 5.084078 -0.300903 2.098139

H 4.901297 -1.132176 2.771433

C 5.513211 1.883298 0.329976

H 5.655317 2.708484 -0.359331

C 4.061092 0.587893 1.782408

C 6.322091 -0.091122 1.500729

H 7.141229 -0.772885 1.706772

C 6.535738 0.991831 0.632016

H 7.513676 1.122359 0.180146

H 2.942061 -5.632951 -3.254435

H 4.193590 -3.783652 -1.048619

C -4.078958 -3.372088 1.172170

C -3.265134 -2.434434 0.545707

C -2.207611 -1.824407 1.208167

C -1.954200 -2.190130 2.522880

C -2.741213 -3.148176 3.164262

C -3.808315 -3.738971 2.489519

C -5.187919 -3.828245 0.258226

C -5.117736 -2.894574 -0.978405

C -3.742176 -2.135750 -0.857927

H -1.581014 -1.078447 0.732226

H -2.524109 -3.418648 4.191573

H -4.428857 -4.469833 2.998920

H -6.179605 -3.759192 0.716955

H -5.134565 -3.463799 -1.907590

H -5.048882 -4.869753 -0.047784

H -3.059564 -2.472096 -1.639488

N -3.910718 -0.683342 -1.010250

O -6.232167 -2.027678 -1.084398

C -3.026848 0.296857 -1.311448

C -5.054788 -0.075942 -0.572520

N -3.680529 1.435913 -1.062788

N -4.945283 1.218257 -0.606517

C -6.208885 -0.940716 -0.183485

H -6.102006 -1.272306 0.859514

H -7.143982 -0.390613 -0.287248

C -3.170909 2.788100 -1.025376

C -2.649523 3.223186 0.192735

C -3.276752 3.588057 -2.158247

C -2.208400 4.542523 0.253327

C -2.823822 4.900269 -2.044384

C -2.286100 5.390293 -0.853056

H -1.785724 4.911424 1.183876

H -2.884083 5.552456 -2.911808

Br -0.517162 -1.335031 3.383179

C -3.812036 3.028946 -3.445591

H -3.119957 2.267854 -3.819668

H -4.791391 2.561897 -3.302398

H -3.911926 3.812898 -4.197645

C -1.770776 6.802874 -0.760349

H -0.676601 6.809577 -0.744867

H -2.099170 7.402725 -1.611533

H -2.114540 7.287322 0.157285

C -2.562421 2.292357 1.372927

H -3.529017 1.821828 1.579297

H -1.831795 1.494318 1.194590

H -2.240475 2.826217 2.266620

C 0.959871 2.439068 2.160220

F 0.279613 3.302968 1.391483

F 0.056288 1.730952 2.852450

F 1.664848 3.160863 3.048515

Vibrational frequencies

-117.4792 -60.6323 17.6648

23.6081 31.0682 33.3243

37.7736 39.5808 48.9000

51.8772 56.5892 58.9335

60.6322 66.3312 68.9643

76.5103 83.5866 85.7032

86.6892 99.5179 103.1348

109.5037 113.2629 123.6276

126.2133 130.8505 140.2987

147.2296 149.5109 158.5051

159.3298 167.0844 168.1026

171.5948 178.3768 186.2574

203.0570 210.3161 217.8145

219.3139 221.7517 230.3577

233.2552 245.5055 250.7833

256.9489 263.5725 277.4059

284.1484 285.0675 290.5687

293.1986 302.5292 306.3167

320.2761 325.9105 327.8906

338.6769 342.4009 351.2574

355.1750 359.7363 385.8380

397.8241 403.4532 417.7657

419.2431 422.3454 447.1315

459.2866 466.5678 469.4268

479.8355 499.0305 503.7184

506.6137 513.5911 516.9898

521.7525 533.2322 536.5536

541.5856 546.3451 550.6223

557.5829 558.4772 572.4513

577.1580 580.7957 588.8824

593.2344 609.6239 619.4571

621.4837 625.5975 634.4420

649.9014 665.1011 669.5493

677.2633 679.8913 703.9767

717.7531 718.8001 720.5310

726.8257 727.7746 746.7789

747.4802 756.1534 756.6410

761.0495 762.6910 776.6664

782.8286 799.7411 801.1091

802.6979 808.6542 813.9583

836.3366 842.7151 853.4511

855.9828 861.0604 872.8520

877.8875 878.9980 887.0315

903.3880 908.1752 910.0102

910.9197 914.7537 919.0894

927.4006 929.4229 952.7731

965.2975 975.3286 977.5662

981.6508 983.7679 985.3511

991.0131 995.4762 1007.9346

1010.7500 1015.0932 1027.8495

1027.9782 1036.1887 1039.6219

1043.5533 1050.1376 1051.5399

1056.9355 1057.4154 1061.0889

1061.5576 1063.1284 1065.1618

1066.5469 1068.2868 1075.3649

1084.6873 1112.6677 1119.5297

1121.6248 1130.4126 1133.1278

1133.8225 1144.1392 1154.3669

1157.4262 1159.9068 1172.6628

1178.2175 1182.5422 1186.7717

1199.8954 1201.2930 1209.0403

1210.4623 1221.9130 1225.4577

1229.8233 1242.2054 1248.3345

1268.0518 1268.4968 1269.1365

1273.3825 1274.8402 1281.6486

1286.5174 1287.8797 1302.3257

1310.3990 1318.4528 1319.3930

1320.0783 1323.5695 1329.5616

1342.2196 1343.5037 1346.8070

1356.2461 1360.1726 1366.2596

1367.3964 1377.5205 1379.9720

1385.4300 1395.8909 1401.5087

1411.6706 1416.8051 1421.0782

1427.0813 1428.5824 1436.5334

1458.2113 1459.4379 1471.6061

1472.4161 1477.5584 1484.1535

1487.2635 1494.8960 1496.4164

1498.5647 1501.6085 1502.1475

1503.1972 1504.1402 1511.8879

1515.5732 1522.1300 1527.8197

1529.6176 1534.0754 1535.6615

1539.3214 1542.4450 1545.5757

1560.2620 1574.6351 1662.5650

1664.1059 1673.9282 1686.7409

1687.0314 1687.7104 1690.5567

1691.4637 1693.1309 1696.9979

1701.6772 1707.0751 1755.0437

1863.2477 1900.8962 3046.5374

3061.7719 3062.6274 3066.0336

3070.2727 3071.9858 3085.7112

3117.4135 3127.8149 3129.9512

3130.7903 3140.4345 3144.6929

3146.5286 3158.9842 3166.5281

3175.4060 3178.8535 3179.6518

3189.7090 3192.1147 3196.1392

3200.1570 3200.8712 3204.2612

3205.6393 3206.8149 3208.6718

3210.1956 3211.5260 3213.9044

3215.3868 3224.4380 3224.9097

3226.7475 3227.5734 3228.1582

3242.9784 3244.5821 3307.7148

**TS5SS'_Φ= 73º_**

Zero-point correction= 0.810242

Thermal correction to Energy= 0.863604

Thermal correction to Enthalpy= 0.864549

Thermal correction to Gibbs Free Energy= 0.720712

Sum of electronic and zero-point Energies= -5393.970241

Sum of electronic and thermal Energies= -5393.916878

Sum of electronic and thermal Enthalpies= -5393.915934

Sum of electronic and thermal Free Energies= -5394.059770

Cartesian coordinates

O -2.346283 -0.366352 -2.782771

O -3.645698 -0.143597 2.693923

O 1.418905 1.986922 1.924782

O -5.255353 -1.646670 0.639902

N -3.003343 1.893882 1.828132

C -3.940798 2.722397 2.545727

H -4.509886 2.070005 3.208922

H -4.623983 3.217184 1.846720

C -1.779119 0.026844 1.082535

C 1.615380 0.831012 1.469274

C -1.475703 -2.023987 5.317268

H -1.870474 -1.782264 6.299179

C 0.771943 -0.257834 1.450300

H 1.064887 -1.199359 1.016821

C -2.191059 2.330834 0.778774

C -1.490618 1.243038 0.240940

C -0.561984 -0.157868 2.116610

H -0.542604 0.785107 2.676143

C -0.787059 -1.289992 3.106927

C -0.692449 1.422365 -0.874977

H -0.203748 0.575108 -1.350776

C -1.154789 -3.339990 4.998143

C -0.646997 -3.633956 3.735128

H -0.394405 -4.657182 3.474231

C -1.294269 -1.012857 4.378059

H -1.553031 0.011674 4.630699

C -2.941258 0.520668 1.960573

C -2.059122 -1.282776 0.338276

H -1.146454 -1.578854 -0.198716

H -2.285987 -2.054100 1.080519

C -0.589284 2.702821 -1.428503

H 0.017146 2.859031 -2.314432

C -0.458558 -2.617178 2.803493

H -0.052818 -2.865783 1.824823

C -1.261330 3.780295 -0.856699

H -1.153760 4.769030 -1.292583

C -3.073065 -0.279323 -1.822877

C -2.084091 3.611057 0.259654

H -2.626779 4.444376 0.693504

C -3.218599 -1.323256 -0.692488

C -4.024243 0.823008 -1.528025

C -4.572876 -0.937631 -0.053378

C -5.867901 1.309953 -0.025736

H -6.525725 1.004809 0.781738

C -4.120527 2.070245 -2.137152

H -3.438339 2.349031 -2.933176

C -4.882137 0.448548 -0.496245

C -5.957915 2.565437 -0.619163

H -6.707827 3.270291 -0.273770

C -5.096310 2.939747 -1.662468

H -5.195306 3.927954 -2.099959

H -1.298518 -4.130588 5.727660

H -3.415861 3.479922 3.134762

C 3.678309 -3.683608 -0.564109

C 2.995921 -2.508731 -0.272338

C 2.034123 -1.989233 -1.128298

C 1.718123 -2.708828 -2.271053

C 2.363175 -3.908451 -2.572344

C 3.355632 -4.392086 -1.720584

C 4.726188 -3.971742 0.482069

C 4.788645 -2.697269 1.365364

C 3.488684 -1.885389 1.013467

H 1.532259 -1.050459 -0.918997

H 2.097685 -4.451838 -3.471949

H 3.875238 -5.312223 -1.969309

H 5.713813 -4.174254 0.055849

H 4.791021 -2.946756 2.426341

H 4.452684 -4.840217 1.089502

H 2.757268 -1.943378 1.824002

N 3.782114 -0.471492 0.788009

O 5.981920 -1.954833 1.180527

C 2.990979 0.624997 0.860601

C 4.955759 -0.098685 0.192245

N 3.731977 1.603894 0.329041

N 4.959270 1.167997 -0.088886

C 6.007011 -1.146660 0.020652

H 5.817542 -1.732151 -0.890788

H 6.992661 -0.687212 -0.050882

C 3.332008 2.945655 -0.018046

C 2.834453 3.138823 -1.306944

C 3.521572 3.975173 0.899564

C 2.496294 4.440407 -1.669448

C 3.170735 5.259256 0.487559

C 2.653539 5.508145 -0.784132

H 2.103195 4.624034 -2.667274

H 3.299944 6.084440 1.183191

Br 0.367659 -2.000276 -3.380762

C 4.045065 3.686851 2.276544

H 3.303596 3.078228 2.803230

H 4.985342 3.128034 2.232980

H 4.217892 4.612139 2.828573

C 2.253044 6.901048 -1.197093

H 1.162792 6.996890 -1.221933

H 2.638202 7.647434 -0.499502

H 2.626345 7.138875 -2.196770

C 2.677705 1.980197 -2.256698

H 3.632209 1.470186 -2.420193

H 1.969847 1.242602 -1.860722

H 2.302498 2.320495 -3.223635

C -3.299218 -2.724303 -1.274663

F -2.168115 -3.069400 -1.902376

F -3.522087 -3.642999 -0.328979

F -4.300101 -2.807318 -2.166427

Vibrational frequencies

8.9132 13.9308 23.7797

29.5383 30.6625 39.3594

40.5648 43.3865 47.3608

52.0564 58.7781 60.1316

66.3099 67.3785 71.8126

76.4936 81.1339 83.8891

87.4454 97.8135 101.1841

111.8949 117.9425 123.9794

128.8447 134.2855 142.1186

146.5686 153.5834 155.7538

164.5490 165.6593 179.8404

183.4382 194.1631 195.6416

202.7379 202.9503 216.2205

218.0068 226.5997 230.0282

243.8447 246.6613 249.8162

255.7402 263.5663 271.9995

278.5136 281.4624 294.8016

300.8323 311.9850 315.3804

324.8295 326.4611 328.8822

340.6030 348.0257 356.1036

361.2882 395.0876 401.8780

410.5738 415.0159 421.4436

426.1819 449.5875 456.2678

461.9816 479.4052 484.9087

500.2990 500.7742 514.1696

520.8577 521.5136 522.8372

536.3330 538.5156 542.7817

547.1803 549.4100 554.0872

556.2118 569.5407 576.7701

589.3442 589.7160 598.8089

617.4003 619.7568 628.8416

633.0881 651.2196 658.1439

662.5956 669.3901 677.1708

689.6921 699.6123 712.5689

718.0304 724.9267 729.2994

731.7497 739.1720 747.1739

753.6980 757.4199 760.2748

763.8358 778.0213 780.3206

793.1529 802.5282 804.6459

812.2622 830.3842 832.4202

847.3169 850.3923 868.8494

875.3024 875.9113 877.9952

880.9921 889.0440 903.5246

905.3214 908.2444 909.8118

912.9179 919.8306 942.8675

962.7598 963.2718 969.7276

981.6531 982.8257 986.4240

989.9904 992.0844 993.4332

1006.0368 1013.9015 1016.5428

1019.7397 1022.1789 1032.7905

1037.6730 1037.9910 1039.6197

1049.9570 1051.1193 1057.8232

1059.0683 1061.6715 1064.4137

1065.5840 1066.7515 1069.5838

1073.4529 1083.4280 1092.0062

1109.0463 1118.7487 1119.5881

1127.3970 1130.8124 1135.2755

1151.7470 1155.0647 1157.0821

1159.3596 1165.7207 1178.1818

1179.5939 1183.5411 1193.6745

1196.9189 1203.5481 1204.4574

1210.1558 1222.0240 1227.0861

1233.0921 1247.2044 1251.2001

1262.0948 1275.2006 1278.5663

1280.5975 1282.3491 1285.8100

1287.2179 1295.4128 1298.0811

1304.3041 1315.5156 1319.7846

1324.7916 1329.3234 1335.2917

1338.0252 1343.3750 1346.8514

1349.7670 1356.2015 1358.5600

1369.9411 1370.7988 1379.8335

1385.5946 1394.5902 1401.8852

1404.0726 1406.4186 1413.9610

1422.7841 1427.0733 1431.0902

1458.7385 1459.8924 1470.0199

1476.4006 1479.5333 1485.5439

1488.5429 1491.4953 1496.8652

1499.6578 1499.9271 1501.2457

1501.7684 1504.8488 1505.2931

1510.7261 1515.1421 1521.3787

1527.1699 1527.7417 1529.1735

1536.1360 1547.4488 1549.2256

1557.0906 1566.0354 1675.1514

1679.1633 1687.8074 1689.4803

1690.8257 1691.6705 1694.8818

1695.7310 1696.5737 1701.2734

1706.4978 1710.1800 1846.3326

1871.1841 1912.5322 3054.5361

3062.4892 3063.8897 3066.2985

3069.2337 3070.9272 3086.1057

3093.9357 3132.4309 3134.2748

3134.7858 3138.6335 3139.0394

3141.6965 3144.8100 3166.8508

3169.2006 3170.6643 3171.8229

3177.9364 3185.2724 3187.3596

3188.3209 3192.9435 3199.8890

3201.0015 3210.0133 3212.3771

3213.9775 3215.9193 3216.2639

3219.9860 3220.4160 3223.1065

3228.5925 3230.0083 3232.7794

3239.5905 3244.6404 3311.4260

**TS5SS'_Φ= -82º_**

Zero-point correction= 0.808395

Thermal correction to Energy= 0.860899

Thermal correction to Enthalpy= 0.861844

Thermal correction to Gibbs Free Energy= 0.723144

Sum of electronic and zero-point Energies= -5393.974044

Sum of electronic and thermal Energies= -5393.921539

Sum of electronic and thermal Enthalpies= -5393.920595

Sum of electronic and thermal Free Energies= -5394.059295

Cartesian coordinates

O 5.116042 1.549658 -0.359134

O 0.401648 -0.619323 0.896597

O -1.023042 1.728215 -2.586447

O 1.214943 2.352381 2.053954

N 2.034436 -2.170781 0.344068

C 1.643061 -3.213609 1.257776

H 0.833845 -2.823565 1.879359

H 2.485619 -3.492667 1.898926

C 1.998249 -0.234008 -0.896567

C -1.405320 0.725176 -1.955657

C 2.201647 -3.753978 -3.947702

H 2.932129 -3.926117 -4.731631

C -0.792909 -0.515241 -1.783841

H -1.254207 -1.244902 -1.136604

C 3.110064 -2.229247 -0.535521

C 3.169123 -1.019796 -1.263951

C 0.478912 -0.806447 -2.344126

H 0.842225 -0.068663 -3.058936

C 0.858319 -2.200596 -2.648378

C 4.201980 -0.825935 -2.174707

H 4.281486 0.107750 -2.725029

C 1.687795 -4.823670 -3.220277

C 0.754862 -4.585903 -2.211168

H 0.346930 -5.415662 -1.641144

C 1.790496 -2.456847 -3.661326

H 2.201558 -1.622086 -4.221778

C 1.367700 -0.962165 0.210222

C 1.923985 1.268556 -0.933063

H 2.293178 1.638834 -1.895317

H 0.883473 1.598061 -0.827284

C 5.148662 -1.837336 -2.353027

H 5.962190 -1.691272 -3.055874

C 0.343310 -3.288496 -1.928989

H -0.367739 -3.119482 -1.122985

C 5.058744 -3.033204 -1.639177

H 5.800110 -3.809830 -1.798758

C 4.150700 1.328789 0.328852

C 4.033547 -3.247137 -0.712980

H 3.963431 -4.174843 -0.153688

C 2.759510 1.983892 0.181976

C 4.058295 0.382347 1.474373

C 2.131958 1.737372 1.566902

C 2.517661 -0.172031 3.269647

H 1.575702 0.002209 3.778695

C 4.911362 -0.664370 1.812778

H 5.804002 -0.860289 1.227385

C 2.890801 0.623809 2.192089

C 3.361956 -1.222786 3.608059

H 3.098997 -1.881433 4.430072

C 4.544183 -1.465677 2.888386

H 5.171413 -2.305165 3.171219

H 2.009407 -5.837366 -3.437437

H 1.298957 -4.099297 0.710434

C -4.448862 -3.005068 0.558068

C -3.502076 -2.047723 0.204656

C -2.506662 -1.633097 1.083344

C -2.480878 -2.222296 2.341055

C -3.394209 -3.208444 2.711097

C -4.385315 -3.603763 1.813864

C -5.459331 -3.193155 -0.546419

C -5.242503 -1.990174 -1.495546

C -3.795808 -1.476889 -1.169614

H -1.766683 -0.878852 0.829994

H -3.341654 -3.644498 3.702316

H -5.113556 -4.352278 2.110606

H -6.494237 -3.206831 -0.192582

H -5.310810 -2.283447 -2.543436

H -5.285229 -4.131644 -1.083373

H -3.082897 -1.793824 -1.934283

N -3.762200 -0.015751 -1.098684

O -6.235982 -0.988250 -1.347363

C -2.759291 0.871091 -1.277834

C -4.799533 0.657709 -0.510879

N -3.230590 2.022988 -0.801047

N -4.504007 1.908424 -0.322505

C -6.056789 -0.108654 -0.253333

H -5.990486 -0.647050 0.702294

H -6.911401 0.566872 -0.223328

C -2.463041 3.206771 -0.501133

C -1.725528 3.189336 0.685539

C -2.505346 4.283107 -1.380700

C -1.002572 4.340519 0.986926

C -1.765738 5.410639 -1.030095

C -1.010122 5.452143 0.142446

H -0.390692 4.349345 1.883881

H -1.766655 6.268336 -1.697774

Br -1.195120 -1.611782 3.589557

C -3.272784 4.184789 -2.668834

H -2.832804 3.391483 -3.282021

H -4.323749 3.937510 -2.489465

H -3.228150 5.125395 -3.219950

C -0.174244 6.657149 0.486526

H -0.407522 7.025778 1.489462

H 0.886360 6.389481 0.471944

H -0.333107 7.470063 -0.224963

C -1.678856 1.967585 1.567597

H -2.667285 1.505020 1.670347

H -0.980555 1.223110 1.162637

H -1.314456 2.228331 2.561618

C 2.874327 3.460937 -0.124789

F 1.673302 4.044269 -0.212991

F 3.507587 3.678458 -1.284160

F 3.564528 4.099095 0.836376

Vibrational frequencies

-240.0902 19.1964 22.2140

32.2003 33.7496 35.6736

39.7839 49.1273 56.4314

62.5474 62.9526 65.0834

67.4853 71.9292 76.8366

81.7472 84.3385 87.6445

94.5075 96.7265 105.8796

107.6573 115.5083 124.2513

136.1361 138.9036 143.8721

150.7811 158.4367 159.5671

165.5912 175.2248 179.7213

186.5293 200.0495 201.7091

209.2033 217.5035 221.4154

224.7187 229.9325 242.1398

248.1558 252.3072 256.9601

266.5974 274.0369 275.9314

283.8514 289.7987 290.1316

297.6597 298.6184 308.3704

319.3789 323.6923 328.2311

340.0150 348.0268 349.1926

358.6008 364.5700 383.4543

398.9936 411.1720 415.6535

422.0777 423.3000 440.0845

457.9895 458.9280 476.1718

489.5528 501.0065 505.2060

511.8853 515.0954 522.9828

525.7845 537.8925 538.5514

540.8626 546.3971 549.5509

557.1657 559.3576 577.2565

583.4288 586.4225 589.6791

592.6532 613.0677 614.0215

620.7260 629.3814 632.9223

647.3797 663.8127 673.6786

676.2583 686.1866 701.5053

718.3871 720.4845 727.9560

730.7881 733.9366 748.0103

750.8142 753.3505 757.0248

757.9184 770.8638 778.1251

786.7516 790.9011 795.7510

802.6380 805.2377 811.5266

829.0053 845.5074 847.7834

854.9545 869.7780 870.6134

877.4305 883.4781 887.6384

893.9194 900.2505 905.8699

913.6041 918.9714 920.4149

927.4500 945.5562 960.9145

964.4755 976.0195 977.4190

982.8244 985.0386 988.3027

991.8100 1001.4612 1007.6923

1015.5411 1020.6303 1020.7749

1033.5942 1035.8460 1043.5847

1048.4786 1051.7607 1052.9654

1056.6227 1060.6763 1062.1903

1062.8226 1064.6899 1067.4093

1070.2687 1071.2203 1074.5289

1091.4063 1116.5085 1118.6014

1121.1196 1126.5566 1129.2416

1133.6998 1150.5598 1152.7173

1158.2868 1160.7592 1168.9904

1181.1646 1183.3456 1187.0037

1192.5597 1207.4525 1208.3892

1210.9996 1225.7669 1227.2489

1228.9753 1241.1432 1247.1794

1261.1598 1271.0191 1272.7881

1277.5196 1277.8935 1284.2044

1288.6001 1289.6162 1297.6450

1313.2574 1314.1163 1317.7856

1323.6490 1325.0946 1333.5926

1337.5986 1343.7635 1350.4312

1361.1655 1366.0529 1367.5691

1369.0816 1375.8479 1379.5496

1381.5317 1396.8051 1405.1866

1407.0196 1419.0242 1419.2981

1423.9017 1430.0558 1435.1652

1461.8203 1463.0603 1469.9957

1470.4995 1475.0926 1480.4862

1488.2286 1492.5235 1494.3957

1498.1211 1501.0399 1502.6726

1507.0578 1508.9147 1509.6076

1518.6202 1524.2007 1526.5255

1529.4806 1533.6175 1534.8541

1540.7759 1544.0042 1549.8205

1565.5719 1574.1683 1669.9292

1673.0136 1676.5807 1688.1281

1688.7213 1689.4218 1693.2886

1695.1107 1695.5888 1697.7763

1702.5131 1709.6648 1760.7895

1866.6873 1905.8742 3052.8117

3056.3690 3063.0480 3070.9797

3071.7201 3083.2252 3084.5627

3122.4651 3125.5005 3137.3754

3137.6826 3142.0333 3143.4519

3145.8110 3162.7630 3162.8857

3166.3077 3173.1575 3173.5703

3181.8203 3183.2776 3190.2387

3196.4607 3197.1346 3200.2501

3200.3587 3206.7864 3207.1327

3208.5889 3212.6120 3214.6818

3224.0769 3225.2746 3230.0581

3230.9297 3231.0957 3232.4946

3234.3856 3237.0503 3290.0748

**TS5SS'_Φ= 152º_**

Zero-point correction= 0.807589

Thermal correction to Energy= 0.859870

Thermal correction to Enthalpy= 0.860814

Thermal correction to Gibbs Free Energy= 0.721074

Sum of electronic and zero-point Energies= -5393.984534

Sum of electronic and thermal Energies= -5393.932253

Sum of electronic and thermal Enthalpies= -5393.931309

Sum of electronic and thermal Free Energies= -5394.071049

Cartesian coordinates

O 4.274583 -1.696559 2.109171

O 2.928863 1.579715 -1.969766

O -0.605102 1.781584 -2.351074

O 5.950901 0.782612 -1.478600

N 1.505772 1.782759 -0.148434

C 1.203345 3.185029 -0.286746

H 1.898779 3.589253 -1.023993

H 1.343070 3.696049 0.672069

C 2.275275 -0.337897 -0.615625

C -1.098620 0.831658 -1.713784

C 2.022476 -4.123991 -3.294118

H 2.647737 -4.462454 -4.114118

C -0.567638 -0.422147 -1.423503

H -1.100887 -1.073202 -0.741516

C 1.011818 0.936597 0.830774

C 1.541759 -0.359782 0.638027

C 0.677468 -0.853490 -1.971405

H 1.056695 -0.236493 -2.786338

C 0.933293 -2.302804 -2.109783

C 1.203311 -1.375088 1.524015

H 1.628953 -2.367778 1.406951

C 1.510484 -5.041119 -2.378970

C 0.706800 -4.591871 -1.332770

H 0.305460 -5.299945 -0.614342

C 1.738007 -2.769249 -3.157983

H 2.142816 -2.054820 -3.870173

C 2.316234 1.083753 -1.032740

C 3.440892 -1.223927 -0.952873

H 3.190329 -2.271316 -0.749922

H 3.677242 -1.130891 -2.018632

C 0.331198 -1.094023 2.579421

H 0.049704 -1.882832 3.267810

C 0.414484 -3.238383 -1.200571

H -0.203989 -2.906365 -0.371304

C -0.187483 0.192253 2.752805

H -0.872359 0.385622 3.574203

C 4.432666 -0.781128 1.339052

C 0.159865 1.233436 1.885599

H -0.218686 2.241946 2.022515

C 4.748564 -0.904744 -0.163981

C 4.339705 0.672244 1.639762

C 5.281718 0.499977 -0.517792

C 4.810930 2.803821 0.578657

H 5.178521 3.363921 -0.274515

C 3.797307 1.290164 2.762218

H 3.388378 0.695749 3.573130

C 4.833673 1.413104 0.569743

C 4.267177 3.427379 1.696342

H 4.217335 4.511038 1.736361

C 3.763040 2.679808 2.773368

H 3.330566 3.199852 3.622249

H 1.735669 -6.097831 -2.479844

H 0.180825 3.326391 -0.648238

C -4.717811 -3.046614 -0.329485

C -3.749619 -2.051748 -0.205870

C -3.132358 -1.773228 1.010923

C -3.523577 -2.534022 2.110114

C -4.470762 -3.551416 2.003626

C -5.071095 -3.813376 0.775340

C -5.269863 -3.090556 -1.731847

C -4.839529 -1.747816 -2.356235

C -3.577256 -1.327499 -1.528872

H -2.384932 -0.989810 1.123314

H -4.745987 -4.119808 2.884635

H -5.823852 -4.591093 0.693954

H -6.357098 -3.195022 -1.772687

H -4.586472 -1.847980 -3.412441

H -4.834118 -3.922782 -2.295680

H -2.658262 -1.608735 -2.049404

N -3.554831 0.118292 -1.326675

O -5.879196 -0.779838 -2.328842

C -2.551778 1.021295 -1.287954

C -4.718771 0.774887 -1.033463

N -3.152961 2.174405 -0.974235

N -4.501951 2.036781 -0.814893

C -5.988407 -0.004979 -1.148182

H -6.150487 -0.625369 -0.257104

H -6.832973 0.672367 -1.273109

C -2.529759 3.421314 -0.610308

C -2.338084 3.650098 0.756812

C -2.207375 4.345362 -1.601667

C -1.708391 4.837392 1.118839

C -1.579722 5.520118 -1.183197

C -1.307092 5.772859 0.160580

H -1.532899 5.040738 2.172854

H -1.296811 6.254471 -1.932746

Br -2.793728 -2.162618 3.817404

C -2.524815 4.088501 -3.047436

H -1.901073 3.270163 -3.415294

H -3.575618 3.806982 -3.168861

H -2.336535 4.982748 -3.643548

C -0.604113 7.035458 0.586208

H 0.338469 6.800766 1.089993

H -0.380719 7.673623 -0.270524

H -1.217253 7.606746 1.289183

C -2.847301 2.677384 1.789026

H -3.941123 2.692996 1.819127

H -2.529353 1.651450 1.575787

H -2.474247 2.941274 2.780857

C 5.786112 -1.971579 -0.437029

F 5.369171 -3.182656 -0.044781

F 6.077548 -2.056805 -1.740200

F 6.928257 -1.707458 0.219466

Vibrational frequencies

-248.4659 -76.8148 16.8525

20.3103 23.5997 29.1064

38.1492 39.1749 45.2194

45.6228 50.8857 53.6310

59.9484 61.7437 71.0384

77.1135 79.2360 84.6295

94.5065 96.1506 102.7904

104.8910 111.8312 114.4097

124.3329 131.8896 137.7137

147.2020 148.4411 152.7747

156.3790 157.2457 167.2260

175.0923 181.1121 194.6853

201.1798 207.2316 208.0938

221.1809 222.0187 228.1082

233.3423 247.5520 248.2112

251.2143 268.3391 274.1353

281.1763 283.5669 288.5624

294.6603 298.6432 306.6476

317.5218 326.6896 332.7476

335.2824 347.1736 350.4000

352.3598 360.0979 384.6851

399.9428 413.4602 415.3881

420.7859 425.4677 430.8239

447.9890 459.5569 480.3492

497.9005 500.6665 505.2441

513.1010 513.8959 526.0642

527.5282 536.6264 538.7470

543.2927 546.6686 550.2555

557.9839 559.8852 577.9279

582.7007 585.2224 589.6701

601.6293 609.3639 614.0216

619.7706 629.9151 634.4715

653.0934 662.3500 672.0761

677.4701 689.0683 716.4764

720.9376 721.4241 727.0981

735.6517 741.4153 750.7966

752.8642 756.3279 758.2873

759.0160 761.7986 779.2944

783.9463 797.8017 801.0481

801.6481 806.8179 813.7254

833.1638 848.0163 854.7771

870.8776 871.9089 876.8754

877.8422 882.6274 887.5536

904.2117 904.3419 907.6799

911.2399 915.2222 919.2232

937.9323 946.7210 947.7327

954.7737 963.6642 969.7280

982.9958 986.3791 993.9754

1006.5365 1012.4688 1014.9801

1021.0411 1021.9639 1024.5636

1035.4915 1036.9266 1040.7691

1045.7530 1048.7794 1049.4361

1056.2309 1059.5819 1060.6788

1063.2086 1063.4893 1069.5933

1071.6166 1073.5580 1076.1833

1092.1747 1106.1593 1116.4967

1117.6498 1126.7570 1128.9125

1136.6527 1150.0682 1151.2320

1156.4970 1160.1087 1167.7025

1182.2996 1183.5660 1184.4904

1194.4440 1204.4381 1208.2436

1211.2460 1223.3269 1226.1272

1232.6711 1238.6664 1249.2767

1251.3720 1267.0357 1276.2648

1276.6220 1279.2924 1281.4622

1284.2380 1290.3238 1291.1127

1305.4650 1310.9371 1315.3235

1323.4989 1326.2252 1330.9512

1336.6916 1340.3813 1352.3362

1361.9716 1366.0658 1367.2827

1371.1275 1375.9632 1377.5493

1381.1711 1396.5176 1404.4423

1411.4128 1417.9967 1419.1969

1425.3938 1429.1580 1432.1820

1459.9291 1461.5798 1467.9498

1471.0300 1471.3164 1479.1063

1484.3281 1485.1078 1491.1545

1491.7434 1493.9089 1497.8564

1501.0408 1504.3772 1507.6069

1510.4865 1520.5873 1525.2668

1526.6894 1529.4663 1529.9154

1534.9355 1541.6879 1555.1557

1564.8356 1568.3985 1668.5706

1670.0916 1671.2742 1686.9006

1687.8469 1688.0825 1691.1969

1693.0543 1696.0429 1697.3127

1698.2757 1713.8278 1800.0494

1873.2591 1912.3684 3064.2360

3067.4757 3069.2412 3071.2842

3085.1008 3087.2376 3088.3857

3133.7759 3136.8350 3137.2174

3144.6866 3146.9452 3151.6755

3157.9085 3158.8575 3159.5918

3169.3850 3169.6019 3180.1269

3182.3606 3190.1159 3192.0020

3192.9542 3194.2721 3197.2085

3201.4879 3205.1046 3206.4833

3214.4731 3214.9777 3218.5262

3218.6857 3224.7272 3225.5208

3227.0199 3228.7919 3234.6424

3235.3227 3238.4726 3253.3497
